# Classification of Gene Variants in a Danish Population with Suspected Predisposition to Hereditary Breast and/or Ovarian Cancer

**DOI:** 10.3390/cancers17111819

**Published:** 2025-05-29

**Authors:** Anne K. Munch, Elisabeth S. Feldner, Caroline H. Bækgaard, Mie B. Larsen, Naja Slemming-Adamsen, Desirée S. Boonen, Nanna B. Møller, Inge S. Pedersen, Thomas V. O. Hansen, Thorkild Terkelsen, Mark Burton, Qin Hao, Susanne E. Boonen, Mads Thomassen

**Affiliations:** 1 Department of Clinical Genetics, Odense University Hospital, J.B. Winslows Vej 4, Entrance 24, 5000 Odense, Denmark; anne.kulmback.munch@rsyd.dk (A.K.M.); elisabeth.simone.feldner.01@regionh.dk (E.S.F.); caroline.hey.baekgaard@rsyd.dk (C.H.B.); mie.bohnensack.larsen@rsyd.dk (M.B.L.); najsen07@gmail.com (N.S.-A.); desiree@boonen.dk (D.S.B.); mark.burton@rsyd.dk (M.B.); qin.hao@rsyd.dk (Q.H.); susanne.eriksen.boonen@rsyd.dk (S.E.B.); 2 Department of Clinical Genetics, Aarhus University Hospital, Brendstrupgårdsvej 21C, 8200 Aarhus, Denmark; nannabakmoller@gmail.com (N.B.M.); thorkild.terkelsen@clin.au.dk (T.T.); 3 Department of Molecular Diagnostics, Aalborg University Hospital, Reberbansgade 15, 9000 Aalborg, Denmark; isp@rn.dk; 4 Department of Clinical Medicine, Aalborg University, Selma Lagerløfsvej 249, 9260 Gistrup, Denmark; 5 Clinical Cancer Research Center, Sdr. Skovvej 15, 9000 Aalborg, Denmark; 6 Department of Clinical Genetics, Rigshospitalet, Copenhagen University Hospital, Blegdamsvej 9, 2100 Copenhagen, Denmark; thomas.van.overeem.hansen@regionh.dk; 7 Department of Clinical Medicine, University of Copenhagen, Blegdamsvej 3B A, 2200 Copenhagen, Denmark; 8 Department of Biomedicine, Aarhus University, Høegh-Guldbergsgade 10, 8000 Aarhus, Denmark; 9 Department of Clinical Research, University of Southern Denmark, Campusvej 55, 5230 Odense, Denmark; 10 Clinical Genome Center, Odense University Hospital, University of Southern Denmark, J.B. Winslows Vej 15, 5000 Odense, Denmark

**Keywords:** hereditary, breast cancer, ovarian cancer, genetics, variant of unknown significance, association analysis, splice analysis, RNA sequencing

## Abstract

Background: Gene variants of unknown significance (VUSs) present a challenge in genetic counselling. The primary aim of this study was to describe the spectrum of genetic findings in a cohort of 5923 Danish patients with suspected predisposition to hereditary breast and/or ovarian cancer, with a focus on classifying gene variants and investigating their distribution. Methods: The gene variants were classified using the American College of Medical Genetics (ACMG) guidelines as well as gene-specific guidelines where applicable. The identified VUSs were further examined through association analysis, comparison of the frequencies in this Danish population to those in the Swedish population using gnomAD 2.1, and splice analysis using RNA sequencing. Results: Of 167 variants that were clinically classified as VUSs prior to this research study, 38 (22.8%) were either up- or downgraded based on the guidelines that were used. We found that 630 patients (10.6%) carried a likely pathogenic or pathogenic variant, mainly in *BRCA1* (31.9%) and *BRCA2* (26.0%). VUSs were carried by 1606 (27.1%) patients, mainly in *BARD1* (27.6%) and *ATM* (19.3%). Our association study assigned criteria for 10 gene variants, while our splice analysis assigned criteria for 3 gene variants but did not reclassify the variants. Conclusions: A total of 22.8% of the 167 variants that were observed in this study and which were previously classified as VUSs in a clinical setting were reclassified in this study. In total, 10.6% of the patients with a suspected predisposition to hereditary breast and/or ovarian cancer carried a likely pathogenic or pathogenic variant. The high incidence of VUSs observed in this study reflects the challenges faced in the daily clinical setting.

## Simple Summary

This study aimed to classify and investigate the distribution of gene variants in 13 clinically relevant genes of 5923 Danish patients with suspected hereditary predisposition to breast and/or ovarian cancer, all of whom were tested with the same gene panel. The growth in genetic analysis over the last 25 years has generated an increasing number of variants of unknown significance (VUSs). These present challenges for daily clinical counselling and decision-making about whether a carrier should be offered inclusion in a surveillance program or risk-reducing surgery. We examined VUSs using two methods: an association analysis comparing the case group to a Swedish control group, and splice analysis using RNA sequencing.

## 1. Introduction

Breast cancer is the most frequent cancer in Danish women, with around 4850 Danish women being diagnosed every year [1]. Ovarian cancer is the most lethal gynecologic cancer and is often diagnosed at an advanced stage [2]. Around 550 Danish women are diagnosed with ovarian cancer every year [3].

It is estimated that 5–10% of breast and ovarian cancers can be explained by monogenetic causes, and patients with a suspected hereditary predisposition to breast and/or ovarian cancer are often referred for genetic risk assessment [4]. The risk of developing breast and/or ovarian cancer (BC/OC) depends on the patient’s family history, environmental factors, and/or the presence of variants in susceptibility genes. Pathogenic variants in BC/OC susceptibility genes with a high penetrance (*BRCA1*, *BRCA2*, *CDH1*, *PALB2*, *PTEN*, *STK11*, and *TP53*) lead to a significantly increased risk of developing BC and/or OC. Genes with a moderate penetrance (*ATM*, *BARD1*, *BRIP1*, *CHEK2*, *RAD51C*, and *RAD51D*) induce a moderately increased risk of developing BC and/or OC. Based on their cancer risk assessment, some gene variant carriers and their relatives are offered either surveillance or risk-reducing surgery [5]. Additionally, carriers of a *BRCA1* or *BRCA2* gene mutation may be treated with PARP inhibitors [6,7]. A Danish study on the distribution of gene variants in patients with a suspected predisposition to breast and/or ovarian cancer has not been published since 2008 [8,9], and genetic testing technologies have changed significantly since then.

The implementation of next-generation sequencing (NGS) has made genetic testing faster and cheaper, thus increasing the number of patients who are tested. This has led to the greater detection of variants, including variants of unknown clinical significance (VUSs) [10]. A gene variant is classified as a VUS when the criteria for classification conflict with each other or when the gene’s attributes are insufficient for a likely pathogenic/pathogenic or a likely benign/benign classification [11]. The reclassification of VUSs is important to improving patient management, but the systematic reevaluation of VUSs is often not performed in the clinical setting due to limited resources. The current study aims to address this gap between the literature and the clinical setting.

The aim of this register-based study was to classify and investigate the distribution of gene variants in a Danish population with suspected predisposition to hereditary breast and/or ovarian cancer. The specific objectives were as follows:

1. To classify gene variants as either pathogenic, likely pathogenic, likely benign, benign, or a variant of unknown significance (VUS) and to determine their respective distributions;
2. To reclassify VUSs using two different methods:
  (a) Association analysis comparing the prevalence of VUSs in this Danish population to the Swedish population using gnomAD;
  (b) Splice analysis using RNA sequencing.

**Figure 1:**
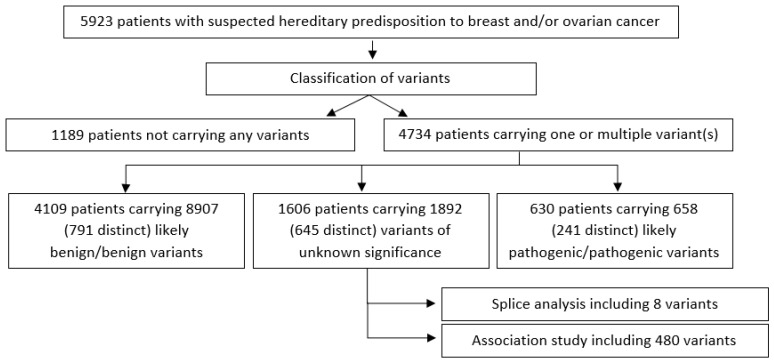
Flowchart of the procedures used in the current study.

The overall study flow is shown in Figure 1. Likely pathogenic and pathogenic are referred to as LP/P, and likely benign and benign are referred to as LB/B.

## 2. Materials and Methods

### 2.1. Study Population and Gene Panels

This register-based study consisted of 5923 patients with a suspected hereditary predisposition to breast and/or ovarian cancer. Patients had been referred according to the criteria of the Danish Cancer Cooperative Group (DBCG) (Table S1) [12,13,14,15] and their data were clinically analyzed between 1 January 2012 and 31 December 2022. Patients originated from Odense University Hospital (OUH), Aarhus University Hospital (AUH), and Zealand University Hospital, Roskilde (SUH). Next-generation sequencing (NGS) was performed using a gene panel at Odense University Hospital. The SWEA gene panel in this research study included 13, of which 11 were from the daily clinical setting (*ATM*, *BRCA1*, *BRCA2*, *BRIP1*, *CHEK2*, *PALB2*, *PTEN*, *RAD51C*, *RAD51D*, *STK11*, and *TP53*), one was a candidate gene (*BARD1*), and one was used for additional analysis on request (*CDH1*). The Lynch syndrome genes (*EPCAM*, *MLH1*, *MSH2*, *MSH6*, and *PMS2)* were excluded from the current study as data were not available for all patients. Samples were only included if germline SWEA-panel sequencing data were available and if they complied with quality criteria. A total of 2539 of the 5923 patients have previously been used for validation of BOADICEA [16].

### 2.2. Data Collection

A blood or tissue sample was used for genetic analysis, preferentially from a patient with confirmed cancer and alternatively from a healthy relative [5]. To determine the cancer diagnosis, we used data from the Danish Pathology Data Bank (DPDB) to label cancers as a primary cancer, direct invasion, or metastasis from the anatomic origin of interest. For a metastasis with unspecified origin, breast cancer was presumed for invasive ductal or lobular carcinoma while ovarian cancer was presumed for serous carcinoma. In situ cancer was excluded. Breast cancer estrogen receptor (ER) and human epidermal growth factor receptor 2 (HER2) status were collected.

### 2.3. Gene Panel Analysis

Sample preparation for NGS was performed using 3 µg DNA and Agilent’s SureSelectXT Reagent kit (Agilent Technologies Inc., Santa Clara, CA, USA) together with a custom-designed adaptor. Custom gene SureSelextXT panel (Agilent Technologies Inc., Santa Clara, CA, USA) (SWEA panel) designed at Lund University, Sweden, was used for target enrichment [17]. The panel included all introns and UTRs.

2 × 75 bp paired-end sequencing in an Illumina NextSeq (Illumina Inc., San Diego, CA, USA) or NovaSeq High Output (Illumina Inc., San Diego, CA, USA) flow cell was applied for the sequencing. The sequencing quality criteria required minimum coverage of 20× in 95% of the targeted regions. The mean coverage was >100×.

Data analysis had previously been performed using pipelines that changed over time. In the current study, we re-analyzed all data using Illumina DRAGEN Bio-IT platform (Illumina, San Diego, CA, USA) for alignment and variant calling. Human genome reference GRCh37 was used to align the sequencing data.

VCF files were imported to VarSeq (Golden Helix, Bozeman, MT, USA) for annotation and filtering. Variants were annotated using RefSeq transcripts and filtered by sequencing ontology and the gene-specific BA1 (benign stand-alone criterion in ACMG guidelines) population frequency.

VarSeq CNV caller (Golden Helix, Bozeman, MT, USA) was used to call copy number variants, using whole exons as the target region.

### 2.4. Variant Classification

The American College of Medical Genetics (ACMG) guidelines were used for variant classification [11]. Gene-specific guidelines were used when available: *ATM* v.1.3 [18], *BRCA1* v.1.1 [19], *BRCA2* v.1.1 [20], *CDH1* v.3.1 [21], *PALB2* v.1.1 [22], *PTEN* v.3.1 [23], and *TP53* v.1.4 [24]. For genes without a specific guideline at the time of classification (*BARD1*, *BRIP1*, *CHEK2*, *RAD51C*, *RAD51D*, and *STK11*), the general ACMG guidelines were applied, using the following decisions.

Decisions regarding benign criteria: As breast cancer is a common disorder in Denmark, BS1 (Allele frequency is greater than expected for disorder) was not applicable [11]. For BS4, at least three people in the same family should have the gene variant and not be affected. BP1 would not be applicable due to the limited knowledge about the prevalence of truncating variants. BP4 was dependent on the type of variant; missense variants should have a BayesDel no-AF ≤ 0.16 (same cut-off as the *BRCA* guidelines v.1.1) and a REVEL score ≤ 0.249 (same cut-off as the *TP53* guideline v.1.4). Silent, splice region, and deep intronic variants should have a SpliceAI score < 0.1 (same cut-off as the *BRCA* guidelines v.1.1). BP7 could be used for silent or deep intronic variants (+7/−21) that had no impact on splicing, defined as SpliceAI < 0.1.

Decisions regarding pathogenic criteria: Downgrading PM2 from moderate to supporting and allowing an allele count of a maximum of five in gnomAD v.4.0. For PP1, at least three people in the same family should have the gene variant and be affected. PP2 was not applicable due to the limited knowledge about the rate of benign missense variations. PP3 was dependent on the type of variant; missense variants should have a BayesDel no-AF ≥ 0.28 (same cut-off as the *BRCA* guidelines v.1.1) and a REVEL score > 0.733 (same cut-off as the *TP53* guideline v.1.4). Silent, splice region, and deep intronic variants should have a SpliceAI score ≥ 0.2 (same cut-off as the *BRCA* guidelines v.1.1) and were analyzed further using the RAW score.

As *CHEK2* pathogenic missense variants often have low penetrance, we conducted a search of the literature for odds ratio (OR). Likely pathogenic variants with an OR < 2.0 or with no existing data were downgraded to VUS. A conservative approach was used for variants with conflicting data, and they were consistently downgraded to VUSs. Furthermore, we defined splice region variants as +/− 20 nucleotides. If a variant was further away than +/− 20, it was defined as an intronic variant.

All variants with a SpliceAI delta > 0.2 were analyzed further by using the SpliceAI raw score to predict the possible splice effect. We compared changes in the natural and alternative splice sites to determine the specific splicing effect of each variant.

Gene variant information was collected in different databases and software, including VarSeq version 2.6.2 release, ClinVar (March and April 2024 release were used), MobiDetails (https://mobidetails.chu-montpellier.fr/, accessed on 1 March 2024), the Human Gene Mutation Database (HGMD Professional 2025.1), and Alamut^TM^ Visual Plus version 1.1. The information was cross-checked with ACMG criteria to see which criteria were fulfilled. The criteria were later combined according to the scoring rules [11], but the scoring rules could differ among the gene-specific guidelines [18,19,20,21,22,23,24]. The scoring rules consisted of five categories: C5 = pathogenic, C4 = likely pathogenic, C3 = variant of unknown significance, C2 = likely benign, and C1 = benign. Based on downgrading PM2 to supporting, another way to gain C4 was fulfilling PVS1 and one pathogenic-supporting criterion, which was adopted from the VCEP *BRCA* guideline [25]. Classification was performed during March and April 2024.

#### 2.4.1. Classification of Copy Number Variants (CNV)

We included only duplications with ratio > 1.3 and z-score > 2, and deletions with ratio < 0.75 and z-score < −2. Formalin-fixed paraffin-embedded (FFPE) tissue was excluded to reduce noise. Afterwards, a conservative visual assessment was made, which determined whether there was a visual clear change in ratio and z-score and lack of heterozygote variants in the area, and multiplex ligation-dependent probe amplification (MPLA) data, when available from earlier examination, were used to sort the remaining variants. CNV classification depends on type, and we were not able to locate breakpoints exactly or perform splice prediction on either deletions or duplications. Uncertainty about whether duplications were in tandem or not led to a C3 classification. Deletions with unknown breakpoints were classified as C4 [11].

#### 2.4.2. Classification of 5′-UTR Variants

5′-UTR variants were not well covered by the ACMG guidelines, resulting in mostly VUS classification (besides a few variants with high frequency in gnomAD). Therefore, we made four suggested, not validated, criteria to prioritize 5′-UTR variants. Benign criteria: BP4: CADD score < 20, BP6: reported benign in ClinVar, and BP7: beyond position +7/−21 and SpliceAI < 0.1. Pathogenic criteria: PP3: PreTIS score > 0.7 [26] and a minimum 0.05 increase from wildtype to mutated allele. Furthermore, criteria PM2_supporting and BS1 were applicable.

Suggested likely benign required at least two benign criteria and no PP3 criteria. Otherwise, the variants would remain VUSs and be included in the association analysis.

### 2.5. Association Analysis Method

The association analysis was only performed on VUS. The cohort was stratified into two subgroups: breast cancer patients and ovarian cancer patients. A patient was included in both subgroups if they were diagnosed with both cancer types. Those without breast and/or ovarian cancer were only included in the total study population analysis. We used the Swedish population information in the Genome Aggregation Database (gnomAD) v.2.1 as control group. Furthermore, the frequencies for non-Finnish Europeans (NFEs) v.4.0 and v.2.1 were used. Statistical analysis was not performed on NFEs due to risk of bias based on possible differences in ethnicity and geography. For variants with significant results (FDR ≤ 0.05), we applied PS4 as supporting as recommended by the general ACMG guidelines [11] due to the use of a general population as control group and patients referred to clinical laboratory. In line with the *ATM* guideline [18], we used OR > 2 as cut-off for genes without gene-specific guidelines since variants with OR < 2 were not seen as clinically relevant, although the general cut-off is OR > 5.0.

We followed gene-specific guidelines when these were available (See Table S2) [18,19,20,21,22,23,24]. *BRCA1* and *BRCA2* guidelines specified that cases should be matched to controls by ethnicity and country; therefore, the criterion was not applicable for our data [19,20].

### 2.6. Splice Analysis Method

Based on splice prediction and clinical assessment, 32 variants were selected for splice analysis (project approval ID: S-20220080). We invited one or two patients per variant to undergo splice analysis and ended with 47 patients invited. Thirty-eight had missing data due to non-response or death, so 9 patients were included (eight distinct variants), whose variants consisted of the following classifications prior to splice analysis: five C3 variants, one C4, one C2, and one C1. The benign and likely benign variants were included due to their high splice prediction. Blood samples were collected from each patient who consented, including two 4 mL EDTA and two 10 mL PAXgene.

Peripheral blood mononuclear cells (PBMCs) were extracted from patient EDTA blood samples using a density gradient (Lymphoprep^TM^, STEMCELL Technologies, Vancouver, BC, Canada). Purified PBMCs were cultured in 10 mL Medium 199 containing phytohemagglutinin for 4–6 days at 37 °C. To inhibit nonsense-mediated decay (NMD), 200 µL puromycin was used and incubated for 4–6 h before cell harvest.

Total RNA was extracted from each cell culture using the RNeasy Plus Kit (QIAGEN, Venlo, The Netherlands, Cat. No. 74104). A targeted approach was applied for splicing analysis. cDNA synthesis was carried out using the SuperScript IV First-Strand Synthesis System (ThermoFisher Scientific, Waltham, MA, USA, Cat. No. 18091050), followed by PCR enrichment using AccuPrime^TM^ Taq DNA Polymerase, High Fidelity (Invitrogen, ThermoFisher Scientific, Waltham, MA, USA, Cat. No. 12346-086 and 12346-094). Primers for enrichment were designed using NCBI Primer BLAST (https://www.ncbi.nlm.nih.gov/tools/primer-blast/, accessed on 24 February 2024) (See Table S3) [27].

Data processing and alignment were performed using Illumina DRAGEN Bio-IT Platform (Illumina, CA, USA), and the Human transcriptome reference GRCh38 RefSeq was used for mapping. Bam files were imported to Integrative Genomics Viewer for analysis of splicing patterns [28]. four–five control patients (8–10 samples) were included in the experimental setup.

The study quality could be affected by many factors such as RNA material, use of nonsense-mediated decay (NMD) inhibition, and tissue-matched controls [18,29]. For all samples, we used EDTA blood from the proband, the same NMD inhibitor throughout the experiments, reverse transcript-PCR, deep-targeted sequence data, and EDTA blood from 4–5 controls. We followed the ClinGen SVI Splicing Subgroup recommendation of using PVS1 (RNA) and BP7_strong (RNA) for splicing data when applicable [29]. SVI recommends downgrading PVS1 by one step for a near-complete effect [29], which is in line with the gene-specific *ATM* guideline [18]. We decided to downgrade one time for a near-complete benign effect as well. As cut-off for a near-complete pathogenic splice variant, we used ≤10% functional gene expression as suggested by SVI Splicing Subgroup [29]. For a near-complete benign variant, we used the cut-off ≥90% functional gene expression. Percentage of affected junction reads was calculated as recommended by Davy et al. [30].

### 2.7. Statistical Analysis

Allele counts obtained from VarSeq were used to calculate the distribution of patients with (1) LP/P variants, (2) VUSs, or (3) LB/B variants, which was also calculated in the two subgroups of breast cancer and ovarian cancer. For (1) and (2), the distribution based on gene and sequence ontology was also calculated.

The allele counts were used as input for the association study. For each tested variant, these allele counts were grouped as either carrier or non-carrier. This procedure was also applied to the control group. As the allele data were counted using numbers, we used a 2 × 2 contingency table of these allele counts for a Fisher’s exact test to calculate the odds ratio and associated *p*-values for the association analysis. The associated *p*-values were corrected for multiple testing using the false discovery rate (FDR), and FDR values ≤ 0.05 were considered statistically significant.

The open-source R-environment (version 4.4.1) and Microsoft Excel were used for all of the above calculations.

## 3. Results

A total of 6824 samples were analyzed using the SWEA panel between 1 January 2012 and 31 December 2022. Of the 901 samples that were discarded, 266 had missing data, 179 were of low quality, and 456 were duplicates or tumor tissue. The final study population was 5923 patients. Table 1 provides further details of these patients.

### 3.1. Distribution of Variants After Classification

In the current study, 38 (22.8%) of the 167 variants that had previously been classified as VUSs in the clinical setting were either upgraded or downgraded using the general ACMG guidelines and gene-specific guidelines.

**Figure 2:**
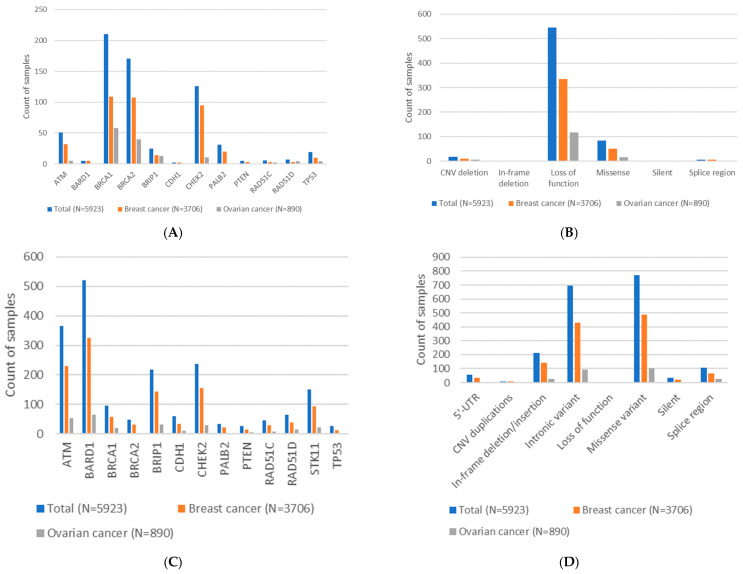
Distribution of identified likely pathogenic variants, pathogenic variants, and variants of unknown significance (VUSs). (**A**) Distribution of 658 likely pathogenic and pathogenic variants (241 distinct) based on genes in the study population of 5923 patients, of whom 3706 had breast cancer and 890 had ovarian cancer. (**B**) Distribution of the 658 likely pathogenic and pathogenic variants based on sequence ontology. Splice region variants are located within +/−20 of the splice site. (**C**) Distribution of 1892 VUSs (645 distinct) based on genes in the study population. (**D**) Distribution of the 1892 VUSs (645 distinct) based on sequence ontology. Splice region variants were defined as located within +/−20 of the splice site. Intronic variants were defined as further away than +/− 20 nucleotides from the splice site.

In 630 patients (10.6%), we identified 658 LP/P variants (241 distinct variants). One patient had three LP/P variants, and eight patients had two LP/P variants. A total of 390 breast cancer patients (10.5%) and 131 ovarian cancer patients (14.7%) carried an LP/P variant, mainly in *BRCA1* (31.9%) and *BRCA2* (26.0%), as shown in Figure 2A and Table 2. The most frequent type of variant was loss of function (83.0%), which was defined as those that exhibited nonsense, frameshift, a canonical +/−1 or 2 splice site, an initiation codon, and single or multi-exon deletion (Figure 2B and Table 2). The classification and frequency of all LP/P variants are shown in Table S4, and the details for the CNVs are provided in Table S8.

In 1606 patients (27.1%), we classified 1892 VUSs (645 distinct). Two patients had four VUSs, 32 patients had three VUSs, and 209 patients had two VUSs. A total of 1010 breast cancer patients (27.3%) and 230 ovarian cancer patients (25.8%) carried a VUS, and these were mainly in *BARD1* (27.6%) and *ATM* (19.3%), as shown in Figure 2C and Table 2. The two most frequent types of variants were missense (40.9%) and deep intronic (36.7%), as shown in Figure 2D and Table 2. The classifications and frequencies of VUSs are shown in Tables S5, S7 and S8.

In 4109 patients (69.4%), we classified 8907 LB/B variants (791 distinct variants). A total of 2196 patients had more than one variant.

Based on our classification criteria, 66 5′-UTR variants were downgraded from VUSs to likely benign, so only 35 variants were classified as VUSs. Of these, one was likely benign according to the ACMG guidelines, but it was reclassified as a VUS due to a exhibiting positive translational start site prediction. Fifteen variants were classified as likely benign or benign using either the ACMG guidelines or the suggested criteria. The entire 5′-UTR analysis can be seen in Table S7.

In this research study, a total of 364 of the 791 distinct LB/B variants, 114 of the 645 distinct VUSs, and 152 of the 241 distinct LP/P variants had already been classified by the national Danish cancer variant classification group (Cancer Variant Classification Denmark, ccDK) [31] or the Evidence-based Network for the Interpretation of Germline Mutant Alleles (ENIGMA), and, therefore, were not re-evaluated in this study.

### 3.2. Association Analysis

Of the 645 distinct VUSs observed in this study, 4 CNV, 35 5′-UTR, and 126 intronic variants were excluded from the association analysis due to a lack of control group data.

Of the remaining 480 distinct VUSs, 9 variants in the breast cancer group and 3 in the ovarian cancer group were significantly associated with breast or ovarian cancer risk after correction for multiple testing (FDR ≤ 0.05) (Table 3). However, applying PS4_supporting to any of the nine variants did not result in reclassification, and they remained VUSs. The complete analysis is shown in Table S9.

### 3.3. Splice Analysis

**Figure 3:**
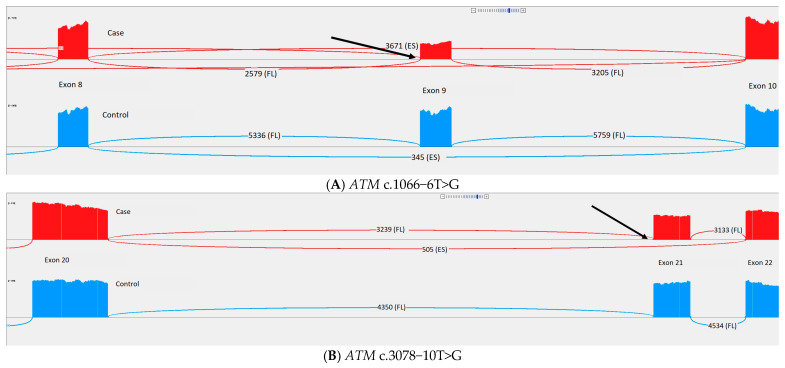

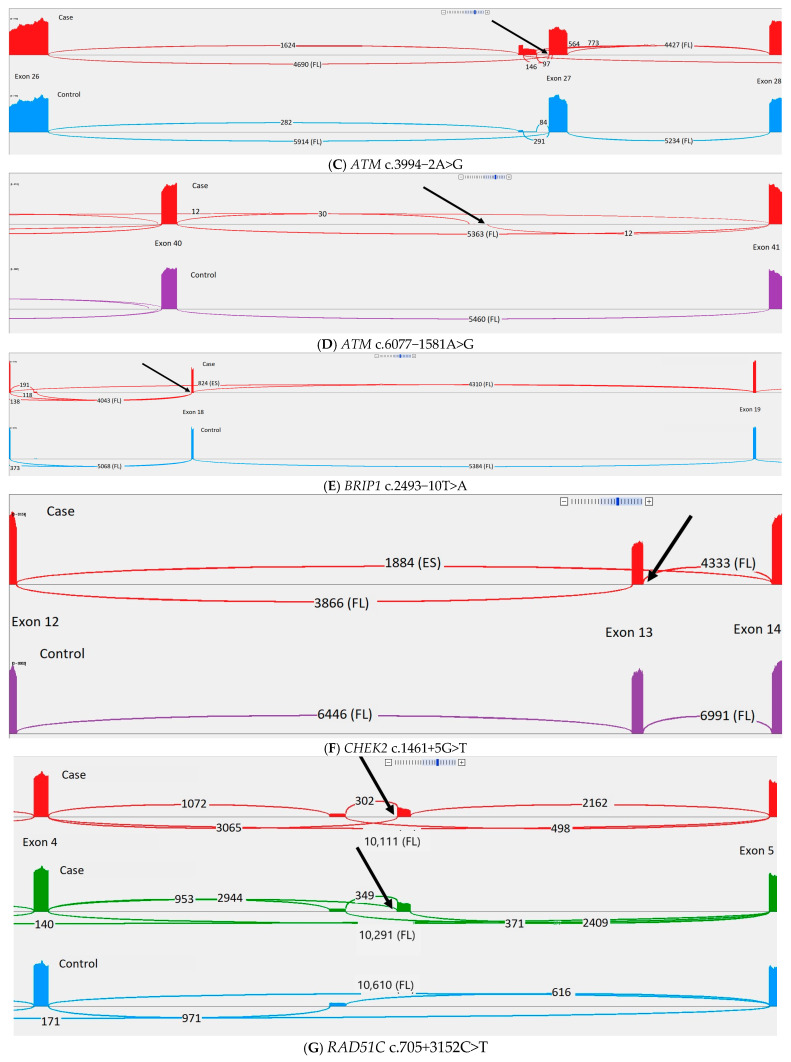

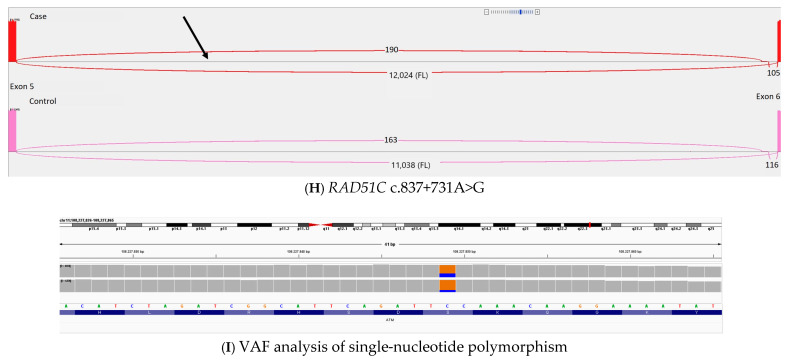
Sashimi plots of the eight distinct variants (nine samples) included in the splice analysis. Each sashimi plot shows the proband(s) and one control, both of which were treated with nonsense-mediated decay (NMD) inhibitor. The arrow indicates the position of the gene variant in the proband(s). Sashimi plots showing variants which were both treated and untreated with NMD inhibitor and all controls are available in Figure S1. (**A**) *ATM* c.1066−6T>G. (**B**) *ATM* c.3078−10T>G. (**C**) *ATM* c.3994−2A>G. (**D**) *ATM* c.6007−1581A>G. (**E**) *BRIP1* c.2493−10T>A. (**F**) *CHEK2* c.1461+5G>T. (**G**) *RAD51C* c.705+3152C>T. (**H**) *RAD51C* c.837+731A>G. (**I**) VAF analysis of single-nucleotide polymorphism located in the coding area in exon 3. FL: full-length. ES: exon skipping.

Eight distinct variants (nine samples) were included in the splice analysis based on splice prediction and clinical assessment. Sashimi plots for the splice analysis are shown in Figure 3 and Figure S1. Splice prediction for the variants is shown in Table 4.

*ATM* c.1066-6T>G showed skipping of exon 9 leading to frameshift and stop-codon in exon 10. As our data showed the exon skipping to be complete, we assigned PVS1 (RNA) to this gene variant, which challenged its prior classification of benign.

In *ATM* c.3078-10T>G, there was skipping of exon 21 leading to frameshift and stop-codon in exon 22. Exon skipping was present in 15.9% of the junctions and, thus, no criterion was applicable.

In *ATM* c.3994-2A>G, there was skipping of exon 27 leading to frameshift and stop-codon in exon 28. Furthermore, there was intron retention affecting a cryptic exon that is normally observed at very low abundance. The combinations of these two events leads to the inclusion of 37 amino acids that contain a stop-codon, which was present in 34% of the mutant transcript. Due to the location of the splice variant, we were surprised at the low amount of abnormal splicing in the variant allele. Therefore, we examined the variant allele frequency (VAF) for a single-nucleotide polymorphism located in the coding area in exon 3 (see Figure 3I). This showed that the VAF was skewed, and that the allele with abnormal splicing was only present in 30% of the transcripts. The skewed VAF and low percentage of abnormal splicing indicate incomplete NMD inhibition. The effect of the c.3994-2A>G splice variant is probably greater than shown in the splice analysis due to PCR bias. As the exon 3 variant was measured in another PCR fragment, it was unlikely that our result was biased by the PCR fragment size. Nevertheless, no classification code was applicable.

*ATM* c.6007-1581A>G showed the inclusion of an in-frame pseudoexon. As this pseudoexon was only present in a few junctions (0.39%), we assigned BP7_strong, reclassifying the variant from likely benign to benign.

*BRIP1* c.2493-10T>A showed in-frame skipping of exon 18 in 19.7% of the junctions, but a lack of exon 18 was also present in some controls. This suggested that this gene variant is a naturally occurring isoform, and, therefore, no criterion was applicable.

In *CHEK2* c.1461+5G>T, there was skipping of exon 13 leading to frameshift and stop-codon in exon 14. Exon skipping was present in 46% of the junctions, and, therefore, no criterion was applicable.

*RAD51C* c.705+3152C>T was present in two probands and showed the inclusion of an in-frame pseudoexon. A few junctions had inclusion of the pseudoexon together with the presence of a rare natural transcript exon, leading to out-of-frame inclusion. The pseudoexon was present in 25.8% of the junctions and, therefore, no criterion was applicable. The rare natural transcript exon was present in 7.8% of the junctions.

In *RAD51C* c.837+731A>G, there was no difference between the variant and the controls, so we assigned BP7_strong (RNA), although, with the general ACMG guidelines, this was not enough for reclassification. When gene-specific guidelines are published for *RAD51C*, we expect that the gene variant will be reclassified as likely benign.

## 4. Discussion

In a cohort of 5923 patients with suspected predisposition to hereditary breast and/or ovarian cancer, we identified 658 likely pathogenic/pathogenic variants in 630 patients (10.6%), and 1892 variants in 1606 patients were classified as VUSs (27.1%). The remaining patients carried either likely benign/benign variants or no variants. We attempted to reclassify 645 distinct VUSs identified in our study using association analysis and splice analysis. In the association analysis, PS4_supporting was applied to 10 variants, but none were reclassified. In the splice analysis, eight distinct variants (different to those found significant in the association analysis) were included. No ACMG criterion was applicable for five of the eight variants, given that the ratio between the functional and non-functional transcripts did not allow for reclassification as benign or pathogenic.

One of the five variants, *CHEK2* c.1461+5G>T, showed exon skipping in 46% of the mutant transcripts. However, Sanoguera-Miralles described exon 13 skipping in 85.8% and exon 13 skipping together with partial exon 12 skipping in 12.8% of variant transcripts [32]. This might be due to differences between the use of a constructed model system in a minigene assay and the use of RNA sequencing on a patient sample; PCR bias is also likely to impact the results that are obtained [33]. Another variant, *ATM* c.3994-2A>G, showed abnormal splicing in 34% of the mutant transcripts. In a mini-gene assay by Bueno-Martínez and colleagues, the same variant resulted in 100% exon 27 skipping, which supports a potential pathogenic effect [34]. A possible incomplete NMD inhibition in our study, together with different model systems, could explain the difference between our study and that of Bueno-Martínez et al., but the splice effect in this variant appears to be more complex than merely exon skipping. Interestingly, we also observed the retention of an exon reported in another isoform. This supports the use of patient samples for splicing analysis.

One of the eight distinct variants, *ATM* c.1066-6T>G, was classified as benign by an expert panel [35], which conflicts with our splice analysis that showed complete exon 9 skipping. Schröder et al. described *ATM* c.1066-6T>G as having reduced *ATM* protein levels and kinase activity [36]. Other studies support the possible pathogenic effect of *ATM* c.1066-6T>G on either ataxia telangiectasia or breast cancer [37,38]. However, in multiple cases, *ATM* c.1066-6T>G was identified together with another pathogenic *ATM* variant that did not have an ataxia telangiectasia phenotype [39,40], and some articles state that *ATM* c.1066-6T>G is benign [41,42]. Furthermore, this variant is reported as homozygous in gnomAD 2.1. Further studies are needed to evaluate this variant.

We classified three distinct loss of function variants as variants of unknown significance. *RAD51C* c.1025_1026delAA was not predicted to undergo NMD, while *RAD51D* c.1A>T was classified by the national Danish cancer variant classification group (Cancer variant classification Denmark, ccDK). For *BRIP1* c.2905+2T>A, unpublished results from an ongoing project within the ENIGMA consortium show that variants downstream of c.2576 should likely be classified as VUSs. These three variants are challenging due to their uncertain pathogenic potential and require further investigation.

Recent Danish papers on the distribution of gene variants in a 2006–08 cohort of patients with a suspected hereditary predisposition to breast and/or ovarian cancer found that 28% of the highly selected cohort had a pathogenic *BRCA1* or *BRCA2* variant [8,9,31]. We found LP/P variants in 6.4% of our cohort. Three explanations for this difference are changes in referral guidelines, the dramatic increase in the number of patients being genetically tested, and increased sensitivity of assessment techniques. In addition, the breast/ovarian gene panel used in the clinical setting has expanded. The Department of Clinical Genetics at Odense University Hospital added *BRIP1*, *EPCAM*, *MLH1*, *MSH2*, *MSH6*, *PALB2*, *PMS2*, *RAD51C*, and *RAD51D* in October 2017 and *PTEN*, *STK11*, and *TP53* in June 2019. Two further genes, *ATM* and *CHEK2*, were added in November 2022, and the breast/ovarian gene panel used in the clinical setting currently consists of these 16 genes. All 13 genes in the current study were implemented for research purposes in 2012. Direct comparisons between the two previous Danish studies and the current study are thus not possible.

Other studies investigating the prevalence of LP/P variants in breast and/or ovarian cancer patients include a 2021 study from the Breast Cancer Association Consortium [43], which identified 2832 protein-truncating variants (4.8%) in 113,000 patients when analyzing the 13 genes that are also included in our study. In 2018, Hauke et al. [44] observed 329 pathogenic variants (5.9%) and 811 VUSs (14.5%) in 5589 *BRCA*-negative patients in seven genes that were included in our study (*ATM*, *CDH1*, *CHEK2*, *PALB2*, *RAD51C*, *RAD51D*, and *TP53*). Öfverholm et al., in 2023 [17], identified 765 patients who had at least one pathogenic variant (16.6%) from a sample of 4622 women with a suspected hereditary predisposition to breast and/or ovarian cancer. The difference between these studies and ours could be due to the underrepresentation of *BRCA*-positive patients, our study not selecting based on family history, differences in inclusion criteria, more restrictive referral criteria, the number of healthy relatives in the cohort, and the use of the ACMG guidelines.

### Strength and Limitations

A strength of the current study was the inclusion of all patients who were referred (and originally analyzed using SWEA panel), which ensured large samples. The genetic analyses were performed at the same university hospital and using the same panel, ensuring quality and consistency, and the data were re-analyzed using a state-of-the-art pipeline. The use of pathological data ensured appropriate clinical diagnosis, and the gene variants were thoroughly classified using both the ACMG guidelines and gene-specific guidelines.

However, only 62.6% of our patients were diagnosed with breast cancer and 15% with ovarian cancer; thus, there is a risk that our results are underestimations. Furthermore, 92.3% of the patients that were included were women. Although breast cancer is rare in men [45], it is uncertain to what extent the results of this study are applicable to men.

This study was designed based on the ACMG guidelines for classification, and these were applied for 6 of the 13 genes that were included. The ACMG guidelines have not been updated since 2015, however, and the scientific field has expanded since then. It has been proposed that one strong benign criterion should be enough to classify a gene variant as being likely benign [46], and that the criteria PP5 and BP6 should be removed [47]. This would change the gene variant classifications assigned in the current study.

The primary limitations in our association analysis were the population size (despite the sample reaching nearly 6000 patients) and the control group. The Swedish population of gnomAD 2.1 was not a perfectly matched control group, limiting the weight of PS4, and this criterion was therefore inapplicable for *BRCA1* and *BRCA2*. A future study could include a control group that is also matched by ethnicity and country to provide more accurate results. The lack of longitudinal data means that we cannot know whether some healthy patients will be diagnosed with cancer later in life, or if some patients carry germline mutations in genes that are yet to be identified or in genes that are not yet included in gene panels. The association analysis included a Fisher’s exact test and a significance cut-off of ≤0.05 for FDR, but a Bonferroni test would have given fewer significant results.

A limitation in the splice analysis was the risk of PCR bias, which could have overestimated the abundance of shorter isoforms. Heterozygote variants were only available in the tested area for *ATM* c.3994-2A>G, which reduced our ability to ascertain PCR bias in this study. Additionally, the number of patients and the lack of overlapping variants limit the power of the current study.

## 5. Conclusions

In our sample of patients with suspected predisposition to hereditary breast and/or ovarian cancer, 10.6% of patients carried a likely pathogenic/pathogenic variant, and 27.1% carried a variant of unknown significance. These frequencies could be useful in the daily clinical setting when considering how often a monogenetic cause should be expected for such hereditary predisposition. The application of the ACMG guidelines led to the re-classification of 22.8% of the previous variants of unknown significance, thus possibly reducing the clinical challenges associated with these variants. The association analysis assigned criteria to 10 variants, while the splice analysis assigned criteria to 3 variants. Further research into variant classification and additional Variant Curation Expert Panel (VCEP) guidelines is needed to reduce the number of variants of unknown significance and to improve genetic counselling.

## Figures and Tables

**Table 1:** Clinical characteristics of our study population of 5923 patients with suspected hereditary predisposition to breast and/or ovarian cancer.

	Number (%)
Sex, female	5467 (92.3%)
Cancer ^a^	4598 (77.6%)
Breast cancer ^b^	3706 (62.6%)
Bilateral breast cancer	564 (9.5%)
Ovarian cancer	890 (15.0%)
Prostate cancer	106 (1.8%)
Pancreatic cancer	34 (0.6%)
Average age breast cancer patients ^c^	51.4 years
Average age ovarian cancer patients ^d^	61.8 years
ER, positive ^e^	2588 (69.9%)
ER, negative ^e^	782 (21.1%)
ER, unspecified ^e^	336 (9.1%)
HER2, positive ^e^	517 (14.0%)
HER2, negative ^e^	2595 (70.0%)
HER2, unspecified ^e^	594 (16.0%)

^a^ breast, ovarian, pancreatic, or prostate cancer. ^b^ both uni- and bilateral breast cancer. ^c^ age at first diagnosis of breast cancer. ^d^ age at first diagnosis of ovarian cancer. ^e^ calculated from the total number of breast cancer patients.

**Table 2:** Distribution of variants of unknown significance (VUSs) and likely pathogenic or pathogenic variants (LP/P) in 13 genes among 5923 patients.

Total Number of	VUS Number	LP/P Number
Identified variants	1892	658
Carriers	1606	630
Distinct variants	645	241
**Variant types**	**VUS** Count of total variants (count of distinct variants)	**LP/P** Count of total variants (count of distinct variants)
Loss of function	3 (3)	546 (187)
In-frame deletion/insertion	212 (17)	2 (2)
Missense	773 (383)	83 (33)
Silent	36 (19)	2 (2)
Splice region	108 (58)	7 (6)
Intronic	695 (126)	
5-UTR	56 (35)	
Copy number	9 (4)	18 (11 distinct)
**Gene**	**VUS** Count of total variants (count of distinct variants, percentages compared to the total population)	**LP/P** Count of total variants (count of distinct variants, percentages compared to the total population)
*ATM*	365 (197, 6.16%)	51 (27, 0.86%)
*BARD1*	522 (53, 8.81%)	5 (4, 0.08%)
*BRCA1*	96 (38, 1.62%)	210 (66, 3.55%)
*BRCA2*	48 (32, 0.81%)	171 (68, 2.89%)
*BRIP1*	217 (60, 3.66%)	25 (6, 0.42%)
*CDH1*	60 (40, 1.01%)	2 (2, 0.03%)
*CHEK2*	236 (56, 3.98%)	126 (10, 2.13%)
*CHEK2 c.1100delC*		99 (1.67%)
*PALB2*	34 (24, 0.57%)	31 (20, 0.52%)
*PTEN*	27 (8, 0.46%)	5 (5, 0.08%)
*RAD51C*	45 (23, 0.76%)	6 (3, 0.10%)
*RAD51D*	64 (26, 1.08%)	7 (3, 0.12%)
*STK11*	152 (34, 2.57%)	
*TP53*	26 (15, 0.44%)	19 (16, 0.32%)

Carriers: number of patients carrying a gene variant. VUS: variant of unknown significance. LP/P: likely pathogenic/pathogenic.

**Table 3:** Results from association analysis with a significant false discovery rate (FDR) compared with the Swedish population data in gnomAD 2.1. Association analysis was conducted on variants of unknown significance (VUSs) in 3706 patients with breast cancer (BC) and 890 patients with ovarian cancer (OC).

			Number of Samples and Frequencies						Association Study: gnomAD 2.1 Swedish vs.	
			Study Population		Genome Aggregation Database (gnomAD) Groups				BC	OC
Gene	Sequence Ontology	HGVSc.	BC (*n* = 3706)	OC (*n* = 890)	gnomAD2.1 (Sweden) (*n* = 13,067)	gnomAD2.1 (Sweden) Frequency	gnomAD2.1 (NFE) Frequency (*n* = 56,885)	gnomAD4.0 (NFE) Frequency (*n* = 590,031)	OR (95%CI); FDR	OR (95% CI); FDR
*ATM*	Missense	NM_000051.4:c.3519G>C	2	3	0	0	0	3.39 × ^10−6^	∞(0.66–∞); 0.36	∞(6.08–∞); **0.041**
*ATM*	Synonymous	NM_000051.4:c.7521C>T	1	4	0	0	8.79 × 10^−5^	0.00015	∞(0.09–∞); 0.36	∞(9.71–∞); **0.0039**
*ATM*	Missense	NM_000051.4:c.8428A>C	7	0	0	0	0.00012	7.80 × 10^−5^	∞(5.09–∞); **0.0041**	NA
*BARD1*	Splice_region	NM_000465.4:c.1569-13C>G	5	2	0	0	8.79 × 10^−5^	2.034 × 10^−5^	∞(3.23–∞); **0.028**	∞(2.76–∞); 0.21
*BARD1*	Missense	NM_000465.4:c.1915T>C	6	0	0	0	8.79 × 10^−5^	0.00011	∞(4.16–∞); **0.0079**	NA
*CHEK2*	Missense	NM_007194.4:c.433C>T	6	1	0	0	0.00011	0.00011	∞(4.16–∞); **0.0079**	∞(0.38–∞); 0.48
*CHEK2*	Missense	NM_007194.4:c.715G>A	6	2	0	0	0.00011	0.00023	∞(4.16–∞); **0.0079**	∞(2.76–∞); 0.21
*CHEK2*	Missense	NM_007194.4:c.1183G>C	8	2	3	0.00023	8.79 × 10^−5^	5.59 × 10^−5^	9.42(2.26–55.12); **0.028**	9.8(0.82–85.67); 0.48
*CHEK2*	Missense	NM_007194.4:c.1427C>T	22	2	14	0.0011	0.0010	0.00098	5.57(2.72–11.78); **0.00024**	2.1(0.23–9.16); 1
*BRCA1*	Splice_region	NM_007294.4:c.4096+3A>G	8	5	0	0	0	5.084 × 10^−6^	∞(6.03–∞); **0.0013**	∞(13.50–∞); **0.00005**
*BRIP1*	In-frame_ deletion	NM_032043.3:c.1687_1689delGAT	9	2	2	0.00015	3.52 × 10^−5^	0	15.9(3.29–151.18); **0.0052**	14.7(1.06–202.8); 0.48

Bolding indicates significant FDR values (FDR ≤ 0.05). 95% CI: 95% confidence interval. BC: breast cancer. gnomAD: the Genome Aggregation Database. FDR: false discovery rate. HGVSc.: Human Genome Variation Society coding sequence name. NFE: non-Finnish European. OC: ovarian cancer. OR: odds ratio. CI: confidence interval. NA: not avaible. We applied PS4_supporting to eight of nine variants with FDR ≤ 0.05 in the breast cancer group and two of three variants in the ovarian cancer group, but they all remained VUSs. *BRCA1* c.4096+3A>G was significant in both groups, but PS4 was not applicable due to the gene-specific guideline requiring a country-matched control group.

**Table 4:** Classification and criteria prior to splice analysis, number of samples in gnomAD, and classification in ClinVar and SpliceAI predictions for the eight variants (nine samples) included in the splice analysis. The parentheses show the position in relation to the variant. Tables S4–S6 provide more information on the classification prior to splice analysis.

Variant	Prior Classification	Criteria Used for Prior Classification	Criteria After RNA Sequencing	Samples gnomAD 4.0 (NFE)	Samples gnomAD 2.1 (NFE)	Samples gnomAD 2.1 (Swedish)	ClinVar	SpliceAI Delta Acceptor Loss (Position)	SpliceAI Delta Acceptor Gain (Position)	SpliceAI Delta Donor Loss (Position)	SpliceAI Delta Donor Gain (Position)
*ATM* c.1066-6T>G	C1	BS1BP2_strong	PVS1 (RNA)	3044	253	43	C1: 12 C2: 11 C3: 7	0.62 (6)	0.00 (1)	0.00 (5)	0.00 (−1)
*ATM* c.3078-10T>G	C3	PM2_supporting PP3		0	0	0	C3: 2	0.70 (10)	0.00 (1)	0.00 (43)	0.00 (−1)
*ATM* c.6007-1581A>G	C2	BS1	BP7_ strong	235	0	0	NA	0.00 (284)	0.23 (−105)	0.05 (143)	0.64 (0)
*ATM* c.3994-2A>G	C4	PVS1PM2_supporting		6	4	4	C4: 6 C5: 3	0.99 (2)	0.42 (22)	0.00 (20)	0.00 (−40)
*BRIP1* c.2493-10T>A	C3	PM2_supportingPP3		1	1	1	C3: 2 C2: 2	0.00 (−2)	0.30 (−10)	0.00 (−11)	0.00 (−1)
*CHEK2* c.1461+5G>T	C3	PM2_supportingPP3		0	0	0	C4: 1 C3: 4	0.00 (33)	0.00 (−21)	0.90 (5)	0.00 (50)
*RAD51C* c.705+3152C>T	C3	PM2_supportingPP3		2	0	0	NA	0.00 (127)	0.46 (8)	0.00 (−464)	0.34 (127)
*RAD51C* c.837+731A>G	C3	PM2_supporting PP3	BP7_ strong	0	0	0	NA	0.00 (58)	0.23 (−125)	0.06 (−69)	0.66 (−5)

Classification categories: C1: benign, C2: likely benign, C3: variant of unknown significance, C4: likely pathogenic, C5: pathogenic. NFE: non-Finnish Europeans. gnomAD: Genome Aggregation Database. NA: not applicable.

## Data Availability

The datasets presented in this article are not readily available as we do not have permission for sharing. This is also unlikely to be permitted in the future as the datasets contain sensitive personal data with important health information.

## Supplementary Materials

The following supporting information can be downloaded at: https://www.mdpi.com/article/10.3390/cancers17111819/s1, Table S1: Referral criteria from the Danish Breast Cancer Cooperative Group (DBCG); Table S2: PS4 criteria; Table S3: Primer sequences for splice analysis; Table S4: Classification and frequencies of likely pathogenic and pathogenic variants; Table S5: Classification and frequencies of variants of unknown significance (VUS); Table S6: Classification and frequencies of likely benign and benign variants; Table S7: Classification and frequencies of 5′-UTR variants; Table S8: Classification and frequencies of copy number variants (CNV); Table S9: Association analysis for variants of unknown significance (VUS); Table S10: Blank version of declaration of consent; Figure S1: Sashimi plots.
