# Supplementary material for: Classification of Gene Variants in a Danish Population with Suspected Predisposition to Hereditary Breast and/or Ovarian Cancer
Source: Cancers (Basel). 2025 May 29;17(11):1819. doi: 10.3390/cancers17111819 (PMC12153586; doi:10.3390/cancers17111819)

Supplementary materials

Supplementary material overview

Table S1: Referral criteria from Danish Breast Cancer Cooperative Group (DBCG)

Table S2: PS4 criteria

Table S3: Primer sequences for splice analysis

Table S4: Classification and frequencies of likely pathogenic and pathogenic variants

Table S5: Classification and frequencies of variants with unknown significance (VUS)

Table S6: Classification and frequencies of likely benign and benign variants

Table S7: Classification and frequencies of 5'-UTR variants

Table S8: Classification and frequencies of copy number variants (CNV)

Table S9: Association analysis of variants with unknown significance (VUS)

Table S10: Blank version of declaration of consent

Figure S1: Sashimi plots

Table S1: Referral criteria for the Department of Clinical Genetics recommended by The Danish Breast Cancer Cooperative Group in the study period.

The following list is a collection of guidelines from 2010 [10], 2014 [11], 2016 [12] and 2020 [13] to cover the study period from 2012-2022. The guidelines from 2010 and 2014 had the same referral criteria, and the new/changed criteria from 2016 and 2020 is marked.

- Suspicion of hereditary disposition to breast cancer should be raised in families with at least one of the following criteria is fulfilled:
    - A patient diagnosed with breast cancer before turning 40 years.
    - A female diagnosed with breast cancer in age 40-50 years, who have not already been investigated for gene variants in *BRCA1* and *BRCA2* with a normal result.[2020]
    - A patient diagnosed with both breast cancer and ovarian cancer.
    - A female diagnosed with ovarian cancer.[2016]
    - A female diagnosed with bilateral breast cancer. [2016]
    - A female diagnosed with breast cancer before age of 60 years and the tumor is ER and HER2 negative or is "basal-like" gene expression.[2016]
    - A female diagnosed with breast cancer before age of 60 years and the invasive tumor component is ER negative and HER2 normal or is basal-like molecular subtype in the case of low ER status (1-9%).[2020]
    - Two first-degree relatives diagnosed with breast cancer before age of 50 years and/or ovarian cancer.
    - ≥ Two first degree relatives diagnosed with breast cancer. [2020]
    - Three first-degree relatives diagnosed with breast cancer, of which one was diagnosed before age of 50 years.
    - Three first-degree relatives diagnosed with breast cancer. [2016]
    - A man diagnosed with breast cancer.
- Reference to genetic risk assessment and counselling should be made in families, which:
    - Suspicion of hereditary disposition, confer the above.
    - Families with an identified mutation, which increase the risk of breast and/or ovarian cancer.
    - Families with an identified disease-disposing gene variant, which predisposes of breast cancer.[2020]

- A gene variant of unknown significance (VUS, C3) have been detected earlier, and the patient now want a reassessment if it predispose to breast cancer.[2020]

- It has former been assessed that an increased risk of breast cancer is inherited and there is indication for a new consultation e.g. new cancer cases in the family, the patient is approaching the year for the recommended surveillance program or the patient needs a follow-up.

Table S2: PS4 criteria

Gene	Use general guideline	Use gene specific guideline	Specification on the gene specific guidelines
ATM		x	Strong: Case-control studies; p-value ≤.05 AND (Odds ratio, hazard ratio, or relative risk ≥2 OR lower 95% CI ≥1.5).<br>Moderate: Do not use for proband counting.
BARD1	x		
BRCA1		x	Instructions: Case dataset should be ethnicity and country-matched to control dataset. If case-control LR estimates are available for a given dataset, these should be used in preference to case-control OR, under code PP4 (or BP5, if appropriate). Do not used Proband Counting as originally described.<br>Strong: The prevalence of the variant in affected individuals is significantly increased compared to the prevalence in controls. Case-control studies; p-value ≤0.05 and OR ≥4 (lower confidence interval excludes 2.0).
BRCA2		x	Same as *BRCA1*
BRIP1	x		
CDH1		x	Instructions: Use the 2020 updated clinical practice guidelines as the HDGC phenotype criteria. PS4 cannot be applied to variants that meet BS1 or BA1, or to variant in which less than 30% reported individuals meet HDGC criteria:<br>Very Strong: ≥Sixteen families meet HDGC criteria.<br>Strong: Four - Fifteen families meet HDGC criteria.<br>Moderate: Two or three families meet HDGC criteria.<br>Supporting: One family meets HDGC criteria.
CHEK2	x		
PALB2		x	Strong: Case-control studies; p-value ≤.05 AND (Odds ratio, hazard ratio, or relative risk ≥3 OR lower 95% CI ≥1.5).<br>Moderate: Do not use. Proband counting for gene causing a common disorder need to be calibrated in a population-specific way before use.
PTEN		x	The criterion is unlikely to be used in this manner for a condition as rare as PHTS. However, if sufficiently powered, a case-control study finding an odds ratio >2 for a PHTS component phenotype with p<0.05 and 95% confidence interval lower limit >1.5, this criteria may be applied. However, this criterion may not be applied in combination with PP4.<br>This criterion may not be applied if BS1 applies. Phenotype specific scores are added across independent probands and calculated as follows:<br>Adults:<br>1 point per proband with CC score >30<br>0.5 point per proband with CC score of 25-29

			Children:<br>1 point per proband with pediatric phenotype > 5<br>0.5 points per proband with pediatric phenotype score of 4, but autism/developmental delay/intellectual disability may not contribute to the score.<br>Very Strong: Probands with specificity score ≥16<br>Strong: Probands with specificity score 4-15.5 OR The prevalence of the variant in affected individuals is significantly increased compared with the prevalence in controls.<br>Moderate: Probands with specificity score of 2-3.5<br>Supporting: Phenotype specific for disease with single genetic etiology. Proband(s) with specificity score of 1-1.5
RAD51C	x		
RAD51D	x		
STK11	x		
TP53		x	There are two widely used criteria used for assessing the likelihood of LFS; Classical and Chompret criteria with the Chompret criteria being less restrictive. Individuals meeting Revised Chompret criteria have an estimated ~30% risk of harboring a pathogenic TP53 variant (Bougeard, et al 2015; PMID: 26014290).<br>Members of the TP53 VCEP calculated likelihood ratios for a patient meeting Classic LFS or Revised Chompret criteria using multigene panel testing from Ambry Genetics Laboratory. Our data showed that individuals meeting Revised Chompret criteria had a LR of >2.08 to 4.3 to <18.7<br>Therefore, we recommend using the following point system for determining the weight of PS4 evidence:<br>Proband meeting Revised Chompret criteria = 0.5 point<br>Proband meeting classic LFS criteria = 1 point<br>Strong: 4 or more points<br>Moderate: 2-3 points<br>Supporting: 1 point<br>This rule code cannot be applied when a variant also meets BA1 or BS1 criteria.
CC score: Cleveland Clinic score<br>CI: Confidence interval<br>LFS: Li-Fraumeni syndrome<br>LR: Likelihood ratio<br>HDGC: Hereditary Diffuse Gastric Cancer<br>OR: Odds ratio<br>PHTS: PTEN Hamartoma Tumor Syndrome<br>VCEP: Variant Curation Expert Panel			

Table S3: Primer sequences for functional tests.

**Gene**	**Forward 5'-3'**	**Length forward**	**Reverse 5'-3'**	**Length reverse**
ATM (exon 1-24)	TTGACCTTCCGAGTGCAGTG	20	ACACAGGGCAAACAAAGCCT	20
ATM (exon 22-43)	CACCAAGTTCGCATGTTGGC	20	TGCATATTCCTCCATGCTGCT	21
BRIP1	CCTCCAGACAGTTAGGAATCTGA	23	TGAGGGCATGATCCAAACGA	20
CHEK2	CTGAGGCTGCGGAGAGTG	18	GTGTTCAAACCACGGAGTTCA	21
RAD51C	TCCGGGGTTAGCAGGTGA	18	ACACTTTGAGATTTGTTTCTGGGTT	25

Table S4: Classification and frequencies of all the identified likely pathogenic and pathogenic variants found in our study populaiton of 5,923 patients with a clinical suspicion of predispostion to heredirary breast- and/or ovarian cancer.
ACMG: American College of Medical Genetics
AG: Acceptor gain
AL: Acceptor loss
AT/A-T: Ataxia-Telangiectasia
BC: Breast cancer
BRCT: BRCA1 C Terminus
ccDK: Cancer (variant) classification DK (The variants classified by this group were not assigned ACMG criteria)
DG: Donor gain
DGC: Differentiated glioblastoma cells
DL: Donor loss
DNE: Dominante-negative effect
ENIGMA: Evidence-based Network for the Interpretation of Germline Mutant Alleles (The variants classified by this group were not assigned ACMG criteria)
GnomAD: Genome Aggregation Database
HDGC: Hereditary diffuse gastric cancer
HDR: Homology-directed repair
HGMD: The Human Gene Mutation Database
HR: Homologous recombination
HRD:Homologous recombination deficiency
LBC: Leukaemia and blood cancer
LOF: Loss of function
mESC: Mouse embryonic stem cell
NMD: Nonsense-mediated decay PTC: Premature termination codons
OC: Ovarian cancer
SRC: Signet ring cell
SS: Splice site
SSF:SpliceSiteFinder
XRCC2: X-ray repair cross complementing 2

Gene	Sequence Ontology	HGVS c.	HGVS p.	Classification	Assigned ACMG criteria	Comments	#Samples_Total (N=5,923)	#Hom samples_Total (N=5,923)	#Samples_BC (N=3,706)	#Hom samples_BC (N=3,706)	#Samples_OC (N=890)	#Hom samples_OC (N=890)
ATM	splice_donor_variant	NM_000051.4:c.1235_1235+10delGGTAAAGTGTT	NP_000042.3:p.?	Likely Pathogenic	PVS1 (Exon skipping, disrupts reading frame, predicted to undergo NMD)<br>PM2_supporting (Absent from GnomAD)	SpliceAI AL: 0.51 and DL: 0.86. Possibly exon skipping. RAW score donor site changes from 0.85 to 0 and acceptor site changes from 0.71 to 0.20	1	0	0	0	0	0
ATM	stop_gained	NM_000051.4:c.1304T>G	NP_000042.3:p.Leu435Ter	Pathogenic	PVS1 (Nonsense variant predicted to undergo NMD)<br>PM2_supporting (Absent from GnomAD)<br>PM5_supporting (Frameshift variant with PTC upstream p.3047)		1	0	0	0	1	0
ATM	frameshift_variant	NM_000051.4:c.1564_1565delGA	NP_000042.3:p.Glu522Ilefs*43	Pathogenic	PVS1 (Frameshift variant predicted to undergo NMD)<br>PM5_supporting (Frameshift variant with PTC upstream p.3047)		1	0	1	0	0	0
ATM	splice_donor_variant	NM_000051.4:c.1898+2T>G		Pathogenic	PVS1_strong (Splice donor variant in exon 12, predicted exon skipping or cryptic splice site, but preservation of reading frame)<br>PM3_strong: (Variant found in A-T patients in trans with c.5825C>T (PMID: 24090759) and another found in trans with c.484 C>T (PMID: 23566627))	SpliceAI: AL:0.57. DG: 0.49 and DL: 1.00. Possibly exon skipping or partiel exon deletion. RAW score natural acceptor changes from 0.99 to 0.42, natural donor changes from 0.99 to 0 and alternative donor changes from 0.02 to 0.57	1	0	1	0	0	0
ATM	synonymous_variant	NM_000051.4:c.2250G>A	NP_000042.3:p.Lys750=	Likely Pathogenic	PP3 (SpliceAI DL: 0.94 and AL: 0.74. Possibly exon skipping. RAW score donor changes from 0.99 to 0.06 and acceptor changes from 0.99 to 0.22)<br>PM3_strong (Variant found in 5 patients from A-T families witn unknown phase (PMID: 10980530. PMID: 9463314 and PMID: 9887333))<br>PS3_moderate (A-T patients and radiosensitivitet 18% (PMID: 18634022))		1	0	1	0	0	0
ATM	frameshift_variant	NM_000051.4:c.2284_2285delCT	NP_000042.3:p.Leu762Valfs*2	Pathogenic	PVS1 (Frameshift variant predicted to undergo NMD)<br>PM3_strong (A-T patient with the variant in homozygote state (PMID: 8845835) and 3 patients in 2 different families with the variant in compund heterozygote state (PMID: 9463314))		1	0	1	0	0	0
ATM	splice_donor_variant	NM_000051.4:c.3284+1G>A		Likely Pathogenic	PVS1 (Exon skipping or cryptic splice site that disrupts reading frame and predicted to undergo NMD)<br>PM2_supporting (Absent from GnomAD)	SpliceAI DL: 0.99 and AL: 0.47. Possibly exon skipping. RAW score donor changes from 0.99 to 0 and acceptor changes from 0.99 to 0.52	1	0	0	0	0	0
ATM	stop_gained	NM_000051.4:c.3663G>A	NP_000042.3:p.Trp1221Ter	Pathogenic	PVS1 (Nonsense variant predicted to undergo NMD)<br>PM5_supporting (Truncating variant with PTC upstream p.R3047)<br>PM2_supporting (GnomAD frequency 0.00000314)		1	0	1	0	0	0
ATM	frameshift_variant	NM_000051.4:c.3712_3716delTTATT	NP_000042.3:p.Leu1238Lysfs*6	Pathogenic	PVS1 (Frameshift variant predicted to undergo NMD)<br>PM5_supporting (Frameshift variant with PTC upstream p.R3047)<br>PM2_supporting (GnomAD frequency 0.00000912)		1	0	0	0	0	0
ATM	stop_gained	NM_000051.4:c.3826C>T	NP_000042.3:p.Gln1276Ter	Pathogenic	PVS1 (Nonsense variant predicted to undergo NMD)<br>PM2_supporting (Absent from GnomAD)<br>PM5_supporting (Frameshift variant with PTC upstream p.3047)		1	0	0	0	1	0
ATM	splice_donor_variant	NM_000051.4:c.3993+1G>A		Pathogenic	PVS1_strong (Cryptic SS that preserves reading frame)<br>PM3_strong (3 unrelated A-T patients with the variant in trans (PMID: 10980530 and 12815592))	SpliceAI DL: 1.00 and DG: 0.76. Possibly partiel exon deletion. RAW score naturel donor site changes from 0.99 to 0 and alternative donor site changes from 0.19 to 0.95	9	0	8	0	0	0
ATM	splice_acceptor_variant	NM_000051.4:c.3994-2A>G		Likely Pathogenic	PVS1 (Splice acceptor variant predicted to result in exon skipping or cryptic splice site that disrupt reading frame)<br>PM2_supporting (GnomAD frequency only 0.00000487)	SpliceAI AL: 0.99. AG: 0.42 and DL: 0.63. Possibly exon skipping or partiel exon deletion. RAW score natural donor changes from 0.99 to 0.36, natural acceptor changes from 0.99 to 0 and alternative acceptor changes from 0.03 to 0.45	1	0	0	0	0	0
ATM	frameshift_variant	NM_000051.4:c.4335_4338delTGTT	NP_000042.3:p.Phe1445Leufs*5	Pathogenic	PVS1 (Frameshift variant predicted to undergo NMD)<br>PM2_supporting (Absent from GnomAD)<br>PM5_supporting (Frameshift variant with PTC upstream p.R3047)		1	0	1	0	0	0
ATM	frameshift_variant	NM_000051.4:c.4632_4635delCTTA	NP_000042.3:p.Tyr1544Ter	Pathogenic	ccDK		8	0	7	0	0	0
ATM	stop_gained	NM_000051.4:c.5326G>T	NP_000042.3:p.Glu1776Ter	Pathogenic	PVS1 (Nonsense variant predicted to undergo NMD)<br>PM2_supporting (Absent from GnomAD)<br>PM5_supporting (Nonsense variant with PTC upstream p.3047)		4	0	3	0	1	0
ATM	frameshift_variant	NM_000051.4:c.5405dupA	NP_000042.3:p.His1802Glnfs*2	Pathogenic	PVS1 (Frameshift variant predicted to undergo NMD)<br>PM2_supporting (Absent from GnomAD)<br>PM5_supporting (Frameshift variant with PTC upstream p.R3047)		2	0	1	0	0	0
ATM	stop_gained	NM_000051.4:c.601C>T	NP_000042.3:p.Gln201Ter	Pathogenic	PVS1 (Nonsense variant predicted to undergo NMD)<br>PM2_supporting (Only two in GnomAD)<br>PM5_supporting (Nonsense variant with PTC upstream p.R3047)		1	0	1	0	0	0
ATM	frameshift_variant	NM_000051.4:c.6498_6499delGT	NP_000042.3:p.Tyr2167Phefs*7	Pathogenic	PVS1 (Frameshift variant predicted to undergo NMD upsteam p.Glu2979)<br>PM5_supporting (PTC upstream p.Arg3047)<br>PM2_supporting (Absent from GnomAD 2.1.)		1	0	1	0	0	0
ATM	frameshift_variant	NM_000051.4:c.7592dupT	NP_000042.3:p.Met2531Ilefs*9	Likely Pathogenic	PVS1 (Frameshift variant predicted to undergo NMD)<br>PM2_supporting (Absent from GnomAD)		1	0	0	0	0	0
ATM	splice_donor_variant	NM_000051.4:c.7629_7629+4delTGTAA	NP_000042.3:p.?	Pathogenic	ccDK		5	0	2	0	0	0
ATM	in-frame_deletion	NM_000051.4:c.7638_7646delTAGAATTTC	NP_000042.3:p.Arg2547_Ser2549del	Pathogenic	PS3_supporting (Functional study showing inactive ATM kinase (PMID: 19431188))<br>PM3_very strong (Multiple A-T patients affected by the variant, either unknown, homozygote or in trans phase (PMID: 9443866, PMID: 8845835, PMID: 9150358, PMID: 10817650, PMID: 9463314 and PMID: 12552559))<br>PM2_supporting (Absent from GnomAD 2.1.)		1	0	0	0	0	0
ATM	missense_variant	NM_000051.4:c.8147T>C	NP_000042.3:p.Val2716Ala	Pathogenic	PP3 (Revel=0,905)<br>PS3_moderate (Functional study showing failing to elicit ATM kinase activity and failed to alter the extent of radiosensitivity (PMID: 11805335))<br>PM3_very strong (In ClinVar laboratories reported A-T patients with the variant in trans (PMID: 11805335, PMID: 25957637 and PMID: 21965147) or unknown phase (PMID: 21354641))		1	0	1	0	0	0

ATM	missense_variant	NM_000051.4:c.8494C>T	NP_000042.3:p.Arg2832Cys	Pathogenic	PS3_moderate (Functional studies showing the variant results in significant reduction of ATM protein expression and kinase activity and intermediate radiosensitivity (PMID: 10873394, 18634022, 19431188, 22017321)<br>PM3_very strong (Variant found in A-T patients: 3 A-T patients observeed in compound heterozygot phase (PMID: 10873394), 1 A-T patient observered in compound heterozygote phase (PMID: 12552559),2 A-T patients observered in compound heterozygot phase (PMID: 12673797), 1 A-T patient observered in compound heterozygot and 1 A-T patient observered in homozygot phase (PMID: 19431188) and 1 A-T patient observered in compound heterozygote phase (PMID: 22017321))<br>PP3 (Revel=0.83)		1	0	0	0	0	0
ATM	splice_donor_variant	NM_000051.4:c.8786+1G>A		Pathogenic	ccDK		2	0	1	0	1	0
ATM	splice_acceptor_variant	NM_000051.4:c.8787-2A>G		Likely Pathogenic	PVS1 (Exon skipping or cryptic splice site that disrupts the reading frame and predicted to undergo NMD)<br>PM2_supporting (Absent from GnomAD)	SpliceAI AL: 0.86 and DL: 0.93. Possibly exon skipping. RAW score donor changes from 0.94 to 0.01 and acceptor changes from 0.86 to 0.00	1	0	1	0	0	0
ATM	stop_gained	NM_000051.4:c.923G>A	NP_000042.3:p.Trp308Ter	Pathogenic	PVS1 (Nonsense variant predicted to undergo NMD)<br>PM2_supporting (Absent from GnomAD)<br>PM5_supporting (Nonsense variant with PTC upstream p.3047)	AL: 0.27 and DL: 0.37. Possibly exon skipping. RAW score acceptor changes from 0.96 to 0.69 and donor changes from 0.97 to 0.82	1	0	0	0	1	0
ATM	frameshift_variant	NM_000051.4:c.943_944delTT	NP_000042.3:p.Leu315Ilefs*2	Pathogenic	PVS1 (Frameshift variant predicted to undergo NMD)<br>PM2_supporting (GnomAD 0.00000136925)<br>PM5_supporting (Frameshift variant with PTC upstream p.3047)		1	0	0	0	0	0
BARD1	frameshift_variant	NM_000465.4:c.1321_1324delATAC	NP_000456.2:p.Ile441Leufs*33	Likely Pathogenic	PVS1 (Frameshift variant predicted to undergo NMD)<br>PM2_supporting (Absent from GnomAD)		1	0	1	0	0	0
BARD1	stop_gained	NM_000465.4:c.1690C>T	NP_000456.2:p.Gln564Ter	Pathogenic	PVS1 (Nonsense variant predicted to undergo NMD)<br>PM1 (Located in BRCT domain)<br>PP5 (Laboratories reported it pathogenic in ClinVar)		1	0	1	0	0	0
BARD1	stop_gained	NM_000465.4:c.2143C>T	NP_000456.2:p.Gln715Ter	Likely Pathogenic	PVS1_strong (Nonsense variant not predicted to undergo NMD, but critical to protein function)<br>PM2_supporting (Only 1 in GnomAD)<br>PM1 (Located in BRCT domain)<br>PP5 (Laboratories reported it as pathogenic in ClinVar)		1	0	1	0	0	0
BARD1	stop_gained	NM_000465.4:c.298C>T	NP_000456.2:p.Gln100Ter	Likely Pathogenic	PVS1 (Nonsense variant predicted to undergo NMD)<br>PP5 (Laboratories reported it is pathogenic in ClinVar)		1	0	1	0	0	0
BRCA1	stop_gained	NM_007294.4:c.1012A>T	NP_009225.1:p.Lys338Ter	Pathogenic	ENIGMA		1	0	0	0	0	0
BRCA1	frameshift_variant	NM_007294.4:c.1015_1016delAA	NP_009225.1:p.Lys339Glyfs*6	Pathogenic	ccDK		1	0	1	0	0	0
BRCA1	stop_gained	NM_007294.4:c.1058G>A	NP_009225.1:p.Trp353Ter	Pathogenic	ENIGMA		1	0	0	0	1	0
BRCA1	stop_gained	NM_007294.4:c.1059G>A	NP_009225.1:p.Trp353Ter	Pathogenic	ENIGMA		1	0	1	0	0	0
BRCA1	frameshift_variant	NM_007294.4:c.1082_1092delCAGAGAATCCT	NP_009225.1:p.Ser361Ter	Pathogenic	ENIGMA		1	0	0	0	1	0
BRCA1	missense_variant	NM_007294.4:c.115T>G	NP_009225.1:p.Cys39Gly	Pathogenic	ccDK		3	0	2	0	0	0
BRCA1	frameshift_variant	NM_007294.4:c.1265_1266dupAT	NP_009225.1:p.Ser423Ilefs*8	Pathogenic	ENIGMA		1	0	0	0	1	0
BRCA1	missense_variant	NM_007294.4:c.130T>A	NP_009225.1:p.Cys44Ser	Pathogenic	ENIGMA		5	0	2	0	1	0
BRCA1	splice_region_variant	NM_007294.4:c.134+5G>C		Likely Pathogenic	PS3 (Reported by one calibrated study incorporating mRNA splicing effects to exhibit protein function similar to pathogenic control variants (PMID:30209399))<br>PP3 (SpliceAI DL:0.92 and AL: 0.83. Possibly exon skipping. RAW score donor changes from 0.98 to 0.06 and acceptor changes from 0.96 to 0.13.)<br>PM2_supporting (Absent from GnomAD)		1	0	1	0	0	0
BRCA1	missense_variant	NM_007294.4:c.140G>A	NP_009225.1:p.Cys47Tyr	Pathogenic	ENIGMA		1	0	1	0	0	0
BRCA1	missense_variant	NM_007294.4:c.143T>G	NP_009225.1:p.Met48Arg	Likely Pathogenic	ccDK		1	0	1	0	0	0
BRCA1	frameshift_variant	NM_007294.4:c.1556delA	NP_009225.1:p.Lys519Argfs*13	Pathogenic	ENIGMA		3	0	2	0	0	0
BRCA1	stop_gained	NM_007294.4:c.1687C>T	NP_009225.1:p.Gln563Ter	Pathogenic	ENIGMA		6	0	3	0	4	0
BRCA1	missense_variant	NM_007294.4:c.181T>G	NP_009225.1:p.Cys61Gly	Pathogenic	ENIGMA		11	0	7	0	2	0
BRCA1	missense_variant	NM_007294.4:c.190T>C	NP_009225.1:p.Cys64Arg	Likely Pathogenic	ccDK		2	0	1	0	1	0
BRCA1	frameshift_variant	NM_007294.4:c.1953_1956delGAAA	NP_009225.1:p.Lys653Serfs*47	Pathogenic	ENIGMA		1	0	0	0	1	0
BRCA1	frameshift_variant	NM_007294.4:c.1961delA	NP_009225.1:p.Lys654Serfs*47	Pathogenic	ENIGMA		1	0	1	0	0	0
BRCA1	frameshift_variant	NM_007294.4:c.2110_2111delAA	NP_009225.1:p.Asn704Cysfs*7	Pathogenic	ENIGMA		1	0	0	0	1	0
BRCA1	frameshift_variant	NM_007294.4:c.2216_2217delAA	NP_009225.1:p.Lys739Serfs*3	Pathogenic	ENIGMA		1	0	1	0	0	0
BRCA1	frameshift_variant	NM_007294.4:c.2475delC	NP_009225.1:p.Asp825Glufs*21	Pathogenic	ENIGMA		39	0	23	0	11	0
BRCA1	frameshift_variant	NM_007294.4:c.2524dupG	NP_009225.1:p.Glu842Glyfs*10	Pathogenic	ENIGMA		1	0	1	0	0	0
BRCA1	frameshift_variant	NM_007294.4:c.2779dupG	NP_009225.1:p.Ala927Glyfs*11	Pathogenic	PVS1 (Frameshift variant predicted to undergo NMD)<br>PM5_strong (PTC in exon 10(11))		1	0	0	0	1	0
BRCA1	stop_gained	NM_007294.4:c.2800C>T	NP_009225.1:p.Gln934Ter	Pathogenic	ENIGMA		1	0	0	0	0	0
BRCA1	frameshift_variant	NM_007294.4:c.2866_2870delTCTCA	NP_009225.1:p.Ser956Valfs*13	Pathogenic	ENIGMA		1	0	0	0	0	0
BRCA1	frameshift_variant	NM_007294.4:c.3048_3052dupTGAGA	NP_009225.1:p.Asn1018Metfs*8	Pathogenic	ENIGMA		3	0	2	0	1	0
BRCA1	frameshift_variant	NM_007294.4:c.3247_3251delATGCT	NP_009225.1:p.Met1083Ter	Pathogenic	ENIGMA		1	0	1	0	0	0
BRCA1	frameshift_variant	NM_007294.4:c.3262dupG	NP_009225.1:p.Val1088Glyfs*5	Pathogenic	PVS1 (Frameshift variant predicted to undergo NMD and be in a biologically relevant transcript)<br>PM5_strong (PTC in exon 10 (11))		1	0	1	0	1	0
BRCA1	stop_gained	NM_007294.4:c.3319G>T	NP_009225.1:p.Glu1107Ter	Pathogenic	ENIGMA		14	0	5	0	8	0
BRCA1	stop_gained	NM_007294.4:c.3400G>T	NP_009225.1:p.Glu1134Ter	Pathogenic	ENIGMA		2	0	0	0	2	0
BRCA1	frameshift_variant	NM_007294.4:c.3477_3480delAAAG	NP_009225.1:p.Ile1159Metfs*50	Pathogenic	ENIGMA		1	0	0	0	0	0
BRCA1	frameshift_variant	NM_007294.4:c.3478_3479delAA	NP_009225.1:p.Lys1160Glyfs*4	Pathogenic	ENIGMA		2	0	0	0	2	0
BRCA1	frameshift_variant	NM_007294.4:c.3481_3491delGAAGATACTAG	NP_009225.1:p.Glu1161Phefs*3	Pathogenic	ENIGMA		1	0	0	0	0	0
BRCA1	stop_gained	NM_007294.4:c.3607C>T	NP_009225.1:p.Arg1203Ter	Pathogenic	ENIGMA		4	0	3	0	0	0
BRCA1	stop_gained	NM_007294.4:c.3640G>T	NP_009225.1:p.Glu1214Ter	Pathogenic	ENIGMA		1	0	1	0	0	0
BRCA1	frameshift_variant	NM_007294.4:c.3665delA	NP_009225.1:p.Glu1222Glyfs*13	Pathogenic	PVS1(frameshift variant predicted to undergo NMD)<br>PM5_strong (PTC in exon 10(11))		1	0	1	0	0	0
BRCA1	frameshift_variant	NM_007294.4:c.3700_3704delGTAAA	NP_009225.1:p.Val1234Glnfs*8	Pathogenic	ENIGMA		9	0	4	0	2	0
BRCA1	frameshift_variant	NM_007294.4:c.3710delT	NP_009225.1:p.Ile1237Asnfs*27	Pathogenic	ENIGMA		3	0	0	0	2	0
BRCA1	stop_gained	NM_007294.4:c.3718C>T	NP_009225.1:p.Gln1240Ter	Pathogenic	ENIGMA		1	0	0	0	0	0
BRCA1	frameshift_variant	NM_007294.4:c.3869_3870delAA	NP_009225.1:p.Lys1290Metfs*4	Pathogenic	ENIGMA		1	0	0	0	0	0
BRCA1	frameshift_variant	NM_007294.4:c.3874delT	NP_009225.1:p.Ser1292Leufs*15	Pathogenic	ENIGMA		5	0	5	0	0	0
BRCA1	stop_gained	NM_007294.4:c.3904G>T	NP_009225.1:p.Glu1302Ter	Pathogenic	ENIGMA		3	0	1	0	1	0
BRCA1	stop_gained	NM_007294.4:c.4033G>T	NP_009225.1:p.Glu1345Ter	Pathogenic	ENIGMA		1	0	1	0	0	0
BRCA1	frameshift_variant	NM_007294.4:c.4035delA	NP_009225.1:p.Glu1346Lysfs*20	Pathogenic	ENIGMA		1	0	0	0	1	0
BRCA1	frameshift_variant	NM_007294.4:c.4146_4155dupCTCAGGGCTA	NP_009225.1:p.Ser1386Leufs*8	Pathogenic	ENIGMA		1	0	1	0	0	0
BRCA1	splice_donor_variant	NM_007294.4:c.4185+1G>A		Pathogenic	ccDK		1	0	0	0	1	0
BRCA1	stop_gained	NM_007294.4:c.427G>T	NP_009225.1:p.Glu143Ter	Pathogenic	ENIGMA		3	0	1	0	0	0
BRCA1	stop_gained	NM_007294.4:c.4327C>T	NP_009225.1:p.Arg1443Ter	Pathogenic	ENIGMA		2	0	1	0	0	0
BRCA1	frameshift_variant	NM_007294.4:c.4684_4685delCC	NP_009225.1:p.Pro1562Leufs*11	Pathogenic	ENIGMA		1	0	0	0	0	0
BRCA1	frameshift_variant	NM_007294.4:c.4685dupC	NP_009225.1:p.Tyr1563Leufs*11	Pathogenic	ccDK		1	0	1	0	0	0
BRCA1	stop_gained	NM_007294.4:c.4689C>G	NP_009225.1:p.Tyr1563Ter	Pathogenic	ENIGMA		1	0	1	0	0	0
BRCA1	missense_variant	NM_007294.4:c.4964C>T	NP_009225.1:p.Ser1655Phe	Likely Pathogenic	ccDK		1	0	1	0	0	0
BRCA1	missense_variant	NM_007294.4:c.5089T>C	NP_009225.1:p.Cys1697Arg	Pathogenic	ENIGMA		7	0	3	0	2	0
BRCA1	missense_variant	NM_007294.4:c.5096G>A	NP_009225.1:p.Arg1699Gln	Pathogenic	ENIGMA		12	0	5	0	4	0
BRCA1	missense_variant	NM_007294.4:c.5143A>C	NP_009225.1:p.Ser1715Arg	Pathogenic	ENIGMA		2	0	1	0	0	0
BRCA1	splice_acceptor_variant	NM_007294.4:c.5153-1G>C		Pathogenic	ENIGMA		1	0	0	0	1	0
BRCA1	missense_variant	NM_007294.4:c.5213G>A	NP_009225.1:p.Gly1738Glu	Pathogenic	ccDK		2	0	1	0	1	0
BRCA1	stop_gained	NM_007294.4:c.5251C>T	NP_009225.1:p.Arg1751Ter	Pathogenic	ENIGMA		1	0	0	0	0	0
BRCA1	frameshift_variant	NM_007294.4:c.5266dupC	NP_009225.1:p.Gln1756Profs*74	Pathogenic	ENIGMA		13	0	9	0	3	0
BRCA1	missense_variant	NM_007294.4:c.5297T>A	NP_009225.1:p.Ile1766Asn	Likely Pathogenic	ccDK		1	0	1	0	0	0
BRCA1	splice_donor_variant	NM_007294.4:c.5406+1G>A		Likely Pathogenic	ccDK		1	0	1	0	0	0
BRCA1	splice_acceptor_variant	NM_007294.4:c.5407-1G>T		Likely Pathogenic	ccDK		2	0	1	0	0	0
BRCA1	stop_gained	NM_007294.4:c.5503C>T	NP_009225.1:p.Arg1835Ter	Pathogenic	ENIGMA		4	0	1	0	0	0
BRCA1	frameshift_variant	NM_007294.4:c.68_69delAG	NP_009225.1:p.Glu23Valfs*17	Pathogenic	ENIGMA		3	0	1	0	0	0
BRCA1	frameshift_variant	NM_007294.4:c.843_846delCTCA	NP_009225.1:p.Ser282Tyrfs*15	Pathogenic	ENIGMA		1	0	0	0	0	0

BRCA1	frameshift_variant	NM_007294.4:c.923_924delGC	NP_009225.1:p.Ser308Lysfs*11	Pathogenic	ENIGMA		1	0	1	0	0	0
BRCA1	stop_gained	NM_007294.4:c.967G>T	NP_009225.1:p.Gly323Ter	Pathogenic	ccDK		1	0	0	0	1	0
BRCA2	frameshift_variant	NM_000059.4:c.1310_1313delAAGA	NP_000050.3:p.Lys437Ilefs*22	Pathogenic	ENIGMA		8	0	4	0	0	0
BRCA2	stop_gained	NM_000059.4:c.145G>T	NP_000050.3:p.Glu49Ter	Pathogenic	ENIGMA		2	0	1	0	1	0
BRCA2	frameshift_variant	NM_000059.4:c.1813delA	NP_000050.3:p.Ile605Tyrfs*9	Pathogenic	ENIGMA		2	0	2	0	0	0
BRCA2	frameshift_variant	NM_000059.4:c.1813dupA	NP_000050.3:p.Ile605Asnfs*11	Pathogenic	ENIGMA		1	0	0	0	1	0
BRCA2	stop_gained	NM_000059.4:c.1819A>T	NP_000050.3:p.Lys607Ter	Pathogenic	ENIGMA		1	0	1	0	0	0
BRCA2	stop_gained	NM_000059.4:c.1855C>T	NP_000050.3:p.Gln619Ter	Pathogenic	ENIGMA		1	0	0	0	0	0
BRCA2	stop_gained	NM_000059.4:c.2231C>G	NP_000050.3:p.Ser744Ter	Pathogenic	ENIGMA		1	0	0	0	0	0
BRCA2	frameshift_variant	NM_000059.4:c.2450delA	NP_000050.3:p.Lys817Argfs*8	Pathogenic	ENIGMA		1	0	1	0	0	0
BRCA2	frameshift_variant	NM_000059.4:c.2808_2811delACAA	NP_000050.3:p.Ala938Profs*21	Pathogenic	ENIGMA		2	0	1	0	0	0
BRCA2	stop_gained	NM_000059.4:c.2830A>T	NP_000050.3:p.Lys944Ter	Pathogenic	ENIGMA		4	0	3	0	2	0
BRCA2	stop_gained	NM_000059.4:c.289G>T	NP_000050.3:p.Glu97Ter	Pathogenic	ENIGMA		1	0	1	0	0	0
BRCA2	splice_donor_variant	NM_000059.4:c.316+1G>T		Pathogenic	ENIGMA		2	0	1	0	0	0
BRCA2	splice_region_variant	NM_000059.4:c.316+5G>A		Pathogenic	ENIGMA		2	0	2	0	0	0
BRCA2	frameshift_variant	NM_000059.4:c.3530_3533delACAG	NP_000050.3:p.Asp1177Alafs*19	Pathogenic	ENIGMA		7	0	4	0	1	0
BRCA2	frameshift_variant	NM_000059.4:c.3847_3848delGT	NP_000050.3:p.Val1283Lysfs*2	Pathogenic	ENIGMA		4	0	3	0	1	0
BRCA2	frameshift_variant	NM_000059.4:c.4139_4140dupTT	NP_000050.3:p.Lys1381Leufs*8	Pathogenic	ENIGMA		1	0	1	0	0	0
BRCA2	frameshift_variant	NM_000059.4:c.4399delC	NP_000050.3:p.His1467Ilefs*4	Pathogenic	PVS1 (Frameshift variant predicted to undergo NMD and be in biologically relevant transcript)<br>PM5_strong (PTC in exon 11)		1	0	1	0	1	0
BRCA2	frameshift_variant	NM_000059.4:c.4570_4573delTTTC	NP_000050.3:p.Phe1524Ilefs*18	Pathogenic	PVS1 (Frameshift variant predicted to undergo NMD and be in biologically relevant transcript)<br>PM5_strong (PTC in exon 11)		1	0	1	0	0	0
BRCA2	frameshift_variant	NM_000059.4:c.469_470delAA	NP_000050.3:p.Lys157Valfs*25	Pathogenic	ENIGMA		2	0	2	0	0	0
BRCA2	frameshift_variant	NM_000059.4:c.5073dupA	NP_000050.3:p.Trp1692Metfs*3	Pathogenic	ENIGMA		1	0	1	0	0	0
BRCA2	frameshift_variant	NM_000059.4:c.5164_5165delAG	NP_000050.3:p.Ser1722Tyrfs*4	Pathogenic	ENIGMA		4	0	2	0	1	0
BRCA2	frameshift_variant	NM_000059.4:c.5213_5216delCTTA	NP_000050.3:p.Thr1738Ilefs*2	Pathogenic	ccDK		1	0	1	0	0	0
BRCA2	frameshift_variant	NM_000059.4:c.5217_5223delTTTAAGT	NP_000050.3:p.Tyr1739Ter	Pathogenic	ENIGMA		1	0	0	0	1	0
BRCA2	frameshift_variant	NM_000059.4:c.5307_5311delTTCTG	NP_000050.3:p.Asp1769Glufs*3	Pathogenic	PVS1 (Frameshift variant predicted to undergo NMD in exon 11)<br>PM5_strong (PTC in biologically transcript)<br>PM2_supporting (Absent from GnomAD)		1	0	1	0	0	0
BRCA2	frameshift_variant	NM_000059.4:c.5352delC	NP_000050.3:p.Asn1784Lysfs*7	Pathogenic	ENIGMA		3	0	2	0	1	0
BRCA2	frameshift_variant	NM_000059.4:c.5576_5579delTTAA	NP_000050.3:p.Ile1859Lysfs*3	Pathogenic	ENIGMA		2	0	1	0	1	0
BRCA2	stop_gained	NM_000059.4:c.5645C>A	NP_000050.3:p.Ser1882Ter	Pathogenic	ENIGMA		3	0	2	0	2	0
BRCA2	stop_gained	NM_000059.4:c.5682C>G	NP_000050.3:p.Tyr1894Ter	Pathogenic	ENIGMA		2	0	1	0	1	0
BRCA2	frameshift_variant	NM_000059.4:c.5722_5723delCT	NP_000050.3:p.Leu1908Argfs*2	Pathogenic	ENIGMA		2	0	2	0	1	0
BRCA2	frameshift_variant	NM_000059.4:c.5754_5755delTA	NP_000050.3:p.His1918Glnfs*5	Pathogenic	ENIGMA		1	0	1	0	0	0
BRCA2	frameshift_variant	NM_000059.4:c.5845delG	NP_000050.3:p.Asp1949Metfs*14	Pathogenic	PVS1 (Frameshift variant predicted to undergo NMD in exon 11)<br>PM5_strong (PTC in biologically transcript)<br>PM2_supporting (Absent from GnomAD)		1	0	1	0	0	0
BRCA2	frameshift_variant	NM_000059.4:c.6275_6276delTT	NP_000050.3:p.Leu2092Profs*7	Pathogenic	ENIGMA		4	0	3	0	2	0
BRCA2	splice_region_variant	NM_000059.4:c.631+4A>G		Likely Pathogenic	ccDK		1	0	1	0	0	0
BRCA2	frameshift_variant	NM_000059.4:c.6373delA	NP_000050.3:p.Thr2125Profs*12	Pathogenic	ENIGMA		8	0	2	0	1	0
BRCA2	frameshift_variant	NM_000059.4:c.6373dupA	NP_000050.3:p.Thr2125Asnfs*4	Pathogenic	ENIGMA		1	0	1	0	1	0
BRCA2	frameshift_variant	NM_000059.4:c.6405_6409delCTTAA	NP_000050.3:p.Asn2135Lysfs*3	Pathogenic	ENIGMA		1	0	1	0	0	0
BRCA2	frameshift_variant	NM_000059.4:c.6408_6414delAAATGTT	NP_000050.3:p.Asn2137Lysfs*29	Pathogenic	ENIGMA		1	0	0	0	0	0
BRCA2	frameshift_variant	NM_000059.4:c.6486_6489delACAA	NP_000050.3:p.Lys2162Asnfs*5	Pathogenic	ENIGMA		13	0	7	0	5	0
BRCA2	frameshift_variant	NM_000059.4:c.6594_6595delAA	NP_000050.3:p.Glu2198Aspfs*4	Pathogenic	ccDK		1	0	1	0	0	0
BRCA2	missense_variant	NM_000059.4:c.7007G>A	NP_000050.3:p.Arg2336His	Pathogenic	ENIGMA		1	0	1	0	0	0
BRCA2	splice_acceptor_variant	NM_000059.4:c.7008-1G>A		Pathogenic	ENIGMA		1	0	0	0	0	0
BRCA2	frameshift_variant	NM_000059.4:c.7069_7070delCT	NP_000050.3:p.Leu2357Valfs*2	Pathogenic	ENIGMA		7	0	5	0	1	0
BRCA2	frameshift_variant	NM_000059.4:c.7097dupT	NP_000050.3:p.Thr2367Aspfs*25	Pathogenic	ENIGMA		15	0	7	0	6	0
BRCA2	frameshift_variant	NM_000059.4:c.7152delA	NP_000050.3:p.Val2385Phefs*9	Pathogenic	ENIGMA		1	0	1	0	0	0
BRCA2	Disruptive in-frame deletion	NM_000059.4:c.72_85delinsTTTAAATAGAT	NP_000050.3:p.Leu24_Leu29delinsPheLeuAsnArgPhe	Likely Pathogenic	PP3 (Located in functional domain and BayesDel=0.6431)<br>PM2_supporting (Absent from GnomAD)<br>PS3 (mESC assay in-frame changes in protein sequence and suggested a different absolute level of exon 3 (PMID: 35979650))		1	0	1	0	0	0
BRCA2	frameshift_variant	NM_000059.4:c.7251_7252delCA	NP_000050.3:p.His2417Glnfs*3	Pathogenic	ENIGMA		1	0	1	0	0	0
BRCA2	stop_gained	NM_000059.4:c.7501C>T	NP_000050.3:p.Gln2501Ter	Pathogenic	ENIGMA		2	0	2	0	0	0
BRCA2	splice_donor_variant	NM_000059.4:c.7617+1G>A		Pathogenic	ENIGMA		15	0	11	0	5	0
BRCA2	frameshift_variant	NM_000059.4:c.771_775delTCAAA	NP_000050.3:p.Asn257Lysfs*17	Pathogenic	ENIGMA		1	0	1	0	0	0
BRCA2	missense_variant	NM_000059.4:c.7878G>C	NP_000050.3:p.Trp2626Cys	Pathogenic	ENIGMA		6	0	4	0	1	0
BRCA2	missense_variant	NM_000059.4:c.7879A>T	NP_000050.3:p.Ile2627Phe	Pathogenic	ENIGMA		1	0	1	0	0	0
BRCA2	frameshift_variant	NM_000059.4:c.7913_7917delTTCCT	NP_000050.3:p.Phe2638Ter	Pathogenic	ENIGMA		1	0	1	0	0	0
BRCA2	splice_region_variant	NM_000059.4:c.7976+5G>A		Likely Pathogenic	PS1_moderate (Another likely pathogenic variant in same position: c.7976+5G>T (PMID: 29969168), which was predicted to cause exon 17 skipping)<br>PP3 (SpliceAI DL: 0.9 and AL: 0.78. Possibly exon skipping. RAW score donor changes from 0.97 to 0.03 and acceptor changes from 0.97 to 0.19.)<br>PM2_supporting (Only 1 in GnomAD)		1	0	0	0	1	0
BRCA2	missense_variant	NM_000059.4:c.7987G>A	NP_000050.3:p.Glu2663Lys	Likely Pathogenic	PS3 (Functional study showing a damanging effect (PMID: 33609447))<br>PP3_supporting (Located inside functional domain and BayesDel=0.32)<br>PM2_supporting (Only 1 in GnomAD)		1	0	1	0	0	0
BRCA2	missense_variant	NM_000059.4:c.7988A>T	NP_000050.3:p.Glu2663Val	Pathogenic	ENIGMA		5	0	3	0	0	0
BRCA2	frameshift_variant	NM_000059.4:c.8474delC	NP_000050.3:p.Ala2825Aspfs*38	Pathogenic	ENIGMA		1	0	1	0	0	0
BRCA2	stop_gained	NM_000059.4:c.8536G>T	NP_000050.3:p.Glu2846Ter	Pathogenic	ENIGMA		1	0	1	0	0	0
BRCA2	frameshift_variant	NM_000059.4:c.8575delC	NP_000050.3:p.Gln2859Lysfs*4	Pathogenic	ENIGMA		1	0	0	0	1	0
BRCA2	frameshift_variant	NM_000059.4:c.8730delT	NP_000050.3:p.Asn2910Lysfs*17	Pathogenic	ENIGMA		1	0	0	0	0	0
BRCA2	splice_acceptor_variant	NM_000059.4:c.8755-1G>C		Pathogenic	PVS1 (Splice acceptor variant predicted to lead to PTC and NMD)<br>PS1 (Variant c.8755-1G>A, in the same position, is classified C5 by ENIGMA)<br>PM2_supporting (Absent from GnomAD)		1	0	0	0	0	0
BRCA2	frameshift_variant	NM_000059.4:c.9016_9017delTA	NP_000050.3:p.Tyr3006Glnfs*11	Pathogenic	ENIGMA		2	0	1	0	0	0
BRCA2	frameshift_variant	NM_000059.4:c.9017dupA	NP_000050.3:p.Tyr3006Ter	Pathogenic	ENIGMA		1	0	1	0	0	0
BRCA2	frameshift_variant	NM_000059.4:c.9027delT	NP_000050.3:p.His3010Ilefs*18	Pathogenic	ENIGMA		1	0	1	0	0	0
BRCA2	frameshift_variant	NM_000059.4:c.9097dupA	NP_000050.3:p.Thr3033Asnfs*11	Pathogenic	ENIGMA		1	0	1	0	0	0
BRCA2	synonymous_variant	NM_000059.4:c.9117G>A	NP_000050.3:p.Pro3039=	Pathogenic	ENIGMA		1	0	0	0	0	0
BRCA2	missense_variant	NM_000059.4:c.9154C>T	NP_000050.3:p.Arg3052Trp	Pathogenic	ENIGMA		1	0	0	0	0	0
BRCA2	stop_gained	NM_000059.4:c.9382C>T	NP_000050.3:p.Arg3128Ter	Pathogenic	ENIGMA		1	0	1	0	0	0
BRCA2	frameshift_variant	NM_000059.4:c.9699_9702delTATG	NP_000050.3:p.Cys3233Trpfs*15	Pathogenic	ENIGMA		1	0	0	0	0	0
BRIP1	frameshift_variant	NM_032043.3:c.1683_1684delGA	NP_114432.2:p.Gln561Hisfs*2	Pathogenic	PVS1 (Frameshift variant predicted to undergo NMD)<br>PM2_supporting (Absent from GnomAD)<br>PP5 (Laboratories reported it pathogenic in ClinVar)	SpliceAI AG: 0.22. Possibly partiel exon deletion. RAW score alternative acceptor 0.23 and natural acceptor 0.98	1	0	0	0	1	0
BRIP1	splice_donor_variant	NM_032043.3:c.2097+1G>C		Likely Pathogenic	ccDK		13	0	8	0	7	0
BRIP1	stop_gained	NM_032043.3:c.2377C>T	NP_114432.2:p.Gln793Ter	Pathogenic	PVS1 (Nonsense variant predicted to undergo NMD)<br>PM2_supporting (Only 2 in GnomAD)<br>PP5 (Laboratories reported it pathogenic at ClinVar)	SpliceAI AL: 0.53 and DL: 0.52. Possibly exon skipping. RAW score donor changes from 0.99 to 0.48 and acceptor changes from 0.96 to 0.43	1	0	1	0	0	0
BRIP1	stop_gained	NM_032043.3:c.2392C>T	NP_114432.2:p.Arg798Ter	Likely Pathogenic	PVS1 (Nonsense variant predicted to undergo NMD)<br>PP5 (Laboratories reported it as pathogenic at ClinVar)		2	0	1	0	0	0
BRIP1	stop_gained	NM_032043.3:c.2400C>G	NP_114432.2:p.Tyr800Ter	Likely Pathogenic	ccDK		6	0	3	0	3	0
BRIP1	missense_variant	NM_032043.3:c.2543G>A	NP_114432.2:p.Arg848His	Likely Pathogenic	PP3 (Revel=0.932 and BayesDel 0.54)<br>PP1 (Co-segegation in three family members (PMID: 27074266))<br>PS3 (Functional study showing decrease in helicase activity (PMID: 33028645))		1	0	1	0	1	0
CDH1	frameshift_variant	NM_004360.5:c.1009_1010delAG	NP_004351.1:p.Ser337Phefs*12	Pathogenic	PVS1 (Frameshift variant predicted to undergo NMD)<br>PM5_supporting (Frameshift variant predicted to undergo NMD)<br>PM2_supporting (Absent from GnomAD)	SpliceAI AG: 0.99 and AL: 1.00. Possibly partiel exon deletion. RAW score natural acceptor changes from 0.99 to 0 and alternative acceptor changes from 0 to 0.99	1	0	1	0	0	0

CDH1	splice_acceptor_variant	NM_004360.5:c.49-2A>C		Pathogenic	ccDK		1	0	1	0	0	0
CHEK2	frameshift_variant	NM_007194.4:c.1022delA	NP_009125.1:p.Asn341Thrfs*8	Pathogenic	PVS1 (Frameshift variant predicted to undergo NMD)<br>PM2_supporting (Absent from GnomAD)<br>PP5 (Invitae reported it pathogenic in ClinVar)		1	0	1	0	0	0
CHEK2	frameshift_variant	NM_007194.4:c.1100delC	NP_009125.1:p.Thr367Metfs*15	Pathogenic	ccDK		99	1	76	1	10	0
CHEK2	frameshift_variant	NM_007194.4:c.1489delG	NP_009125.1:p.Asp497Ilefs*16	Pathogenic	PVS1 (Frameshift variant predicted to undergo NMD)<br>PP5 (Laboratories reported it pathogenic in ClinVar)<br>PM2_supporting (Absent from GnomAD v.2.1.)		1	0	1	0	0	0
CHEK2	frameshift_variant	NM_007194.4:c.255delT	NP_009125.1:p.Glu86Argfs*24	Likely Pathogenic	PVS1 (Frameshift variant predicted to undergo NMD)<br>PM2_supporting (Absent from GnomAD)		1	0	0	0	0	0
CHEK2	missense_variant	NM_007194.4:c.349A>G	NP_009125.1:p.Arg117Gly	Likely Pathogenic	PP3 (Revel=0.93 and BayesDel 0.54)<br>PS3 (Functional studies showing damaging effect (PMID: 22419737 and PMID: 30851065))<br>PP5 (Laboratories reported it pathogenic in ClinVar)	Literature search in ClinVar and HGMD showed OR=2.26 (PMID: 27595995); OR=2.936 (PMID: 33471991); OR=2.83 (PMID: 37449874) and therefore remained likely pathogenic	3	0	3	0	0	0
CHEK2	frameshift_variant	NM_007194.4:c.432delT	NP_009125.1:p.Arg145Glyfs*16	Pathogenic	PVS1 (Frameshift variant predicted to undergo NMD)<br>PM2_supporting (Only 1 in GnomAD)<br>PP5 (Laboratories reported it pathogenic in ClinVar)		1	0	0	0	0	0
CHEK2	splice_donor_variant	NM_007194.4:c.444+1G>A		Likely Pathogenic	PVS1 (Possibly cryptic splice site leading to intron retention, that disrupts reading frame and predicted to undergo NMD)<br>PP1 (Two affected family members with the variant, furthermore 1 not tested but affected (PMID: 12533788)	SpliceAI DL:1.00 and DG: 0.94. Possibly intron retention. RAW score natural donor site changes from 0.99 to 0 and alternative donor site changes from 0.05 to 0.99	10	0	8	0	0	0
CHEK2	frameshift_variant	NM_007194.4:c.466dupT	NP_009125.1:p.Tyr156Leufs*30	Likely Pathogenic	PVS1 (Frameshift variant predicted to undergo NMD)<br>PM2_supporting (Absent from GnomAD)		1	0	1	0	0	0
CHEK2	frameshift_variant	NM_007194.4:c.655delG	NP_009125.1:p.Glu219Asnfs*16	Pathogenic	PVS1 (Frameshift variant predicted to undergo NMD)<br>PP5 (Laboratories reported it pathogenic in ClinVar)<br>PM2_supporting (Absent from GnomAD v.2.1)		4	0	2	0	0	0
CHEK2	frameshift_variant	NM_007194.4:c.935_936delAA	NP_009125.1:p.Lys312Serfs*5	Likely Pathogenic	PVS1 (Frameshift variant predicted to undergo NMD)<br>PM2_supporting (Absent from GnomAD)		1	0	1	0	0	0
PALB2	frameshift_variant	NM_024675.4:c.1019delA	NP_078951.2:p.Asn340Metfs*6	Pathogenic	ccDK		1	0	1	0	0	0
PALB2	stop_gained	NM_024675.4:c.1451T>A	NP_078951.2:p.Leu484Ter	Pathogenic	ccDK		1	0	1	0	0	0
PALB2	frameshift_variant	NM_024675.4:c.1454_1457delCTAA	NP_078951.2:p.Thr485Lysfs*75	Pathogenic	ccDK		2	0	1	0	0	0
PALB2	frameshift_variant	NM_024675.4:c.1538delC	NP_078951.2:p.Thr513Lysfs*48	Pathogenic	ccDK		1	0	0	0	0	0
PALB2	frameshift_variant	NM_024675.4:c.1660_1666delGAAAAAT	NP_078951.2:p.Glu554Tyrfs*5	Pathogenic	PVS1 (Frameshift variant upstream c.3301)<br>PM2_supporting (Absent from GnomAD)<br>PM5_supporting (Frameshift variant with PTC upstream p.Tyr1183)		1	0	0	0	0	0
PALB2	frameshift_variant	NM_024675.4:c.172_175delTTGT	NP_078951.2:p.Gln60Argfs*7	Pathogenic	PVS1 (Frameshift variant predicted to undergo NMD)<br>PS4 (Case-kontrol study (PMID: 34006922) showing odds ratio=5.02 and p-value=0.007)<br>PM5_supporting (PTC upstream p.Tyr1183)		2	0	1	0	0	0
PALB2	stop_gained	NM_024675.4:c.1919C>A	NP_078951.2:p.Ser640Ter	Pathogenic	PVS1 (Nonsense variant upstream c.3301)<br>PM2_supporting (Only 1 in GnomAD)<br>PM5_supporting (Nonsense variant with PTC upstream p.Tyr1183)		1	0	1	0	0	0
PALB2	splice_donor_variant	NM_024675.4:c.2586+1G>A		Likely Pathogenic	ccDK		2	0	2	0	0	0
PALB2	stop_gained	NM_024675.4:c.2718G>A	NP_078951.2:p.Trp906Ter	Pathogenic	ccDK		2	0	1	0	0	0
PALB2	splice_donor_variant	NM_024675.4:c.2748+1G>T		Likely Pathogenic	ccDK		1	0	1	0	0	0
PALB2	splice_acceptor_variant	NM_024675.4:c.2835-6_2841delGCTTAGGGCATTG	NP_078951.2:p.?	Likely Pathogenic	PVS1 (Exon skipping that disrupts the reading frame and predicted to undergo NMD)<br>PM2_supporting (Absent from GnomAD)	SpliceAI AL: 0.99 and AG: 0.87. Possibly partiel exon deletion. RAW score natural acceptor changes from 0.99 to 0 and alterantive acceptor changes from 0.02 to 0.89	1	0	1	0	0	0
PALB2	initiator_codon_variant	NM_024675.4:c.2T>G	NP_078951.2:p.Met1Arg	Likely Pathogenic	PVS1 (Initiator codon variant)<br>PM2_supporting (Absent from GnomAD)		1	0	1	0	0	0
PALB2	splice_region_variant	NM_024675.4:c.3113+5G>A		Likely Pathogenic	PVS1_strong (Cloneseq splicing analysis shows up-regulation of a cryptic donor site (PMID: 30890586))<br>PS1_moderate (In the same position c.3113+5G>C was classified as likely pathogenic or pathogenic in ClinVar)<br>PM2_supporting (Absent from GnomAD)	SpliceAI AL:0.43. DG: 0.24 and DL:0.36. Possibly exon skipping. RAW score natural acceptor changes from 0.99 to 0.56 and natural donor changes from 0.99 to 0.64	1	1	1	1	0	0
PALB2	stop_gained	NM_024675.4:c.3256C>T	NP_078951.2:p.Arg1086Ter	Likely Pathogenic	ccDK		4	0	2	0	0	0
PALB2	frameshift_variant	NM_024675.4:c.3456dupA	NP_078951.2:p.Pro1153Thrfs*4	Pathogenic	ccDK		1	0	0	0	0	0
PALB2	frameshift_variant	NM_024675.4:c.3507_3508delTC	NP_078951.2:p.His1170Phefs*19	Pathogenic	PVS1 (Frameshift variant not predicted to undergo NMD, but introduces a PTC upstream p.His1184)<br>PM2_supporting (GnomAD 0.00000304539)<br>PM5_supporting (PTC upstream Tyr1183)		2	0	1	0	0	0
PALB2	frameshift_variant	NM_024675.4:c.509_510delGA	NP_078951.2:p.Arg170Ilefs*14	Likely Pathogenic	ccDK		2	0	2	0	0	0
PALB2	frameshift_variant	NM_024675.4:c.651_654delTGAA	NP_078951.2:p.Asn217Lysfs*5	Pathogenic	ccDK		1	0	1	0	0	0
PALB2	stop_gained	NM_024675.4:c.712A>T	NP_078951.2:p.Arg238Ter	Pathogenic	PVS1 (Nonsense variant predicted to undergo NMD)<br>PM5_supporting (Stop codon upstream p.Tyr1183)<br>PM2_supporting (GnomAD frequency 0.00000342)		1	0	0	0	1	0
PALB2	frameshift_variant	NM_024675.4:c.760_761delTC	NP_078951.2:p.Ser254Argfs*2	Pathogenic	ccDK		1	0	1	0	0	0
PTEN	missense_variant	NM_000314.8:c.314G>A	NP_000305.3:p.Cys105Tyr	Likely Pathogenic	PM2_supporting (Absent from GnomAD)<br>PM6 (ClinGen expert panel reports in ClinVar that the variant is assumed de novo in a patient with disease and no family history, but without confirmation of paternity and maternity (internal laboratory contributor ClinVar Organization ID 26957))<br>PP2 (Missense)<br>PP3 (Revel=0.9)<br>PS4_supporting(ClinGen expert panel reports in ClinVar that there are proband(s) with phenotype specificity score of 1-1.5. (internal laboratory contributor ClinVar Organization ID 26957))		1	0	1	0	0	0
PTEN	frameshift_variant	NM_000314.8:c.40dupA	NP_000305.3:p.Arg14Lysfs*30	Pathogenic	PVS1 (Nonsense variant predicted to undergo NMD upstream c.1121)<br>PM2_supporting (Absent from GnomAD)<br>PM6 (De novo variant without confirmed parental testing (PMID: 10777358))		1	0	1	0	0	0
PTEN	frameshift_variant	NM_000314.8:c.455_461delTAGATTT	NP_000305.3:p.Leu152Profs*5	Likely Pathogenic	PVS1 (Frameshift variant predicted to undergo NMD)<br>PM2_supporting (Absent from GnomAD)		1	0	0	0	1	0
PTEN	missense_variant	NM_000314.8:c.518G>A	NP_000305.3:p.Arg173His	Likely Pathogenic	PS2_strong (Variant found in 4 year old boy and confirmed not present in his parents (PMID: 37307869))<br>PS3_moderate (Functional study showing a phosphatase activity on 1.34 (PMID: 29706350))<br>PM2_supporting (Only 3 in GnomAD)<br>PP3 (Revel=0.949)<br>PP2 (Missense variant)		1	0	1	0	0	0
PTEN	splice_donor_variant	NM_000314.8:c.801+1G>T		Likely Pathogenic	PVS1 (Exon skipping that disrupts reading frame and undergo NMD)<br>PM2_supporting (Absent from GnomAD)	SpliceAI DL: 1.0. AL: 0.53 and DG: 0.23. Possibly exon skipping or intron retention. RAW score natural acceptor changes from 0.98 to 0.45, natural donor changes from 0.99 to 0.03 and alternative donor changes from 0 to 0.28	1	0	0	0	0	0
RAD51C	frameshift_variant	NM_058216.3:c.498delT	NP_478123.1:p.Asp167Ilefs*4	Pathogenic	ccDK		3	0	2	0	1	0
RAD51C	frameshift_variant	NM_058216.3:c.774delT	NP_478123.1:p.Thr259Leufs*4	Pathogenic	PVS1 (Frameshift variant predicted to undergo NMD)<br>PP5 (Laboratories reported it pathogenic in ClinVar)<br>PM2_supporting (Absent from GnomAD v.2.1.)		1	0	0	0	0	0
RAD51C	splice_region_variant	NM_058216.3:c.904+5G>T		Likely Pathogenic	PP1 (Family with 3 carriers, who are affected by either BC or OC (PMID: 20400964))<br>PP3 (SpliceAI AL: 0.95 and DL: 0.94. Possibly exon skipping. RAW score donor site changes from 0.98 to 0.04 and acceptor site changes from 0.97 to 0.02.)<br>PS3 (Functional study showing the variant effects splicing (PMID: 20400964))		1	0	1	0	0	0
RAD51D	frameshift_variant	NM_002878.4:c.564_568delTGTGGinsA	NP_002869.3:p.Val189fs	Pathogenic	ccDK		3	0	1	0	2	0

RAD51D	missense_variant	NM_002878.4:c.620C>T	NP_002869.3:p.Ser207Leu	Likely Pathogenic	PS3 (Research group conducted multiple functional studies and found impaired homologous recombination, XRCC2 binding, and RAD51 foci formation (PMID: 28646019))<br>PP1 (The variant was found to co-segregate in 1 family with ovarian cancer og two families with breast cancer (PMID: 28646019))<br>PP5 (Labs reported it pathogenic in ClinVar)<br>PP3 (Revel=0.819 and BayesDel 0.5043)		1	0	1	0	0	0
RAD51D	stop_gained	NM_002878.4:c.694C>T	NP_002869.3:p.Arg232Ter	Pathogenic	PVS1 (Nonsense variant predicted to undergo NMD)<br>PS4 (Case-control study showing OR=16.07, 95%Cl (5.12;50.46) and p-value=0.0001 (PMID: 32359370))<br>PP5 (Laboratories reported it pathogenic in ClinVar)		1	0	1	0	0	0
TP53	frameshift_variant	NM_000546.6:c.150delT	NP_000537.3:p.Ile50Metfs*73	Likely Pathogenic	PVS1 (Frameshift variant predicted to undergo NMD)<br>PM2_supporting (Absent in GnomAD)		1	0	1	0	0	0
TP53	stop_gained	NM_000546.6:c.309C>G	NP_000537.3:p.Tyr103Ter	Pathogenic	ccDK		1	0	1	0	1	0
TP53	splice_acceptor_variant	NM_000546.6:c.376-1G>A		Pathogenic	PVS1_strong (Cryptic SS, but preserves reading frame)<br>PM2_supporting (Only 1 in GnomAD)<br>PM6_strong (Two patients with rhabdomyosarcoma at age 3 with no parental corfirmation (PMID: 27501770 and PMID: 24382691))	SpliceAI AL:1.00 and AG: 0.98. Possibly partiel exon deletion. RAW score natural acceptor changes from 0.99 to 0 and alternative acceptor changes from 0 to 0.98	1	0	0	0	1	0
TP53	missense_variant	NM_000546.6:c.455C>T	NP_000537.3:p.Pro152Leu	Pathogenic	PS3 (Kato et al.: Non-Functional and Giacomelli et al.: DNE_LOF)<br>PP3_moderat (BayesDel score 0.5581 and aGVGD C65)<br>PM1 (Cancerhotspot found: 38)<br>PS4 (ClinGen expert panel reports in ClinVar that this variant has been reported in at least 8 probands meeting Chompret criteria (PMID: 25584008, 10486318, 17308077, 15654279, 26014290))<br>PP1 (ClinGen expert panel reports in ClinVar that this variant was found to co-segregate with disease in multiple affected family members, with at least 3 meioses observed (PMID: 10486318))		1	0	1	0	0	0
TP53	missense_variant	NM_000546.6:c.542G>A	NP_000537.3:p.Arg181His	Likely Pathogenic	PM1 (Cancerhotspot found: 26)<br>PP3 (aGVGD C25 and BayesDel 0.2584)<br>PP1 (Family with 3 carriers affected (PMID: 30653764))<br>PS4_moderate (4 families meeting chompret criteria (PMID: 1591732, PMID: 30653764, PMID: 21059199 and PMID: 23175693))		4	0	4	0	0	0
TP53	missense_variant	NM_000546.6:c.645T>G	NP_000537.3:p.Ser215Arg	Likely Pathogenic	ccDK		1	0	1	0	0	0
TP53	splice_donor_variant	NM_000546.6:c.672+2T>G		Likely Pathogenic	ccDK		1	0	0	0	0	0
TP53	splice_acceptor_variant	NM_000546.6:c.673-1G>A		Likely Pathogenic	PVS1 (Cryptic splice site disrupts reading frame and undergo NMD)<br>PM2_supporting (Absent from GnomAD)	SpliceAI AL:1.00 and AG: 0.90. Possibly partiel exon deletion. RAW score natural acceptor changes from 0.99 to 0 and alternative acceptor changes from 0 to 0.90	1	0	0	0	0	0
TP53	missense_variant	NM_000546.6:c.713G>A	NP_000537.3:p.Cys238Tyr	Likely Pathogenic	ccDK		1	1	0	0	1	1
TP53	missense_variant	NM_000546.6:c.817C>T	NP_000537.3:p.Arg273Cys	Likely Pathogenic	ccDK		1	0	0	0	1	0
TP53	frameshift_variant	NM_000546.6:c.823delT	NP_000537.3:p.Cys275Valfs*70	Pathogenic	PVS1 (Frameshift variant predicted to undergo NMD)<br>PM2_supporting (Absent from GnomAD)<br>PM1 (Cancerhotspot located: 70)		1	0	0	0	0	0
TP53	missense_variant	NM_000546.6:c.838A>G	NP_000537.3:p.Arg280Gly	Likely Pathogenic	ccDK		1	0	0	0	0	0
TP53	missense_variant	NM_000546.6:c.839G>A	NP_000537.3:p.Arg280Lys	Likely Pathogenic	PM2_supporting (Absent from GnomAD)<br>PP3 (BayesDel=0.5921 and aGVGD klasse: C25)<br>PM1 (Cancerhotspot located: 109)<br>PM6(A case of breast cancer diagnosed before age of 30 (PMID: 34266904) and a case of gliblastoma and brother with Burkitt lymfoma (PMID: 10589545))		1	0	0	0	0	0
TP53	missense_variant	NM_000546.6:c.844C>T	NP_000537.3:p.Arg282Trp	Pathogenic	ccDK		1	1	1	1	0	0
TP53	stop_gained	NM_000546.6:c.853G>T	NP_000537.3:p.Glu285Ter	Likely Pathogenic	ccDK		1	0	0	0	0	0
TP53	stop_gained	NM_000546.6:c.916C>T	NP_000537.3:p.Arg306Ter	Likely Pathogenic	ccDK		1	0	1	0	0	0

Table S5: Classification and frequencies of all the identified variants with unknown significance (VUS) found in our study populaiton of 5,923 patients with a clinical suspicion of predispostion to heredirary breast- and/or ovarian cancer.
ACMG: American College of Medical Genetics
AG: Acceptor gain
AL: Acceptor loss
AT/A-T: Ataxia-Telangiectasia
BC: Breast cancer
BRCT: BRCA1 C Terminus
ccDK: Cancer (variant) classification DK (The variants classified by this group were not assigned ACMG criteria)
DG: Donor gain
DGC: Differentiated glioblastoma cells
DL: Donor loss
DNE: Dominante-negative effect
ENIGMA: Evidence-based Network for the Interpretation of Germline Mutant Alleles (The variants classified by this group were not assigned ACMG criteria)
GnomAD: Genome Aggregation Database
HDGC: Hereditary diffuse gastric cancer
HDR: Homology-directed repair
HGMD: The Human Gene Mutation Database
HR: Homologous recombination
HRD:Homologous recombination deficiency
LBC: Leukaemia and blood cancer
LOF: Loss of function
mESC: Mouse embryonic stem cell
NMD: Nonsense-mediated decay PTC: Premature termination codons
OC: Ovarian cancer
SRC: Signet ring cell
SS: Splice site
SSF:SpliceSiteFinder
XRCC2: X-ray repair cross complementing 2

Gene	Sequence Ontology	HGVS c.	HGVS p.	Classification	Assigned ACMG criteria	Comments	#Samples_Total (N=5,923)	#Hom samples_Total (N=5,923)	#Samples_BC (N=3,706)	#Hom samples_BC (N=3,706)	#Samples_OC (N=890)	#Hom samples_OC (N=890)
ATM	missense_variant	NM_000051.4:c.1021G>A	NP_000042.3:p.Val341Ile	Unknown Significance	BP4 (Revel=0.08)		1	0	0	0	0	0
ATM	missense_variant	NM_000051.4:c.115A>G	NP_000042.3:p.Thr39Ala	Unknown Significance	ccDK		1	0	1	0	0	0
ATM	splice_region_variant	NM_000051.4:c.1236-8T>C		Unknown Significance	BP4 (SpliceAI=0.02)		2	0	2	0	0	0
ATM	missense_variant	NM_000051.4:c.1273G>T	NP_000042.3:p.Ala425Ser	Unknown Significance	BP4 (Revel=0.059)		1	0	1	0	0	0
ATM	missense_variant	NM_000051.4:c.131A>G	NP_000042.3:p.Asp44Gly	Unknown Significance	No criteria met		1	0	1	0	0	0
ATM	missense_variant	NM_000051.4:c.1516G>T	NP_000042.3:p.Gly506Cys	Unknown Significance	PM3_supporting (Proband with A-T in homozygote phase (PMID: 32172615))		1	0	1	0	0	0
ATM	missense_variant	NM_000051.4:c.1595G>A	NP_000042.3:p.Cys532Tyr	Unknown Significance	No criteria met		1	0	0	0	0	0
ATM	missense_variant	NM_000051.4:c.1600C>A	NP_000042.3:p.Pro534Thr	Unknown Significance	PM2_supporting (Absent from GnomAD)		1	0	1	0	0	0
ATM	missense_variant	NM_000051.4:c.1601C>T	NP_000042.3:p.Pro534Leu	Unknown Significance	PM2_supporting (Only 1 in GnomAD)		1	0	1	0	0	0
ATM	synonymous_variant	NM_000051.4:c.1638G>A	NP_000042.3:p.Leu546=	Unknown Significance	PM2_supporting (Absent from GnomAD)<br>PP3 (SpliceAI AL:0.24. Possibly exon skipping. RAW score natural acceptor changes from 0.41 to 0.16 and natural donor changes from 0.55 to 0.46)		1	0	0	0	0	0
ATM	missense_variant	NM_000051.4:c.1705A>C	NP_000042.3:p.Ser569Arg	Unknown Significance	PM2_supporting (Absent from GnomAD)<br>BP4 (Revel=0.103)		1	0	0	0	1	0
ATM	missense_variant	NM_000051.4:c.1709T>C	NP_000042.3:p.Phe570Ser	Unknown Significance	BS3_moderate (Functional study showing normal ATM protein level, normal kinase activity and the radiosensitivity was corrected (PMID: 18634022))<br>PM3_moderate (Two brothers with mild A-T had the variant in trans phase (PMID: 9887333))		4	0	3	0	0	0
ATM	missense_variant	NM_000051.4:c.1727T>C	NP_000042.3:p.Ile576Thr	Unknown Significance	No criteria met		2	0	1	0	1	0
ATM	synonymous_variant	NM_000051.4:c.1773T>C	NP_000042.3:p.Asn591=	Unknown Significance	PP3 (SpliceAI AG:0.20 and DG:0.25. Possibly partiel exon deletion. RAW score natural donor changes from 0.55 to 0.80, natural acceptor changes from 0.41 to 0.61 and alternative acceptor changes from 0.07 to 0.28)		1	0	1	0	0	0
ATM	intron_variant	NM_000051.4:c.1802+272T>G		Unknown Significance	No criteria met		1	0	1	0	0	0
ATM	intron_variant	NM_000051.4:c.1803-35G>C		Unknown Significance	BP4 (SpliceAI=0.00)		1	0	0	0	0	0
ATM	missense_variant	NM_000051.4:c.1814A>G	NP_000042.3:p.His605Arg	Unknown Significance	No criteria met		3	0	2	0	0	0
ATM	intron_variant	NM_000051.4:c.1899-123A>G		Unknown Significance	PP3 (SpliceAI AG: 0.74 and DG: 0.71. Possibly pseudo-exon. RAW score donor 0.71 and acceptor 0.74)		1	0	1	0	0	0
ATM	synonymous_variant	NM_000051.4:c.2040C>T	NP_000042.3:p.Phe680=	Unknown Significance	PP3 (SpliceAI AG:0.22. Possibly partiel exon deletion. RAW score alternative site changes from 0.25 to 0.46 and naturel splice site changes from 0.99 to 0.98)		2	0	2	0	0	0
ATM	missense_variant	NM_000051.4:c.2071G>C	NP_000042.3:p.Asp691His	Unknown Significance	BP4 (Revel=0.171)		3	0	2	0	1	0
ATM	missense_variant	NM_000051.4:c.2075G>A	NP_000042.3:p.Arg692His	Unknown Significance	PM2_supporting (GnomAD frequency 0.00000434)<br>BP4 (Revel=0.029)		1	0	1	0	0	0
ATM	intron_variant	NM_000051.4:c.2124+644C>T		Unknown Significance	No criteria met		1	0	1	0	0	0
ATM	splice_region_variant	NM_000051.4:c.2124+7T>A		Unknown Significance	BP4 (SpliceAI=0.00)<br>PM2_supporting (Only 1 in GnomAD)		1	0	1	0	1	0
ATM	missense_variant	NM_000051.4:c.2149C>T	NP_000042.3:p.Arg717Trp	Unknown Significance	No criteria met		1	0	1	0	1	0
ATM	missense_variant	NM_000051.4:c.214A>G	NP_000042.3:p.Thr72Ala	Unknown Significance	PM2_supporting (Absent from GnomAD)<br>BP4 (Revel=0.091)		1	0	1	0	0	0
ATM	missense_variant	NM_000051.4:c.2150G>A	NP_000042.3:p.Arg717Gln	Unknown Significance	BP4 (Revel=0.148)	SpliceAI AG:0.21. Possibly partiel exon deletion. RAW score alternative acceptor changes from 0 to 0.21 and natural acceptor changes from 0.99 to 0.93	1	0	1	0	0	0
ATM	missense_variant	NM_000051.4:c.221G>A	NP_000042.3:p.Cys74Tyr	Unknown Significance	PM2_supporting (GnomAD frequency 0.00000479)<br>BP4 (Revel=0.114)		1	0	1	0	0	0
ATM	missense_variant	NM_000051.4:c.2221T>C	NP_000042.3:p.Tyr741His	Unknown Significance	PM2_supporting (Absent from GnomAD)<br>BP4 (Revel=0.07)		2	0	1	0	0	0
ATM	missense_variant	NM_000051.4:c.2248A>G	NP_000042.3:p.Lys750Glu	Unknown Significance	No criteria met		2	0	2	0	0	0
ATM	splice_region_variant	NM_000051.4:c.2251-11A>G		Unknown Significance	BP4 (SpliceAI=0.00)		3	0	0	0	0	0
ATM	intron_variant	NM_000051.4:c.2251-63T>G		Unknown Significance	PP3 (SpliceAI AG: 0.26. Possibly intron retention or cryptic splice site. RAW score changed from 0.02 to 0.28)<br>PM2_supporting (Absent from GnomAD)		1	0	1	0	0	0
ATM	missense_variant	NM_000051.4:c.2266G>T	NP_000042.3:p.Ala756Ser	Unknown Significance	PM2_supporting (Absent from GnomAD)<br>BP4 (Revel=0.216)		1	0	1	0	0	0
ATM	missense_variant	NM_000051.4:c.2305G>A	NP_000042.3:p.Glu769Lys	Unknown Significance	PM2_supporting (Absent from GnomAD)		1	0	1	0	0	0
ATM	intron_variant	NM_000051.4:c.2466+1650G>A		Unknown Significance	No criteria met		1	0	1	0	0	0
ATM	splice_region_variant	NM_000051.4:c.2466+7A>G		Unknown Significance	PP3 (SpliceAI AL: 0.41 and DL: 0.27. Possibly exon skipping. RAW score acceptor changes from 0.99 to 0.75 and donor changes from 0.95 to 0.68)		1	0	1	0	0	0
ATM	missense_variant	NM_000051.4:c.2476A>C	NP_000042.3:p.Ile826Leu	Unknown Significance	BP4 (Revel=0.08)		2	0	1	0	0	0
ATM	missense_variant	NM_000051.4:c.2485C>T	NP_000042.3:p.Pro829Ser	Unknown Significance	PM2_supporting (Absent from GnomAD)<br>BP4 (Revel=0.101)		1	0	1	0	0	0
ATM	missense_variant	NM_000051.4:c.2494C>T	NP_000042.3:p.Arg832Cys	Unknown Significance	ccDK		1	0	1	0	0	0
ATM	missense_variant	NM_000051.4:c.2500G_	NP_000042.3:p.Glu834Gln	Unknown Significance	PM2_supporting (Absent from GnomAD)<br>BP4 (Revel=0.067)		1	0	1	0	0	0
ATM	missense_variant	NM_000051.4:c.2519A>T	NP_000042.3:p.Asp840Val	Unknown Significance	BP4 (Revel=0.208)		1	0	1	0	0	0
ATM	missense_variant	NM_000051.4:c.2564T>C	NP_000042.3:p.Met855Thr	Unknown Significance	PM2_supporting (Only 3 in GnomAD)<br>BP4 (Revel=0.042)		1	0	1	0	0	0
ATM	missense_variant	NM_000051.4:c.2602G>A	NP_000042.3:p.Asp868Asn	Unknown Significance	PM2_supporting (Absent from GnomAD)<br>BP4 (Revel=0.182)		1	0	0	0	0	0
ATM	missense_variant	NM_000051.4:c.2650C>G	NP_000042.3:p.Pro884Ala	Unknown Significance	PM2_supporting (Absent from GnomAD)		1	0	1	0	0	0
ATM	missense_variant	NM_000051.4:c.2693T>C	NP_000042.3:p.Leu898Ser	Unknown Significance	PP3 (Revel=0.746)<br>PM2_supporting (GnomAD frequency 0.00000342)		1	0	1	0	0	0
ATM	missense_variant	NM_000051.4:c.2716T>A	NP_000042.3:p.Leu906Met	Unknown Significance	BP4 (Revel=0.194)		1	0	0	0	0	0
ATM	missense_variant	NM_000051.4:c.2804C>T	NP_000042.3:p.Thr935Met	Unknown Significance	BP4 (Revel=0.113)		1	0	1	0	0	0
ATM	missense_variant	NM_000051.4:c.280A>G	NP_000042.3:p.Met94Val	Unknown Significance	BP4 (Revel=0.207)		1	0	0	0	1	0

ATM	intron_variant	NM_000051.4:c.2839-622T>C		Unknown Significance	PM2_supporting (Only 1 in GnomAD)		3	0	1	0	0	0
ATM	missense_variant	NM_000051.4:c.283C>A	NP_000042.3:p.Gln95Lys	Unknown Significance	BP4 (Revel=0.18)		1	0	0	0	0	0
ATM	missense_variant	NM_000051.4:c.2867G>A	NP_000042.3:p.Gly956Glu	Unknown Significance	BP4 (Revel=0.122)		1	0	1	0	0	0
ATM	intron_variant	NM_000051.4:c.2922-23C>A		Unknown Significance	BP4 (SpliceAI=0.00)		2	0	2	0	0	0
ATM	missense_variant	NM_000051.4:c.2924A>G	NP_000042.3:p.Asn975Ser	Unknown Significance	BP4 (Revel=0.049)	SpliceAI AG: 0.86. Possibly partiel exon deletion. RAW score alternative acceptor site changes from 0 to 0.86 and natural acceptor site changes from 0.99 to 0.98	2	0	2	0	0	0
ATM	missense_variant	NM_000051.4:c.2932T>C	NP_000042.3:p.Ser978Pro	Unknown Significance	ccDK		9	0	3	0	3	0
ATM	missense_variant	NM_000051.4:c.2942G>A	NP_000042.3:p.Arg981His	Unknown Significance	No criteria met		1	0	1	0	1	0
ATM	missense_variant	NM_000051.4:c.295A>G	NP_000042.3:p.Ser99Gly	Unknown Significance	No criteria met		1	0	1	0	0	0
ATM	missense_variant	NM_000051.4:c.2960G>A	NP_000042.3:p.Cys987Tyr	Unknown Significance	PM2_supporting (Only 1 in GnomAD)<br>PP3 (Revel=0.821)		1	0	0	0	0	0
ATM	missense_variant	NM_000051.4:c.2983C>G	NP_000042.3:p.Leu995Val	Unknown Significance	PM2_supporting (Only 2 in GnomAD)<br>BP4 (Revel=0.227)		1	0	0	0	1	0
ATM	splice_region_variant	NM_000051.4:c.-30-5dupT		Unknown Significance	BP4 (SpliceAI=0.04)		1	0	1	0	1	0
ATM	missense_variant	NM_000051.4:c.3061G>C	NP_000042.3:p.Val1021Leu	Unknown Significance	PM2_supporting (Only 1 in GnomAD)<br>BP4 (Revel=0.238)		4	0	1	0	0	0
ATM	missense_variant	NM_000051.4:c.3071C>T	NP_000042.3:p.Ala1024Val	Unknown Significance	No criteria met		2	0	2	0	0	0
ATM	splice_region_variant	NM_000051.4:c.3078-10T>G		Unknown Significance	PM2_supporting (Absent from GnomAD)<br>PP3 (SpliceAI AL: 0.70 and DL: 0.58. Possibly exon skipping. RAW score acceptor changes from 0.86 to 0.16 and donor changes from 0.93 to 0.36)		1	0	0	0	1	0
ATM	missense_variant	NM_000051.4:c.3121G>A	NP_000042.3:p.Ala1041Thr	Unknown Significance	PM2_supporting (Absent from GnomAD)		1	0	1	0	1	0
ATM	splice_region_variant	NM_000051.4:c.3154-4G>A		Unknown Significance	BP4 (SpliceAI=0.00)		2	0	2	0	0	0
ATM	splice_region_variant	NM_000051.4:c.3154-4G>T		Unknown Significance	PM2_supporting (GnomAD frequency 0.00000682)<br>BP4 (SpliceAI=0)		3	0	2	0	1	0
ATM	splice_region_variant	NM_000051.4:c.-31G>A		Unknown Significance	PP3 (SpliceAI DL: 0.69. Possibly exon skipping. RAW score changes from 0.72 to 0.03)<br>PM2_supporting (Absent from GnomAD)		1	0	1	0	0	0
ATM	missense_variant	NM_000051.4:c.3260T>C	NP_000042.3:p.Met1087Thr	Unknown Significance	PM2_supporting (GnomAD only 2)		1	0	0	0	0	0
ATM	missense_variant	NM_000051.4:c.3281A>G	NP_000042.3:p.Asn1094Ser	Unknown Significance	BP4 (Revel=0.076)	SpliceAI DG: 0.98. Possibly partiel exon deletion. RAW score alternative donor site changes from 0 to 0.98 and the natural donor site changes from 0.99 to 0.90	1	0	1	0	0	0
ATM	intron_variant	NM_000051.4:c.331+3118G>A		Unknown Significance	PP3 (SpliceAI AG: 0.26 and DG: 0.27. Possibly pseudo-exon. RAW score acceptor 0.31 and donor 0.40)<br>PM2_supporting (Absent from GnomAD)		1	0	0	0	1	0
ATM	missense_variant	NM_000051.4:c.3376A>G	NP_000042.3:p.Lys1126Glu	Unknown Significance	PM2_supporting (Absent from GnomAD)<br>BP4 (Revel=0.095)		2	0	1	0	0	0
ATM	missense_variant	NM_000051.4:c.3407A>G	NP_000042.3:p.His1136Arg	Unknown Significance	BP4 (Revel=0.035)		2	0	0	0	1	0
ATM	missense_variant	NM_000051.4:c.3496C>T	NP_000042.3:p.Pro1166Ser	Unknown Significance	PM2_supporting (Absent from GnomAD)		1	0	1	0	0	0
ATM	missense_variant	NM_000051.4:c.3519G>C	NP_000042.3:p.Leu1173Phe	Unknown Significance	No criteria met		6	0	2	0	3	0
ATM	missense_variant	NM_000051.4:c.3676G>C	NP_000042.3:p.Asp1226His	Unknown Significance	BP4 (Revel=0.08)		3	0	2	0	0	0
ATM	missense_variant	NM_000051.4:c.3743A>G	NP_000042.3:p.Tyr1248Cys	Unknown Significance	No criteria met		1	0	1	0	0	0
ATM	missense_variant	NM_000051.4:c.3806A>G	NP_000042.3:p.Lys1269Arg	Unknown Significance	BP4 (Revel=0.249)		1	0	1	0	0	0
ATM	missense_variant	NM_000051.4:c.3843T>G	NP_000042.3:p.Ser1281Arg	Unknown Significance	BP4 (Revel=0.079)		1	0	1	0	0	0
ATM	missense_variant	NM_000051.4:c.3964C>A	NP_000042.3:p.Leu1322Ile	Unknown Significance	No criteria met		1	0	0	0	0	0
ATM	intron_variant	NM_000051.4:c.3994-173T>A		Unknown Significance	PM2_supporting (Absent from GnomAD)		2	0	1	0	0	0
ATM	missense_variant	NM_000051.4:c.4060C>A	NP_000042.3:p.Pro1354Thr	Unknown Significance	BP4 (Revel=0.057)		2	0	2	0	0	0
ATM	missense_variant	NM_000051.4:c.4082A>G	NP_000042.3:p.Gln1361Arg	Unknown Significance	BP4 (Revel=0.137)		1	0	1	0	0	0
ATM	missense_variant	NM_000051.4:c.4148C>T	NP_000042.3:p.Ser1383Leu	Unknown Significance	PP3 (Revel=0.797)		1	0	1	0	0	0
ATM	missense_variant	NM_000051.4:c.4150C>A	NP_000042.3:p.His1384Asn	Unknown Significance	PM2_supporting (Absent from GnomAD)<br>BP4 (Revel=0.128)		1	0	1	0	0	0
ATM	intron_variant	NM_000051.4:c.4237-29A>G		Unknown Significance	PM2_supporting (Absent from GnomAD)<br>BP4 (SpliceAI=0.00)		5	0	3	0	1	0
ATM	missense_variant	NM_000051.4:c.4264A>T	NP_000042.3:p.Ile1422Leu	Unknown Significance	PM2_supporting (Absent from GnomAD)<br>BP4 (Revel=0.045)		1	0	0	0	1	0
ATM	missense_variant	NM_000051.4:c.4324T>C	NP_000042.3:p.Tyr1442His	Unknown Significance	No criteria met		21	0	14	0	3	0
ATM	missense_variant	NM_000051.4:c.4375G>A	NP_000042.3:p.Gly1459Arg	Unknown Significance	No criteria met		1	0	1	0	0	0
ATM	missense_variant	NM_000051.4:c.4385C>T	NP_000042.3:p.Ala1462Val	Unknown Significance	PM2_supporting (Absent from GnomAD)		1	0	1	0	0	0
ATM	missense_variant	NM_000051.4:c.4388T>G	NP_000042.3:p.Phe1463Cys	Unknown Significance	ccDK		1	0	1	0	0	0
ATM	missense_variant	NM_000051.4:c.4397G>A	NP_000042.3:p.Arg1466Gln	Unknown Significance	PM2_supporting (GnomAD frequency 0.00000479)		1	0	0	0	0	0
ATM	intron_variant	NM_000051.4:c.4437-30T>G		Unknown Significance	No criteria met		1	0	1	0	0	0
ATM	intron_variant	NM_000051.4:c.4437-578A>G		Unknown Significance	No criteria met		1	0	1	0	0	0
ATM	in-frame_deletion	NM_000051.4:c.4442_4444delCTT	NP_000042.3:p.Ser1481del	Unknown Significance	PM2_supporting (GnomAD 0.00000273695)<br>BP4 (SpliceAI=0.08)		1	0	0	0	0	0
ATM	missense_variant	NM_000051.4:c.4465C>T	NP_000042.3:p.Arg1489Cys	Unknown Significance	No criteria met		1	0	1	0	0	0
ATM	missense_variant	NM_000051.4:c.4547A>G	NP_000042.3:p.His1516Arg	Unknown Significance	PM2_supporting (Only 1 in GnomAD)<br>PP3 (Revel=0.815)		2	0	0	0	0	0
ATM	missense_variant	NM_000051.4:c.4561G>C	NP_000042.3:p.Val1521Leu	Unknown Significance	No criteria met		1	0	1	0	0	0
ATM	missense_variant	NM_000051.4:c.4588G>C	NP_000042.3:p.Glu1530Gln	Unknown Significance	PM2_supporting (Absent from GnomAD)<br>BP4 (Revel=0.045)		1	0	1	0	0	0
ATM	splice_region_variant	NM_000051.4:c.4612-8A>T		Unknown Significance	PM2_supporting (Absent from GnomAD)<br>BP4 (SpliceAI=0)		1	0	0	0	1	0
ATM	missense_variant	NM_000051.4:c.4639A>G	NP_000042.3:p.Ile1547Val	Unknown Significance	BP4 (Revel=0.175)		2	0	2	0	0	0
ATM	missense_variant	NM_000051.4:c.4662C>A	NP_000042.3:p.Asn1554Lys	Unknown Significance	BP4 (Revel=0.195)		1	0	1	0	0	0
ATM	missense_variant	NM_000051.4:c.4724G>A	NP_000042.3:p.Arg1575His	Unknown Significance	BS3_supporting (Functional study showing ATM polymorphism (PMID: 19431188))		1	0	0	0	0	0
ATM	missense_variant	NM_000051.4:c.4768C>T	NP_000042.3:p.Leu1590Phe	Unknown Significance	No criteria met		1	0	0	0	1	0
ATM	intron_variant	NM_000051.4:c.4777-60A>T		Unknown Significance	PP3 (SpliceAI AG: 0.42. Possibly intron retention or cryptic splice site. RAW score alternative acceptor 0.42 and naturel acceptor changes from 0.94 to 0.90)		2	0	2	0	0	0
ATM	missense_variant	NM_000051.4:c.4846G>T	NP_000042.3:p.Asp1616Tyr	Unknown Significance	PM2_supporting (Absent in GnomAD)<br>BP4 (Revel=0.235)		1	0	1	0	0	0
ATM	splice_region_variant	NM_000051.4:c.496+4T>C		Unknown Significance	BP4 (SpliceAI=0.00)		1	0	1	0	0	0
ATM	intron_variant	NM_000051.4:c.497-22A>G		Unknown Significance	No criteria met		1	0	1	0	0	0
ATM	missense_variant	NM_000051.4:c.4972G>T	NP_000042.3:p.Ala1658Ser	Unknown Significance	PM2_supporting (Absent from GnomAD)<br>BP4 (Revel=0.12)		1	0	1	0	0	0
ATM	splice_region_variant	NM_000051.4:c.497-3A>T		Unknown Significance	PM2_supporting (Only 2 in GnomAD)<br>BP4 (SpliceAI=0)		1	0	0	0	1	0
ATM	intron_variant	NM_000051.4:c.5006-1154C>G		Unknown Significance	No criteria met		3	0	2	0	1	0
ATM	intron_variant	NM_000051.4:c.5006-253C>G		Unknown Significance	PP3 (SpliceAI DG: 0.42 and AG: 0.20. Possibly pseudo-exon. RAW score donor 0.42 and acceptor 0.25)		11	0	7	0	1	0
ATM	missense_variant	NM_000051.4:c.5089A>G	NP_000042.3:p.Thr1697Ala	Unknown Significance	BP4 (Revel=0.039)		1	0	0	0	0	0
ATM	missense_variant	NM_000051.4:c.5262G>T	NP_000042.3:p.Lys1754Asn	Unknown Significance	No criteria met		2	0	1	0	0	0
ATM	missense_variant	NM_000051.4:c.5278A>G	NP_000042.3:p.Met1760Val	Unknown Significance	ccDK		2	0	1	0	0	0
ATM	splice_region_variant	NM_000051.4:c.5319+3C>A		Unknown Significance	PM2_supporting (GnomAD frequency 0.00000385)<br>BP4 (SpliceAI=0)		1	0	1	0	0	0
ATM	in-frame_deletion	NM_000051.4:c.5417_5422delTAAAGA	NP_000042.3:p.Ile1806_Lys1807del	Unknown Significance	PM2_supporting (Absent from GnomAD)		3	0	3	0	0	0
ATM	intron_variant	NM_000051.4:c.5496+24A>G		Unknown Significance	PP3 (SpliceAI DG: 0.24. Possibly intron retention or cryptic splice site. RAW score alternative donor site 0.24 and alternative donor site changes from 0.97 to 0.96)		1	0	1	0	0	0
ATM	missense_variant	NM_000051.4:c.550A>G	NP_000042.3:p.Arg184Gly	Unknown Significance	BP4 (Revel=0.201)		1	0	1	0	0	0
ATM	missense_variant	NM_000051.4:c.5511T>A	NP_000042.3:p.Phe1837Leu	Unknown Significance	PM2_supporting (Absent from GnomAD)<br>BP4 (Revel=0.156)		1	0	0	0	0	0
ATM	missense_variant	NM_000051.4:c.5590A>G	NP_000042.3:p.Thr1864Ala	Unknown Significance	PM2_supporting (Absent from GnomAD)<br>BP4 (Revel=0.113)		1	0	1	0	0	0
ATM	missense_variant	NM_000051.4:c.5618G>A	NP_000042.3:p.Cys1873Tyr	Unknown Significance	No criteria met		1	0	1	0	0	0
ATM	missense_variant	NM_000051.4:c.5645G>A	NP_000042.3:p.Arg1882Gln	Unknown Significance	No criteria met		3	0	1	0	0	0

ATM	missense_variant	NM_000051.4:c.5740G>C	NP_000042.3:p.Asp1914His	Unknown Significance	PM2_supporting (Absent from GnomAD)		2	0	2	0	0	0
ATM	splice_region_variant	NM_000051.4:c.5762+6G>A		Unknown Significance	BP4 (SpliceAI=0.00)		3	0	1	0	1	0
ATM	missense_variant	NM_000051.4:c.584C>T	NP_000042.3:p.Thr195Ile	Unknown Significance	BP4 (Revel=0.086)<br>PM2_supporting (GnomAD only 2)		1	0	1	0	0	0
ATM	missense_variant	NM_000051.4:c.5870A>G	NP_000042.3:p.Tyr1957Cys	Unknown Significance	PM2_supporting (GnomAD frequency 0.00000205)		1	0	1	0	0	0
ATM	missense_variant	NM_000051.4:c.5890A>G	NP_000042.3:p.Lys1964Glu	Unknown Significance	BP4 (Revel=0.207)		3	0	3	0	0	0
ATM	intron_variant	NM_000051.4:c.5919-101C>T		Unknown Significance	No criteria met		4	0	1	0	0	0
ATM	intron_variant	NM_000051.4:c.5919-84T>C		Unknown Significance	PP3 (SpliceAI AG: 0.26. Intron retention or cryptic splice site. RAW score changes from 0.11 to 0.37)		2	0	1	0	0	0
ATM	intron_variant	NM_000051.4:c.6006+1197delT		Unknown Significance	No criteria met		12	0	10	0	0	0
ATM	intron_variant	NM_000051.4:c.6007-1448G>C		Unknown Significance	PM2_supporting (Absent from GnomAD)		3	0	3	0	0	0
ATM	missense_variant	NM_000051.4:c.6025T>C	NP_000042.3:p.Tyr2009His	Unknown Significance	No criteria met		1	0	0	0	0	0
ATM	missense_variant	NM_000051.4:c.610G>A	NP_000042.3:p.Gly204Arg	Unknown Significance	BP4 (Revel=0.109)		2	0	1	0	1	0
ATM	missense_variant	NM_000051.4:c.6490G>C	NP_000042.3:p.Glu2164Gln	Unknown Significance	PM2_supporting (Absent from GnomAD)		1	0	1	0	0	0
ATM	missense_variant	NM_000051.4:c.654G>T	NP_000042.3:p.Gln218His	Unknown Significance	PM2_supporting (Absent from GnomAD)<br>BP4 (Revel=0.073)		1	0	1	0	0	0
ATM	missense_variant	NM_000051.4:c.6554T>C	NP_000042.3:p.Ile2185Thr	Unknown Significance	No criteria met		3	0	2	0	0	0
ATM	splice_region_variant	NM_000051.4:c.6572+4T>C		Unknown Significance	BP4 (SpliceAI=0)		1	0	1	0	0	0
ATM	intron_variant	NM_000051.4:c.663-194A>G		Unknown Significance	PM2_supporting (Absent from GnomAD)<br>PP3 (SpliceAI DG: 0.30 and AG: 0.18. Possibly pseudo-exon. RAW score donor 0.30 and acceptor 0.18)		1	0	1	0	0	0
ATM	intron_variant	NM_000051.4:c.663-38C>G		Unknown Significance	PP3 (SpliceAI AG: 0.25. Possibly intron retention or cryptic splice site. RAW score alternative acceptor 0.39 and naturel acceptor changes from 0.94 to 0.93)		1	0	0	0	0	0
ATM	missense_variant	NM_000051.4:c.6652A>C	NP_000042.3:p.Ser2218Arg	Unknown Significance	BP4 (Revel=0.096)	SpliceAI AG: 0.67 and AL: 0.69.Possibly partiel exon deletion. RAW score alternative splice site changes from 0.33 to 0.99 and naturel acceptor splice site changes from 0.92 to 0.34	2	0	2	0	0	0
ATM	missense_variant	NM_000051.4:c.670A>G	NP_000042.3:p.Lys224Glu	Unknown Significance	ccDK		4	0	1	0	1	0
ATM	in-frame-delins	NM_000051.4:c.6736_6755delinsCA	NP_000042.3:p.Cys2246_Thr2252delinsHis	Unknown Significance	PM2_supporting (Absent from GnomAD)<br>PM3_supporting (According to ClinVar the variant has been reported in the homozygous state in an individual with ataxia-telangiectasia (Castellvi-Bel 1999))		1	0	1	0	0	0
ATM	missense_variant	NM_000051.4:c.6820G>A	NP_000042.3:p.Ala2274Thr	Unknown Significance	BS3_moderate (Functional study showing normal kinase activity and correction of radiosensitivity (PMID: 11805335) and another functional functional study showing ATM polymorphism (PMID: 19431188))		1	0	0	0	0	0
ATM	missense_variant	NM_000051.4:c.6895T>C	NP_000042.3:p.Phe2299Leu	Unknown Significance	PM2_supporting (Absent from GnomAD)		2	0	1	0	0	0
ATM	missense_variant	NM_000051.4:c.68G>A	NP_000042.3:p.Arg23Gln	Unknown Significance	PM2_supporting (GnomAD frequency 0.0000089)		2	0	2	0	0	0
ATM	missense_variant	NM_000051.4:c.7075A>C	NP_000042.3:p.Thr2359Pro	Unknown Significance	PM2_supporting (Absent from GnomAD)<br>BP4 (Revel=0.188)		1	0	1	0	0	0
ATM	missense_variant	NM_000051.4:c.7223C>T	NP_000042.3:p.Ser2408Leu	Unknown Significance	PM2_supporting (GnomAD frequency 0.00000958)		1	0	1	0	0	0
ATM	missense_variant	NM_000051.4:c.7309T>C	NP_000042.3:p.Tyr2437His	Unknown Significance	PM2_supporting (Only 1 in GnomAD)<br>PP3 (Revel=0.824)		1	0	1	0	0	0
ATM	synonymous_variant	NM_000051.4:c.7314A>C	NP_000042.3:p.Thr2438=	Unknown Significance	PP3 (SpliceAI AL: 0.71 and AG: 0.54. Possibly cryptic site dispeares and a new possible cryptic splice site possibly created. RAW score alternative acceptor changes from 0.71 to 0.00 and the other acceptor changes from 0.14 to 0.68)		1	0	1	0	0	0
ATM	missense_variant	NM_000051.4:c.7316T>C	NP_000042.3:p.Val2439Ala	Unknown Significance	PM2_supporting (GnomAD frequency 0.00000372)		1	0	1	0	0	0
ATM	intron_variant	NM_000051.4:c.73-25A>G		Unknown Significance	BP4 (SpliceAI=0.00)		1	0	0	0	1	0
ATM	missense_variant	NM_000051.4:c.7429G>A	NP_000042.3:p.Gly2477Arg	Unknown Significance	PM2_supporting (Absent from GnomAD)		1	0	1	0	0	0
ATM	missense_variant	NM_000051.4:c.7475T>G	NP_000042.3:p.Leu2492Arg	Unknown Significance	ccDK		7	0	4	0	0	0
ATM	missense_variant	NM_000051.4:c.749G>A	NP_000042.3:p.Arg250Gln	Unknown Significance	BP4 (Revel=0.055)		2	0	2	0	0	0
ATM	missense_variant	NM_000051.4:c.7502A>G	NP_000042.3:p.Asn2501Ser	Unknown Significance	No criteria met		1	0	0	0	0	0
ATM	splice_region_variant	NM_000051.4:c.7516-9dupT		Unknown Significance	BP4 (SpliceAI=0)		1	0	0	0	1	0
ATM	synonymous_variant	NM_000051.4:c.7521C>T	NP_000042.3:p.Asp2507=	Unknown Significance	No criteria met		7	0	1	0	4	0
ATM	intron_variant	NM_000051.4:c.7630-37_7630-34delGTTT		Unknown Significance	BP4 (SpliceAI=0)		1	0	0	0	1	0
ATM	missense_variant	NM_000051.4:c.7740A>C	NP_000042.3:p.Arg2580Ser	Unknown Significance	No criteria met		1	0	1	0	0	0
ATM	missense_variant	NM_000051.4:c.7775C>G	NP_000042.3:p.Ser2592Cys	Unknown Significance	PP3 (Revel=0.765)<br>PS3_moderate (Functional study showing no kinase activity and failure to correct the radiosensitivity (PMID: 11805335))<br>PM3_supporting (In ClinVar the laboratories reported one patient with A-T but unknown phase)		3	0	2	0	1	0
ATM	intron_variant	NM_000051.4:c.7789-21T>G		Unknown Significance	BP4 (SpliceAI=0)		2	0	1	0	0	0
ATM	missense_variant	NM_000051.4:c.7871G>C	NP_000042.3:p.Cys2624Ser	Unknown Significance	PM2_supporting (Only 5 in GnomAD)		1	0	0	0	0	0
ATM	missense_variant	NM_000051.4:c.7875T>G	NP_000042.3:p.Asp2625Glu	Unknown Significance	No criteria met		1	0	0	0	0	0
ATM	missense_variant	NM_000051.4:c.7876G>C	NP_000042.3:p.Ala2626Pro	Unknown Significance	No criteria met		1	0	0	0	0	0
ATM	missense_variant	NM_000051.4:c.7912T>G	NP_000042.3:p.Trp2638Gly	Unknown Significance	PM2_supporting (Only 4 in GnomAD)		1	0	0	0	0	0
ATM	missense_variant	NM_000051.4:c.7919C>T	NP_000042.3:p.Thr2640Ile	Unknown Significance	ccDK		2	0	2	0	0	0
ATM	missense_variant	NM_000051.4:c.8014G>T	NP_000042.3:p.Asp2672Tyr	Unknown Significance	PM2_supporting (Only 1 in GnomAD)<br>PP3 (Revel=0.761)		1	0	0	0	0	0
ATM	missense_variant	NM_000051.4:c.8071C>T	NP_000042.3:p.Arg2691Cys	Unknown Significance	No criteria met		1	0	1	0	0	0
ATM	missense_variant	NM_000051.4:c.8072G>A	NP_000042.3:p.Arg2691His	Unknown Significance	BP4 (Revel=0.199)		1	0	1	0	0	0
ATM	missense_variant	NM_000051.4:c.8096C>T	NP_000042.3:p.Pro2699Leu	Unknown Significance	PM2_supporting (Absent from GnomAD)<br>PP3 (Revel=0.952)<br>PS3_supporting (Functional study showing ATM kinase inactive mutation (PMID: 19431188))<br>PM3_moderate (In ClinVar the laboratories reported at least 1 patient with Ataxia Telangiectasia in conjunction with another ATM alteration)		2	0	0	0	2	0
ATM	missense_variant	NM_000051.4:c.8153G>A	NP_000042.3:p.Gly2718Asp	Unknown Significance	PM2_supporting (Absent from GnomAD)<br>PP3 (Revel=0.941)		1	0	1	0	0	0
ATM	missense_variant	NM_000051.4:c.8165T>C	NP_000042.3:p.Leu2722Pro	Unknown Significance	PM2_supporting (Absent from GnomAD)<br>PP3 (Revel=0.947)		1	0	0	0	0	0
ATM	intron_variant	NM_000051.4:c.8269-2276G>T		Unknown Significance	No criteria met		2	0	2	0	0	0
ATM	missense_variant	NM_000051.4:c.8327T>C	NP_000042.3:p.Ile2776Thr	Unknown Significance	ccDK		3	0	1	0	1	0
ATM	missense_variant	NM_000051.4:c.8354A>G	NP_000042.3:p.Asp2785Gly	Unknown Significance	BP4 (Revel=0.143)<br>PM2_supporting (Absent from GnomAD)		1	0	1	0	0	0
ATM	missense_variant	NM_000051.4:c.8428A>C	NP_000042.3:p.Lys2810Gln	Unknown Significance	BP4 (Revel=0.117)		7	0	7	0	0	0
ATM	in-frame_deletion	NM_000051.4:c.8504_8518delGCATGGAAAAATTCT	NP_000042.3:p.Cys2835_Phe2839del	Unknown Significance	PM2_supporting (Absent from GnomAD)<br>PM3_supporting (1 family with A-T but unknown phase; PMID: 10980530)		2	0	1	0	1	0
ATM	missense_variant	NM_000051.4:c.8560C>T	NP_000042.3:p.Arg2854Cys	Unknown Significance	PP3 (Revel=0.904)		1	0	1	0	0	0
ATM	intron_variant	NM_000051.4:c.8585-357T>A		Unknown Significance	PM2_supporting (Absent from GnomAD)<br>PP3 (SpliceAI DG: 0.33and AG: 0.14. Possibly pseudo-exon. RAW score donor 0.34 and acceptor 0.14)		1	0	1	0	0	0
ATM	missense_variant	NM_000051.4:c.8624A>C	NP_000042.3:p.Asn2875Thr	Unknown Significance	PM2_supporting (Only 1 in GnomAD)<br>PP3 (Revel=0.929)		1	0	0	0	0	0
ATM	missense_variant	NM_000051.4:c.8625T>G	NP_000042.3:p.Asn2875Lys	Unknown Significance	PM2_supporting (Absent from GnomAD)<br>PP3 (Revel=0.819)		1	0	0	0	1	0
ATM	missense_variant	NM_000051.4:c.8732C>T	NP_000042.3:p.Thr2911Ile	Unknown Significance	PM2_supporting (Absent from GnomAD)<br>PP3 (Revel=0.809)<br>PM3_moderate (In ClinVar the laboratories reported A-T patients with the variant, but unknown or in trans phase)		2	0	0	0	0	0
ATM	missense_variant	NM_000051.4:c.8734A>G	NP_000042.3:p.Arg2912Gly	Unknown Significance	BP2_strong (In ClinVar laboratories reported that this variant has been observed in a compound heterozygous state in multiple individuals without Ataxia-Telangiectasia; GTR Lab IDs: 61756. 500031)<br>PP3 (Revel=0.883)		1	0	0	0	1	0
ATM	splice_region_variant	NM_000051.4:c.8787-15T>G		Unknown Significance	PM2_supporting (Absent from GnomAD)<br>PP3 (SpliceAI AL: 0.76 and DL: 0.81. Possibly exon skipping. RAW score natural acceptor changes from 0.86 to 0.10 and natural donor 0.94 to 0.14)		1	0	0	0	0	0
ATM	intron_variant	NM_000051.4:c.8787-25G>A		Unknown Significance	BP4 (SpliceAI=0)		1	0	0	0	1	0
ATM	intron_variant	NM_000051.4:c.8787-28T>C		Unknown Significance	BP4 (SpliceAI=0)		2	0	1	0	1	0

ATM	splice_region_variant	NM_000051.4:c.8787-6C>T		Unknown Significance	BP4 (SpliceAI=0.00)		2	0	2	0	0	0
ATM	intron_variant	NM_000051.4:c.8850+2029A>G		Unknown Significance	PM2_supporting (Absent from GnomAD)<br>PP3 (SpliceAI AG:0.55. Possibly cryptic splice site and no nearby donor site. RAW score changes from 0 to 0.55)		1	0	1	0	0	0
ATM	splice_region_variant	NM_000051.4:c.8850+4A>C		Unknown Significance	No criteria met		2	0	2	0	0	0
ATM	missense_variant	NM_000051.4:c.8965C>G	NP_000042.3:p.Gln2989Glu	Unknown Significance	BP4 (Revel=0.121)		1	0	1	0	0	0
ATM	missense_variant	NM_000051.4:c.9023G>C	NP_000042.3:p.Arg3008Pro	Unknown Significance	PM2_supporting (Only 1 in GnomAD)		2	0	0	0	1	0
ATM	missense_variant	NM_000051.4:c.902G>A	NP_000042.3:p.Gly301Asp	Unknown Significance	ccDK		3	0	1	0	0	0
ATM	missense_variant	NM_000051.4:c.9032T>G	NP_000042.3:p.Met3011Arg	Unknown Significance	PM2_supporting (Only 2 in GnomAD)<br>BP4 (Revel=0.221)		1	0	1	0	0	0
ATM	missense_variant	NM_000051.4:c.9086G>A	NP_000042.3:p.Gly3029Asp	Unknown Significance	BP4 (Revel=0.23)		5	0	4	0	0	0
ATM	missense_variant	NM_000051.4:c.9086G>T	NP_000042.3:p.Gly3029Val	Unknown Significance	No criteria met		1	0	1	0	1	0
ATM	missense_variant	NM_000051.4:c.9156G>T	NP_000042.3:p.Trp3052Cys	Unknown Significance	PM2_supporting (Absent from GnomAD)<br>PP3 (Revel=0.947)		1	0	0	0	0	0
ATM	synonymous_variant	NM_000051.4:c.9168G>A	NP_000042.3:p.Val3056=	Unknown Significance	PP3 (SpliceAI DG: 0.64 and AG: 0.46. Possibly two cryptic splice sites. RAW score alternative donor changes from 0.35 to 0.99 and alternative acceptor changes from 0.19 to 0.65)		3	0	2	0	0	0
ATM	missense_variant	NM_000051.4:c.94C>T	NP_000042.3:p.Arg32Cys	Unknown Significance	BP4 (Revel=0.181)		1	0	0	0	1	0
ATM	missense_variant	NM_000051.4:c.986G>A	NP_000042.3:p.Arg329Lys	Unknown Significance	No criteria met		1	0	1	0	0	0
BARD1	missense_variant	NM_000465.4:c.1013C>A	NP_000456.2:p.Thr338Asn	Unknown Significance	PM2_suppoting (GnomAD only 2)<br>BP4 (Revel=score 0.084 and BayesDel=-0.6266)		1	0	0	0	0	0
BARD1	in-frame_deletion	NM_000465.4:c.1075_1095del	NP_000456.2:p.Leu359_Pro365del	Unknown Significance	BP6 (Laboratories reported it benign in Clinvar)<br>PM4 (Protein length changed due to result of in-frame deletion)		176	0	116	0	23	0
BARD1	missense_variant	NM_000465.4:c.1115T>G	NP_000456.2:p.Val372Gly	Unknown Significance	BP4 (Revel=0.06 and bayesDel -0.401)<br>PM2_supporting (Absent from GnomAD)		1	0	0	0	0	0
BARD1	missense_variant	NM_000465.4:c.1282G>A	NP_000456.2:p.Gly428Arg	Unknown Significance	PM2_supporting (Absent from GnomAD)		1	0	1	0	0	0
BARD1	missense_variant	NM_000465.4:c.1307C>G	NP_000456.2:p.Ser436Cys	Unknown Significance	BP4 (Revel=0.237 and BayesDel=-0.21)<br>PM2_supporting (GnomAD only 5)		1	0	1	0	0	0
BARD1	splice_region_variant	NM_000465.4:c.1315-15TC		Unknown Significance	BP4 (SpliceAI=0.04)<br>PM2_supporting (Absent from GnomAD)		1	0	1	0	0	0
BARD1	intron_variant	NM_000465.4:c.1315-843dupT		Unknown Significance	No criteria met		98	0	61	0	9	0
BARD1	missense_variant	NM_000465.4:c.13C>T	NP_000456.2:p.Arg5Trp	Unknown Significance	PM2_supporting (Only 4 in GnomAD)		1	0	0	0	0	0
BARD1	missense_variant	NM_000465.4:c.1498G>A	NP_000456.2:p.Asp500Asn	Unknown Significance	No criteria met		1	0	1	0	0	0
BARD1	intron_variant	NM_000465.4:c.1568+2837T>G		Unknown Significance	PM2_supporting (Only 5 in GnomAD)<br>PP3 (SpliceAI AG: 0.14 and DG: 0.22. Possibly pseudo exon. RAW score acceptor 0.35 and donor 0.23)		1	0	1	0	0	0
BARD1	splice_region_variant	NM_000465.4:c.1569-13C>G		Unknown Significance	PP3 (SpliceAI; AL: 0.94. AG: 0.36 and DL: 0.62. Possibly exon-skipping or intron-retention. RAW score naturel acceptor changes from 0.96 to 0.02, alternative acceptor 0.36 and naturel donor changes from 0.92 to 0.30)		7	0	5	0	2	0
BARD1	intron_variant	NM_000465.4:c.1569-2410T>A		Unknown Significance	PM2_supporting (Absent from GnomAD)		1	0	1	0	0	0
BARD1	intron_variant	NM_000465.4:c.1569-592T>C		Unknown Significance	PM2_supporting (Absent from GnomAD)		1	0	1	0	0	0
BARD1	splice_region_variant	NM_000465.4:c.158+3A>C		Unknown Significance	PM2_supporting (Absent from GnomAD)<br>PP3 (SpliceAI DL: 0.83 and DG: 0.38. Possibly exon skipping or intron retention. RAW score natural donor changes from 0.98 to 0.15 and alternative donor changes from 0.25 to 0.63)		1	0	1	0	0	0
BARD1	intron_variant	NM_000465.4:c.158+4382C>T		Unknown Significance	No criteria met		55	0	26	0	8	0
BARD1	missense_variant	NM_000465.4:c.1586G>A	NP_000456.2:p.Arg529Gln	Unknown Significance	No criteria met	SpliceAI AG: 0.70 and AL: 0.35. Possibly partiel exon skipping. RAW score natural acceptor changes from 0.96 to 0.61 and alternative acceptor changes from 0 to 0.70	1	0	1	0	0	0
BARD1	synonymous_variant	NM_000465.4:c.1593C>T	NP_000456.2:p.Val531=	Unknown Significance	PP3 (SpliceAI AG:0.20. Possibly partiel exon deletion. RAW score alternative splice site changes from 0.12 to 0.31 and the naturel splice site changes from 0.95 to 0.85)		5	0	5	0	0	0
BARD1	intron_variant	NM_000465.4:c.159-735_159-732delCACT		Unknown Significance	PM2_Supporting (Only 1 in GnomAD)		1	0	1	0	0	0
BARD1	missense_variant	NM_000465.4:c.1598A>T	NP_000456.2:p.Tyr533Phe	Unknown Significance	BS3 (Functional study showing no damaing effect (PMID: 30925164))	SpliceAI AG: 0.63 and AL: 0.23. Possibly partiel exon deletion. RAW score natural acceptor changes from 0.96 to 0.73 and alternative acceptor changes from 0.12 to 0.74	1	0	1	0	0	0
BARD1	missense_variant	NM_000465.4:c.1646A>G	NP_000456.2:p.Asn549Ser	Unknown Significance	BP4 (Revel=0.111 and BayesDel=0.463)		1	0	0	0	1	0
BARD1	missense_variant	NM_000465.4:c.1693C>T	NP_000456.2:p.Arg565Cys	Unknown Significance	No criteria met		1	0	1	0	0	0
BARD1	missense_variant	NM_000465.4:c.1718T>C	NP_000456.2:p.Ile573Thr	Unknown Significance	No criteria met		1	0	1	0	0	0
BARD1	missense_variant	NM_000465.4:c.1757G>T	NP_000456.2:p.Ser586Ile	Unknown Significance	BP4 (Revel=0.248 and BayesDel=-0.339)		3	0	3	0	0	0
BARD1	synonymous_variant	NM_000465.4:c.1767A>G	NP_000456.2:p.Ala589=	Unknown Significance	PM2_supporting (Absent from GnomAD)<br>PP3 (SpliceAI DG: 0.22. Possibly partiel exon deletion. RAW score alternative splice site changes from 0 to 0.22 and naturel donor site changes from 0.99 to 0.98)		1	0	0	0	0	0
BARD1	missense_variant	NM_000465.4:c.1813A>G	NP_000456.2:p.Thr605Ala	Unknown Significance	PM2_supporting (Only 1 in GnomAD)	SpliceAI AG: 0.92. Possibly partiel exon deletion. RAW score alternative acceptor 0.92 and natural acceptor changes from 0.97 to 0.94	1	0	0	0	1	0
BARD1	intron_variant	NM_000465.4:c.1903+3400A>G		Unknown Significance	PP3 (SpliceAI AG: 0.28 and DG: 0.16. Possibly pseudo exon. RAW score acceptor 0.35 and donor 0.16)		1	0	1	0	0	0
BARD1	intron_variant	NM_000465.4:c.1903+3436G>A		Unknown Significance	PP3 (SpliceAI AG: 0.29 and DG: 0.17. Possibly pseudo exon. RAW score acceptor 0.35 and donor 0.18)		1	0	0	0	0	0
BARD1	intron_variant	NM_000465.4:c.1904-485A>G		Unknown Significance	PP3 (SpliceAI DG: 0.22 and AG: 0.15. Possibly pseudo-exon. RAW score donor 0.52 and acceptor 0.27)		2	0	1	0	1	0
BARD1	intron_variant	NM_000465.4:c.1904-84_1904-81delCTTA		Unknown Significance	PP3 (SpliceAI AG: 0.11 and DG: 0.21. Possibly pseudo exon. RAW score acceptor 0.15 and donor 0.28)		1	0	1	0	0	0
BARD1	missense_variant	NM_000465.4:c.1915T>C	NP_000456.2:p.Cys639Arg	Unknown Significance	No criteria met		7	0	6	0	0	0
BARD1	missense_variant	NM_000465.4:c.1943A>G	NP_000456.2:p.Glu648Gly	Unknown Significance	PM2_supporting (Absent from GnomAD)		2	0	2	0	0	0
BARD1	missense_variant	NM_000465.4:c.1961C>T	NP_000456.2:p.Pro654Leu	Unknown Significance	BP4 (Revel=0.041 and BayesDel=-0.4133)<br>PM2_supporting (Absent from GnomAD)		1	0	1	0	0	0
BARD1	missense_variant	NM_000465.4:c.2125C>T	NP_000456.2:p.Pro709Ser	Unknown Significance	BP4 (Revel=0.24 and BayesDel=-0.215)		2	0	0	0	0	0
BARD1	missense_variant	NM_000465.4:c.2153A>G	NP_000456.2:p.Asn718Ser	Unknown Significance	BP4 (Revel=0.174 and BayesDel=-0.475)		1	0	1	0	0	0
BARD1	intron_variant	NM_000465.4:c.216-591A>G		Unknown Significance	No criteria met		109	1	66	0	14	0
BARD1	intron_variant	NM_000465.4:c.216-617G>A		Unknown Significance	PP3 (SpliceAI DG: 0.22. Possibly cryptic splite site. RAW score donor changes from 0.03 to 0.25. No acceptor site nearby)		8	0	2	0	2	0
BARD1	missense_variant	NM_000465.4:c.2311G>C	NP_000456.2:p.Glu771Gln	Unknown Significance	PM2_supporting (Absent from GnomAD)<br>BP4 (Revel=0.024 and BayesDel=-0.66312)		1	0	1	0	0	0
BARD1	in-frame_deletion	NM_000465.4:c.26_40delACCGGCAGCCGAGGA	NP_000456.2:p.Asn9_Arg13del	Unknown Significance	PM4 (Protein length changes as a result of in-frame deletion in non-repeat variant)		1	0	1	0	0	0
BARD1	intron_variant	NM_000465.4:c.365-100A>G		Unknown Significance	PM2_supporting (Only 4 in GnomAD)<br>PP3 (SpliceAI DG:0.23. Possibly cryptic splice site. No nearby acceptor. RAW score 0.23)		1	0	1	0	0	0
BARD1	intron_variant	NM_000465.4:c.365-189A>C		Unknown Significance	PP3 (SpliceAI AG: 0.34 and DG: 0.36. Possibly pseudo exon. RAW score acceptor 0.50 and donor 0.51)<br>PM2_supporting (GnomAD only 2)		1	0	0	0	1	0
BARD1	missense_variant	NM_000465.4:c.386G>C	NP_000456.2:p.Arg129Thr	Unknown Significance	BP4 (Revel=0.057 and BayesDel=-0.3996)<br>PM2_supporting (Absent from GnomAD)		1	0	0	0	0	0
BARD1	missense_variant	NM_000465.4:c.464G>C	NP_000456.2:p.Arg155Thr	Unknown Significance	PM2_supporting (Absent from GnomAD)		1	0	0	0	1	0
BARD1	missense_variant	NM_000465.4:c.54C>G	NP_000456.2:p.Asn18Lys	Unknown Significance	No criteria met		1	0	1	0	0	0
BARD1	missense_variant	NM_000465.4:c.580A>T	NP_000456.2:p.Arg194Trp	Unknown Significance	BP4 (Revel=0.09 and BayesDel=-0.30)<br>PM2_supporting (Only 5 in GnomAD)		1	0	0	0	0	0
BARD1	missense_variant	NM_000465.4:c.617A>G	NP_000456.2:p.Gln206Arg	Unknown Significance	BP4 (Revel=0.024 and BayesDel=-0.49)<br>PM2_supporting (Absent from GnomAD)		1	0	1	0	0	0
BARD1	missense_variant	NM_000465.4:c.632T>C	NP_000456.2:p.Leu211Ser	Unknown Significance	No criteria met		2	0	1	0	1	0
BARD1	missense_variant	NM_000465.4:c.647A>T	NP_000456.2:p.Gln216Leu	Unknown Significance	BP4 (Revel=0.042 and BayesDel=-0.38)<br>PM2_supporting (Absent from GnomAD)		1	0	0	0	0	0
BARD1	missense_variant	NM_000465.4:c.652T>A	NP_000456.2:p.Trp218Arg	Unknown Significance	PM2_supporting (Absent from GnomAD)		1	0	0	0	0	0
BARD1	missense_variant	NM_000465.4:c.659T>C	NP_000456.2:p.Leu220Ser	Unknown Significance	BP4 (Revel=0.142 and BayesDel=-0.310)		3	0	3	0	1	0

BARD1	missense_variant	NM_000465.4:c.667G>C	NP_000456.2:p.Glu223Gln	Unknown Significance	BP4 (Revel=0.102 and BayesDel=-0.42)<br>PM2_supporting (Absent from GnomAD)		1	0	1	0	0	0
BARD1	missense_variant	NM_000465.4:c.701A>T	NP_000456.2:p.Glu234Val	Unknown Significance	PM2_supporting (Absent in GnomAD)<br>BP4 (Revel=0.197 and BayesDel=-0.24)		2	0	2	0	0	0
BARD1	missense_variant	NM_000465.4:c.709C>G	NP_000456.2:p.Gln237Glu	Unknown Significance	BP4 (Revel=0.175 and BayesDel=-0.56)		4	0	2	0	0	0
BARD1	missense_variant	NM_000465.4:c.722C>G	NP_000456.2:p.Ser241Cys	Unknown Significance	BP6 (Laboratories reported it benign in ClinVar)		1	0	1	0	0	0
BRCA1	missense_variant	NM_007294.4:c.1030G>A	NP_009225.1:p.Ala344Thr	Unknown Significance	ccDK		4	0	3	0	1	0
BRCA1	missense_variant	NM_007294.4:c.1100:c._G	NP_009225.1:p.Thr367Ser	Unknown Significance	ccDK		1	0	1	0	0	0
BRCA1	missense_variant	NM_007294.4:c.1117A>C	NP_009225.1:p.Ile373Leu	Unknown Significance	ccDK		1	0	1	0	0	0
BRCA1	missense_variant	NM_007294.4:c.1589A>C	NP_009225.1:p.Glu530Ala	Unknown Significance	ccDK		2	0	0	0	0	0
BRCA1	missense_variant	NM_007294.4:c.1786C>G	NP_009225.1:p.Leu596Val	Unknown Significance	ccDK		1	0	1	0	0	0
BRCA1	intron_variant	NM_007294.4:c.-19-31T>G		Unknown Significance	ccDK		1	0	0	0	0	0
BRCA1	intron_variant	NM_007294.4:c.-20+496A>G		Unknown Significance	No criteria met		1	0	0	0	1	0
BRCA1	missense_variant	NM_007294.4:c.2993T>A	NP_009225.1:p.Leu998Gln	Unknown Significance	ccDK		1	0	1	0	0	0
BRCA1	splice_region_variant	NM_007294.4:c.302-15C>G		Unknown Significance	ccDK		2	0	0	0	2	0
BRCA1	missense_variant	NM_007294.4:c.3230G>A	NP_009225.1:p.Gly1077Glu	Unknown Significance	ccDK		1	0	1	0	0	0
BRCA1	missense_variant	NM_007294.4:c.3454G>A	NP_009225.1:p.Asp1152Asn	Unknown Significance	ccDK		1	0	0	0	1	0
BRCA1	missense_variant	NM_007294.4:c.3889T>A	NP_009225.1:p.Ser1297Thr	Unknown Significance	ccDK		1	0	1	0	0	0
BRCA1	splice_region_variant	NM_007294.4:c.4096+3A>G		Unknown Significance	ENIGMA		11	0	8	0	5	0
BRCA1	missense_variant	NM_007294.4:c.4166G>A	NP_009225.1:p.Ser1389Asn	Unknown Significance	ccDK		1	0	1	0	0	0
BRCA1	intron_variant	NM_007294.4:c.4185+1622G>C		Unknown Significance	PM2_supporting (Absent from GnomAD)		1	0	1	0	0	0
BRCA1	intron_variant	NM_007294.4:c.4185+625G>A		Unknown Significance	No criteria met		5	0	3	0	0	0
BRCA1	intron_variant	NM_007294.4:c.4185+659G>A		Unknown Significance	No criteria met		1	0	0	0	0	0
BRCA1	intron_variant	NM_007294.4:c.4186-2784G>C		Unknown Significance	No criteria met		1	0	1	0	0	0
BRCA1	intron_variant	NM_007294.4:c.4186-50A>C		Unknown Significance	PM2_supporting (Absent from GnomAD)<br>PP3 (SpliceAI AG:0.27. Possibly cryptic splice site or intron retention. RAW score acceptor changes from 0.01 to 0.28)		1	0	0	0	0	0
BRCA1	missense_variant	NM_007294.4:c.4193A>G	NP_009225.1:p.Asp1398Gly	Unknown Significance	ccDK		2	0	1	0	1	0
BRCA1	intron_variant	NM_007294.4:c.4357+1912G>A		Unknown Significance	PM2_supporting (Only 1 in GnomAD)		1	0	1	0	0	0
BRCA1	intron_variant	NM_007294.4:c.4357+2735C>G		Unknown Significance	No criteria met		3	0	3	0	0	0
BRCA1	intron_variant	NM_007294.4:c.441+1309G>A		Unknown Significance	No criteria met		4	1	1	0	1	0
BRCA1	missense_variant	NM_007294.4:c.4625C>G	NP_009225.1:p.Ser1542Cys	Unknown Significance	ccDK		1	0	1	0	0	0
BRCA1	splice_region_variant	NM_007294.4:c.4676-8C>G		Unknown Significance	ccDK		6	0	4	0	1	0
BRCA1	intron_variant	NM_007294.4:c.4987-857A>C		Unknown Significance	PM2_supporting (Absent from GnomAD)		2	0	1	0	0	0
BRCA1	missense_variant	NM_007294.4:c.5050A>G	NP_009225.1:p.Thr1684Ala	Unknown Significance	ccDK		2	0	1	0	0	0
BRCA1	intron_variant	NM_007294.4:c.5074+156G>A		Unknown Significance	PP3 (SpliceAI DG: 0.43. Cryptic splice site or intron retension. RAW score donor changes from 0.02 to 0.45)		1	0	0	0	0	0
BRCA1	splice_region_variant	NM_007294.4:c.5075-7T>C		Unknown Significance	ccDK		1	0	0	0	0	0
BRCA1	intron_variant	NM_007294.4:c.5193+123delG		Unknown Significance	PP3 (SpliceAI AG: 0.47. Possibly cryptic splice site. RAW score acceptor changes from 0.02 to 0.49)		3	0	3	0	0	0
BRCA1	intron_variant	NM_007294.4:c.5193+838A>T		Unknown Significance	PM2_supporting (Absent from GnomAD)<br>PP3 (SpliceAI AG: 0.85 and DG: 0.95. Possibly pseudo-exon. RAW score acceptor 0.87 and donor 0.95)		2	0	1	0	1	0
BRCA1	intron_variant	NM_007294.4:c.5333-716C>T		Unknown Significance	PM2_supporting (Absent from GnomAD)		1	0	0	0	0	0
BRCA1	missense_variant	NM_007294.4:c.5348T>C	NP_009225.1:p.Met1783Thr	Unknown Significance	ccDK		1	0	0	0	1	0
BRCA1	missense_variant	NM_007294.4:c.587A>T	NP_009225.1:p.Tyr196Phe	Unknown Significance	ccDK		1	0	0	0	0	0
BRCA1	intron_variant	NM_007294.4:c.593+112G>A		Unknown Significance	No criteria met		1	0	1	0	0	0
BRCA1	intron_variant	NM_007294.4:c.594-532G>A		Unknown Significance	No criteria met		15	0	9	0	4	0
BRCA1	missense_variant	NM_007294.4:c.656A>T	NP_009225.1:p.Asp219Val	Unknown Significance	PM2_supporting (Absent from GnomAD)	SpliceAI DL: 0.50 and AL: 0.58. Possibly exon skipping. RAW score donor changes from 0.79 to 0.29 and acceptor changes from 0.83 to 0.25	1	0	1	0	0	0
BRCA1	missense_variant	NM_007294.4:c.922A>T	NP_009225.1:p.Ser308Cys	Unknown Significance	ccDK		1	0	1	0	0	0
BRCA2	intron_variant	NM_000059.4:c.317-433T>A		Unknown Significance	PM2_supporting (Absent from GnomAD)		1	0	1	0	0	0
BRCA2	in-frame_deletion	NM_000059.4:c.3733_3738delGAGAAT	NP_000050.3:p.Glu1245_Asn1246del	Unknown Significance	ccDK		1	0	1	0	0	0
BRCA2	synonymous_variant	NM_000059.4:c.436C>T	NP_000050.3:p.Leu146=	Unknown Significance	ccDK		1	0	1	0	0	0
BRCA2	splice_region_variant	NM_000059.4:c.517-7C>T		Unknown Significance	ccDK		1	0	1	0	0	0
BRCA2	in-frame_deletion	NM_000059.4:c.5272_5274delAAT	NP_000050.3:p.Asn1758del	Unknown Significance	ccDK		7	0	6	0	0	0
BRCA2	missense_variant	NM_000059.4:c.565G>T	NP_000050.3:p.Asp189Tyr	Unknown Significance	ccDK		1	0	1	0	0	0
BRCA2	splice_region_variant	NM_000059.4:c.6842-4T>C		Unknown Significance	ccDK		1	0	1	0	0	0
BRCA2	missense_variant	NM_000059.4:c.6842G>A	NP_000050.3:p.Gly2281Glu	Unknown Significance	ccDK		1	0	0	0	0	0
BRCA2	intron_variant	NM_000059.4:c.7436-366delG		Unknown Significance	PM2_supporting (Absent from GnomAD)		2	0	0	0	0	0
BRCA2	missense_variant	NM_000059.4:c.7448G>A	NP_000050.3:p.Ser2483Asn	Unknown Significance	ccDK		1	0	0	0	0	0
BRCA2	missense_variant	NM_000059.4:c.7504C>T	NP_000050.3:p.Arg2502Cys	Unknown Significance	ccDK		1	0	1	0	0	0
BRCA2	in-frame_deletion	NM_000059.4:c.7584_7586delAGG	NP_000050.3:p.Gly2529del	Unknown Significance	PM2_supporting (Absent from GnomAD)		1	0	1	0	0	0
BRCA2	missense_variant	NM_000059.4:c.7616A>G	NP_000050.3:p.Gln2539Arg	Unknown Significance	ccDK		2	0	1	0	0	0
BRCA2	missense_variant	NM_000059.4:c.7883T>C	NP_000050.3:p.Ile2628Thr	Unknown Significance	ccDK		1	0	1	0	1	0
BRCA2	missense_variant	NM_000059.4:c.7895C>A	NP_000050.3:p.Ala2632Glu	Unknown Significance	ccDK		1	0	1	0	0	0
BRCA2	missense_variant	NM_000059.4:c.8116A>G	NP_000050.3:p.Asn2706Asp	Unknown Significance	ccDK		1	0	0	0	0	0
BRCA2	missense_variant	NM_000059.4:c.8131G>A	NP_000050.3:p.Ala2711Thr	Unknown Significance	BP4 (Inside functional domain and BayesDel=-0.20 and SpliceAI=0.0)<br>PM2_supporting (Absent from GnomAD)		1	0	1	0	0	0
BRCA2	missense_variant	NM_000059.4:c.8140C>G	NP_000050.3:p.Gln2714Glu	Unknown Significance	ccDK		1	0	1	0	0	0
BRCA2	intron_variant	NM_000059.4:c.8331+1922A>G		Unknown Significance	PM2_supporting (Absent from GnomAD)		1	0	1	0	0	0
BRCA2	intron_variant	NM_000059.4:c.8331+388T>G		Unknown Significance	No criteria met		4	0	3	0	0	0
BRCA2	missense_variant	NM_000059.4:c.8351G>A	NP_000050.3:p.Arg2784Gln	Unknown Significance	ccDK		2	0	0	0	0	0
BRCA2	missense_variant	NM_000059.4:c.8452G>A	NP_000050.3:p.Val2818Ile	Unknown Significance	ccDK		1	0	1	0	0	0
BRCA2	splice_region_variant	NM_000059.4:c.8488-6T>G		Unknown Significance	ccDK		1	0	0	0	0	0
BRCA2	intron_variant	NM_000059.4:c.8633-1299C>T		Unknown Significance	No criteria met		3	0	3	0	0	0
BRCA2	missense_variant	NM_000059.4:c.8663G>A	NP_000050.3:p.Arg2888His	Unknown Significance	ccDK		1	0	1	0	0	0
BRCA2	intron_variant	NM_000059.4:c.8755-108A>G		Unknown Significance	PM2_supporting (Absent from GnomAD)		1	0	1	0	0	0
BRCA2	missense_variant	NM_000059.4:c.8902A>G	NP_000050.3:p.Thr2968Ala	Unknown Significance	ccDK		1	0	1	0	0	0
BRCA2	splice_region_variant	NM_000059.4:c.8954-17C>T		Unknown Significance	BP4 (SpliceAI=0.04)		2	0	1	0	0	0
BRCA2	missense_variant	NM_000059.4:c.8957T>C	NP_000050.3:p.Ile2986Thr	Unknown Significance	BP4 (In functional domain. BayesDel=-0.27 and SpliceAI=0.06)		1	0	0	0	0	0
BRCA2	missense_variant	NM_000059.4:c.9171C>G	NP_000050.3:p.Phe3057Leu	Unknown Significance	ccDK		1	0	0	0	0	0
BRCA2	intron_variant	NM_000059.4:c.9256+3731G>T		Unknown Significance	PP3 (SpliceAI AG: 0.31 and DG: 0.11. Possibly cryptic splice site or pseudo-exon. RAW score acceptor 0.49 and donor 0.20)		1	0	1	0	0	0
BRCA2	intron_variant	NM_000059.4:c.9257-28A>G		Unknown Significance	No criteria met		1	0	0	0	0	0
BRIP1	missense_variant	NM_032043.3:c.1093A>G	NP_114432.2:p.Ile365Val	Unknown Significance	BP4 (Revel=0.117 and BayesDel=-0.50149)<br>PM2_supporting (Only 3 in GnomAD)		1	0	1	0	0	0
BRIP1	intron_variant	NM_032043.3:c.1140+612T>C		Unknown Significance	PM2_supporting (Absent from GnomAD)<br>PP3 (SpliceAI; AG: 0.29 and DG: 0.33. Possibly pseudo-exon. RAW-score acceptor site 0.34 and donor site 0.47)		2	0	2	0	0	0
BRIP1	missense_variant	NM_032043.3:c.1247G>A	NP_114432.2:p.Arg416Gln	Unknown Significance	ccDK		1	0	0	0	0	0
BRIP1	missense_variant	NM_032043.3:c.1255C>T	NP_114432.2:p.Arg419Trp	Unknown Significance	ccDK		10	0	7	0	1	0
BRIP1	intron_variant	NM_032043.3:c.1340+2153A>G		Unknown Significance	PP3 (SpliceAI; AG: 0.30 and DG: 0.32. Possibly pseudo-exon. RAW-score acceptor site 0.39 and donor site 0.37)		12	0	8	0	1	0
BRIP1	missense_variant	NM_032043.3:c.1352C>T	NP_114432.2:p.Ala451Val	Unknown Significance	BP4 (Revel=0.146 and BayesDel=-0.35)<br>PM2_supporting (Only 5 in GnomAD)		1	0	1	0	0	0
BRIP1	missense_variant	NM_032043.3:c.139C>G	NP_114432.2:p.Pro47Ala	Unknown Significance	ccDK		7	0	3	0	3	0
BRIP1	missense_variant	NM_032043.3:c.1433A>G	NP_114432.2:p.His478Arg	Unknown Significance	BP6 (Laboratories reported it benign in ClinVar)		1	0	0	0	0	0
BRIP1	intron_variant	NM_032043.3:c.1474-1510_1474-1509insG		Unknown Significance	No criteria met		19	0	14	0	3	0
BRIP1	missense_variant	NM_032043.3:c.1547T>A	NP_114432.2:p.Val516Glu	Unknown Significance	ccDK		1	0	0	0	1	0
BRIP1	missense_variant	NM_032043.3:c.1660C>G	NP_114432.2:p.Gln554Glu	Unknown Significance	BP4 (Revel=0.161 and BayesDel=-0.24)		1	0	0	0	0	0
BRIP1	in-frame_deletion	NM_032043.3:c.1687_1689delGAT	NP_114432.2:p.Asp563del	Unknown Significance	ccDK		11	0	9	0	2	0
BRIP1	splice_region_variant	NM_032043.3:c.1795-9T>G		Unknown Significance	No criteria met		1	0	1	0	0	0
BRIP1	missense_variant	NM_032043.3:c.1899C>G	NP_114432.2:p.Ile633Met	Unknown Significance	BP4 (Revel=0.156 and BayesDel=-0.37)		1	0	0	0	1	0

BRIP1	intron_variant	NM_032043.3:c.1935+1533A>G		Unknown Significance	PM2_supporting (Only 1 in GnomAD)		1	0	1	0	0	0
BRIP1	splice_region_variant	NM_032043.3:c.1935+4_1935+7delAGTT		Unknown Significance	PP3 (SpliceAI DL:0.76, AL: 0.75, DG: 0.36 and AG: 0.37. Possibly exon skipping and pseudo-exon. RAW score naturel donor site changes from 0.77 to 0 and natural acceptor site changes from 0.87 to 0.12. Alternative donor site changes from 0.38 to 0.74 and alternative acceptor changes from 0.11 to 0.47)<br>PM2_supporting (Absent from GnomAD)		1	0	1	0	0	0
BRIP1	intron_variant	NM_032043.3:c.1936-1701A>G		Unknown Significance	PM2_supporting (Only 2 in GnomAD)<br>PP3 (SpliceAI; AG: 0.43 and DG: 0.41. Possibly pseudo-exon. RAW-score acceptor site 0.44 and donor site 0.41).		1	0	0	0	1	0
BRIP1	intron_variant	NM_032043.3:c.2097+14998A>C		Unknown Significance	No criteria met		1	0	0	0	0	0
BRIP1	missense_variant	NM_032043.3:c.2146A>G	NP_114432.2:p.Asn716Asp	Unknown Significance	BP4 (Revel=0.229 and BayesDel=-0.44)<br>PM2_supporting (Absent from GnomAD)		1	0	1	0	0	0
BRIP1	missense_variant	NM_032043.3:c.2233G>A	NP_114432.2:p.Ala745Thr	Unknown Significance	BS3 (Functional study showing no damanging effect and does not affect BRIP1 function (PMID: 31822495))		1	0	0	0	0	0
BRIP1	missense_variant	NM_032043.3:c.2325T>G	NP_114432.2:p.Asn775Lys	Unknown Significance	No criteria met		1	0	0	0	0	0
BRIP1	intron_variant	NM_032043.3:c.2379+3581G>C		Unknown Significance	PM2_supporting (Absent from GnomAD)		1	0	0	0	1	0
BRIP1	missense_variant	NM_032043.3:c.2440C>T	NP_114432.2:p.Arg814Cys	Unknown Significance	BP6 (Laboratories reported it benign in ClinVar)		1	0	1	0	0	0
BRIP1	missense_variant	NM_032043.3:c.2441G>A	NP_114432.2:p.Arg814His	Unknown Significance	No criteria met		11	0	6	0	2	0
BRIP1	synonymous_variant	NM_032043.3:c.2484C>T	NP_114432.2:p.Ala828=	Unknown Significance	No criteria met		1	0	0	0	0	0
BRIP1	splice_region_variant	NM_032043.3:c.2493-10T>A		Unknown Significance	PM2_supporting (Only 1 in GnomAD)<br>PP3 (SpliceAI; AL: 0.30 and DL: 0.37. Possibly exon skipping. RAW-score acceptor site changes from 0.69 to 0.38 and donor site changes from 0.59 to 0.23)		4	0	4	0	0	0
BRIP1	intron_variant	NM_032043.3:c.2576-1055T>C		Unknown Significance	PM2_supporting (Absent from GnomAD)<br>PP3 (SpliceAI DG: 0.20 and AG: 0.11. Possibly pseudo-exon. RAW score donor changes from 0.34 to 0.54 and acceptor changes from 0.22 to 0.33)		1	0	1	0	0	0
BRIP1	intron_variant	NM_032043.3:c.2576-968C>T		Unknown Significance	No criteria met		1	0	0	0	0	0
BRIP1	in-frame_deletion	NM_032043.3:c.258_269delTTGTTGTGCATG	NP_114432.2:p.Cys87_Cys90del	Unknown Significance	No criteria met		2	0	1	0	0	0
BRIP1	missense_variant	NM_032043.3:c.2840A>G	NP_114432.2:p.Gln947Arg	Unknown Significance	All variants after c.2576 in BRIP1 should be C3, decided by ccDK.		1	0	0	0	0	0
BRIP1	splice_donor_variant	NM_032043.3:c.2905+2T>A		Unknown Significance	All variants after c.2576 in BRIP1 should be C3, decided by ccDK.<br>PVS1_moderate (Skip exon 19, preserves reading frame, unknown protein function, variant removes <10% af protein)<br>PM2 (Only 1 in GnomAD)	SpliceAI DL:0.87. Possibly exon skipping or partiel exon deletion. RAW score naturel donor site changes from 0.87 to 0 and alternative donor site changes from 0.25 to 0.31	1	0	1	0	0	0
BRIP1	missense_variant	NM_032043.3:c.293A>G	NP_114432.2:p.Asn98Ser	Unknown Significance	BP4 (Revel=0.029 and BayesDel=-0.74)		1	0	1	0	0	0
BRIP1	missense_variant	NM_032043.3:c.3050C>T	NP_114432.2:p.Pro1017Leu	Unknown Significance	All variants after c.2576 in BRIP1 should be C3, decided by ccDK.		4	0	4	0	0	0
BRIP1	synonymous_variant	NM_032043.3:c.3069C>T	NP_114432.2:p.Leu1023=	Unknown Significance	All variants after c.2576 in BRIP1 should be C3, decided by ccDK.<br>BP4 (SpliceAI=0)<br>BP7 (Silent variant and no impact on splicing)		1	0	1	0	0	0
BRIP1	missense_variant	NM_032043.3:c.3103C>T	NP_114432.2:p.Arg1035Cys	Unknown Significance	All variants after c.2576 in BRIP1 should be C3, decided by ccDK.		2	0	1	0	0	0
BRIP1	missense_variant	NM_032043.3:c.3149C>A	NP_114432.2:p.Thr1050Asn	Unknown Significance	All variants after c.2576 in BRIP1 should be C3, decided by ccDK.<br>BP4 (BayesDel=-0.445 and REVEL=0.124)		1	0	1	0	0	0
BRIP1	missense_variant	NM_032043.3:c.316C>T	NP_114432.2:p.Arg106Cys	Unknown Significance	BP4 (Revel=0.067 and BayesDel=-0.557)		3	0	1	0	0	0
BRIP1	missense_variant	NM_032043.3:c.3205C>G	NP_114432.2:p.Gln1069Glu	Unknown Significance	All variants after c.2576 in BRIP1 should be C3, decided by ccDK.<br>BP4 (Revel=0.218 and BayesDel=-0.34)<br>PM2_supporting (Absent from GnomAD)		1	0	1	0	0	0
BRIP1	missense_variant	NM_032043.3:c.3254G>C	NP_114432.2:p.Arg1085Thr	Unknown Significance	All variants after c.2576 in BRIP1 should be C3, decided by ccDK.<br>BP4 (Revel=0.073 and BayesDel=-0.44)<br>PM2_supporting (Absent from GnomAD)		1	0	1	0	0	0
BRIP1	missense_variant	NM_032043.3:c.3371A>G	NP_114432.2:p.Glu1124Gly	Unknown Significance	All variants after c.2576 in BRIP1 should be C3, decided by ccDK.		1	0	1	0	0	0
BRIP1	disruptive_in-frame_insertion	NM_032043.3:c.3404_3405insCAG	NP_114432.2:p.Glu1135delinsAspArg	Unknown Significance	All variants after c.2576 in BRIP1 should be C3, decided by ccDK.<br>PM4 (Protein length because of in-frame insertion)<br>PM2_supporting (Absent from GnomAD)		1	0	1	0	0	0
BRIP1	missense_variant	NM_032043.3:c.3444C>A	NP_114432.2:p.Asp1148Glu	Unknown Significance	All variants after c.2576 in BRIP1 should be C3, decided by ccDK.		2	0	2	0	0	0
BRIP1	synonymous_variant	NM_032043.3:c.3498A>G	NP_114432.2:p.Leu1166=	Unknown Significance	All variants after c.2576 in BRIP1 should be C3, decided by ccDK.<br>BP4 (SpliceAI=0.00)<br>BP7 (Silent variant with SpliceAI=0.00)		1	0	1	0	0	0
BRIP1	synonymous_variant	NM_032043.3:c.3519T>C	NP_114432.2:p.Ile1173=	Unknown Significance	All variants after c.2576 in BRIP1 should be C3, decided by ccDK.<br>BP4 (SpliceAI=0.00)<br>BP7 (Silent variant and SpliceAI=0.00)		1	0	0	0	1	0
BRIP1	missense_variant	NM_032043.3:c.3529A>C	NP_114432.2:p.Lys1177Gln	Unknown Significance	All variants after c.2576 in BRIP1 should be C3, decided by ccDK.<br>No criteria met		1	0	0	0	0	0
BRIP1	splice_region_variant	NM_032043.3:c.380-17T>A		Unknown Significance	PP3 (SpliceAI AL:0.24 and DL: 0.18. Possibly exon skipping. RAW score acceptor changes from 0.55 to 0.31 and donor changes from 0.87 to 0.69)		2	0	0	0	1	0
BRIP1	missense_variant	NM_032043.3:c.415T>G	NP_114432.2:p.Ser139Ala	Unknown Significance	BP4 (Revel=0.126 and BayesDel=-0.234)		2	0	1	0	0	0
BRIP1	missense_variant	NM_032043.3:c.475A>G	NP_114432.2:p.Lys159Glu	Unknown Significance	PM2_supporting (Absent from GnomAD)	SpliceAI AL: 0.19 and DL: 0.20. Possibly exon skipping. RAW score acceptor changes from 0.55 to 0.35 and donor changes from 0.87 to 0.68	1	0	1	0	0	0
BRIP1	missense_variant	NM_032043.3:c.550G>T	NP_114432.2:p.Asp184Tyr	Unknown Significance	PS3 (Functional study showing exon 5 skipping (PMID: 30230034))		2	0	1	0	0	0
BRIP1	intron_variant	NM_032043.3:c.627+1281G>A		Unknown Significance	PM2_supporting (Absent from GnomAD)<br>PP3 (SpliceAI AG: 0.53- Possibly cryptic splice site, no nearby donor site. RAW score 0.55)		1	0	1	0	0	0
BRIP1	splice_region_variant	NM_032043.3:c.627+5G>A		Unknown Significance	ccDK		4	0	2	0	0	0
BRIP1	splice_region_variant	NM_032043.3:c.627+6T>C		Unknown Significance	PM2_supporting (Absent from GnomAD)		2	0	0	0	2	0
BRIP1	intron_variant	NM_032043.3:c.628-1969G>C		Unknown Significance	PP3 (SpliceAI; AG: 0.53 and DG: 0.56. Possibly pseudo-exon. RAW-score acceptor site 0.52 and donor site 0.57)		58	0	42	0	9	0
BRIP1	intron_variant	NM_032043.3:c.628-2761G>A		Unknown Significance	PP3 (SpliceAI; AG: 0.25 and DG: 0.19. Possibly pseudo-exon. RAW-score acceptor site 0.25 and donor site 0.43)		2	0	2	0	0	0
BRIP1	missense_variant	NM_032043.3:c.641G>C	NP_114432.2:p.Cys214Ser	Unknown Significance	No criteria met	SpliceAI AG: 0.20. Possibly partiel exon deletion. RAW score alternative splice site changes from 0.32 to 0.52 and natural splice site changes from 0.99 to 0.99	1	0	1	0	0	0
BRIP1	missense_variant	NM_032043.3:c.728T>C	NP_114432.2:p.Ile243Thr	Unknown Significance	BP4 (Revel=0.078 and BayesDel=-0.36)		1	0	1	0	0	0
BRIP1	missense_variant	NM_032043.3:c.774G>C	NP_114432.2:p.Gln258His	Unknown Significance	PM2_supporting (Only 1 in GnomAD)		2	0	1	0	0	0
BRIP1	missense_variant	NM_032043.3:c.790C>T	NP_114432.2:p.Arg264Trp	Unknown Significance	No criteria met		12	0	5	0	2	0
BRIP1	missense_variant	NM_032043.3:c.897G>A	NP_114432.2:p.Met299Ile	Unknown Significance	PS3 (In vitro assay showing a gain of function(PMID: 17145708 and PMID: 14983014))<br>PM2_supporting (Absent from GnomAD)		2	0	2	0	0	0
BRIP1	missense_variant	NM_032043.3:c.956T>C	NP_114432.2:p.Ile319Thr	Unknown Significance	PM2_supporting (Absent from GnomAD)		1	0	1	0	0	0
CDH1	missense_variant	NM_004360.5:c.1143G>T	NP_004351.1:p.Lys381Asn	Unknown Significance	PM2_supporting (Absent from GnomAD)		2	0	0	0	1	0
CDH1	missense_variant	NM_004360.5:c.1336G>A	NP_004351.1:p.Ala446Thr	Unknown Significance	PM2_supporting (Only 1 in GnomAD)		1	0	0	0	0	0
CDH1	missense_variant	NM_004360.5:c.1360G>A	NP_004351.1:p.Val454Ile	Unknown Significance	ccDK		2	0	2	0	0	0
CDH1	missense_variant	NM_004360.5:c.1417G>A	NP_004351.1:p.Val473Ile	Unknown Significance	ccDK		5	0	2	0	3	0
CDH1	intron_variant	NM_004360.5:c.1566-38T>A		Unknown Significance	PM2_supporting (GnomAD only 2)<br>PP3 (SpliceAI AG: 0.78. Possibly intron retention. RAW score alternative splice site 0.78 and natural acceptor changes from 0.99 to 0.96, MaxEntScan alternative splice site changes from 0 to 6.62 and SSF changes from 0 to 90.11)		1	0	0	0	0	0
CDH1	intron_variant	NM_004360.5:c.163+1035dupT		Unknown Significance	No criteria met		1	1	1	1	0	0
CDH1	intron_variant	NM_004360.5:c.163+1132G>A		Unknown Significance	PM2_supporting (Only 1 in GnomAD)		1	0	0	0	0	0
CDH1	missense_variant	NM_004360.5:c.1774G>T	NP_004351.1:p.Ala592Ser	Unknown Significance	ccDK		1	0	1	0	0	0
CDH1	missense_variant	NM_004360.5:c.1817A>T	NP_004351.1:p.Asn606Ile	Unknown Significance	PM2_supporting (GnomAD absent)		1	0	0	0	1	0
CDH1	missense_variant	NM_004360.5:c.1873C>T	NP_004351.1:p.Pro625Ser	Unknown Significance	PM2_supporting (GnomAD absent)		1	0	0	0	0	0
CDH1	missense_variant	NM_004360.5:c.1876T>G	NP_004351.1:p.Phe626Val	Unknown Significance	PM2_supporting (GnomAD 0.0000477111)		1	0	0	0	0	0
CDH1	missense_variant	NM_004360.5:c.1930G>A	NP_004351.1:p.Asp644Asn	Unknown Significance	PM2_supporting (GnomAD frequency 0.00000868)		2	0	2	0	0	0
CDH1	missense_variant	NM_004360.5:c.1939C>G	NP_004351.1:p.Gln647Glu	Unknown Significance	PM2_Supporting (Absent from GnomAD)		1	0	1	0	0	0
CDH1	missense_variant	NM_004360.5:c.2005C>T	NP_004351.1:p.Leu669Phe	Unknown Significance	ccDK		1	0	1	0	0	0
CDH1	missense_variant	NM_004360.5:c.2017C>A	NP_004351.1:p.Gln673Lys	Unknown Significance	No criteria met		1	0	0	0	0	0
CDH1	intron_variant	NM_004360.5:c.2165-1439G>A		Unknown Significance	PM2_supporting (Absent from GnomAD)		1	0	0	0	1	0
CDH1	missense_variant	NM_004360.5:c.2202A>T	NP_004351.1:p.Arg734Ser	Unknown Significance	No criteria met		2	0	1	0	1	0
CDH1	missense_variant	NM_004360.5:c.2307G>C	NP_004351.1:p.Leu769Phe	Unknown Significance	PM2_supporting (Absent from GnomAD)		1	0	0	0	0	0

CDH1	missense_variant	NM_004360.5:c.2335C>T	NP_004351.1:p.Arg779Trp	Unknown Significance	PM2_supporting (GnomAD frequency only 0.00000991)		2	0	2	0	0	0
CDH1	missense_variant	NM_004360.5:c.2350C>T	NP_004351.1:p.Arg784Cys	Unknown Significance	PM2_supporting (GnomAD less than 0.00001)		1	0	1	0	0	0
CDH1	missense_variant	NM_004360.5:c.2359G>T	NP_004351.1:p.Val787Phe	Unknown Significance	PM2_supporting (Absent from GnomAD)		1	0	1	0	0	0
CDH1	missense_variant	NM_004360.5:c.2363C>T	NP_004351.1:p.Ala788Val	Unknown Significance	ccDK		2	0	2	0	0	0
CDH1	missense_variant	NM_004360.5:c.2390A>G	NP_004351.1:p.Tyr797Cys	Unknown Significance	PM2_supporting (GnomAD frequency 0.00000041)		1	0	0	0	0	0
CDH1	intron_variant	NM_004360.5:c.2440-1128A>G		Unknown Significance	No criteria met		1	0	0	0	1	0
CDH1	intron_variant	NM_004360.5:c.2440-392G>C		Unknown Significance	PM2_supporting (Absent from GnomAD)		1	0	1	0	0	0
CDH1	splice_region_variant	NM_004360.5:c.2440-6_2440-4delCTT		Unknown Significance	ccDK		2	0	2	0	0	0
CDH1	missense_variant	NM_004360.5:c.2474C>T	NP_004351.1:p.Pro825Leu	Unknown Significance	ccDK		5	0	4	0	1	0
CDH1	missense_variant	NM_004360.5:c.2515G>A	NP_004351.1:p.Gly839Ser	Unknown Significance	No criteria met		1	0	0	0	0	0
CDH1	missense_variant	NM_004360.5:c.2561A>T	NP_004351.1:p.Asp854Val	Unknown Significance	PM2_supporting (Absent from GnomAD)		1	0	1	0	0	0
CDH1	missense_variant	NM_004360.5:c.2597G>T	NP_004351.1:p.Gly866Val	Unknown Significance	PM2_supporting (Absent from GnomAD)		1	0	1	0	0	0
CDH1	missense_variant	NM_004360.5:c.304G>T	NP_004351.1:p.Ala102Ser	Unknown Significance	PM2_supporting (GnomAD 0.000002736)		1	0	1	0	0	0
CDH1	splice_region_variant	NM_004360.5:c.388-8C>T		Unknown Significance	BP4 (SpliceAI=0.00. natural splice site MaxEntScan is 12.20 and does not change and SSF is 91.37 and changes to 92.98)		2	0	2	0	0	0
CDH1	missense_variant	NM_004360.5:c.455A>G	NP_004351.1:p.Gln152Arg	Unknown Significance	PM2_supporting (GnomAD 0.00000205216)		2	0	1	0	1	0
CDH1	intron_variant	NM_004360.5:c.48+109A>G		Unknown Significance	PM2_supporting (Absent from GnomAD)<br>PP3 (SpliceAI DG: 0.78. Possibly cryptic splice site or intron retention.RAW score donor 0.79. Besides that MaxEntScore 5.96, SSF 74.01 and NNSplice 0.61)		1	0	0	0	1	0
CDH1	intron_variant	NM_004360.5:c.48+188G>A		Unknown Significance	PM2_supporting (Only 1 in GnomAD)		2	0	1	0	0	0
CDH1	intron_variant	NM_004360.5:c.49-398A>T		Unknown Significance	No criteria met		1	0	1	0	0	0
CDH1	splice_region_variant	NM_004360.5:c.532-19dupT		Unknown Significance	PM2_supporting (Absent from GnomAD)<br>BP4 (SpliceAI=0.00, natural splice site MaxEntScore is 10.59 and does not changes and SSF is 90.52 and does not change)		1	0	1	0	0	0
CDH1	missense_variant	NM_004360.5:c.616A>T	NP_004351.1:p.Ile206Phe	Unknown Significance	PM2_supporting (Absent from GnomAD)		1	0	0	0	0	0
CDH1	missense_variant	NM_004360.5:c.937G>C	NP_004351.1:p.Asp313His	Unknown Significance	PM2_supporting (Absent from GnomAD)		2	0	1	0	0	0
CDH1	in-frame_insertion	NM_004360.5:c.984_986dupGGT	NP_004351.1:p.Val329dup	Unknown Significance	ccDK		1	0	0	0	0	0
CHEK2	missense_variant	NM_007194.4:c.1007A>T	NP_009125.1:p.Gln336Leu	Unknown Significance	PM2_supporting (Absent from GnomAD)		1	0	1	0	0	0
CHEK2	intron_variant	NM_007194.4:c.1009-144A>G		Unknown Significance	PP3 (SpliceAI AG: 0.38 and AL: 0.77. Possibly loss of one cryptic site and creation of new one. RAW score for loss of cryptic site changes from 0.77 to 0 and creating of new changes from 0.03 to 0.41)		2	0	0	0	0	0
CHEK2	intron_variant	NM_007194.4:c.1009-153T>C		Unknown Significance	PM2_supporting (GnomAD only 4)<br>PP3 (SpliceAI AG:0.32 and AL: 0.35. Possibly loss of cryptic splice site and creation of a new. RAW score loss of splice site changes from 0.77 to 0.43 and creation of new changes from 0.03 to 0.35)		1	0	0	0	0	0
CHEK2	missense_variant	NM_007194.4:c.1022A>C	NP_009125.1:p.Asn341Thr	Unknown Significance	No criteria met		5	0	1	0	1	0
CHEK2	missense_variant	NM_007194.4:c.1039G>A	NP_009125.1:p.Asp347Asn	Unknown significance	PS3 (Functional study showing damaging effect (PMID: 30851065))<br>PM1 (Located in conserved kinase domain)	No existing data on Odds ratio in ClinVar or HGMD, therefore downgraded to VUS	1	0	1	0	0	0
CHEK2	missense_variant	NM_007194.4:c.1067C>T	NP_009125.1:p.Ser356Leu	Unknown Significance	BP4 (Revel=0.214 and BayesDel=-0.0043)<br>BS3 (Functional study showing no damaing effect (PMID: 30851065))<br>PS3 (Functional study showing only intermediate effect (PMID: 31050813))		1	0	1	0	0	0
CHEK2	missense_variant	NM_007194.4:c.1091T>C	NP_009125.1:p.Ile364Thr	Unknown Significance	PS3 (Functional study showing damaging effect (PMID 31050813))<br>BS3 (Functional study showing no damaging effect (PMID: 30851065))		1	0	1	0	0	0
CHEK2	splice_region_variant	NM_007194.4:c.1096-4T>C		Unknown Significance	BP4 (SpliceAI=0.01)		1	0	0	0	0	0
CHEK2	splice_region_variant	NM_007194.4:c.1096-6T>G		Unknown Significance	PM2_supporting (Only 3 in GnomAD)<br>PP3 (SpliceAI AL: 0.54. AL: 0.49 and DL: 0.33. Possibly exon skipping or intron retention. RAW score natural donor changes from 0.96 to 0.63, natural acceptor changes from 0.97 to 0.43 and alternative acceptor changes from 0 to 0.49)		1	0	1	0	0	0
CHEK2	disruptive_in-frame_deletion	NM_007194.4:c.1109_1111delGGC	NP_009125.1:p.Gly370_His371delinsAsp	Unknown Significance	PM2_supporting (Absent from GnomAD)<br>PM4 (Protein length changes due to in-frame deletion in non-repeat region)		1	0	0	0	1	0
CHEK2	missense_variant	NM_007194.4:c.1111C>T	NP_009125.1:p.His371Tyr	Unknown Significance	BS3 (Functional study showing no damaing effect (PMID: 30851065))<br>PS3 (Functional study showing decrease i phosphorylation by 50% (PMID: 21618645))		1	0	0	0	0	0
CHEK2	missense_variant	NM_007194.4:c.1175C>T	NP_009125.1:p.Ala392Val	Unknown Significance	PP3 (Revel=0.825 and BayesDel=0.355)<br>PS3 (Functional study showing damaging effect (PMID: 30851065))		1	0	1	0	0	0
CHEK2	missense_variant	NM_007194.4:c.1183G>C	NP_009125.1:p.Val395Leu	Unknown Significance	BS3 (Functional study showing no damaging effekt (PMID: 30851065))<br>PS3 (Functional study showing deleterious effect (PMID: 31050813))		14	0	8	0	2	0
CHEK2	splice_region_variant	NM_007194.4:c.1260-6delA		Unknown Significance	PM2_supporting (Absent from GnomAD)<br>PP3 (SpliceAI AL: 0.25. Possibly loss of cryptic splice site. RAW score cryptic splice site changes from 0.86 to 0.61 and natural acceptor changes from 0.94 to 0.98)		1	0	1	0	0	0
CHEK2	missense_variant	NM_007194.4:c.1265G>A	NP_009125.1:p.Ser422Asn	Unknown Significance	BP4 (Revel=0.178 and BayesDel=-0.38)	SpliceAI AL: 0.31 and AG: 0.13. Possibly partiel exon deletion. RAW score natural acceptor changes from 0.94 to 0.65 and alternative acceptor changes from 0.86 to 0.99	3	0	2	0	0	0
CHEK2	missense_variant	NM_007194.4:c.1283C>T	NP_009125.1:p.Ser428Phe	Unknown significance	PS4_moderate (Described as a founder mutation in Ashkenazi Jewish with approximately 2 –fold increased risk of breast cancer (PMID: 15649950))<br>PS3 (Functional study in yeast incates abnormal CHEK2 activity (PMID: 22419737))<br>PP5 (Laboratories reported it pathogenic in ClinVar)	Literature search in ClinVar and HGMD showed 1 study with OR=2.13 (PMID: 15649950) and1 study with OR=0.695 (PMID: 33471991). The conservative approach was chosen and therefore downgraded to VUS	2	0	2	0	0	0
CHEK2	synonymous_variant	NM_007194.4:c.1287G>A	NP_009125.1:p.Glu429=	Unknown Significance	BP6 (Laboratories reported it benign in ClinVar)		1	0	0	0	1	0
CHEK2	missense_variant	NM_007194.4:c.1307T>G	NP_009125.1:p.Leu436Arg	Unknown Significance	PP3 (Revel=0.791 and BayesDel=0.304)<br>PM2_supporting (Only 1 in GnomAD)		1	0	1	0	0	0
CHEK2	missense_variant	NM_007194.4:c.1312G>T	NP_009125.1:p.Asp438Tyr	Unknown Significance	BS3 (Functional study showing no damaging effekt (PMID: 30851065))<br>PS3 (Functional study showing 70% reduction in activity (PMID: 17721994))		3	0	2	0	0	0
CHEK2	missense_variant	NM_007194.4:c.1383C>G	NP_009125.1:p.Asp461Glu	Unknown Significance	BP4 (Revel=0.226 and BayesDel=-0.1354)		5	0	4	0	1	0
CHEK2	missense_variant	NM_007194.4:c.1421G>A	NP_009125.1:p.Arg474His	Unknown significance	PS3 (Functinel study showing ex. decreased kinase activity (PMID: 31050813))<br>PP3 (Revel=0.867 and BayesDel=0.39)<br>PM2_supporting (Absent from GnomAD v.2.1)	No existing data on Odds ratio in ClinVar or HGMD, therefore downgraded to VUS	3	0	2	0	0	0
CHEK2	missense_variant	NM_007194.4:c.1421G>C	NP_009125.1:p.Arg474Pro	Unknown Significance	PP3 (Revel=0.884 and BayesDel=0.39)<br>PM2_supporting (Absent from GnomAD)		1	0	0	0	0	0
CHEK2	missense_variant	NM_007194.4:c.1427C>T	NP_009125.1:p.Thr476Met	Unknown Significance	PS3 (Two functional studies showing a deleterious effect based on respectively kinase activity and DNA damage (PMID: 22114986 and PMID: 22419737))<br>BS3 (Functional study showing a neutral effect (PMID: 31050813))		30	0	22	0	2	0
CHEK2	missense_variant	NM_007194.4:c.1450C>A	NP_009125.1:p.Pro484Thr	Unknown Significance	BS3 (Functional study showing no damaing effect (PMID: 30851065))		2	0	1	0	0	0
CHEK2	splice_region_variant	NM_007194.4:c.1461+5G>T		Unknown Significance	PM2_supporting (Only 1 in GnomAD)<br>PP3 (SpliceAI AL: 0.68 and DL: 0.90. Possibly exon-skipping. RAW score acceptor changes from 0.99 to 0.31 and donor changes from 0.99 to 0.09)		2	0	1	0	0	0
CHEK2	intron_variant	NM_007194.4:c.1462-1829A>G		Unknown Significance	PM2_supporting (Only 3 in GnomAD)<br>PP3 (SpliceAI DG: 0.22. Possibly cryptic splice site. RAW score 0.22. No nearby acceptor site)		2	0	2	0	0	0
CHEK2	missense_variant	NM_007194.4:c.1522C>G	NP_009125.1:p.Leu508Val	Unknown Significance	BP4 (Revel=0.071 and BayesDel=-0.46)		3	0	3	0	0	0
CHEK2	missense_variant	NM_007194.4:c.153G>C	NP_009125.1:p.Gln51His	Unknown Significance	BP4 (Revel=0.137 and BayesDel=0.02099)<br>PM2_supporting (Absent from GnomAD)		2	0	0	0	1	0
CHEK2	missense_variant	NM_007194.4:c.190G>A	NP_009125.1:p.Glu64Lys	Unknown Significance	PS3 (Multiple functional studies showing damaging effekt (PMID: 16835864, PMID: 31050813 and PMID: 22419737))<br>BS3 (Functional study showing a benign effect (PMID: 30851065))		15	0	8	0	1	0
CHEK2	in-frame_deletion	NM_007194.4:c.246_260delCCAAGAACCTGAGGA	NP_009125.1:p.Asp82_Glu86del	Unknown Significance	PM4 (Protein length changes as a result of in-frame deletion)<br>PS3_moderate (Mutants exhibited 40–50% of wild-type activity (PMID: 17721994))		1	0	1	0	0	0
CHEK2	missense_variant	NM_007194.4:c.323G>C	NP_009125.1:p.Cys108Ser	Unknown Significance	PP3 (Revel=0.799 and BayesDel=0.376)<br>PM2_supporting (Absent from GnomAD)		1	0	1	0	0	0
CHEK2	missense_variant	NM_007194.4:c.332A>G	NP_009125.1:p.Asp111Gly	Unknown Significance	PM2_supporting (Absent from GnomAD)		1	0	1	0	0	0
CHEK2	missense_variant	NM_007194.4:c.433C>T	NP_009125.1:p.Arg145Trp	Unknown significance	PS3 (Multiple functional assay showing damaging effekt (PMID: 11298456. PMID: 12049740 and PMID: 30851065))<br>PP3 (Revel=0.812 and BayesDel=0.403708)<br>PP1 (3 affected family members with the variant (PMID: 22419737))	Literature search in ClinVar and HGMD showed 1 study with OR=1.965 (PMID: 33471991) and therefore downgraded to VUS	6	0	6	0	1	0
CHEK2	missense_variant	NM_007194.4:c.434G>A	NP_009125.1:p.Arg145Gln	Unknown Significance	BS3 (Functional study showing no damaging effekt (PMID: 30851065))		1	0	1	0	0	0

CHEK2	missense_variant	NM_007194.4:c.442A>G	NP_009125.1:p.Arg148Gly	Unknown significance	PS3 (Functional study showing damaging effekt (PMID: 30851065))<br>PP3 (Revel=0.85 and BayesDel=0.39)<br>PM2_supporting (Only 3 in GnomAD)	No existing data on Odds ratio in ClinVar or HGMD, therefore downgraded to VUS	1	0	0	0	1	0
CHEK2	splice_region_variant	NM_007194.4:c.445-7A>C		Unknown Significance	BP4 (SpliceAI=0.07)<br>PM2_supporting (Absent from GnomAD)		1	0	1	0	0	0
CHEK2	missense_variant	NM_007194.4:c.451G>T	NP_009125.1:p.Gly151Cys	Unknown Significance	PM2_supporting (Only 1 in GnomAD)<br>PP3 (Revel=0.799 and BayesDel=0.32)<br>BS3 (Functional study showing a benign characterization (PMID: 30851065))		1	0	0	0	0	0
CHEK2	missense_variant	NM_007194.4:c.470T>C	NP_009125.1:p.Ile157Thr	Unknown significance	PS3 (Multiple functional studies showing damaging effekt (PMID: 22419737 and PMID: 11298456))<br>PP5 (laboratories reported it pathogenic in ClinVar)<br>PP1 (5 affected family members with the variant (PMID: 22419737))	Literature search in ClinVar and HGMD showed 3 studies with OR from 1.21 to 1.6 (PMID: 36136322, 33471974, 21514219) and therefore downgraded to VUS	51	0	32	0	10	0
CHEK2	missense_variant	NM_007194.4:c.478A>G	NP_009125.1:p.Ile160Val	Unknown Significance	No criteria met		1	0	1	0	0	0
CHEK2	missense_variant	NM_007194.4:c.524T>C	NP_009125.1:p.Val175Ala	Unknown Significance	PM2_supporting (Absent from GnomAD)		3	0	1	0	1	0
CHEK2	missense_variant	NM_007194.4:c.539G>A	NP_009125.1:p.Arg180His	Unknown Significance	BS3 (Functional study classified variant as neutral according to phosphorylation (PMID: 31050813))<br>PS3_moderate(Functional study showing reduced stability and reduced protein expression (PMID: 16982735))		5	0	4	0	0	0
CHEK2	missense_variant	NM_007194.4:c.541C>T	NP_009125.1:p.Arg181Cys	Unknown Significance	PS3 (Functional study showing a reduced kinase activity (PMID: 16835864))<br>BS3 (Functional study showing no damaing effect (PMID: 30851065))		1	0	1	0	0	0
CHEK2	missense_variant	NM_007194.4:c.542G>A	NP_009125.1:p.Arg181His	Unknown Significance	BS3 (Functional study showing no damaing effect (PMID: 30851065))		2	0	1	0	0	0
CHEK2	intron_variant	NM_007194.4:c.592+607G>A		Unknown Significance	PM2_supporting (Only 1 in GnomAD)		1	0	1	0	0	0
CHEK2	missense_variant	NM_007194.4:c.604T>C	NP_009125.1:p.Phe202Leu	Unknown Significance	PM2_supporting (Only 2 in GnomAD)		6	0	5	0	1	0
CHEK2	synonymous_variant	NM_007194.4:c.612G>T	NP_009125.1:p.Leu204=	Unknown Significance	PM2_supporting (Only 2 in GnomAD)<br>PP3 (SpliceAI AG: 0.33 and AL: 0.21.Possibly partiel exon deletion. RAW score natural acceptor changes from 0.94 to 0.73 and alternative acceptor changes from 0.41 to 0.74)		1	0	1	0	0	0
CHEK2	missense_variant	NM_007194.4:c.683G>A	NP_009125.1:p.Ser228Asn	Unknown Significance	BP4 (Revel=0.135 and BayesDel=-0.212)<br>PM2_supporting (Absent from GnomAD)	SpliceAI AL:0.92 and DL: 0.96. Exon skipping. RAW score donor changes from 0.96 to 0 and acceptor changes from 0.94 to 0.02	1	0	1	0	0	0
CHEK2	intron_variant	NM_007194.4:c.684-86A>G		Unknown Significance	No criteria met		17	0	9	0	3	0
CHEK2	missense_variant	NM_007194.4:c.715G>A	NP_009125.1:p.Glu239Lys	Unknown Significance	PS3 (Functional study showing reduced kinase activity (PMID: 16835864))<br>BS3 (Functional study showing no damaing effect (PMID: 30851065))<br>PP1 (3 affected family members with the variant (PMID: 33692755))		9	0	6	0	2	0
CHEK2	missense_variant	NM_007194.4:c.839T>G	NP_009125.1:p.Leu280Arg	Unknown Significance	PM2_supporting (Only 1 in GnomAD)		3	0	3	0	0	0
CHEK2	splice_region_variant	NM_007194.4:c.847-17T>C		Unknown Significance	BP6 (Laboratories reported it benign in ClinVar)		1	0	1	0	0	0
CHEK2	missense_variant	NM_007194.4:c.851G>A	NP_009125.1:p.Cys284Tyr	Unknown Significance	PS3 (Functional study showing that the variant has LOF behavior (PMID: 33606978))<br>PM2_supporting (GnomAD only 2)		2	0	1	0	0	0
CHEK2	missense_variant	NM_007194.4:c.855C>G	NP_009125.1:p.Ile285Met	Unknown Significance	PS3 (Functional study showing that it is functional the same as wildtype, when assesing KAP1 phosphorylation and CHEK2 autophosphorylation (PMID: 37449874))		1	0	1	0	0	0
CHEK2	missense_variant	NM_007194.4:c.8G>T	NP_009125.1:p.Arg3Leu	Unknown Significance	No criteria met		1	0	1	0	0	0
CHEK2	missense_variant	NM_007194.4:c.980A>G	NP_009125.1:p.Tyr327Cys	Unknown Significance	BS3 (Functional study showing no damaing effect (PMID: 30851065))<br>PS3 (Functional study showing damaing effect (PMID: 31050813))		1	0	0	0	0	0
CHEK2	missense_variant	NM_007194.4:c.991A>G	NP_009125.1:p.Met331Val	Unknown Significance	PM2_supporting (Only 1 in GnomAD)		1	0	1	0	0	0
PALB2	missense_variant	NM_024675.4:c.1427G>T	NP_078951.2:p.Arg476Ile	Unknown Significance	ccDK		3	0	0	0	0	0
PALB2	splice_region_variant	NM_024675.4:c.1685-3T>C		Unknown Significance	ccDK		1	0	1	0	0	0
PALB2	missense_variant	NM_024675.4:c.1960A>G	NP_078951.2:p.Ile654Val	Unknown Significance	BP1 (Missense variant)		1	0	0	0	0	0
PALB2	missense_variant	NM_024675.4:c.2272C>A	NP_078951.2:p.Pro758Thr	Unknown Significance	ccDK		1	0	1	0	0	0
PALB2	missense_variant	NM_024675.4:c.2456A>G	NP_078951.2:p.Lys819Arg	Unknown Significance	ccDK		1	0	1	0	0	0
PALB2	missense_variant	NM_024675.4:c.2564T>C	NP_078951.2:p.Leu855Pro	Unknown Significance	ccDK		3	0	1	0	1	0
PALB2	missense_variant	NM_024675.4:c.2755G>A	NP_078951.2:p.Val919Ile	Unknown Significance	ccDK		1	0	1	0	0	0
PALB2	splice_region_variant	NM_024675.4:c.2835-11A>G		Unknown Significance	ccDK		2	0	2	0	0	0
PALB2	splice_region_variant	NM_024675.4:c.2835-3T>C		Unknown Significance	ccDK		1	0	0	0	1	0
PALB2	missense_variant	NM_024675.4:c.2897T>C	NP_078951.2:p.Ile966Thr	Unknown Significance	ccDK		1	0	1	0	0	0
PALB2	missense_variant	NM_024675.4:c.3054G>T	NP_078951.2:p.Glu1018Asp	Unknown Significance	ccDK		2	0	1	0	0	0
PALB2	missense_variant	NM_024675.4:c.3055G>T	NP_078951.2:p.Val1019Phe	Unknown Significance	ccDK		1	0	0	0	1	0
PALB2	splice_region_variant	NM_024675.4:c.3114-4T>A		Unknown Significance	PM2_supporting (Absent from GnomAD)<br>BP4 (SpliceAI=0.03)		1	0	0	0	0	0
PALB2	missense_variant	NM_024675.4:c.3146T>C	NP_078951.2:p.Met1049Thr	Unknown Significance	ccDK		1	0	1	0	0	0
PALB2	in-frame_deletion	NM_024675.4:c.3157_3159delGAT	NP_078951.2:p.Asp1053del	Unknown Significance	PP3 (SpliceAI DL:0.21 and AL: 0.17. Possibly exon skipping. RAW score donor changes from 0.96 to 0.75 and acceptor changes from 0.94 to 0.78)<br>PM2_supporting (Only 1 in GnomAD)		1	0	1	0	0	0
PALB2	missense_variant	NM_024675.4:c.3203G>A	NP_078951.2:p.Gly1068Glu	Unknown Significance	BP1 (Missense variant)		1	0	1	0	0	0
PALB2	missense_variant	NM_024675.4:c.3350G>A	NP_078951.2:p.Arg1117Lys	Unknown Significance	ccDK		1	0	1	0	0	0
PALB2	splice_region_variant	NM_024675.4:c.3351-6T>C		Unknown Significance	ccDK		2	0	1	0	0	0
PALB2	missense_variant	NM_024675.4:c.3508C>T	NP_078951.2:p.His1170Tyr	Unknown Significance	ccDK		1	0	1	0	0	0
PALB2	missense_variant	NM_024675.4:c.3518C>T	NP_078951.2:p.Ala1173Val	Unknown Significance	ccDK		1	0	1	0	0	0
PALB2	intron_variant	NM_024675.4:c.48+758A>T		Unknown Significance	PM2_supporting (Absent from GnomAD)		1	0	1	0	0	0
PALB2	missense_variant	NM_024675.4:c.632A>G	NP_078951.2:p.Glu211Gly	Unknown Significance	ccDK		4	0	4	0	0	0
PALB2	missense_variant	NM_024675.4:c.656A>C	NP_078951.2:p.Asp219Ala	Unknown Significance	ccDK		1	0	1	0	0	0
PALB2	missense_variant	NM_024675.4:c.766A>G	NP_078951.2:p.Ser256Gly	Unknown Significance	ccDK		1	0	1	0	0	0
PTEN	missense_variant	NM_000314.8:c.1061C>A	NP_000305.3:p.Pro354Gln	Unknown Significance	ccDK		3	0	2	0	0	0
PTEN	intron_variant	NM_000314.8:c.165-15339G>A		Unknown Significance	PM2_supporting (Only 1 in GnomAD)		1	0	1	0	0	0
PTEN	intron_variant	NM_000314.8:c.209+2047A>G		Unknown Significance	PM2_supporting (Absent from GnomAD)<br>PP3(SpliceAI AG:0.56 and DG:0.53. Possibly pseudo-exon. RAW score alternative acceptor changes from 0.33 to 0.88 and alternative donor changes from 0.23 to 0.76)		1	0	1	0	0	0
PTEN	missense_variant	NM_000314.8:c.450G>T	NP_000305.3:p.Glu150Asp	Unknown Significance	PM2_supporting (Absent from GnomAD)<br>PP2 (Missense variant)<br>BS3_supporting(Phosphatase activity 0.42 (PMID: 29706350))		1	0	1	0	0	0
PTEN	intron_variant	NM_000314.8:c.492+2756G>T		Unknown Significance	PM2_supporting (Absent from GnomAD)		2	0	0	0	2	0
PTEN	missense_variant	NM_000314.8:c.700C>T	NP_000305.3:p.Arg234Trp	Unknown Significance	PP2 (Missense variant)<br>BS3_supporting (Functional study showing phosphatase activity 0.10 (PMID:29706350))		1	0	1	0	0	0
PTEN	intron_variant	NM_000314.8:c.79+10891delC		Unknown Significance	PM2_supporting (Absent from GnomAD)		1	0	1	0	0	0
PTEN	missense_variant	NM_000314.8:c.956C>T	NP_000305.3:p.Thr319Ile	Unknown Significance	PM2_supporting (GnomAD only 2)<br>PP3 (Revel=0.745)		1	0	1	0	0	0
RAD51C	frameshift_variant	NM_058216.3:c.1025_1026delAA	NP_478123.1:p.Lys342Thrfs*5	Unknown Significance	PVS1_strong (Frameshift variant, not predicted to undergo NMD, but important to protein function)<br>PM2_supporting (Only 1 in GnomAD)	SpliceAI AL: 0.52 and DL: 0.39. Possibly exon skipping. RAW score donor changes from 0.88 to 0.49 and acceptor changes from 0.84 to 0.32	1	0	1	0	0	0
RAD51C	splice_region_variant	NM_058216.3:c.1026+6T>C		Unknown Significance	PP3 (SpliceAI AL: 0.73 and DL: 0.84. Possibly exon-skipping. RAW score acceptor changes from 0.84 to 0.12 and donor changes from 0.87 to 0.03)<br>PM2_supporting (Absent from GnomAD)		1	0	0	0	1	0
RAD51C	splice_region_variant	NM_058216.3:c.145+3A>G		Unknown Significance	PP3 (SpliceAI DL: 0.63. Possibly partiel exon deletion. RAW score naturel donor site changes from 0.86 to 0.24 and alternative donor site changes from 0.83 to 0.90)<br>PM2_supporting (Only 1 in GnomAD)		1	0	0	0	0	0
RAD51C	intron_variant	NM_058216.3:c.145+93C>G		Unknown Significance	PP3 (SpliceAI DG: 0.32. Possibly intron retention. RAW score alternative donor changes from 0 to 0.33 and natural donor changes from 0.86 to 0.86)		1	0	1	0	0	0
RAD51C	missense_variant	NM_058216.3:c.158C>G	NP_478123.1:p.Ser53Cys	Unknown Significance	PM2_supporting (Only 5 in GnomAD)<br>BS3 (HDR assay shows neutral effect (PMID: 37253112)		1	0	0	0	1	0
RAD51C	splice_region_variant	NM_058216.3:c.405-8G>A		Unknown Significance	BP4 (SpliceAI=0.00)<br>PM2_supporting (Absent from GnomAD)		1	0	1	0	0	0
RAD51C	missense_variant	NM_058216.3:c.422A>G	NP_478123.1:p.Asp141Gly	Unknown Significance	PM2_supporting (Absent from GnomAD)		1	0	1	0	0	0

RAD51C	missense_variant	NM_058216.3:c.425T>C	NP_478123.1:p.Val142Ala	Unknown Significance	PM2_supporting (Absent from GnomAD)<br>BP4 (Revel=0.206 and BayesDel=-0.295)		1	0	1	0	0	0
RAD51C	missense_variant	NM_058216.3:c.428A>G	NP_478123.1:p.Gln143Arg	Unknown Significance	PS3 (Functional study showing RAD51C-deficient. It was not able to restore RAD51 foci formation (PMID: 22451500) and another functional study showing reduced HR frequency (PMID: 25292178))		1	0	1	0	0	0
RAD51C	missense_variant	NM_058216.3:c.479C>G	NP_478123.1:p.Thr160Arg	Unknown Significance	PM2_supporting (Absent from GnomAD)		1	0	0	0	1	0
RAD51C	missense_variant	NM_058216.3:c.482A>T	NP_478123.1:p.Glu161Val	Unknown Significance	PP3 (Revel=score 0.892 and BayesDel=0.34)<br>PM2_supporting (Only 5 in GnomAD)		1	0	1	0	0	0
RAD51C	missense_variant	NM_058216.3:c.523G>A	NP_478123.1:p.Ala175Thr	Unknown Significance	BS3 (Functional study showed remained neutral in HDR assay (PMID: 37253112))		1	0	0	0	0	0
RAD51C	missense_variant	NM_058216.3:c.578G>A	NP_478123.1:p.Arg193Gln	Unknown Significance	BP4 (Revel=0.069 and BayesDel=-0.484)		1	0	1	0	0	0
RAD51C	missense_variant	NM_058216.3:c.61C>T	NP_478123.1:p.Pro21Ser	Unknown Significance	PM2_supporting (Only 1 in GnomAD)<br>BP4 (Revel=0.128 and BayesDel=-0.477)		2	0	1	0	0	0
RAD51C	intron_variant	NM_058216.3:c.705+3152C>T		Unknown Significance	PM2_supporting (Only 3 in GnomAD)<br>PP3 (SpliceAI AG: 0.46 and DG: 0.34. Possibly pseudo exon. RAW score acceptor 0.84 and donor 0.82)		2	0	1	0	1	0
RAD51C	intron_variant	NM_058216.3:c.706-926G>T		Unknown Significance	No criteria met		4	0	4	0	0	0
RAD51C	missense_variant	NM_058216.3:c.784T>G	NP_478123.1:p.Leu262Val	Unknown Significance	BS3 (Functional study showing neutral effect, but also SpliceAI=0.7 (PMID: 37253112))	SpliceAI DG: 0.71. Possibly partiel exon deletion. RAW score alternative donor site changes from 0 to 0.71 and naturel donor site changes from 0.99 to 0.98	5	0	3	0	1	0
RAD51C	intron_variant	NM_058216.3:c.837+621A>G		Unknown Significance	No criteria met		8	1	6	1	2	0
RAD51C	intron_variant	NM_058216.3:c.837+731A>G		Unknown Significance	PP3 (SpliceAI AG: 0.23 and DG: 0.66. Possibly pseudo exon. RAW score acceptor 0.87 and donor 0.73)<br>PM2_supporting (Absent from GnomAD)		1	0	1	0	0	0
RAD51C	intron_variant	NM_058216.3:c.905-1082G>A		Unknown Significance	PM2_supporting (Only 2 in GnomAD)		2	0	0	0	0	0
RAD51C	missense_variant	NM_058216.3:c.952G>A	NP_478123.1:p.Asp318Asn	Unknown Significance	ccDK		4	0	2	0	0	0
RAD51C	intron_variant	NM_058216.3:c.966-2249G>A		Unknown Significance	PP3 (SpliceAI AG: 0.20 and DG: 0.16. Possibly pseudo-exon. RAW score acceptor 0.40 and donor 0.36)<br>PM2_supporting (Only 1 in GnomAD)		2	0	2	0	0	0
RAD51C	intron_variant	NM_058216.3:c.966-2263G>A		Unknown Significance	PM2_supporting (Absent from GnomAD)		2	0	2	0	0	0
RAD51D	missense_variant	NM_002878.4:c.131G>A	NP_002869.3:p.Gly44Asp	Unknown Significance	BP4 (Revel=0.115 and BayesDel=-0.2356)		2	0	1	0	0	0
RAD51D	missense_variant	NM_002878.4:c.167T>G	NP_002869.3:p.Val56Gly	Unknown Significance	No criteria met		1	0	1	0	1	0
RAD51D	missense_variant	NM_002878.4:c.180G>T	NP_002869.3:p.Gln60His	Unknown Significance	PM2_supporting (Only 2 in GnomAD)<br>BP4 (Revel=0.204 and BayesDel=-0.148)		1	0	1	0	0	0
RAD51D	initiator_codon_variant	NM_002878.4:c.1A>T	NP_002869.3:p.Met1Leu	Unknown Significance	ccDK		1	0	0	0	0	0
RAD51D	missense_variant	NM_002878.4:c.26G>C	NP_002869.3:p.Cys9Ser	Unknown Significance	ccDK		4	0	3	0	1	0
RAD51D	missense_variant	NM_002878.4:c.316G>A	NP_002869.3:p.Val106Ile	Unknown Significance	PM2_supporting (Absent from GnomAD)<br>BP4 (Revel=0.066 and BayesDel=-0.19)		1	0	1	0	0	0
RAD51D	missense_variant	NM_002878.4:c.325C>T	NP_002869.3:p.Pro109Ser	Unknown Significance	PM2_supporting (Absent from GnomAD)<br>BP4 (Revel=0.209 and BayesDel=-0.052)		1	0	1	0	0	0
RAD51D	synonymous_variant	NM_002878.4:c.333C>T	NP_002869.3:p.Ser111=	Unknown Significance	PP3 (SpliceAI DL:0.24 and AL:0.17. Possibly exon-skipping. RAW score acceptor changes from 0.87 to 0.70 and donor changes from 0.92 to 0.68)		1	0	1	0	0	0
RAD51D	missense_variant	NM_002878.4:c.334G>A	NP_002869.3:p.Gly112Ser	Unknown Significance	PP3 (Revel=0.94 and BayesDel=0.52)		2	0	1	0	0	0
RAD51D	missense_variant	NM_002878.4:c.355T>C	NP_002869.3:p.Cys119Arg	Unknown Significance	BP4 (Revel=0.219 and BayesDel=-0.01757)		5	0	5	0	1	0
RAD51D	missense_variant	NM_002878.4:c.412A>G	NP_002869.3:p.Asn138Asp	Unknown Significance	ccDK		3	0	1	0	1	0
RAD51D	missense_variant	NM_002878.4:c.422T>C	NP_002869.3:p.Leu141Pro	Unknown Significance	No criteria met		1	0	0	0	1	0
RAD51D	missense_variant	NM_002878.4:c.433C>G	NP_002869.3:p.Arg145Gly	Unknown Significance	PM2_supporting (Absent from GnomAD)		1	0	1	0	0	0
RAD51D	missense_variant	NM_002878.4:c.433C>T	NP_002869.3:p.Arg145Cys	Unknown Significance	No criteria met		1	0	1	0	0	0
RAD51D	missense_variant	NM_002878.4:c.493C>T	NP_002869.3:p.Arg165Trp	Unknown Significance	No criteria met		1	0	1	0	0	0
RAD51D	missense_variant	NM_002878.4:c.712G>T	NP_002869.3:p.Ala238Ser	Unknown Significance	PM2_supporting (Absent from GnomAD)		1	0	0	0	1	0
RAD51D	missense_variant	NM_002878.4:c.715C>T	NP_002869.3:p.Arg239Trp	Unknown Significance	No criteria met		1	0	1	0	0	0
RAD51D	missense_variant	NM_002878.4:c.716G>A	NP_002869.3:p.Arg239Gln	Unknown Significance	ccDK		1	0	0	0	0	0
RAD51D	missense_variant	NM_002878.4:c.758G>A	NP_002869.3:p.Arg253Gln	Unknown Significance	No criteria met		2	0	0	0	1	0
RAD51D	intron_variant	NM_002878.4:c.82+152C>T		Unknown Significance	No criteria met		10	0	7	0	3	0
RAD51D	missense_variant	NM_002878.4:c.824G>A	NP_002869.3:p.Arg275Gln	Unknown Significance	No criteria met		1	0	1	0	0	0
RAD51D	missense_variant	NM_002878.4:c.872G>A	NP_002869.3:p.Arg291His	Unknown Significance	ccDK		7	0	3	0	2	0
RAD51D	missense_variant	NM_002878.4:c.878C>T	NP_002869.3:p.Ala293Val	Unknown Significance	BP4 (Revel=0.174 and BayesDel=-0.594)		1	0	0	0	0	0
RAD51D	missense_variant	NM_002878.4:c.899G>A	NP_002869.3:p.Arg300Gln	Unknown Significance	No criteria met		1	0	1	0	0	0
RAD51D	splice_region_variant	NM_002878.4:c.904-3C>T		Unknown Significance	BP4 (SpliceAI=0.07)		4	0	1	0	2	0
RAD51D	missense_variant	NM_002878.4:c.932T>A	NP_002869.3:p.Ile311Asn	Unknown Significance	No criteria met		2	0	2	0	0	0
STK11	intron_variant	NM_000455.5:c.*17-365G>A		Unknown Significance	No criteria met		4	0	2	0	1	0
STK11	missense_variant	NM_000455.5:c.1036G>A	NP_000446.1:p.Gly346Ser	Unknown Significance	ccDK		1	0	1	0	0	0
STK11	missense_variant	NM_000455.5:c.1077C>G	NP_000446.1:p.Asp359Glu	Unknown Significance	ccDK		1	0	1	0	1	0
STK11	intron_variant	NM_000455.5:c.1109-558G>A		Unknown Significance	No criteria met		18	0	9	0	2	0
STK11	intron_variant	NM_000455.5:c.1109-923A>C		Unknown Significance	PM2_supporting (Only 1 in GnomAD)		1	0	0	0	0	0
STK11	missense_variant	NM_000455.5:c.113C>T	NP_000446.1:p.Pro38Leu	Unknown Significance	PM2_supporting (Only 1 in GnomAD)		1	0	0	0	0	0
STK11	missense_variant	NM_000455.5:c.1151G>A	NP_000446.1:p.Arg384Gln	Unknown Significance	ccDK		3	0	2	0	0	0
STK11	missense_variant	NM_000455.5:c.1211C>T	NP_000446.1:p.Ser404Phe	Unknown Significance	ccDK		3	0	3	0	0	0
STK11	missense_variant	NM_000455.5:c.1220A>C	NP_000446.1:p.Glu407Ala	Unknown Significance	PM2_supporting (GnomAD only 1)<br>BP4 (Revel=0.235 and BayesDel=-0.21)		2	0	0	0	0	0
STK11	missense_variant	NM_000455.5:c.1225C>T	NP_000446.1:p.Arg409Trp	Unknown Significance	ccDK		1	0	0	0	0	0
STK11	missense_variant	NM_000455.5:c.1244G>A	NP_000446.1:p.Arg415His	Unknown Significance	ccDK		1	0	0	0	1	0
STK11	missense_variant	NM_000455.5:c.124C>G	NP_000446.1:p.Arg42Gly	Unknown Significance	ccDK		1	0	1	0	0	0
STK11	missense_variant	NM_000455.5:c.1274G>A	NP_000446.1:p.Arg425His	Unknown Significance	PM2_supporting (Absent from GnomAD)		1	0	1	0	0	0
STK11	synonymous_variant	NM_000455.5:c.1301G>A	NP_000446.1:p.Ter434=	Unknown Significance	PM2_supporting (Only 1 in GnomAD)<br>PP3 (SpliceAI DG: 0.73. Possibly partiel exon skipping. RAW score alternative donor site changes from 0.12 to 0.86 and natural donor site changes from 0.98 to 0.86)		1	0	1	0	0	0
STK11	missense_variant	NM_000455.5:c.139G>A	NP_000446.1:p.Gly47Ser	Unknown Significance	PM2_supporting (Only 1 in GnomAD)		1	0	1	0	0	0
STK11	intron_variant	NM_000455.5:c.290+3229G>A		Unknown Significance	PM2_supporting (Absent from GnomAD)		1	0	0	0	1	0
STK11	intron_variant	NM_000455.5:c.291-1237G>A		Unknown Significance	PP3 (SpliceAI DG: 0.21. Possibly cryptic splice site, no nearby acceptor site. RAW score changes from 0.01 to 0.21)		1	0	1	0	0	0
STK11	intron_variant	NM_000455.5:c.291-2759G>A		Unknown Significance	PM2_supporting (Only 5 in GnomAD)		1	0	0	0	0	0
STK11	missense_variant	NM_000455.5:c.566C>T	NP_000446.1:p.Thr189Ile	Unknown Significance	ccDK		1	0	0	0	0	0
STK11	synonymous_variant	NM_000455.5:c.618G>A	NP_000446.1:p.Ala206=	Unknown Significance	PP3 (SpliceAI AG: 0.58. Possibly partiel exon deletion. RAW score alternative acceptive site changes from 0 to 0.58 and naturel acceptor site changes from 0.99 to 0.98)		2	0	2	0	0	0
STK11	missense_variant	NM_000455.5:c.631C>T	NP_000446.1:p.Arg211Trp	Unknown Significance	ccDK		1	0	0	0	1	0
STK11	missense_variant	NM_000455.5:c.632G>A	NP_000446.1:p.Arg211Gln	Unknown Significance	BP4 (Revel=0.215 and BayesDel=0.0172)		1	0	1	0	0	0
STK11	missense_variant	NM_000455.5:c.721G>A	NP_000446.1:p.Ala241Thr	Unknown Significance	No criteria met		1	0	1	0	0	0
STK11	intron_variant	NM_000455.5:c.863-268C>T		Unknown Significance	PP3 (SpliceAI AG: 0.27. Possibly cryptic splite site. RAW score acceptor changes from 0.19 to 0.46. No donor site nearby)		1	0	1	0	0	0
STK11	intron_variant	NM_000455.5:c.863-272C>T		Unknown Significance	No criteria met		3	0	2	0	0	0
STK11	intron_variant	NM_000455.5:c.863-296C>T		Unknown Significance	PP3 (SpliceAI AG: 0.22. Possibly cryptic splite site. RAW score acceptor changes from 0.63 to 0.72. No donor site nearby)		4	0	3	0	0	0
STK11	missense_variant	NM_000455.5:c.875A>T	NP_000446.1:p.Tyr292Phe	Unknown Significance	No criteria met		1	0	1	0	0	0
STK11	intron_variant	NM_000455.5:c.920+458C>G		Unknown Significance	PM2_supporting (Absent from GnomAD)<br>PP3 (SpliceAI AG: 0.24. Possibly cryptic splite site. RAW score acceptor changes from not existing to 0.24. No donor site nearby)		1	0	0	0	1	0
STK11	intron_variant	NM_000455.5:c.921-161G>C		Unknown Significance	PM2_supporting (Only 1 in GnomAD)<br>PP3 (SpliceAI AG: 0.63. Possibly intron retention. RAW score acceptor changes from not existing to 0.63. Naturel acceptor site changes from 0.997 to 0.996)		1	0	0	0	0	0
STK11	intron_variant	NM_000455.5:c.921-419C>T		Unknown Significance	PP3 (SpliceAI DG: 0.40. Possibly cryptic splite site. RAW score donor changes from not existing to 0.40. No acceptor site nearby)		73	5	49	4	11	1
STK11	intron_variant	NM_000455.5:c.921-66T>C		Unknown Significance	No criteria met		1	0	1	0	0	0

STK11	intron_variant	NM_000455.5:c.921-87G>A		Unknown Significance	PM2_supporting (Absent from GnomAD)		1	0	0	0	0	0
STK11	missense_variant	NM_000455.5:c.944C>T	NP_000446.1:p.Pro315Leu	Unknown Significance	BP4 (Revel=0.09 and BayesDel=-0.44)		2	0	1	0	0	0
STK11	missense_variant	NM_000455.5:c.992G>A	NP_000446.1:p.Arg331Gln	Unknown Significance	ccDK		2	0	1	0	1	0
TP53	missense_variant	NM_000546.6:c.14A>G	NP_000537.3:p.Gln5Arg	Unknown Significance	ccDK		1	0	1	0	0	0
TP53	missense_variant	NM_000546.6:c.214C>A	NP_000537.3:p.Pro72Thr	Unknown Significance	ccDK		1	0	1	0	0	0
TP53	missense_variant	NM_000546.6:c.251C>T	NP_000537.3:p.Ala84Val	Unknown Significance	ccDK		1	0	0	0	0	0
TP53	in-frame_deletion	NM_000546.6:c.336_350delCTTCTTGCATTCTGG	NP_000537.3:p.Phe113_Gly117del	Unknown Significance	PM1 (Cancerhotspot located:17)<br>PM2_supporting (Absent from GnomAD)		1	0	0	0	1	0
TP53	missense_variant	NM_000546.6:c.460G>C	NP_000537.3:p.Gly154Arg	Unknown Significance	ccDK		1	0	0	0	0	0
TP53	missense_variant	NM_000546.6:c.523C>T	NP_000537.3:p.Arg175Cys	Unknown Significance	ccDK		3	0	0	0	0	0
TP53	splice_region_variant	NM_000546.6:c.560-11_560-8dupATTG		Unknown Significance	ccDK		1	0	1	0	0	0
TP53	missense_variant	NM_000546.6:c.572C>G	NP_000537.3:p.Pro191Arg	Unknown Significance	ccDK		5	0	3	0	1	0
TP53	synonymous_variant	NM_000546.6:c.666G>T	NP_000537.3:p.Pro222=	Unknown Significance	ccDK		4	0	3	0	0	0
TP53	splice_region_variant	NM_000546.6:c.672+9T>C		Unknown Significance	ccDK		1	0	1	0	0	0
TP53	missense_variant	NM_000546.6:c.82G>A	NP_000537.3:p.Glu28Lys	Unknown Significance	ccDK		1	0	1	0	0	0
TP53	missense_variant	NM_000546.6:c.847C>T	NP_000537.3:p.Arg283Cys	Unknown Significance	ccDK		1	0	1	0	0	0
TP53	splice_region_variant	NM_000546.6:c.97-4A>G		Unknown Significance	BP4 (SpliceAI=0.03)<br>PM2_supporting (Absent from GnomAD)		2	0	1	0	0	0
TP53	splice_region_variant	NM_000546.6:c.97-9C>T		Unknown Significance	ccDK		1	0	0	0	0	0
TP53	intron_variant	NM_000546.6:c.993+88A>G		Unknown Significance	PP3 (SpliceAI AG: 0.20. Possibly cryptic splice site, but no possible donor site nearby.<br>RAW score changes from 0 to 0.20)		1	0	0	0	0	0

Table S6: Classification ad frequencies of all the identified likely benign and benign variants found in our study populaiton of 5,923 patients with a clinical suspicion of predispostion to heredirary breast- and/or ovarian cancer.
ACMG: American College of Medical Genetics
AG: Acceptor gain
AL: Acceptor loss
AT/A-T: Ataxia-Telangiectasia
BC: Breast cancer
BRCT: BRCA1 C Terminus
ccDK: Cancer (variant) classification DK (The variants classified by this group were not assigned ACMG criteria)
DG: Donor gain
DGC: Differentiated glioblastoma cells
DL: Donor loss
DNE: Dominante-negative effect
ENIGMA: Evidence-based Network for the Interpretation of Germline Mutant Alleles (The variants classified by this group were not assigned ACMG criteria)
GnomAD: Genome Aggregation Database
HDGC: Hereditary diffuse gastric cancer
HDR: Homology-directed repair
HGMD: The Human Gene Mutation Database
HR: Homologous recombination
HRD:Homologous recombination deficiency
LBC: Leukaemia and blood cancer
LOF: Loss of function
mESC: Mouse embryonic stem cell
NMD: Nonsense-mediated decay PTC: Premature termination codons
OC: Ovarian cancer
SRC: Signet ring cell
SS: Splice site
SSF:SpliceSiteFinder
XRCC2: X-ray repair cross complementing 2

Gene	Sequence Ontology	HGVS c.	HGVS p.	Classification	Assigned ACMG criteria	Comments	#Samples_Total (N=5,923)	#Hom samples_Total (N=5,923)	#Samples_BC (N=3,706)	#Hom samples_BC (N=3,706)	#Samples_OC (N=890)	#Hom samples_OC (N=890)
ATM	missense_variant	NM_000051.4:c.1009C>T	NP_000042.3:p.Arg337Cys	Likely Benign	ccDK		3	0	3	0	0	0
ATM	synonymous_variant	NM_000051.4:c.1020C>A	NP_000042.3:p.Ala340=	Likely Benign	BP4 (SpliceAI=0)<br>BP7 (Silent variant with no impact on splicing)		1	0	1	0	0	0
ATM	splice_region_variant	NM_000051.4:c.1066-6T>G		Benign	BS1 (GnomAD frequency 0.00209)<br>BP2_strong (Laboratories reported in ClinVar that the variant is observed in a homozygous and compound heterozygous state in multiple individuals without Ataxia-Telangiectasia; GTR Lab IDs: 61756, 26957, 500031)	SpliceAI AL: 0.62 and DL: 0.63. Possibly exon skipping. RAW score acceptor changes from 0.70 to 0.09 and donor changes from 0.86 to 0.23	42	0	24	0	5	0
ATM	missense_variant	NM_000051.4:c.1073A>G	NP_000042.3:p.Asn358Ser	Likely Benign	BP4 (Revel=0.054)<br>BP2_strong (In ClinVar ClinGen Expert panel reported that the variant has been observed in a homozygous and compound heterozygous state in multiple individuals without biallelic disease; GTR Lab IDs: 500031. 61756)		1	0	0	0	0	0
ATM	synonymous_variant	NM_000051.4:c.1176C>G	NP_000042.3:p.Gly392=	Benign	BS1 (GnomAD frequency 0.00268475)<br>BP4 (SpliceAI=0.01)<br>BP7 (Silent variant with no impact on splicing)<br>BP2_strong (In ClinVar laboratories reported that the variant has been observed in a homozygous and compound heterozygous state in multiple individuals without Ataxia-Telangiectasia (GTR Lab IDs: 500031. 61756))		3	1	2	1	1	0
ATM	missense_variant	NM_000051.4:c.1229T>C	NP_000042.3:p.Val410Ala	Likely Benign	ccDK		26	0	17	0	5	0
ATM	synonymous_variant	NM_000051.4:c.1272T>C	NP_000042.3:p.Pro424=	Likely Benign	BP4 (SpliceAI=0)<br>BP7 (Silent variant with no impact on splicing)		1	0	1	0	0	0
ATM	synonymous_variant	NM_000051.4:c.1380G>C	NP_000042.3:p.Thr460=	Likely Benign	BP4 (SpliceAI=0.03)<br>BP7 (Silent variant with no impact on splicing)		1	0	0	0	0	0
ATM	synonymous_variant	NM_000051.4:c.1494G>A	NP_000042.3:p.Glu498=	Likely Benign	BP4 (SpliceAI=0.00)<br>BP7 (Silent variant with no impact on splicing)		1	0	1	0	0	0
ATM	missense_variant	NM_000051.4:c.1541G>A	NP_000042.3:p.Gly514Asp	Likely Benign	BS1 (GnomAD frequency 0.00324)		2	0	1	0	0	0
ATM	synonymous_variant	NM_000051.4:c.162T>C	NP_000042.3:p.Tyr54=	Likely Benign	ccDK		29	0	22	0	7	0
ATM	missense_variant	NM_000051.4:c.1636C>G	NP_000042.3:p.Leu546Val	Likely Benign	BS1 (GnomAD frequency 0.0027)<br>BS3_supporting (Functional study showing ATM polymorphism (PMID: 19431188))<br>BP4 (Revel=score 0.067)		2	0	0	0	1	0
ATM	missense_variant	NM_000051.4:c.1744T>C	NP_000042.3:p.Phe582Leu	Likely Benign	ccDK		6	0	5	0	0	0
ATM	intron_variant	NM_000051.4:c.1802+22G>T		Likely Benign	BP4 (SpliceAI=0.00)<br>BP7 (Deep intronic variant, +7/-40, and no effect on splicing)		1	0	0	0	0	0
ATM	intron_variant	NM_000051.4:c.1802+36dupT		Likely Benign	BP4 (SpliceAI=0.00)<br>BP7 (Deep intronic variant, +7/-40, and no effect on splicing)		3	0	3	0	0	0
ATM	missense_variant	NM_000051.4:c.1810C>T	NP_000042.3:p.Pro604Ser	Benign	ccDK		10	0	9	0	0	0
ATM	splice_region_variant	NM_000051.4:c.186-17A>G		Likely Benign	BS1 (GnomAD frequency 0.00346)<br>BP4 (SpliceAI=0.00)		8	0	7	0	1	0
ATM	synonymous_variant	NM_000051.4:c.1986T>C	NP_000042.3:p.Phe662=	Likely Benign	BS1 (GnomAD 0.000623)<br>BP4 (SpliceAI=0.04)<br>BP7 (Silent variant with no impact on splicing)		6	0	5	0	0	0
ATM	synonymous_variant	NM_000051.4:c.2013A>T	NP_000042.3:p.Ile671=	Likely Benign	BP4 (SpliceAI=0.00)<br>BP7 (Silent variant with no impact on splicing)		2	0	1	0	0	0
ATM	missense_variant	NM_000051.4:c.2096A>G	NP_000042.3:p.Glu699Gly	Likely Benign	BS1 (GnomAD frequency 0.000136)		1	0	1	0	0	0
ATM	intron_variant	NM_000051.4:c.2125-48T>C		Likely Benign	BS1 (GnomAD frequency 0.0016565)<br>BP4 (SpliceAI=0.00)<br>BP7 (Deep intronic variant, +7/-40, and no effect on splicing)		19	0	11	0	3	0
ATM	synonymous_variant	NM_000051.4:c.2127T>C	NP_000042.3:p.Ile709=	Likely Benign	BP4 (SpliceAI=0.00)<br>BP7 (Silent variant with no impact on splicing)		3	0	3	0	0	0
ATM	synonymous_variant	NM_000051.4:c.2193C>T	NP_000042.3:p.Tyr731=	Likely Benign	BS1 (GnomAD 0.00271216)<br>BP4 (SpliceAI=0.0)<br>BP7 (Silent variant with no impact on splicing)		2	0	0	0	1	0
ATM	missense_variant	NM_000051.4:c.2289T>A	NP_000042.3:p.Phe763Leu	Likely Benign	ccDK		7	0	6	0	0	0
ATM	synonymous_variant	NM_000051.4:c.2370T>C	NP_000042.3:p.Cys790=	Likely Benign	BP4 (SpliceAI=0.00)<br>BP7 (Silent variant with no impact on splicing)		1	0	0	0	1	0
ATM	intron_variant	NM_000051.4:c.2376+40A>G		Likely Benign	BP4 (SpliceAI=0.00)<br>BP7 (Deep intronic variant, +7/-40, and no effect on splicing)		2	0	1	0	1	0
ATM	missense_variant	NM_000051.4:c.2442C>A	NP_000042.3:p.Asp814Glu	Likely Benign	BS1 (GnomAD frequency 0.00136)		2	0	0	0	1	0
ATM	synonymous_variant	NM_000051.4:c.2466A>G	NP_000042.3:p.Leu822=	Likely Benign	BP4 (SpliceAI=0)<br>BP7 (Silent variant and no impact on splicing)		1	0	1	0	1	0
ATM	synonymous_variant	NM_000051.4:c.2523T>C	NP_000042.3:p.Asp841=	Likely Benign	BP4 (SpliceAI=0)<br>BP7 (Silent variant with no impact on splicing)		1	0	1	0	0	0
ATM	missense_variant	NM_000051.4:c.2572T>C	NP_000042.3:p.Phe858Leu	Benign	ccDK		234	2	148	1	34	0
ATM	missense_variant	NM_000051.4:c.2614C>T	NP_000042.3:p.Pro872Ser	Benign	BS1 (GnomAD frequency 0.002414)<br>BP4 (Revel=0.122)<br>BP2_strong (On ClinVar an expert panel states that this variant has been observed in a homozygous or compound heterozygous state in multiple individuals without Ataxia-Telangiectasia; Clinical Diagnostic Laboratories)		1	0	0	0	1	0
ATM	synonymous_variant	NM_000051.4:c.2679A>G	NP_000042.3:p.Gln893=	Likely Benign	BP4 (SpliceAI=0.01)<br>BP7 (Silent variant with no impact on splicing)		3	0	3	0	0	0
ATM	synonymous_variant	NM_000051.4:c.2685A>G	NP_000042.3:p.Leu895=	Likely Benign	BS1 (GnomAD 0.0024312)<br>BP4 (SpliceAI=0.00)<br>BP7 (Silent variant with no impact on splicing)		1	0	0	0	1	0

ATM	synonymous_variant	NM_000051.4:c.2805G>C	NP_000042.3:p.Thr935=	Likely Benign	BS1 (GnomAD 0.000612281)<br>BP4 (SpliceAI=0.02)<br>BP7 (Silent variant with no impact on splicing)		6	0	4	0	0	0
ATM	synonymous_variant	NM_000051.4:c.2859G>A	NP_000042.3:p.Glu953=	Likely Benign	BP4 (SpliceAI=0.07)<br>BP7 (Silent variant with no impact on splicing)		1	0	1	0	0	0
ATM	missense_variant	NM_000051.4:c.3118A>G	NP_000042.3:p.Met1040Val	Benign	BS1 (GnomAD frequency 0.002097)<br>BP4 (Revel=0.096)<br>BP2_strong (In ClinVar ClinGen Expert panel reported that the variant has been observed in a homozygous state in multiple individuals without Ataxia-Telangiectasia; GTR Lab IDs: 500031, 61756)		1	0	1	0	0	0
ATM	synonymous_variant	NM_000051.4:c.3135C>T	NP_000042.3:p.Cys1045=	Likely Benign	BP4 (SpliceAI=0.06)<br>BP7 (Silent variant with no impact on splicing)		2	0	1	0	0	0
ATM	synonymous_variant	NM_000051.4:c.3150T>C	NP_000042.3:p.Leu1050=	Likely Benign	BS1 (GnomAD 0.000803)<br>BP4 (SpliceAI=0.02)<br>BP7 (Silent variant with no impact on splicing)		6	0	2	0	1	0
ATM	intron_variant	NM_000051.4:c.331+2857_331+2860delAGTT		Likely Benign	BS1 (GnomAD frequency 0.0016869)		17	0	9	0	5	0
ATM	intron_variant	NM_000051.4:c.331+48C>T		Likely Benign	BS1 (GnomAD frequency 0.001306)<br>BP4 (SpliceAI=0.00)<br>BP7 (Deep intronic variant, +7/-40, and no effect on splicing)		13	0	8	0	2	0
ATM	synonymous_variant	NM_000051.4:c.3378A>G	NP_000042.3:p.Lys1126=	Likely Benign	BP4 (SpliceAI=0)<br>BP7 (Silent variant and no impact on splicing)		1	0	1	0	0	0
ATM	missense_variant	NM_000051.4:c.3383A>G	NP_000042.3:p.Gln1128Arg	Likely Benign	BS1 (GnomAD frequency 0.00113)<br>BP4 (Revel=0.21)		1	0	0	0	0	0
ATM	intron_variant	NM_000051.4:c.3402+32_3402+34delCAT		Likely Benign	BS1 (GnomAD frequency 0.001465)<br>BP4 (SpliceAI=0.00)<br>BP7 (Deep intronic variant, +7/-40, and no effect on splicing)		6	0	4	0	1	0
ATM	splice_region_variant	NM_000051.4:c.3403-15_3403-14insTA		Likely Benign	BS1 (GnomAD frequency 0.000535)<br>BP4 (SpliceAI=0.00)		3	0	2	0	1	0
ATM	splice_region_variant	NM_000051.4:c.3403-16_3403-15insA		Likely Benign	BS1 (GnomAD frequency 0.000556)<br>BP4 (SpliceAI=0.00)		3	0	2	0	1	0
ATM	synonymous_variant	NM_000051.4:c.3517T>C	NP_000042.3:p.Leu1173=	Likely Benign	BP4 (SpliceAI=0.07)<br>BP7 (Silent variant with no impact on splicing)		1	0	0	0	0	0
ATM	synonymous_variant	NM_000051.4:c.3588A>G	NP_000042.3:p.Lys1196=	Likely Benign	BP4 (Splice 0.00)<br>BP7 (Silent variant with no impact on splicing)		2	0	2	0	0	0
ATM	synonymous_variant	NM_000051.4:c.3897C>G	NP_000042.3:p.Ala1299=	Likely Benign	BP4 (SpliceAI=0.00)<br>BP7 (Silent variant with no impact on splicing)		1	0	0	0	0	0
ATM	missense_variant	NM_000051.4:c.3925G>A	NP_000042.3:p.Ala1309Thr	Benign	BS1 (GnomAD frequency 0.001135)<br>BP4 (Revel=0.076)<br>BP2_strong (In ClinVar ClinGen expert panel reported that the variant has been observed in a homozygous and compound heterozygous state in multiple individuals without ataxia-telangiectasia;Internal laboratory contributions)		16	0	13	0	3	0
ATM	intron_variant	NM_000051.4:c.3993+48A>G		Likely Benign	BS1 (GnomAD frequency 0.001397)<br>BP4 (SpliceAI=0.00)<br>BP7 (Deep intronic variant, +7/-40, and no effect on splicing)		2	0	0	0	1	0
ATM	synonymous_variant	NM_000051.4:c.4042T>C	NP_000042.3:p.Leu1348=	Likely Benign	BS1 (GnomAD 0.00102425)<br>BP4 (SpliceAI=0.00)<br>BP7 (Silent variant with no impact on splicing)		2	0	1	0	0	0
ATM	splice_region_variant	NM_000051.4:c.4109+11T>C		Likely Benign	BP4 (SpliceAI=0.00)<br>BP7 (Deep intronic variant, +7/-40, and no effect on splicing)		3	0	3	0	0	0
ATM	missense_variant	NM_000051.4:c.4362A>C	NP_000042.3:p.Lys1454Asn	Likely Benign	ccDK		12	0	7	0	1	0
ATM	missense_variant	NM_000051.4:c.4424A>G	NP_000042.3:p.Tyr1475Cys	Likely Benign	BS1 (GnomAD frequency 0.000974)		12	0	7	0	1	0
ATM	synonymous_variant	NM_000051.4:c.4473C>T	NP_000042.3:p.Phe1491=	Likely Benign	ccDK		18	0	8	0	4	0
ATM	intron_variant	NM_000051.4:c.4612-52_4612-49delACTT		Likely Benign	BP4 (SpliceAI=0.00)<br>BP7 (Deep intronic variant, +7/-40, and no effect on splicing)		3	0	3	0	0	0
ATM	missense_variant	NM_000051.4:c.4709T>C	NP_000042.3:p.Val1570Ala	Likely Benign	ccDK		4	0	3	0	0	0
ATM	intron_variant	NM_000051.4:c.4776+23_4776+24insG		Likely Benign	BP4 (SpliceAI=0.00)<br>BP7 (Deep intronic variant, +7/-40, and no effect on splicing)		1	0	0	0	1	0
ATM	intron_variant	NM_000051.4:c.4776+41C>T		Likely Benign	BS1 (GnomAD frequency 0.001399)<br>BP4 (SpliceAI=0.00)<br>BP7 (Deep intronic variant, +7/-40, and no effect on splicing)		2	0	0	0	1	0
ATM	missense_variant	NM_000051.4:c.4949A>G	NP_000042.3:p.Asn1650Ser	Likely Benign	BP4 (Revel=0.059)<br>BS1 (GnomAD v.2.1 frequency 0.0006)		1	0	0	0	0	0
ATM	splice_region_variant	NM_000051.4:c.5005+15T>A		Likely Benign	BP4 (SpliceAI=0.00)<br>BP7 (Deep intronic variant, +7/-40, and no effect on splicing)		1	0	1	0	0	0
ATM	splice_region_variant	NM_000051.4:c.5005+18G>A		Likely Benign	BS1 (GnomAD frequency 0.00069)<br>BP4 (SpliceAI=0.00)<br>BP7 (Deep intronic variant, +7/-40, and no effect on splicing)		1	0	1	0	0	0
ATM	intron_variant	NM_000051.4:c.5005+42C>G		Likely Benign	BS1 (GnomAD frequency 0.000913)<br>BP4 (SpliceAI=0.00)<br>BP7 (Deep intronic variant, +7/-40, and no effect on splicing)		9	0	3	0	5	0
ATM	missense_variant	NM_000051.4:c.5071A>C	NP_000042.3:p.Ser1691Arg	Likely Benign	ccDK		38	0	19	0	7	0
ATM	intron_variant	NM_000051.4:c.5320-48A>G		Likely Benign	BP4 (SpliceAI=0.00)<br>BP7 (Deep intronic variant, +7/-40, and no effect on splicing)		1	0	1	0	0	0
ATM	missense_variant	NM_000051.4:c.544G>C	NP_000042.3:p.Val182Leu	Likely Benign	BS1 (GnomAD 0.001939)<br>BP4 (Revel=0.039)		7	0	6	0	1	0
ATM	missense_variant	NM_000051.4:c.5558A>T	NP_000042.3:p.Asp1853Val	Benign	ccDK		69	1	43	0	7	0
ATM	intron_variant	NM_000051.4:c.5675-50T>C		Likely Benign	BP4 (SpliceAI=0.00)<br>BP7 (Deep intronic variant, +7/-40, and no effect on splicing)		2	0	2	0	1	0
ATM	intron_variant	NM_000051.4:c.5762+1025T>C		Likely Benign	BP4 (SpliceAI=0)<br>BP7 (Deep intronic variant, +7/-40, and no effect on splicing)		2	0	1	0	0	0
ATM	intron_variant	NM_000051.4:c.5762+26C>T		Likely Benign	BS1 (GnomAD frequency 0.000707)<br>BP4 (SpliceAI=0.00)<br>BP7 (Deep intronic variant, +7/-40, and no effect on splicing)		1	0	1	0	0	0
ATM	intron_variant	NM_000051.4:c.5762+998delG		Likely Benign	BP4 (SpliceAI=0)<br>BP7 (Deep intronic variant, +7/-40, and no effect on splicing)		1	0	1	0	0	0
ATM	splice_region_variant	NM_000051.4:c.6006+17C>T		Likely Benign	BP4 (SpliceAI=0.00)<br>BP7 (Deep intronic variant, +7/-40, and no effect on splicing)		2	0	1	0	0	0
ATM	intron_variant	NM_000051.4:c.6007-1581A>G		Likely Benign	BS1 (GnomAD frequency 0.0046)	SpliceAI DG: 0.64 and AG: 0.23. Possibly pseudo-exon. RAW score donor 0.64 and acceptor 0.42.	83	1	48	1	11	0
ATM	missense_variant	NM_000051.4:c.6067G>A	NP_000042.3:p.Gly2023Arg	Likely Benign	ccDK		21	0	12	0	4	0
ATM	splice_region_variant	NM_000051.4:c.6095+15T>C		Likely Benign	BP4 (SpliceAI=0.00)<br>BP7 (Deep intronic variant, +7/-40, and no effect on splicing)<br>BS1 (GnomAD frequency 0.002423)		1	0	0	0	1	0
ATM	intron_variant	NM_000051.4:c.6095+26G>T		Likely Benign	PM2_supporting (Absent from GnomAD)<br>BP4 (SpliceAI=0.00)<br>BP7 (Deep intronic variant, +7/-40, and no effect on splicing)		1	0	0	0	0	0
ATM	synonymous_variant	NM_000051.4:c.609C>T	NP_000042.3:p.Asp203=	Likely Benign	ccDK		23	0	16	0	3	0
ATM	missense_variant	NM_000051.4:c.6235G>A	NP_000042.3:p.Val2079Ile	Likely Benign	BS1 (GnomAD frequency 0.001713)<br>BP4 (Revel=0.078)		8	0	5	0	2	0
ATM	synonymous_variant	NM_000051.4:c.6333T>C	NP_000042.3:p.His2111=	Likely Benign	BP4 (SpliceAI=0.00)<br>BP7 (Silent variant with no impact on splicing)		2	0	2	0	0	0
ATM	intron_variant	NM_000051.4:c.6347+47C>T		Likely Benign	BP4 (SpliceAI=0)<br>BP7 (Deep intronic variant, +7/-40, and no effect on splicing)		3	0	1	0	1	0
ATM	missense_variant	NM_000051.4:c.6437G>C	NP_000042.3:p.Ser2146Thr	Likely Benign	BS1 (GnomAD frequency 0.000707)<br>BP4 (Revel=0.046)		1	0	0	0	1	0
ATM	intron_variant	NM_000051.4:c.6453-37_6453-34delCTTT		Likely Benign	BS1 (GnomAD frequency 0.0008683)<br>BP4 (SpliceAI=0.00)		15	0	7	0	2	0

ATM	intron_variant	NM_000051.4:c.6453-550T>C		Likely Benign	BS1 (GnomAD frequency 0.0005777)		6	0	3	0	2	0
ATM	splice_region_variant	NM_000051.4:c.6572+12G>T		Likely Benign	BS1 (GnomAD frequency 0.0023)<br>BP4 (SpliceAI=0.00)<br>BP7 (Deep intronic variant, +7/-40, and no effect on splicing)		1	0	0	0	1	0
ATM	synonymous_variant	NM_000051.4:c.657T>C	NP_000042.3:p.Cys219=	Likely Benign	BP4 (SpliceAI=0)<br>BP7 (Silent variant with no impact on splicing)<br>BS1 (GnomAD frequency 0.004896)		1	0	1	0	0	0
ATM	intron_variant	NM_000051.4:c.662+38T>C		Likely Benign	BS1 (GnomAD frequency 0.00484)<br>BP4 (SpliceAI=0.00)<br>BP7 (Deep intronic variant, +7/-40, and no effect on splicing)		9	1	7	1	1	0
ATM	missense_variant	NM_000051.4:c.6860G>C	NP_000042.3:p.Gly2287Ala	Likely Benign	BP4 (Revel=0.077)<br>BS3_moderate (Functional study showing normal kinase activity and corrected the radiosensitive phenotype (PMID: 11805335))		4	0	3	0	1	0
ATM	synonymous_variant	NM_000051.4:c.6888A>T	NP_000042.3:p.Ala2296=	Likely Benign	BP4 (SpliceAI=0.00)<br>BP7 (Silent variant with no impact on splicing)		1	0	1	0	0	0
ATM	missense_variant	NM_000051.4:c.6919C>T	NP_000042.3:p.Leu2307Phe	Likely Benign	BS1 (GnomAD frequency 0.000745)		1	0	0	0	1	0
ATM	splice_region_variant	NM_000051.4:c.6975+13delT		Likely Benign	BP4 (SpliceAI=0)<br>BP7 (Deep intronic variant, +7/-40, and no impact on splicing)		1	0	1	0	0	0
ATM	missense_variant	NM_000051.4:c.6995T>C	NP_000042.3:p.Leu2332Pro	Benign	BS1 (GnomAD frequency 0.00111)<br>BP4 (Revel=0.193)<br>BP2_strong (In ClinVar ClinGen expert panel reports the variant have been observed in a homozygous and compound heterozygous state in multiple individuals without biallelic disease; GTR Lab IDs: 61756. 500031)		1	0	0	0	1	0
ATM	synonymous_variant	NM_000051.4:c.7239G>A	NP_000042.3:p.Lys2413=	Likely Benign	PM2_supporting (Absent from GnomAD)<br>BP4 (SpliceAI=0.02)<br>BP7 (Silent variant with no impact on splicing)		1	0	0	0	0	0
ATM	synonymous_variant	NM_000051.4:c.7266G>A	NP_000042.3:p.Glu2422=	Likely Benign	BP4 (SpliceAI=0.00)<br>BP7 (Silent variant with no impact on splicing)		1	0	0	0	0	0
ATM	synonymous_variant	NM_000051.4:c.7383C>T	NP_000042.3:p.Arg2461=	Likely Benign	BP4 (SpliceAI=0.00)<br>BP7 (Silent variant with no impact on splicing)		1	0	1	0	0	0
ATM	missense_variant	NM_000051.4:c.7390T>C	NP_000042.3:p.Cys2464Arg	Likely Benign	BS1 (GnomAD frequency 0.000562)		21	0	11	0	2	0
ATM	intron_variant	NM_000051.4:c.7515+45_7515+46delTT		Likely Benign	BS1 (GnomAD Frequency 0.00099)<br>BP4 (SpliceAI=0)<br>BP7 (Deep intronic variant, +7/-40, and no impact on splicing)		15	0	8	0	3	0
ATM	intron_variant	NM_000051.4:c.7515+49A>T		Likely Benign	PM2_supporting (Absent from GnomAD)<br>BP4 (SpliceAI=0)<br>BP7 (Deep intronic variant, +7/-40, and no impact on splicing)		2	0	2	0	0	0
ATM	synonymous_variant	NM_000051.4:c.7539A>G	NP_000042.3:p.Thr2513=	Likely Benign	BP4 (SpliceAI=0.00)<br>BP7 (Silent variant with no impact on splicing)		1	0	0	0	1	0
ATM	synonymous_variant	NM_000051.4:c.7620C>A	NP_000042.3:p.Val2540=	Likely Benign	BP4 (SpliceAI=0.00)<br>BP7 (Silent variant with no impact on splicing)		2	0	2	0	0	0
ATM	splice_region_variant	NM_000051.4:c.7788+8G>T		Likely Benign	BS1 (GnomAD frequency 0.0027)<br>BP4 (SpliceAI=0.00)<br>BP7 (Deep intronic variant, +7/-40, and no impact on splicing)		15	0	10	0	2	0
ATM	intron_variant	NM_000051.4:c.7928-54_7928-49delCCTTCA		Likely Benign	BP4 (SpliceAI=0)<br>BP7 (Deep intronic variant, +7/-40, and no impact on splicing)		3	0	3	0	0	0
ATM	synonymous_variant	NM_000051.4:c.7983T>C	NP_000042.3:p.Asp2661=	Likely Benign	BP4 (SpliceAI=0.00)<br>BP7 (Silent variant with no impact on splicing)		2	0	1	0	0	0
ATM	synonymous_variant	NM_000051.4:c.7998T>A	NP_000042.3:p.Thr2666=	Likely Benign	BP4 (SpliceAI=0)<br>BP7 (Silent variant with no impact on splicing)		1	0	1	0	0	0
ATM	intron_variant	NM_000051.4:c.8010+30dupT		Likely Benign	BS1 (GnomAD frequency 0.00704)<br>BP4 (SpliceAI=0)<br>BP7 (Deep intronic variant, +7/-40, and no impact on splicing)		2	0	0	0	1	0
ATM	intron_variant	NM_000051.4:c.8419-54_8419-49delTAGTTT		Likely Benign	BS1 (GnomAD frequency 0.0021334)<br>BP4 (SpliceAI=0)<br>BP7 (Deep intronic variant, +7/-40, and no impact on splicing)		24	0	12	0	7	0
ATM	intron_variant	NM_000051.4:c.8584+31C>G		Likely Benign	BP4 (SpliceAI=0.00)<br>BP7 (Deep intronic variant, +7/-40, and no impact on splicing)		1	0	0	0	0	0
ATM	synonymous_variant	NM_000051.4:c.8592C>T	NP_000042.3:p.Tyr2864=	Likely Benign	ccDK		12	0	7	0	3	0
ATM	synonymous_variant	NM_000051.4:c.876G>A	NP_000042.3:p.Pro292=	Likely Benign	BP4 (SpliceAI=0.00)<br>BP7 (Silent variant with no impact on splicing)		1	0	1	0	0	0
ATM	intron_variant	NM_000051.4:c.8850+33_8850+35delTTT		Likely Benign	BP4 (SpliceAI=0.00)<br>BP7 (Deep intronic variant, +7/-40, and no impact on splicing)		1	0	1	0	0	0
ATM	synonymous_variant	NM_000051.4:c.957A>T	NP_000042.3:p.Leu319=	Likely Benign	BP4 (SpliceAI=0)<br>BP7 (Silent variant with no impact on splicing)		1	0	1	0	0	0
ATM	synonymous_variant	NM_000051.4:c.993G>A	NP_000042.3:p.Lys331=	Likely Benign	BP4 (SpliceAI=0)<br>BP7 (Silent variant and no impact on splicing)		1	0	0	0	1	0
ATM	missense_variant	NM_000051.4:c.998C>T	NP_000042.3:p.Ser333Phe	Likely Benign	BS1 (GnomAD frequency 0.00143)<br>BP4 (Revel=score 0.073)<br>BP2_supporting (1 unaffacted patient with unknown phase (PMID: 33280026))		15	0	11	0	3	0
BARD1	synonymous_variant	NM_000465.4:c.1083A>G	NP_000456.2:p.Glu361=	Likely Benign	BP4 (SpliceAI=0.00)<br>BP7 (Silent variant with no impact on splicing)		1	0	1	0	0	0
BARD1	synonymous_variant	NM_000465.4:c.1152C>T	NP_000456.2:p.Ser384=	Likely Benign	BP4 (SpliceAI=0)<br>BP7 (Silent variant and no impact on splicing)		2	0	2	0	0	0
BARD1	synonymous_variant	NM_000465.4:c.126C>T	NP_000456.2:p.Asp42=	Likely Benign	BP4 (SpliceAI=0.01)<br>BP7 (Silent variant with no impact on splicing)		1	0	1	0	0	0
BARD1	intron_variant	NM_000465.4:c.1314+29C>G		Likely Benign	BP4 (SpliceAI=0.04)<br>BP7 (Deep intronic variant, +7/-40, and no effect on splicing)<br>BP6 (Laboratories in ClinVar reported it as benign)		304	3	177	1	58	2
BARD1	synonymous_variant	NM_000465.4:c.1347A>G	NP_000456.2:p.Gln449=	Likely Benign	BP4 (SpliceAI=0.01)<br>BP7 (Silent variant with no impact on splicing)<br>BP6 (Laboratories in ClinVar reported it as benign)		1	0	1	0	0	0
BARD1	intron_variant	NM_000465.4:c.1395+50T>C		Likely Benign	BP7 (Deep intronic variant, +7/-21, and no impact on splicing<br>BP4 (SpliceAI=0.00)		29	0	19	0	2	0
BARD1	synonymous_variant	NM_000465.4:c.13C>A	NP_000456.2:p.Arg5=	Likely Benign	BP4 (SpliceAI=0.00)<br>BP7 (Silent variant with no impact on splicing)<br>PM2_supporitng (Absent from GnomAD)		2	0	1	0	0	0
BARD1	missense_variant	NM_000465.4:c.1409A>G	NP_000456.2:p.Asn470Ser	Likely Benign	BS3 (Functional study predicts that the variant not damaging because of not showing significant alteration in either structure or stability (PMID: 18480049))<br>BP6 (Laboratories in ClinVar reported it benign)		4	0	3	0	1	0
BARD1	synonymous_variant	NM_000465.4:c.1479A>G	NP_000456.2:p.Gln493=	Likely Benign	BP4 (SpliceAI=0)<br>BP7 (Silent variant and no impact on splicing)		1	0	1	0	0	0
BARD1	synonymous_variant	NM_000465.4:c.1491A>G	NP_000456.2:p.Pro497=	Likely Benign	BP4 (SpliceAI=0)<br>BP7 (Silent variant and no impact on splicing)<br>BP6 (Laboratories reported it benign in ClinVar)		2	0	1	0	1	0
BARD1	synonymous_variant	NM_000465.4:c.1548T>C	NP_000456.2:p.Tyr516=	Likely Benign	BP4 (SpliceAI=0)<br>BP7 (Silent variant and no impact on splicing)		1	0	1	0	0	0
BARD1	intron_variant	NM_000465.4:c.1568+39C>G		Likely Benign	BP4 (SpliceAI=0.00)<br>BP7 (Deep intronic variant, +7/-21, and no impact on splicing)		2	0	2	0	0	0
BARD1	intron_variant	NM_000465.4:c.158+41G>T		Likely Benign	BP4 (SpliceAI=0.03)<br>BP7 (Deep intronic variant, +7/-21, and no impact on splicing)		13	0	6	0	0	0
BARD1	missense_variant	NM_000465.4:c.160A>G	NP_000456.2:p.Thr54Ala	Likely Benign	BS3 (Functional study showing no damaging effect (PMID: 26350354))<br>BP4 (Revel=0.216 and BayesDel=-0.386)		3	0	2	0	0	0
BARD1	synonymous_variant	NM_000465.4:c.1623G>A	NP_000456.2:p.Ser541=	Likely Benign	BP4 (SpliceAI=0)<br>BP7 (Silent variant and no impact on splicing)		1	0	1	0	0	0

BARD1	missense_variant	NM_000465.4:c.1670G>C	NP_000456.2:p.Cys557Ser	Likely Benign	BS3 (Functional study showing no effect (PMID: 17848578))<br>BP4 (Revel=0.142 and BayesDel=-0.20)<br>BP6 (Laboratories reported it benign in ClinVar)		303	3	174	1	58	2
BARD1	splice_region_variant	NM_000465.4:c.1677+17C>T		Likely Benign	BP4 (SpliceAI=0.03)<br>BP7 (Deep intronic variant, +7/-21, and no impact on splicing)		3	0	1	0	2	0
BARD1	missense_variant	NM_000465.4:c.1694G>A	NP_000456.2:p.Arg565His	Likely Benign	BP4 (Revel=0.179 and BayesDel=-0.2455)<br>BP6 (Laboratories reported it benign at ClinVar)		2	0	2	0	0	0
BARD1	missense_variant	NM_000465.4:c.1738G>A	NP_000456.2:p.Glu580Lys	Likely Benign	BP4 (Revel=0.138 and BayesDel=-0.477)<br>BP6 (Laboratories reported it benign in ClinVar)		2	0	2	0	0	0
BARD1	intron_variant	NM_000465.4:c.1904-34dupA		Likely Benign	BP4 (SpliceAI=0.02)<br>BP7 (Deep intronic variant, +7/-21, and no impact on splicing)		5	0	4	0	2	0
BARD1	missense_variant	NM_000465.4:c.1933T>C	NP_000456.2:p.Cys645Arg	Likely Benign	BS3 (Functional study showing no damaging effect (PMID: 16061562))<br>BP4 (Revel=0.048 and BayesDel=-0.66)<br>BP6 (Laboratories reported it benign in ClinVar)		3	0	3	0	0	0
BARD1	missense_variant	NM_000465.4:c.1972C>T	NP_000456.2:p.Arg658Cys	Likely Benign	BS3 (Functional studies showing no damaging effect (PMID: 16061562 and PMID: 26350354))<br>BP4 (Revel=0.104 and BayesDel=-0.377)<br>BP6 (Laboratories reported it benign in ClinVar)		80	0	47	0	14	0
BARD1	synonymous_variant	NM_000465.4:c.1977A>G	NP_000456.2:p.Arg659=	Likely Benign	BP4 (SpliceAI=0.00)<br>BP7 (Silent variant with no impact on splicing)<br>BP6 (Laboratories in ClinVar reported it as benign)<br>BS3 (Since no damaging effect on the gene or gene product was detected and allele-specific silencing was dismissed (PMID: 34824355))		33	0	18	0	6	0
BARD1	synonymous_variant	NM_000465.4:c.2013G>A	NP_000456.2:p.Leu671=	Likely Benign	BP4 (SpliceAI=0.00)<br>BP7 (Silent variant with no impact on splicing)		7	0	3	0	2	0
BARD1	missense_variant	NM_000465.4:c.2075T>C	NP_000456.2:p.Ile692Thr	Likely Benign	BP4 (Revel=0.111 and BayesDel=-0.54)<br>BS3 (Functional study showing functional HDR function (PMID: 26350354))		1	0	1	0	0	0
BARD1	missense_variant	NM_000465.4:c.2161G>A	NP_000456.2:p.Ala721Thr	Likely Benign	BS3 (Functional study showing fucntionel HDR assay (PMID: 26350354))<br>BP4 (Revel=0.204 and BayesDel=0.0072)		1	0	1	0	0	0
BARD1	synonymous_variant	NM_000465.4:c.2178C>T	NP_000456.2:p.Pro726=	Likely Benign	BP4 (SpliceAI=0.00)<br>BP7 (Silent variant and no impact on splicing)		1	0	1	0	0	0
BARD1	missense_variant	NM_000465.4:c.2212A>G	NP_000456.2:p.Ile738Val	Likely Benign	BP4 (Revel=0.011 and BayesDel=-0.627)<br>BP6 (Laboratories reported it benign in Clinvar)		91	0	58	0	8	0
BARD1	synonymous_variant	NM_000465.4:c.2262A>G	NP_000456.2:p.Lys754=	Likely Benign	BP4 (SpliceAI=0.00)<br>BP7 (Silent variant with no impact on splicing)		3	0	2	0	0	0
BARD1	missense_variant	NM_000465.4:c.2282G>A	NP_000456.2:p.Ser761Asn	Likely Benign	BP4 (BayesDel=-0.68 and Revel=0.059)<br>BP5 (Laboratories at ClinVar reported as benign)		16	0	15	0	1	0
BARD1	missense_variant	NM_000465.4:c.33G>T	NP_000456.2:p.Gln11His	Likely Benign	BP4 (Revel=0.156 and BayesDel=-0.3697)<br>BP6 (Laboratories reported it benign in ClinVar)		18	0	13	0	4	0
BARD1	missense_variant	NM_000465.4:c.346C>T	NP_000456.2:p.His116Tyr	Likely Benign	BS3 (Functional study showing functional protein (PMID: 26350354))<br>BP4 (Revel=0.054 and BayesDel=-0.474)		4	0	1	0	0	0
BARD1	splice_region_variant	NM_000465.4:c.365-8delT		Likely Benign	BP4 (SpliceAI=0.05)<br>BP5 (Laboratories reported it as benign in ClinVar)		1	0	0	0	0	0
BARD1	missense_variant	NM_000465.4:c.568G>A	NP_000456.2:p.Asp190Asn	Likely Benign	BS3 (Functional study showing no damaging effect (PMID: 30925164))<br>BP4 (Revel=0.053 and BayesDel=-0.567)		3	0	2	0	1	0
BARD1	synonymous_variant	NM_000465.4:c.57G>A	NP_000456.2:p.Glu19=	Likely Benign	BP4 (SpliceAI=0)<br>BP7 (Silent variant and no impact on splicing)		1	0	1	0	0	0
BARD1	synonymous_variant	NM_000465.4:c.609A>C	NP_000456.2:p.Gly203=	Likely Benign	BP4 (SpliceAI=0.00)<br>BP7 (Silent variant with no impact on splicing)<br>BP6 (Laboratories reported it as benign)		98	0	61	0	9	0
BARD1	missense_variant	NM_000465.4:c.668A>G	NP_000456.2:p.Glu223Gly	Likely Benign	BS3 (Functional study showing similar effect as wildtype (PMID: 30925164))<br>BP4 (Revel=0.085 and BayesDel=-0.4483)		2	0	1	0	1	0
BARD1	synonymous_variant	NM_000465.4:c.732C>T	NP_000456.2:p.Ser244=	Likely Benign	BP4 (SpliceAI=0.00)<br>BP7 (Silent variant with no impact on splicing)<br>PM2_supporitng (GnomAD only 1)		1	0	1	0	0	0
BARD1	synonymous_variant	NM_000465.4:c.738A>G	NP_000456.2:p.Pro246=	Likely Benign	BP4 (SpliceAI=0.01)<br>BP7 (Silent variant with no impact on splicing)		4	0	2	0	0	0
BARD1	synonymous_variant	NM_000465.4:c.768T>C	NP_000456.2:p.Gly256=	Likely Benign	BP4 (SpliceAI=0)<br>BP7 (Silent variant and no impact on splicing)<br>PM2_supporting (GnomAD only 1)		1	0	1	0	0	0
BARD1	synonymous_variant	NM_000465.4:c.876G>A	NP_000456.2:p.Lys292=	Likely Benign	BP4 (SpliceAI=0.00)<br>BP7 (Silent variant with no impact on splicing)<br>PM2_supporitng (GnomAD only 1)		3	0	2	0	1	0
BRCA1	synonymous_variant	NM_007294.4:c.1065G>A	NP_009225.1:p.Lys355=	Likely Benign	ENIGMA		2	0	1	0	0	0
BRCA1	synonymous_variant	NM_007294.4:c.1311T>C	NP_009225.1:p.His437=	Likely Benign	ccDK		1	0	0	0	1	0
BRCA1	missense_variant	NM_007294.4:c.1396C>T	NP_009225.1:p.Arg466Trp	Likely Benign	ccDK		1	0	0	0	0	0
BRCA1	missense_variant	NM_007294.4:c.1405G>A	NP_009225.1:p.Ala469Thr	Likely Benign	BP1_strong (Located outside functional domain and SpliceAI=0.00)		1	0	1	0	0	0
BRCA1	missense_variant	NM_007294.4:c.1456T>C	NP_009225.1:p.Phe486Leu	Benign	ENIGMA		1	0	0	0	0	0
BRCA1	missense_variant	NM_007294.4:c.1486C>T	NP_009225.1:p.Arg496Cys	Benign	ENIGMA		5	0	3	0	2	0
BRCA1	missense_variant	NM_007294.4:c.1487G>A	NP_009225.1:p.Arg496His	Benign	ENIGMA		13	0	10	0	2	0
BRCA1	missense_variant	NM_007294.4:c.1555A>C	NP_009225.1:p.Lys519Gln	Benign	ccDK		1	0	1	0	0	0
BRCA1	missense_variant	NM_007294.4:c.1616C>T	NP_009225.1:p.Thr539Met	Benign	ENIGMA		1	0	1	0	0	0
BRCA1	missense_variant	NM_007294.4:c.1648A>C	NP_009225.1:p.Asn550His	Benign	ENIGMA		1	0	0	0	0	0
BRCA1	missense_variant	NM_007294.4:c.172C>A	NP_009225.1:p.Pro58Thr	Likely Benign	ccDK		1	0	1	0	0	0
BRCA1	missense_variant	NM_007294.4:c.1865C>T	NP_009225.1:p.Ala622Val	Benign	ENIGMA		1	0	1	0	0	0
BRCA1	missense_variant	NM_007294.4:c.1879G>A	NP_009225.1:p.Val627Ile	Likely Benign	ccDK		1	0	0	0	0	0
BRCA1	synonymous_variant	NM_007294.4:c.1911T>C	NP_009225.1:p.Thr637=	Likely Benign	ENIGMA		1	0	1	0	0	0
BRCA1	missense_variant	NM_007294.4:c.199G>T	NP_009225.1:p.Asp67Tyr	Benign	ENIGMA		1	0	0	0	1	0
BRCA1	splice_region_variant	NM_007294.4:c.-20+11C>T		Likely Benign	ccDK		11	0	9	0	0	0
BRCA1	missense_variant	NM_007294.4:c.2002C>T	NP_009225.1:p.Leu668Phe	Benign	ENIGMA		2	0	2	0	1	0
BRCA1	synonymous_variant	NM_007294.4:c.217C>T	NP_009225.1:p.Leu73=	Likely Benign	ENIGMA		1	0	1	0	0	0
BRCA1	synonymous_variant	NM_007294.4:c.2223T>C	NP_009225.1:p.Ser741=	Likely Benign	ccDK		1	0	1	0	0	0
BRCA1	missense_variant	NM_007294.4:c.2315T>C	NP_009225.1:p.Val772Ala	Benign	ENIGMA		1	0	1	0	0	0
BRCA1	synonymous_variant	NM_007294.4:c.2352G>A	NP_009225.1:p.Ser784=	Likely Benign	ENIGMA		2	0	1	0	0	0
BRCA1	missense_variant	NM_007294.4:c.2412G>C	NP_009225.1:p.Gln804His	Benign	ENIGMA		2	0	0	0	1	0
BRCA1	missense_variant	NM_007294.4:c.2518A>T	NP_009225.1:p.Ser840Cys	Likely Benign	ccDK		1	0	0	0	0	0
BRCA1	missense_variant	NM_007294.4:c.2521C>T	NP_009225.1:p.Arg841Trp	Benign	ENIGMA		21	0	12	0	2	0
BRCA1	missense_variant	NM_007294.4:c.2522G>A	NP_009225.1:p.Arg841Gln	Likely Benign	ccDK		2	0	1	0	0	0
BRCA1	synonymous_variant	NM_007294.4:c.255G>A	NP_009225.1:p.Glu85=	Likely Benign	ENIGMA		2	0	2	0	0	0
BRCA1	missense_variant	NM_007294.4:c.2590T>G	NP_009225.1:p.Ser864Ala	Likely Benign	ccDK		1	0	1	0	0	0
BRCA1	missense_variant	NM_007294.4:c.2596C>T	NP_009225.1:p.Arg866Cys	Benign	ENIGMA		2	0	2	0	0	0
BRCA1	missense_variant	NM_007294.4:c.2630A>G	NP_009225.1:p.Asn877Ser	Likely Benign	ccDK		1	0	1	0	1	0
BRCA1	in-frame_deletion	NM_007294.4:c.2716_2730delAAGGAAGAAAATCAA	NP_009225.1:p.Lys906_Gln910del	Likely Benign	BP1_Strong (Located outside functional domain and SpliceAI=0.04)		5	0	4	0	1	0
BRCA1	missense_variant	NM_007294.4:c.2728C>G	NP_009225.1:p.Gln910Glu	Likely Benign	ccDK		1	0	0	0	0	0
BRCA1	synonymous_variant	NM_007294.4:c.2733A>G	NP_009225.1:p.Gly911=	Likely Benign	ENIGMA		3	0	2	0	0	0
BRCA1	synonymous_variant	NM_007294.4:c.2814A>G	NP_009225.1:p.Pro938=	Benign	ENIGMA		1	0	1	0	0	0
BRCA1	missense_variant	NM_007294.4:c.2836A>G	NP_009225.1:p.Ile946Val	Likely Benign	ccDK		1	0	0	0	1	0
BRCA1	splice_region_variant	NM_007294.4:c.301+7G>A		Benign	ENIGMA		2	0	1	0	0	0
BRCA1	intron_variant	NM_007294.4:c.302-41T>C		Benign	ENIGMA		2	0	0	0	0	0
BRCA1	missense_variant	NM_007294.4:c.3024G>A	NP_009225.1:p.Met1008Ile	Benign	ENIGMA		3	0	2	0	0	0
BRCA1	missense_variant	NM_007294.4:c.3119G>A	NP_009225.1:p.Ser1040Asn	Benign	ENIGMA		217	2	130	2	45	0
BRCA1	missense_variant	NM_007294.4:c.314A>G	NP_009225.1:p.Tyr105Cys	Benign	ENIGMA		1	0	1	0	0	0
BRCA1	missense_variant	NM_007294.4:c.3302G>A	NP_009225.1:p.Ser1101Asn	Benign	ENIGMA		1	0	1	0	0	0
BRCA1	missense_variant	NM_007294.4:c.3338A>G	NP_009225.1:p.Tyr1113Cys	Likely Benign	BP1_strong (Located outside functional domain and SpliceAI=0.00)		2	0	0	0	0	0
BRCA1	missense_variant	NM_007294.4:c.3416G>T	NP_009225.1:p.Ser1139Ile	Benign	ENIGMA		3	0	3	0	0	0

BRCA1	missense_variant	NM_007294.4:c.3418A>G	NP_009225.1:p.Ser1140Gly	Benign	ENIGMA		4	0	3	0	1	0
BRCA1	synonymous_variant	NM_007294.4:c.3477A>C	NP_009225.1:p.Ile1159=	Likely Benign	BP1_strong (Located outside functional domain and SpliceAI=0)		2	0	0	0	2	0
BRCA1	synonymous_variant	NM_007294.4:c.3576T>C	NP_009225.1:p.Pro1192=	Likely Benign	BP1_strong (Silent variant outside important functional domain AND no splicing predicted. SpliceAI=0.01)		1	0	1	0	0	0
BRCA1	missense_variant	NM_007294.4:c.3608G>A	NP_009225.1:p.Arg1203Gln	Benign	ccDK		2	0	1	0	0	0
BRCA1	synonymous_variant	NM_007294.4:c.3636A>G	NP_009225.1:p.Ser1212=	Likely Benign	ccDK		1	0	0	0	0	0
BRCA1	missense_variant	NM_007294.4:c.3657G>C	NP_009225.1:p.Glu1219Asp	Benign	ENIGMA		1	0	0	0	1	0
BRCA1	missense_variant	NM_007294.4:c.3708T>G	NP_009225.1:p.Asn1236Lys	Benign	ENIGMA		3	0	3	0	0	0
BRCA1	missense_variant	NM_007294.4:c.3713C>T	NP_009225.1:p.Pro1238Leu	Benign	ENIGMA		2	0	1	0	0	0
BRCA1	missense_variant	NM_007294.4:c.3739G>A	NP_009225.1:p.Val1247Ile	Benign	ENIGMA		1	0	1	0	0	0
BRCA1	synonymous_variant	NM_007294.4:c.3747C>T	NP_009225.1:p.Thr1249=	Likely Benign	ccDK		1	0	0	0	1	0
BRCA1	missense_variant	NM_007294.4:c.3748G>A	NP_009225.1:p.Glu1250Lys	Benign	ENIGMA		7	0	4	0	1	0
BRCA1	missense_variant	NM_007294.4:c.3823A>G	NP_009225.1:p.Ile1275Val	Benign	ENIGMA		2	0	1	0	0	0
BRCA1	missense_variant	NM_007294.4:c.3998T>C	NP_009225.1:p.Val1333Ala	Likely Benign	BP1_strong (Located outside functional domain and SpliceAI=0.00)		1	0	1	0	0	0
BRCA1	missense_variant	NM_007294.4:c.4039A>G	NP_009225.1:p.Arg1347Gly	Benign	ENIGMA		94	1	57	0	13	0
BRCA1	intron_variant	NM_007294.4:c.4185+21_4185+22dupTG		Likely Benign	ccDK		5	0	4	0	1	0
BRCA1	synonymous_variant	NM_007294.4:c.4245A>G	NP_009225.1:p.Glu1415=	Likely Benign	ENIGMA		2	0	2	0	0	0
BRCA1	splice_region_variant	NM_007294.4:c.4357+17A>G		Likely Benign	ccDK		2	0	2	0	0	0
BRCA1	intron_variant	NM_007294.4:c.4358-45A>C		Likely Benign	BP4 (SpliceAI=0.02)<br>BP7 (Deep intronic variant ,+7/-21, and no impact on splicing)		1	0	0	0	1	0
BRCA1	intron_variant	NM_007294.4:c.442-21G>A		Likely Benign	BP4 (SpliceAI=0.0)<br>BP7 (Deep intronic variant, +7/-21, and no impact on splicing)		1	0	1	0	0	0
BRCA1	splice_region_variant	NM_007294.4:c.4484+14A>G		Benign	ENIGMA		2	0	2	0	0	0
BRCA1	missense_variant	NM_007294.4:c.4535G>T	NP_009225.1:p.Ser1512Ile	Benign	ENIGMA		54	0	33	0	7	0
BRCA1	missense_variant	NM_007294.4:c.4600G>A	NP_009225.1:p.Val1534Met	Benign	ENIGMA		2	0	0	0	1	0
BRCA1	missense_variant	NM_007294.4:c.4636G>A	NP_009225.1:p.Asp1546Asn	Benign	ENIGMA		1	0	0	0	0	0
BRCA1	synonymous_variant	NM_007294.4:c.4812A>G	NP_009225.1:p.Gln1604=	Benign	ENIGMA		9	0	5	0	2	0
BRCA1	missense_variant	NM_007294.4:c.4883T>C	NP_009225.1:p.Met1628Thr	Benign	ENIGMA		1	0	0	0	1	0
BRCA1	missense_variant	NM_007294.4:c.4910C>T	NP_009225.1:p.Pro1637Leu	Benign	ENIGMA		1	0	1	0	0	0
BRCA1	missense_variant	NM_007294.4:c.4955T>C	NP_009225.1:p.Met1652Thr	Benign	ENIGMA		1	0	0	0	1	0
BRCA1	missense_variant	NM_007294.4:c.4956G>A	NP_009225.1:p.Met1652Ile	Benign	ENIGMA		133	2	80	1	22	0
BRCA1	synonymous_variant	NM_007294.4:c.5052T>G	NP_009225.1:p.Thr1684=	Likely Benign	ccDK		1	0	0	0	0	0
BRCA1	missense_variant	NM_007294.4:c.509G>A	NP_009225.1:p.Arg170Gln	Likely Benign	ccDK		2	0	0	0	1	0
BRCA1	splice_region_variant	NM_007294.4:c.5153-11G>T		Likely Benign	ccDK		1	0	0	0	0	0
BRCA1	synonymous_variant	NM_007294.4:c.5175A>G	NP_009225.1:p.Glu1725=	Likely Benign	ccDK		1	0	1	0	0	0
BRCA1	missense_variant	NM_007294.4:c.5189A>G	NP_009225.1:p.Asn1730Ser	Likely Benign	ccDK		1	0	1	0	0	0
BRCA1	missense_variant	NM_007294.4:c.536A>G	NP_009225.1:p.Tyr179Cys	Benign	ENIGMA		1	0	0	0	0	0
BRCA1	intron_variant	NM_007294.4:c.5406+46_5406+47delTC		Likely Benign	ccDK		2	0	1	0	1	0
BRCA1	intron_variant	NM_007294.4:c.5407-32C>T		Likely Benign	ccDK		3	0	1	0	2	0
BRCA1	missense_variant	NM_007294.4:c.5411T>A	NP_009225.1:p.Val1804Asp	Benign	ENIGMA		1	0	1	0	0	0
BRCA1	splice_region_variant	NM_007294.4:c.5468-10C>A		Benign	ENIGMA		1	0	1	0	0	0
BRCA1	splice_region_variant	NM_007294.4:c.548-17G>T		Benign	ENIGMA		4	0	2	0	0	0
BRCA1	splice_region_variant	NM_007294.4:c.548-18T>G		Likely Benign	ccDK		1	0	0	0	0	0
BRCA1	splice_region_variant	NM_007294.4:c.548-3delT		Likely Benign	ccDK		5	0	3	0	0	0
BRCA1	missense_variant	NM_007294.4:c.5504G>A	NP_009225.1:p.Arg1835Gln	Likely Benign	ccDK		1	0	1	0	0	0
BRCA1	synonymous_variant	NM_007294.4:c.5514G>T	NP_009225.1:p.Val1838=	Likely Benign	ENIGMA		4	0	4	0	0	0
BRCA1	missense_variant	NM_007294.4:c.5536C>A	NP_009225.1:p.Gln1846Lys	Benign	ccDK		1	0	1	0	0	0
BRCA1	missense_variant	NM_007294.4:c.571G>A	NP_009225.1:p.Val191Ile	Benign	ENIGMA		2	0	2	0	0	0
BRCA1	synonymous_variant	NM_007294.4:c.591C>T	NP_009225.1:p.Cys197=	Benign	ENIGMA		6	0	4	0	0	0
BRCA1	synonymous_variant	NM_007294.4:c.597G>A	NP_009225.1:p.Val199=	Likely Benign	ccDK		1	0	0	0	1	0
BRCA1	missense_variant	NM_007294.4:c.612G>C	NP_009225.1:p.Leu204Phe	Likely Benign	ccDK		3	0	3	0	0	0
BRCA1	splice_region_variant	NM_007294.4:c.671-18_671-16delATT		Likely Benign	ccDK		3	0	2	0	0	0
BRCA1	synonymous_variant	NM_007294.4:c.693G>A	NP_009225.1:p.Thr231=	Likely Benign	ENIGMA		3	0	2	0	0	0
BRCA1	missense_variant	NM_007294.4:c.734A>T	NP_009225.1:p.Asp245Val	Benign	BS3_strong (Protein function similar to benign control variants (PMID:32546644))<br>BP1_strong (Located outside functional domain and SpliceAI=0.05)<br>BP5_strong (Likelihood ratio- Prior probaility of pathogenicity 0.02)		1	0	0	0	0	0
BRCA1	missense_variant	NM_007294.4:c.736T>G	NP_009225.1:p.Leu246Val	Benign	ENIGMA		5	0	3	0	2	0
BRCA1	synonymous_variant	NM_007294.4:c.75C>T	NP_009225.1:p.Pro25=	Likely Benign	ENIGMA		2	0	2	0	0	0
BRCA1	splice_region_variant	NM_007294.4:c.81-14C>T		Benign	ENIGMA		4	0	1	0	1	0
BRCA1	synonymous_variant	NM_007294.4:c.825C>T	NP_009225.1:p.Gly275=	Likely Benign	ENIGMA		3	0	2	0	1	0
BRCA1	synonymous_variant	NM_007294.4:c.981A>G	NP_009225.1:p.Thr327=	Benign	ENIGMA		3	0	2	0	0	0
BRCA2	missense_variant	NM_000059.4:c.10089A>G	NP_000050.3:p.Ile3363Met	Likely Benign	ccDK		2	0	1	0	0	0
BRCA2	in-frame_insertion	NM_000059.4:c.10094_10095insGAATTATAT	NP_000050.3:p.Val3365_Ser3366insAsnTyrIle	Likely Benign	ccDK		7	0	7	0	0	0
BRCA2	frameshift_variant	NM_000059.4:c.10095_10096insT	NP_000050.3:p.Ser3366Ter	Likely Benign	BS1_strong (GnomAD 0.00031)<br>PVS1 N/A (Frameshift variant NOT predicted to undergo NMD and role of protein function is unknown)<br>PM5_N/A (PTC in exon 27 is not relevant)		7	0	7	0	0	0
BRCA2	synonymous_variant	NM_000059.4:c.10110G>A	NP_000050.3:p.Arg3370=	Benign	ENIGMA		18	0	11	0	4	0
BRCA2	missense_variant	NM_000059.4:c.10111A>G	NP_000050.3:p.Thr3371Ala	Likely Benign	BP5_moderat (Posterior probability of pathogenicty LR 0.1796)<br>BP1_strong (Outside functional domain and SpliceAI=0.05)		1	0	1	0	0	0
BRCA2	synonymous_variant	NM_000059.4:c.10116T>C	NP_000050.3:p.Ala3372=	Likely Benign	ccDK		1	0	1	0	0	0
BRCA2	missense_variant	NM_000059.4:c.1012G>A	NP_000050.3:p.Ala338Thr	Likely Benign	ccDK		1	0	1	0	0	0
BRCA2	missense_variant	NM_000059.4:c.10199G>A	NP_000050.3:p.Ser3400Asn	Likely Benign	ccDK		1	0	0	0	0	0
BRCA2	missense_variant	NM_000059.4:c.10234A>G	NP_000050.3:p.Ile3412Val	Benign	ENIGMA		18	0	10	0	1	0
BRCA2	missense_variant	NM_000059.4:c.1151C>T	NP_000050.3:p.Ser384Phe	Benign	ENIGMA		14	0	8	0	1	0
BRCA2	missense_variant	NM_000059.4:c.1160T>C	NP_000050.3:p.Val387Ala	Likely Benign	ccDK		1	0	1	0	0	0
BRCA2	missense_variant	NM_000059.4:c.1166C>A	NP_000050.3:p.Pro389Gln	Benign	ENIGMA		1	0	1	0	0	0
BRCA2	synonymous_variant	NM_000059.4:c.1167G>A	NP_000050.3:p.Pro389=	Likely Benign	ENIGMA		2	0	1	0	1	0
BRCA2	missense_variant	NM_000059.4:c.116C>T	NP_000050.3:p.Ala39Val	Benign	ccDK		3	0	2	0	1	0
BRCA2	missense_variant	NM_000059.4:c.1247T>G	NP_000050.3:p.Ile416Ser	Benign	BP1_strong (Outside functional domain and SpliceAI=0.00)<br>BP5_strong (Posterior probability of pathogenicity 0.007 and prior probability of pathogenicity 0.02)		1	0	0	0	0	0
BRCA2	missense_variant	NM_000059.4:c.125A>G	NP_000050.3:p.Tyr42Cys	Benign	ENIGMA		20	0	13	0	4	0
BRCA2	synonymous_variant	NM_000059.4:c.1275A>G	NP_000050.3:p.Glu425=	Benign	ENIGMA		1	0	1	0	0	0
BRCA2	missense_variant	NM_000059.4:c.1342C>T	NP_000050.3:p.Arg448Cys	Likely Benign	ccDK		1	0	0	0	0	0
BRCA2	synonymous_variant	NM_000059.4:c.1354C>T	NP_000050.3:p.Leu452=	Likely Benign	ENIGMA		1	0	0	0	0	0
BRCA2	synonymous_variant	NM_000059.4:c.1362A>G	NP_000050.3:p.Lys454=	Benign	ENIGMA		1	0	1	0	0	0
BRCA2	synonymous_variant	NM_000059.4:c.1365A>G	NP_000050.3:p.Ser455=	Benign	ENIGMA		372	6	245	5	60	0
BRCA2	missense_variant	NM_000059.4:c.1385A>G	NP_000050.3:p.Glu462Gly	Benign	ENIGMA		7	0	6	0	0	0
BRCA2	synonymous_variant	NM_000059.4:c.1395A>C	NP_000050.3:p.Val465=	Likely Benign	ENIGMA		14	0	10	0	1	0
BRCA2	missense_variant	NM_000059.4:c.1460C>A	NP_000050.3:p.Ala487Glu	Benign	ccDK		11	0	8	0	2	0
BRCA2	missense_variant	NM_000059.4:c.1466C>G	NP_000050.3:p.Ser489Cys	Likely Benign	ccDK		1	0	1	0	0	0
BRCA2	synonymous_variant	NM_000059.4:c.1485T>G	NP_000050.3:p.Ala495=	Likely Benign	ccDK		1	0	1	0	0	0
BRCA2	missense_variant	NM_000059.4:c.1514T>C	NP_000050.3:p.Ile505Thr	Benign	ENIGMA		7	0	4	0	0	0
BRCA2	synonymous_variant	NM_000059.4:c.1599T>C	NP_000050.3:p.Thr533=	Likely Benign	ENIGMA		1	0	0	0	0	0
BRCA2	missense_variant	NM_000059.4:c.1662T>G	NP_000050.3:p.Cys554Trp	Benign	ENIGMA		1	0	0	0	0	0
BRCA2	missense_variant	NM_000059.4:c.167A>C	NP_000050.3:p.Asn56Thr	Benign	ENIGMA		2	0	1	0	1	0
BRCA2	missense_variant	NM_000059.4:c.1786G>C	NP_000050.3:p.Asp596His	Benign	ENIGMA		3	0	2	0	0	0
BRCA2	missense_variant	NM_000059.4:c.1792A>G	NP_000050.3:p.Thr598Ala	Benign	ENIGMA		45	0	25	0	4	0
BRCA2	missense_variant	NM_000059.4:c.179A>G	NP_000050.3:p.Asn60Ser	Likely Benign	ccDK		1	0	1	0	1	0
BRCA2	missense_variant	NM_000059.4:c.1804G>A	NP_000050.3:p.Gly602Arg	Benign	ENIGMA		6	0	4	0	1	0
BRCA2	missense_variant	NM_000059.4:c.1817C>T	NP_000050.3:p.Pro606Leu	Likely Benign	ccDK		1	0	0	0	0	0
BRCA2	synonymous_variant	NM_000059.4:c.1818G>A	NP_000050.3:p.Pro606=	Likely Benign	ENIGMA		1	0	1	0	0	0

BRCA2	missense_variant	NM_000059.4:c.1889C>T	NP_000050.3:p.Thr630Ile	Benign	ENIGMA		2	0	1	0	0	0
BRCA2	synonymous_variant	NM_000059.4:c.1938C>T	NP_000050.3:p.Ser646=	Benign	ENIGMA		7	0	4	0	2	0
BRCA2	missense_variant	NM_000059.4:c.1964C>G	NP_000050.3:p.Pro655Arg	Benign	ENIGMA		1	0	0	0	1	0
BRCA2	missense_variant	NM_000059.4:c.2072C>G	NP_000050.3:p.Ala691Gly	Likely Benign	ccDK		1	0	0	0	0	0
BRCA2	missense_variant	NM_000059.4:c.2122T>A	NP_000050.3:p.Ser708Thr	Likely Benign	ccDK		1	0	0	0	0	0
BRCA2	missense_variant	NM_000059.4:c.2125C>G	NP_000050.3:p.Leu709Val	Likely Benign	ccDK		1	0	0	0	0	0
BRCA2	synonymous_variant	NM_000059.4:c.2229T>C	NP_000050.3:p.His743=	Benign	ENIGMA		372	6	245	5	60	0
BRCA2	missense_variant	NM_000059.4:c.223G>C	NP_000050.3:p.Ala75Pro	Benign	ENIGMA		2	0	2	0	0	0
BRCA2	synonymous_variant	NM_000059.4:c.2253T>G	NP_000050.3:p.Thr751=	Likely Benign	ENIGMA		1	0	0	0	0	0
BRCA2	synonymous_variant	NM_000059.4:c.2256C>T	NP_000050.3:p.Asp752=	Likely Benign	ENIGMA		1	0	1	0	0	0
BRCA2	missense_variant	NM_000059.4:c.2306T>G	NP_000050.3:p.Leu769Arg	Likely Benign	ccDK		1	0	1	0	0	0
BRCA2	synonymous_variant	NM_000059.4:c.2538A>C	NP_000050.3:p.Ser846=	Benign	ENIGMA		6	0	4	0	0	0
BRCA2	synonymous_variant	NM_000059.4:c.2592A>G	NP_000050.3:p.Gln864=	Likely Benign	ccDK		1	0	1	0	0	0
BRCA2	synonymous_variant	NM_000059.4:c.267G>A	NP_000050.3:p.Pro89=	Likely Benign	ENIGMA		1	0	1	0	0	0
BRCA2	synonymous_variant	NM_000059.4:c.2754C>T	NP_000050.3:p.Asn918=	Likely Benign	ENIGMA		1	0	0	0	0	0
BRCA2	missense_variant	NM_000059.4:c.2803G>C	NP_000050.3:p.Asp935His	Benign	ENIGMA		1	0	1	0	0	0
BRCA2	missense_variant	NM_000059.4:c.2864A>C	NP_000050.3:p.Asn955Thr	Likely Benign	ccDK		1	0	0	0	1	0
BRCA2	missense_variant	NM_000059.4:c.2870A>G	NP_000050.3:p.Asn957Ser	Likely Benign	ccDK		2	0	2	0	0	0
BRCA2	synonymous_variant	NM_000059.4:c.2883G>A	NP_000050.3:p.Gln961=	Benign	ENIGMA		24	0	13	0	4	0
BRCA2	missense_variant	NM_000059.4:c.28A>G	NP_000050.3:p.Thr10Ala	Likely Benign	ccDK		1	0	1	0	0	0
BRCA2	synonymous_variant	NM_000059.4:c.2919G>A	NP_000050.3:p.Ser973=	Benign	ENIGMA		3	0	3	0	0	0
BRCA2	missense_variant	NM_000059.4:c.2920G>A	NP_000050.3:p.Asp974Asn	Likely Benign	ccDK		1	0	1	0	0	0
BRCA2	missense_variant	NM_000059.4:c.2971A>G	NP_000050.3:p.Asn991Asp	Benign	ENIGMA		371	6	244	5	60	0
BRCA2	missense_variant	NM_000059.4:c.3055C>G	NP_000050.3:p.Leu1019Val	Benign	ENIGMA		2	0	2	0	0	0
BRCA2	synonymous_variant	NM_000059.4:c.3264T>C	NP_000050.3:p.Pro1088=	Benign	ENIGMA		2	0	1	0	1	0
BRCA2	missense_variant	NM_000059.4:c.3302A>G	NP_000050.3:p.His1101Arg	Likely Benign	ccDK		1	0	1	0	0	0
BRCA2	missense_variant	NM_000059.4:c.3311C>T	NP_000050.3:p.Thr1104Ile	Likely Benign	ccDK		1	0	1	0	0	0
BRCA2	synonymous_variant	NM_000059.4:c.3315T>C	NP_000050.3:p.Pro1105=	Likely Benign	ccDK		1	0	1	0	0	0
BRCA2	missense_variant	NM_000059.4:c.3318C>G	NP_000050.3:p.Ser1106Arg	Likely Benign	ccDK		1	0	1	0	0	0
BRCA2	missense_variant	NM_000059.4:c.3330A>C	NP_000050.3:p.Glu1110Asp	Likely Benign	BP1_strong (Located outside functional domain and SpliceAI=0.0)		1	0	1	0	0	0
BRCA2	synonymous_variant	NM_000059.4:c.3333T>C	NP_000050.3:p.Ile1111=	Likely Benign	ccDK		1	0	1	0	0	0
BRCA2	missense_variant	NM_000059.4:c.3346A>T	NP_000050.3:p.Thr1116Ser	Likely Benign	ccDK		1	0	1	0	0	0
BRCA2	missense_variant	NM_000059.4:c.340C>A	NP_000050.3:p.His114Asn	Likely Benign	ccDK		1	0	0	0	0	0
BRCA2	missense_variant	NM_000059.4:c.3445A>G	NP_000050.3:p.Met1149Val	Benign	ccDK		1	0	1	0	0	0
BRCA2	synonymous_variant	NM_000059.4:c.3483T>C	NP_000050.3:p.Asp1161=	Likely Benign	ccDK		1	0	1	0	1	0
BRCA2	missense_variant	NM_000059.4:c.3515C>T	NP_000050.3:p.Ser1172Leu	Benign	ENIGMA		7	0	6	0	1	0
BRCA2	synonymous_variant	NM_000059.4:c.3516G>A	NP_000050.3:p.Ser1172=	Benign	ENIGMA		30	0	17	0	4	0
BRCA2	missense_variant	NM_000059.4:c.3536G>A	NP_000050.3:p.Ser1179Asn	Likely Benign	ccDK		1	0	0	0	0	0
BRCA2	missense_variant	NM_000059.4:c.3762G>T	NP_000050.3:p.Glu1254Asp	Likely Benign	ccDK		2	0	2	0	0	0
BRCA2	missense_variant	NM_000059.4:c.3835A>G	NP_000050.3:p.Asn1279Asp	Likely Benign	ccDK		1	0	0	0	0	0
BRCA2	missense_variant	NM_000059.4:c.3869G>A	NP_000050.3:p.Cys1290Tyr	Benign	ENIGMA		2	0	2	0	0	0
BRCA2	in-frame_deletion	NM_000059.4:c.3890_3892delATA	NP_000050.3:p.Asn1297del	Likely Benign	BP1_strong (Located outside functional domain and SpliceAI=0.01)		1	0	0	0	1	0
BRCA2	missense_variant	NM_000059.4:c.3910A>G	NP_000050.3:p.Thr1304Ala	Likely Benign	ccDK		1	0	0	0	1	0
BRCA2	intron_variant	NM_000059.4:c.-39-36C>G		Benign	BS1 (GnomAD frequency 0.0001907)<br>BP4 (SpliceAI=0)<br>BP5_strong (Prior probability of pathogenicity 0.02)		2	0	1	0	1	0
BRCA2	missense_variant	NM_000059.4:c.3962A>G	NP_000050.3:p.Asp1321Gly	Likely Benign	ccDK		4	0	3	0	1	0
BRCA2	synonymous_variant	NM_000059.4:c.4068G>A	NP_000050.3:p.Leu1356=	Benign	ENIGMA		71	0	45	0	8	0
BRCA2	missense_variant	NM_000059.4:c.4079A>C	NP_000050.3:p.Asp1360Ala	Likely Benign	ccDK		1	0	0	0	1	0
BRCA2	missense_variant	NM_000059.4:c.4090A>C	NP_000050.3:p.Ile1364Leu	Benign	ENIGMA		1	0	1	0	0	0
BRCA2	intron_variant	NM_000059.4:c.425+48A>G		Likely Benign	BP4 (SpliceAI=0.00)<br>BP7 (Deep intronic variant, +7/-21, and no impact on splicing)		3	0	2	0	0	0
BRCA2	missense_variant	NM_000059.4:c.4258G>T	NP_000050.3:p.Asp1420Tyr	Benign	ENIGMA		144	0	85	0	27	0
BRCA2	synonymous_variant	NM_000059.4:c.4362A>G	NP_000050.3:p.Pro1454=	Likely Benign	BP1_strong (Located outside functional domain and SpliceAI=0.0)		1	0	1	0	0	0
BRCA2	missense_variant	NM_000059.4:c.440A>G	NP_000050.3:p.Gln147Arg	Benign	ENIGMA		1	0	1	0	0	0
BRCA2	missense_variant	NM_000059.4:c.4478A>G	NP_000050.3:p.Glu1493Gly	Likely Benign	ccDK		2	0	1	0	0	0
BRCA2	missense_variant	NM_000059.4:c.4502A>G	NP_000050.3:p.Asn1501Ser	Likely Benign	ccDK		1	0	1	0	0	0
BRCA2	missense_variant	NM_000059.4:c.4534C>T	NP_000050.3:p.Arg1512Cys	Likely Benign	ccDK		1	0	1	0	0	0
BRCA2	missense_variant	NM_000059.4:c.4574A>G	NP_000050.3:p.His1525Arg	Likely Benign	ccDK		2	0	1	0	1	0
BRCA2	missense_variant	NM_000059.4:c.4585G>A	NP_000050.3:p.Gly1529Arg	Benign	ENIGMA		8	0	7	0	0	0
BRCA2	synonymous_variant	NM_000059.4:c.4614T>C	NP_000050.3:p.Ser1538=	Likely Benign	ENIGMA		2	0	2	0	0	0
BRCA2	synonymous_variant	NM_000059.4:c.4656T>C	NP_000050.3:p.Gly1552=	Likely Benign	ENIGMA		2	0	1	0	1	0
BRCA2	missense_variant	NM_000059.4:c.4681C>A	NP_000050.3:p.His1561Asn	Benign	BS1_strong (GnomAD 0.004919)<br>BP1_strong (Located outside functional domain and SpliceAI=0.0)		1	0	0	0	0	0
BRCA2	synonymous_variant	NM_000059.4:c.4686A>G	NP_000050.3:p.Gln1562=	Likely Benign	ENIGMA		2	1	0	0	2	1
BRCA2	missense_variant	NM_000059.4:c.4828G>A	NP_000050.3:p.Val1610Met	Likely Benign	ccDK		3	0	3	0	0	0
BRCA2	missense_variant	NM_000059.4:c.4898T>C	NP_000050.3:p.Ile1633Thr	Likely Benign	BP1_strong (Located outside functional domain and SpliceAI=0.00)		1	0	0	0	0	0
BRCA2	missense_variant	NM_000059.4:c.4957A>G	NP_000050.3:p.Thr1653Ala	Likely Benign	ccDK		1	0	1	0	0	0
BRCA2	synonymous_variant	NM_000059.4:c.4977C>T	NP_000050.3:p.Ser1659=	Likely Benign	ENIGMA		1	0	0	0	0	0
BRCA2	missense_variant	NM_000059.4:c.502C>A	NP_000050.3:p.Pro168Thr	Benign	ENIGMA		1	0	1	0	0	0
BRCA2	missense_variant	NM_000059.4:c.5070A>C	NP_000050.3:p.Lys1690Asn	Benign	ENIGMA		7	0	4	0	0	0
BRCA2	missense_variant	NM_000059.4:c.5113A>G	NP_000050.3:p.Ile1705Val	Likely Benign	ccDK		2	0	1	0	1	0
BRCA2	splice_region_variant	NM_000059.4:c.516+18T>C		Benign	ccDK		3	0	3	0	0	0
BRCA2	missense_variant	NM_000059.4:c.5186A>G	NP_000050.3:p.Lys1729Arg	Likely Benign	ccDK		1	0	1	0	0	0
BRCA2	synonymous_variant	NM_000059.4:c.5199C>T	NP_000050.3:p.Ser1733=	Benign	ENIGMA		79	0	50	0	9	0
BRCA2	missense_variant	NM_000059.4:c.5200G>A	NP_000050.3:p.Glu1734Lys	Likely Benign	BP1_strong (Located outside functional domain and SpliceAI=0.01)		1	0	0	0	0	0
BRCA2	missense_variant	NM_000059.4:c.5278T>G	NP_000050.3:p.Ser1760Ala	Likely Benign	ccDK		1	0	1	0	0	0
BRCA2	disruptive_in-frame_deletion	NM_000059.4:c.5303_5305delTTG	NP_000050.3:p.Leu1768_Asp1769delinsHis	Likely Benign	PM2_supporting (Absent from GnomAD)<br>BP1_strong (Located outside functinal domain and SpliceAI=0)		1	0	1	0	0	0
BRCA2	missense_variant	NM_000059.4:c.5312G>A	NP_000050.3:p.Gly1771Asp	Benign	ENIGMA		3	0	1	0	0	0
BRCA2	synonymous_variant	NM_000059.4:c.5418A>G	NP_000050.3:p.Glu1806=	Benign	ENIGMA		2	0	1	0	1	0
BRCA2	missense_variant	NM_000059.4:c.5455C>T	NP_000050.3:p.Pro1819Ser	Benign	ENIGMA		2	0	1	0	0	0
BRCA2	missense_variant	NM_000059.4:c.5596T>C	NP_000050.3:p.Phe1866Leu	Likely Benign	ccDK		3	0	1	0	1	0
BRCA2	missense_variant	NM_000059.4:c.5640T>G	NP_000050.3:p.Asn1880Lys	Benign	ENIGMA		1	0	1	0	0	0
BRCA2	missense_variant	NM_000059.4:c.5744C>T	NP_000050.3:p.Thr1915Met	Benign	ENIGMA		397	5	233	2	61	2
BRCA2	missense_variant	NM_000059.4:c.5768A>C	NP_000050.3:p.Asp1923Ala	Benign	ENIGMA		1	0	0	0	0	0
BRCA2	missense_variant	NM_000059.4:c.5785A>G	NP_000050.3:p.Ile1929Val	Benign	ENIGMA		1	0	0	0	0	0
BRCA2	missense_variant	NM_000059.4:c.5924G>A	NP_000050.3:p.Cys1975Tyr	Likely Benign	ccDK		3	0	3	0	0	0
BRCA2	missense_variant	NM_000059.4:c.5938A>C	NP_000050.3:p.Thr1980Pro	Likely Benign	ccDK		2	0	0	0	2	0
BRCA2	missense_variant	NM_000059.4:c.6100C>T	NP_000050.3:p.Arg2034Cys	Benign	ENIGMA		37	0	24	0	6	0
BRCA2	missense_variant	NM_000059.4:c.6131G>C	NP_000050.3:p.Gly2044Ala	Likely Benign	ccDK		1	0	1	0	0	0
BRCA2	missense_variant	NM_000059.4:c.6182C>T	NP_000050.3:p.Ala2061Val	Likely Benign	ccDK		2	0	1	0	1	0
BRCA2	synonymous_variant	NM_000059.4:c.6216C>T	NP_000050.3:p.Ser2072=	Likely Benign	ENIGMA		1	0	1	0	1	0
BRCA2	intron_variant	NM_000059.4:c.631+25C>T		Likely Benign	ccDK		1	0	1	0	0	0
BRCA2	missense_variant	NM_000059.4:c.6317T>C	NP_000050.3:p.Leu2106Pro	Benign	ccDK		3	0	2	0	0	0
BRCA2	missense_variant	NM_000059.4:c.6322C>T	NP_000050.3:p.Arg2108Cys	Benign	ENIGMA		2	0	2	0	0	0
BRCA2	missense_variant	NM_000059.4:c.6412G>T	NP_000050.3:p.Val2138Phe	Benign	BS1_strong (GnomAD 0.000605)<br>BP1_strong (Located outside functional domain and SpliceAI=0.01)		1	0	0	0	0	0
BRCA2	synonymous_variant	NM_000059.4:c.6444T>C	NP_000050.3:p.Ser2148=	Likely Benign	ENIGMA		1	0	0	0	0	0
BRCA2	missense_variant	NM_000059.4:c.6455C>A	NP_000050.3:p.Ser2152Tyr	Benign	ENIGMA		12	0	7	0	2	0
BRCA2	missense_variant	NM_000059.4:c.6458C>T	NP_000050.3:p.Pro2153Leu	Likely Benign	ccDK		1	0	1	0	0	0
BRCA2	missense_variant	NM_000059.4:c.6665A>G	NP_000050.3:p.Tyr2222Cys	Likely Benign	ccDK		1	0	1	0	0	0

BRCA2	missense_variant	NM_000059.4:c.6698C>A	NP_000050.3:p.Ala2233Asp	Benign	BP1_strong (Located outside functional domain and SpliceAI=0.00)<br>BP5_strong (Posterior probability of pathogenicity 0.0036 an Prior probability of<br>pathogenicity 0.02)		1	0	0	0	1	0
BRCA2	synonymous_variant	NM_000059.4:c.6699T>C	NP_000050.3:p.Ala2233=	Likely Benign	ccDK		1	0	1	0	0	0
BRCA2	missense_variant	NM_000059.4:c.6748A>G	NP_000050.3:p.Thr2250Ala	Benign	ENIGMA		2	0	0	0	1	0
BRCA2	splice_region_variant	NM_000059.4:c.682-12_682-11delTA		Likely Benign	ccDK		5	0	4	0	0	0
BRCA2	missense_variant	NM_000059.4:c.6821G>T	NP_000050.3:p.Gly2274Val	Likely Benign	ccDK		15	0	10	0	1	0
BRCA2	splice_region_variant	NM_000059.4:c.6842-20T>A		Benign	ENIGMA		2	0	1	0	0	0
BRCA2	splice_region_variant	NM_000059.4:c.68-7delT		Benign	ENIGMA		2	0	1	0	0	0
BRCA2	splice_region_variant	NM_000059.4:c.68-7dupT		Benign	ccDK		1	0	1	0	0	0
BRCA2	splice_region_variant	NM_000059.4:c.68-7T>A		Benign	ENIGMA		11	0	7	0	1	0
BRCA2	missense_variant	NM_000059.4:c.6882C>G	NP_000050.3:p.Asp2294Glu	Likely Benign	ccDK		1	0	0	0	0	0
BRCA2	missense_variant	NM_000059.4:c.6935A>T	NP_000050.3:p.Asp2312Val	Benign	ENIGMA		1	0	1	0	0	0
BRCA2	missense_variant	NM_000059.4:c.7017G>C	NP_000050.3:p.Lys2339Asn	Benign	ENIGMA		2	0	1	0	1	0
BRCA2	missense_variant	NM_000059.4:c.7102T>G	NP_000050.3:p.Leu2368Val	Likely Benign	ccDK		2	0	2	0	0	0
BRCA2	missense_variant	NM_000059.4:c.7223C>T	NP_000050.3:p.Pro2408Leu	Likely Benign	ccDK		1	0	1	0	0	0
BRCA2	missense_variant	NM_000059.4:c.7319A>G	NP_000050.3:p.His2440Arg	Benign	ENIGMA		2	0	1	0	1	0
BRCA2	splice_region_variant	NM_000059.4:c.7435+6G>A		Benign	ccDK		1	0	1	0	0	0
BRCA2	missense_variant	NM_000059.4:c.7469T>C	NP_000050.3:p.Ile2490Thr	Benign	ENIGMA		3	0	1	0	1	0
BRCA2	missense_variant	NM_000059.4:c.7544C>T	NP_000050.3:p.Thr2515Ile	Benign	ENIGMA		10	0	9	0	0	0
BRCA2	missense_variant	NM_000059.4:c.7562T>C	NP_000050.3:p.Ile2521Thr	Likely Benign	ccDK		1	0	0	0	0	0
BRCA2	synonymous_variant	NM_000059.4:c.7572A>G	NP_000050.3:p.Lys2524=	Likely Benign	ccDK		1	0	1	0	0	0
BRCA2	missense_variant	NM_000059.4:c.7601C>T	NP_000050.3:p.Ala2534Val	Likely Benign	ccDK		2	0	1	0	0	0
BRCA2	intron_variant	NM_000059.4:c.7618-22T>C		Likely Benign	ccDK		3	0	1	0	1	0
BRCA2	splice_region_variant	NM_000059.4:c.7805+6C>G		Likely Benign	BS1 (GnomAD 0.000488)<br>BP4 (SpliceAI=0.2)		1	0	0	0	0	0
BRCA2	splice_region_variant	NM_000059.4:c.794-11T>C		Likely Benign	ccDK		1	0	1	0	0	0
BRCA2	intron_variant	NM_000059.4:c.794-30T>G		Likely Benign	BP4 (SpliceAI=0.00)<br>BP7 (Deep intronic variant, +7/-21, and no impact on splicing)		1	0	1	0	0	0
BRCA2	synonymous_variant	NM_000059.4:c.7965A>G	NP_000050.3:p.Gln2655=	Likely Benign	ccDK		1	0	1	0	0	0
BRCA2	synonymous_variant	NM_000059.4:c.7992T>A	NP_000050.3:p.Ile2664=	Likely Benign	ENIGMA		6	0	4	0	1	0
BRCA2	missense_variant	NM_000059.4:c.7994A>G	NP_000050.3:p.Asp2665Gly	Benign	ENIGMA		4	0	2	0	1	0
BRCA2	synonymous_variant	NM_000059.4:c.8010G>A	NP_000050.3:p.Ser2670=	Likely Benign	ENIGMA		12	0	5	0	4	0
BRCA2	missense_variant	NM_000059.4:c.8084C>T	NP_000050.3:p.Ser2695Leu	Likely Benign	ccDK		1	0	1	0	0	0
BRCA2	missense_variant	NM_000059.4:c.8090G>A	NP_000050.3:p.Ser2697Asn	Benign	ccDK		1	0	1	0	0	0
BRCA2	missense_variant	NM_000059.4:c.8092G>A	NP_000050.3:p.Ala2698Thr	Likely Benign	ccDK		1	0	1	0	0	0
BRCA2	missense_variant	NM_000059.4:c.8149G>T	NP_000050.3:p.Ala2717Ser	Benign	ENIGMA		32	0	22	0	7	0
BRCA2	missense_variant	NM_000059.4:c.8182G>A	NP_000050.3:p.Val2728Ile	Benign	ENIGMA		63	0	40	0	11	0
BRCA2	missense_variant	NM_000059.4:c.831T>G	NP_000050.3:p.Asn277Lys	Benign	ENIGMA		1	0	1	0	0	0
BRCA2	splice_region_variant	NM_000059.4:c.8331+16C>G		Likely Benign	ccDK		2	0	2	0	0	0
BRCA2	missense_variant	NM_000059.4:c.8350C>T	NP_000050.3:p.Arg2784Trp	Likely Benign	ccDK		1	0	1	0	0	0
BRCA2	missense_variant	NM_000059.4:c.8360G>A	NP_000050.3:p.Arg2787His	Likely Benign	ccDK		1	0	1	0	0	0
BRCA2	synonymous_variant	NM_000059.4:c.8385T>C	NP_000050.3:p.Phe2795=	Likely Benign	BP4 (Located inside functional domain and SpliceAI=0.02)<br>BP7 (Silent variant located inside af functional domain)		1	1	1	1	0	0
BRCA2	splice_region_variant	NM_000059.4:c.8487+19A>G		Benign	ENIGMA		1	0	1	0	0	0
BRCA2	splice_region_variant	NM_000059.4:c.8487+8G>A		Likely Benign	ccDK		1	0	0	0	1	0
BRCA2	missense_variant	NM_000059.4:c.856T>C	NP_000050.3:p.Ser286Pro	Likely Benign	ccDK		2	0	1	0	1	0
BRCA2	splice_region_variant	NM_000059.4:c.8632+15A>G		Likely Benign	ccDK		2	0	2	0	0	0
BRCA2	splice_region_variant	NM_000059.4:c.8633-16C>G		Likely Benign	ccDK		2	0	2	0	0	0
BRCA2	missense_variant	NM_000059.4:c.865A>C	NP_000050.3:p.Asn289His	Benign	ENIGMA		373	6	245	5	60	0
BRCA2	synonymous_variant	NM_000059.4:c.8694G>A	NP_000050.3:p.Leu2898=	Likely Benign	ENIGMA		1	0	1	0	0	0
BRCA2	missense_variant	NM_000059.4:c.8850G>T	NP_000050.3:p.Lys2950Asn	Benign	ccDK		15	0	11	0	2	0
BRCA2	missense_variant	NM_000059.4:c.8851G>A	NP_000050.3:p.Ala2951Thr	Benign	ENIGMA		68	0	45	0	8	0
BRCA2	missense_variant	NM_000059.4:c.8905G>A	NP_000050.3:p.Val2969Met	Benign	ENIGMA		5	0	4	0	1	0
BRCA2	missense_variant	NM_000059.4:c.8917C>T	NP_000050.3:p.Arg2973Cys	Benign	ENIGMA		1	0	0	0	1	0
BRCA2	splice_region_variant	NM_000059.4:c.8953+16C>T		Likely Benign	ccDK		2	0	1	0	0	0
BRCA2	splice_acceptor_variant	NM_000059.4:c.8954-5_8954-2delAACA		Likely Benign	ccDK		2	0	2	0	0	0
BRCA2	missense_variant	NM_000059.4:c.9038C>T	NP_000050.3:p.Thr3013Ile	Benign	ENIGMA		2	0	1	0	0	0
BRCA2	synonymous_variant	NM_000059.4:c.909T>G	NP_000050.3:p.Ser303=	Likely Benign	ENIGMA		4	0	4	0	2	0
BRCA2	missense_variant	NM_000059.4:c.9187C>T	NP_000050.3:p.Pro3063Ser	Likely Benign	ccDK		1	0	1	0	0	0
BRCA2	intron_variant	NM_000059.4:c.9256+46A>T		Likely Benign	BP4 (SpliceAI=0.00)<br>BP7 (Deep intronic variant, +7/-21, and no impact on splicing)		1	0	1	0	0	0
BRCA2	splice_region_variant	NM_000059.4:c.9257-16T>C		Benign	ENIGMA		130	1	89	0	24	1
BRCA2	intron_variant	NM_000059.4:c.9257-49T>C		Benign	ccDK		3	0	2	0	1	0
BRCA2	splice_region_variant	NM_000059.4:c.9257-8C>T		Likely Benign	ccDK		6	0	6	0	0	0
BRCA2	missense_variant	NM_000059.4:c.9292T>C	NP_000050.3:p.Tyr3098His	Benign	ENIGMA		1	0	1	0	0	0
BRCA2	synonymous_variant	NM_000059.4:c.9411T>G	NP_000050.3:p.Thr3137=	Benign	ENIGMA		1	0	0	0	1	0
BRCA2	missense_variant	NM_000059.4:c.943T>A	NP_000050.3:p.Cys315Ser	Benign	ccDK		1	0	0	0	0	0
BRCA2	splice_region_variant	NM_000059.4:c.9501+3A>T		Benign	ENIGMA		3	0	2	0	1	0
BRCA2	splice_region_variant	NM_000059.4:c.9501+4A>G		Likely Benign	ccDK		1	0	1	0	0	0
BRCA2	intron_variant	NM_000059.4:c.9502-49A>G		Likely Benign	BP4 (SpliceAI=0.00)<br>BP7 (Deep intronic variant, +7/-21, and no impact on splicing)		1	0	0	0	1	0
BRCA2	synonymous_variant	NM_000059.4:c.9621C>A	NP_000050.3:p.Ile3207=	Likely Benign	BP1_strong (Located outside functional domain and SpliceAI=0.01)		1	0	1	0	0	0
BRCA2	missense_variant	NM_000059.4:c.964A>C	NP_000050.3:p.Lys322Gln	Likely Benign	ccDK		1	0	1	0	0	0
BRCA2	missense_variant	NM_000059.4:c.9713A>G	NP_000050.3:p.Lys3238Arg	Likely Benign	BP1_Strong (Located outside functional domain and SpliceAI=0.07)		1	0	0	0	0	0
BRCA2	missense_variant	NM_000059.4:c.971G>C	NP_000050.3:p.Arg324Thr	Likely Benign	ccDK		2	0	2	0	0	0
BRCA2	missense_variant	NM_000059.4:c.9730G>A	NP_000050.3:p.Val3244Ile	Benign	ENIGMA		2	0	1	0	1	0
BRCA2	missense_variant	NM_000059.4:c.978C>A	NP_000050.3:p.Ser326Arg	Benign	ENIGMA		18	0	11	0	1	0
BRCA2	missense_variant	NM_000059.4:c.9905G>A	NP_000050.3:p.Arg3302Lys	Benign	ccDK		1	0	1	0	0	0
BRCA2	synonymous_variant	NM_000059.4:c.9918C>A	NP_000050.3:p.Thr3306=	Likely Benign	ENIGMA		1	0	1	0	0	0
BRCA2	stop_gained	NM_000059.4:c.9976A>T	NP_000050.3:p.Lys3326Ter	Benign	ENIGMA		130	1	89	0	24	1
BRIP1	intron_variant	NM_032043.3:c.1628+24A>G		Likely Benign	BP4 (SpliceAI=0.00)<br>BP7 (Deep intron variant, +7/-21, and no impact on splicing)		5	0	4	0	1	0
BRIP1	intron_variant	NM_032043.3:c.205+34C>T		Likely Benign	BP4 (SpliceAI=0.00)<br>BP7 (Deep intron variant, +7/-21, and no impact on splicing)		4	0	3	0	0	0
BRIP1	intron_variant	NM_032043.3:c.206-21T>C		Likely Benign	BP4 (SpliceAI=0.00)<br>BP7 (Deep intronic variant, +7/-21, and no impact on splicing)<br>BP6 (Laboratories reported it benign in ClinVar)		1	0	0	0	0	0
BRIP1	intron_variant	NM_032043.3:c.2097+26delT		Likely Benign	BP4 (SpliceAI=0.00)<br>BP7 (Deep intron variant, +7/-21, and no impact on splicing)		45	0	25	0	9	0
BRIP1	splice_region_variant	NM_032043.3:c.2097+7G>A		Likely Benign	ccDK		73	0	45	0	8	0
BRIP1	missense_variant	NM_032043.3:c.2220G>T	NP_114432.2:p.Gln740His	Likely Benign	ccDK		4	0	4	0	2	0
BRIP1	missense_variant	NM_032043.3:c.2236A>G	NP_114432.2:p.Ile746Val	Likely Benign	BP4 (Revel=0.164 and BayesDel=-0.355)<br>BP6 (Laboratories reported it benign in ClinVar)		2	1	1	0	0	0
BRIP1	synonymous_variant	NM_032043.3:c.2286T>C	NP_114432.2:p.Arg762=	Likely Benign	BP4 (SpliceAI=0)<br>BP7 (Silent variant and no impact on splicing)		1	0	1	0	0	0
BRIP1	splice_region_variant	NM_032043.3:c.-30-3T>C		Likely Benign	BP4 (SpliceAI=0)<br>BP6 (GeneDx reported it benign in ClinVar)		9	0	4	0	3	0
BRIP1	intron_variant	NM_032043.3:c.-31+22G>C		Likely Benign	BP4 (SpliceAI=0)<br>BP7 (Deep intron variant, +7/-21, and no impact on splicing)		44	0	25	0	6	0
BRIP1	intron_variant	NM_032043.3:c.-31+46C>T		Likely Benign	BP4 (SpliceAI=0)<br>BP7 (Deep intron variant, +7/-21, and no impact on splicing)<br>PM2_supporting (Only 5 in GnomAD)		2	0	2	0	0	0

BRIP1	synonymous_variant	NM_032043.3:c.312T>G	NP_114432.2:p.Thr104=	Likely Benign	BP4 (SpliceAI=0.01)<br>BP7 (Silent variant with no impact on splicing)		1	0	1	0	0	0
BRIP1	synonymous_variant	NM_032043.3:c.3459T>C	NP_114432.2:p.Asp1153=	Likely Benign	ccDK		13	0	11	0	1	0
BRIP1	synonymous_variant	NM_032043.3:c.36G>T	NP_114432.2:p.Gly12=	Likely Benign	BP4 (SpliceAI=0.00)<br>BP7 (Silent variant with no impact on splicing)		3	0	1	0	2	0
BRIP1	splice_region_variant	NM_032043.3:c.380-17dupT		Likely Benign	BP6 (Laboratories reported it benign in ClinVar)<br>BP4 (SpliceAI=0)		4	0	2	0	1	0
BRIP1	intron_variant	NM_032043.3:c.380-28G>A		Likely Benign	BP4 (SpliceAI=0.01)<br>BP5 (Reported by laboratories as benign in ClinVar)<br>BP7 (Deep intronic variant, +7/-21, and no impact on splicing)		221	3	148	3	32	0
BRIP1	missense_variant	NM_032043.3:c.430G>A	NP_114432.2:p.Ala144Thr	Likely Benign	BP6 (Laboratories reported it benign in ClinVar)<br>BP4 (Revel=0.14 and BayesDel=-0.299)		3	0	2	0	0	0
BRIP1	missense_variant	NM_032043.3:c.517C>T	NP_114432.2:p.Arg173Cys	Likely Benign	ccDK		60	0	37	0	6	0
BRIP1	missense_variant	NM_032043.3:c.577G>A	NP_114432.2:p.Val193Ile	Benign	ccDK		54	0	39	0	8	0
BRIP1	missense_variant	NM_032043.3:c.584T>C	NP_114432.2:p.Leu195Pro	Likely Benign	ccDK		4	0	3	0	1	0
BRIP1	missense_variant	NM_032043.3:c.890A>G	NP_114432.2:p.Lys297Arg	Likely Benign	ccDK		17	0	10	0	4	0
BRIP1	splice_region_variant	NM_032043.3:c.94-18T>G		Likely Benign	BP4 (SpliceAI=0.02)<br>BP6 (Laboratories reported it benign in ClinVar)		6	0	4	0	0	0
CDH1	missense_variant	NM_004360.5:c.1019C>T	NP_004351.1:p.Thr340Met	Likely Benign	BS2 (The expert panel reports that the variant has been observed in at least 10 (14) individuals without DGC, SRC tumours or LBC and whose families do not suggest HDGC (BS2; SCV000254803.10, SCV000184790.6))		3	0	2	0	0	0
CDH1	splice_region_variant	NM_004360.5:c.1137+20G>C		Likely Benign	PM2_supporting (GnomAD less than 0.00001)<br>BP4 (SpliceAI=0.00, Natural splice site MaxEntScan is 7.31 and does not change and SSF is 78.39 and does not change)<br>BP7 (Deep intronic variants, +7/-21, and no impact on splicing)		1	0	0	0	1	0
CDH1	synonymous_variant	NM_004360.5:c.1161C>T	NP_004351.1:p.Asn387=	Likely Benign	BP4 (SpliceAI=0.00. Natural splice site MaxEntScan is 7.38 and does not change. SSF is 88.72 and does not change)<br>BP7 (Silent variant and no impact on splicing)		1	0	1	0	0	0
CDH1	synonymous_variant	NM_004360.5:c.1272C>T	NP_004351.1:p.Val424=	Likely Benign	ccDK		20	0	13	0	3	0
CDH1	missense_variant	NM_004360.5:c.1298A>G	NP_004351.1:p.Asp433Gly	Benign	ccDK		1	0	1	0	0	0
CDH1	synonymous_variant	NM_004360.5:c.1308G>A	NP_004351.1:p.Leu436=	Likely Benign	BP2 (In GnomAD 6 are homozygot)<br>BP4 (SpliceAI=0.00. Natural splice site MaxEntScan is 7.81 and does not change. SSF is 79.64 and does not change)<br>BP7 (Silent variant and no impact on splicing)		1	0	1	0	0	0
CDH1	synonymous_variant	NM_004360.5:c.1353T>C	NP_004351.1:p.Ile451=	Likely Benign	BP4 (SpliceAI=0.00. Natural splice site MaxEntScan is 10.24 and does not change. SSF is 88.10 and does not change)<br>BP7 (Silent variant and no impact on splicing)		1	0	1	0	0	0
CDH1	synonymous_variant	NM_004360.5:c.1356A>G	NP_004351.1:p.Leu452=	Likely Benign	BP4_supporting (SpliceAI=0.00. Natural splice site MaxEntScan is 10.24 and does not change. SSF is 88.10 and does not change)<br>BP7_supporting (Silent variant and no impact on splicing)<br>PM2_supporting (GnomAD less than 0.00001)		1	0	1	0	0	0
CDH1	synonymous_variant	NM_004360.5:c.1392C>T	NP_004351.1:p.Val464=	Likely Benign	BP4 (SpliceAI=0, MaxEntScan and SSF for natural splice does not change)<br>BP7 (Silent variant and does not impact splicing)		1	0	1	0	0	0
CDH1	synonymous_variant	NM_004360.5:c.1419C>T	NP_004351.1:p.Val473=	Likely Benign	PM2_supporting (GnomAD only 1)<br>BP4 (SpliceAI=0. MaxEntScan and SSF for natural splice does not change)<br>BP7 (Silent variant and does not impact splicing)		1	0	0	0	0	0
CDH1	intron_variant	NM_004360.5:c.164-31468T>C		Likely Benign	BS1 (GnomAD frequency 0.00197)		18	0	9	0	4	0
CDH1	missense_variant	NM_004360.5:c.164T>G	NP_004351.1:p.Val55Gly	Likely Benign	ccDK		8	0	6	0	2	0
CDH1	synonymous_variant	NM_004360.5:c.1689C>T	NP_004351.1:p.Ala563=	Likely Benign	BP4_supporting (SpliceAI=0.00. Natural splice site MaxEntScan is 9.33 and does not change. SSF is 86.16 and does not change)<br>BP7_supporting (Silent variant and does not impact splicing)		2	0	2	0	0	0
CDH1	intron_variant	NM_004360.5:c.1712-37T>G		Likely Benign	BP4 (SpliceAI=0.00. Natural splice site MaxEntScan is 10.72 and does not change. SSF is 87.52 and does not change.)<br>BP7 (Deep intronic variants, +7/-21, and no impact on splicing.<br>BP2 (10 homozygote in GnomAD)		2	0	0	0	2	0
CDH1	synonymous_variant	NM_004360.5:c.1744C>T	NP_004351.1:p.Leu582=	Likely Benign	ccDK		22	0	13	0	3	0
CDH1	intron_variant	NM_004360.5:c.1937-25C>A		Likely Benign	ccDK		1	0	0	0	1	0
CDH1	missense_variant	NM_004360.5:c.2104G>A	NP_004351.1:p.Glu702Lys	Likely Benign	BP2_supporting (Homozygot in GnomAD)<br>BS2_supporting (2 persons with the variant (PMID: 28135145) and at least one in the control group (PMID: 36243179))		2	0	0	0	0	0
CDH1	synonymous_variant	NM_004360.5:c.213C>T	NP_004351.1:p.Leu71=	Likely Benign	BP4_supporting (SpliceAI=0.00. Natural splice site MaxEntScan is 8.17 and does not change. SSF is 86.52 and does not change)<br>BP7_supporting (Silent variant and no impact on splicing)		1	0	0	0	0	0
CDH1	missense_variant	NM_004360.5:c.2204C>T	NP_004351.1:p.Ala735Val	Likely Benign	BS2 (The expert panel reports that the variant has been observed in >10 (188) individuals without a diagnosis of diffuse gastric cancer, signet ring tumor or lobular breast cancer and whose family histories do not suggest HDGC (BS2; PMID: 26898890; ClinVar: SCVs: SCV000186555.7, SCV000260700.10, SCV000210926.16; internal lab contributors))		1	0	0	0	0	0
CDH1	missense_variant	NM_004360.5:c.2329G>A	NP_004351.1:p.Asp777Asn	Likely Benign	ccDK		13	0	4	0	1	0
CDH1	splice_region_variant	NM_004360.5:c.2439+10C>T		Likely Benign	ccDK		32	0	20	0	3	0
CDH1	splice_region_variant	NM_004360.5:c.2440-6C>G		Benign	ccDK		19	1	13	0	0	0
CDH1	missense_variant	NM_004360.5:c.2512A>G	NP_004351.1:p.Ser838Gly	Likely Benign	ccDK		2	0	1	0	0	0
CDH1	missense_variant	NM_004360.5:c.2635G>A	NP_004351.1:p.Gly879Ser	Likely Benign	ccDK		10	0	6	0	2	0
CDH1	missense_variant	NM_004360.5:c.2638G>A	NP_004351.1:p.Glu880Lys	Benign	The ClinGen Expert panel reviewed this variant using:<br>BS1 (This variant is present at a maximum frequency of 0.001153 in the East Asian subpopulation in gnomAD)<br>BS2 (This variant has also been observed in 112 individuals without DGC, LBC or SRC tumours and whose families do not suggest HDGC; internal laboratory contributors).		1	0	1	0	0	0
CDH1	synonymous_variant	NM_004360.5:c.27G>A	NP_004351.1:p.Ser9=	Likely Benign	BP4_supporting (SpliceAI=0.00. Natural splice site MaxEntScan is 8.63 and does not change. SSF is 70.74 and does not change)<br>BP7_supporting (Silent variant and no impact on splicing)		6	0	3	0	1	0
CDH1	missense_variant	NM_004360.5:c.304G>A	NP_004351.1:p.Ala102Thr	Benign	ccDK		1	0	1	0	0	0
CDH1	synonymous_variant	NM_004360.5:c.324A>G	NP_004351.1:p.Arg108=	Benign	ccDK		2	0	1	0	0	0
CDH1	synonymous_variant	NM_004360.5:c.33G>C	NP_004351.1:p.Leu11=	Likely Benign	ccDK		6	0	6	0	0	0
CDH1	synonymous_variant	NM_004360.5:c.375C>T	NP_004351.1:p.Pro125=	Likely Benign	BP4_supporting (SpliceAI=0.00. Natural splice site MaxEntScan is 9.80 and does not changes. SSF is 89.83 and does not change)<br>BP7_supporting (Silent variant and no impact on splicing)<br>PM2_supporting (GnomAD less than 0.00001)		1	0	1	0	0	0
CDH1	synonymous_variant	NM_004360.5:c.381C>T	NP_004351.1:p.Pro127=	Likely Benign	PM2_supporting (GnomAD only 1)<br>BP4 (SpliceAI=0, MaxEntScan and SSF for natural splice does not change)<br>BP7 (Silent variant and do not impact splicing)		1	0	0	0	0	0
CDH1	intron_variant	NM_004360.5:c.387+27C>T		Likely Benign	BP4 (SpliceAI=0.00. Natural splice site MaxEntScore is 9.8 and does nont change. SSF is 89.83 and does not change)<br>BP7 (Deep intronic variants, +7/-21, and no impact on splicing)<br>BP2_supporting (Homozygote in GnomAD)		8	0	4	0	1	0
CDH1	synonymous_variant	NM_004360.5:c.393C>T	NP_004351.1:p.Ser131=	Likely Benign	BP4_supporting (SpliceAI=0.01. Natural splice site MaxEntScan is 12.20 and does not change. SSF is 91.37 and does not change)<br>BP7_supporting (Silent variant and no impact on splicing)		2	0	0	0	0	0
CDH1	in-frame_insertion	NM_004360.5:c.41_46dupTGCTGC	NP_004351.1:p.Leu14_Leu15dup	Likely Benign	The ClinGen expert panel reviewed this variant using:<br>PM2_supporting (This variant has a frequency of 0.0008% in GnomAD)<br>BS2 (This variant has been identified in at least ten individuals without DGC, SRC tumours and LBC and whose families do not suggest HDGC (SCV000569203.4, SCV000186596.5, SCV000545430.4))		2	0	1	0	1	0
CDH1	intron_variant	NM_004360.5:c.48+34T>C		Likely Benign	BP4 (SpliceAI=0.00; Natural splice site MaxEntScan is 8.63 and no impact on splicing. SSF is 70.74 and does not change)<br>BP7 (Deep intronic variants, +7/-21, and no impact on splicing)		1	0	0	0	0	0

CDH1	missense_variant	NM_004360.5:c.671G>A	NP_004351.1:p.Arg224His	Benign	ccDK		6	0	4	0	0	0
CDH1	intron_variant	NM_004360.5:c.687+30C>T		Likely Benign	BP4 (SpliceAI=0.00. Natural splice site MaxEntScore is 8.59 and does not change. SSF is 78.16 and does not change.)<br>BP7 (Deep intronic variants, +7/-21, and no impact on splicing)		2	0	1	0	0	0
CDH1	synonymous_variant	NM_004360.5:c.699C>T	NP_004351.1:p.His233=	Likely Benign	BP2 (Seen 1 time i homozygote in GnomAD)<br>BP4 (SpliceAI=0, MaxEntScan and SSF for natural splice does not change)<br>BP7 (Silent variant and do not impact splicing)		1	0	1	0	0	0
CDH1	synonymous_variant	NM_004360.5:c.768T>C	NP_004351.1:p.Asn256=	Likely Benign	BP4_supporting (SpliceAI=0.07. Natural splice site MaxEntScan is 7.88 and does not change. SSF is 77.67 and does not change.)<br>BP7_supporting (Silent variant and no impact on splicing)<br>PM2_supporting (GnomAD less than 0.00001)		1	0	1	0	0	0
CDH1	missense_variant	NM_004360.5:c.808T>G	NP_004351.1:p.Ser270Ala	Benign	BA1 (The expert panel reports that the variant has a maximum subpopulation frequency of 0.002269 (0.2269%, 57 of 25124 alleles) in the European (Finnish) subpopulation of the gnomAD v2.1.1 cohort)		4	0	2	0	0	0
CDH1	synonymous_variant	NM_004360.5:c.84C>T	NP_004351.1:p.Cys28=	Likely Benign	BP4_supporting (SpliceAI=0.01. Natural splice site MaxEntScan is 10.28 and does not change. SSF is 81.95 and does not change)<br>BP7_supporting (Silent variant and no impact on splicing)		2	0	2	0	1	0
CDH1	missense_variant	NM_004360.5:c.866C>T	NP_004351.1:p.Ala289Val	Likely Benign	ccDK		3	0	1	0	0	0
CDH1	synonymous_variant	NM_004360.5:c.867G>A	NP_004351.1:p.Ala289=	Likely Benign	BP2 (Seen 1 time as homozygote in GnomAD)<br>BP4 (SpliceAI=0, MaxEntScan and SSF for natural splice does not change)<br>BP7 (Silent variant and do not impact splicing)		1	0	0	0	0	0
CDH1	synonymous_variant	NM_004360.5:c.870C>T	NP_004351.1:p.Asp290=	Likely Benign	BP4 (SpliceAI=0, MaxEntScan and SSF for natural splice does not change)<br>BP7 (Silent variant and do not impact splicing)<br>PM2_supporting (GnomAD frequency 0.00000682)		1	0	1	0	0	0
CDH1	synonymous_variant	NM_004360.5:c.879G>A	NP_004351.1:p.Val293=	Likely Benign	BP4_supporting (SpliceAI=0.06. Natural splice site MaxEntScan is 8.10 and does not change and SSF is 87.55 and does not change.)<br>BP7_supporting (Silent variant and no impact on splicing)<br>PM2_supporting (GnomAD less than 0.00001)		2	0	2	0	0	0
CDH1	missense_variant	NM_004360.5:c.892G>A	NP_004351.1:p.Ala298Thr	Benign	ccDK		4	0	3	0	0	0
CDH1	synonymous_variant	NM_004360.5:c.894C>T	NP_004351.1:p.Ala298=	Likely Benign	BP4 (SpliceAI=0. MaxEntScan and SSF for natural splice does not change)<br>BP7 (Silent variant and do not impact splicing)		1	0	1	0	0	0
CDH1	missense_variant	NM_004360.5:c.8C>G	NP_004351.1:p.Pro3Arg	Likely Benign	BS2 (This variant was observed more than 70 probands without a personal history of DGC, SRC tumours or LBC and whose families do not meet HDGC clinical criteria (SCV000210891.12, SCV000186617.6))		3	0	3	0	0	0
CDH1	synonymous_variant	NM_004360.5:c.933C>G	NP_004351.1:p.Leu311=	Benign	BA1 (In ClinVar the ClinGen expert panel reports that the variant has an allele frequency of 0.03128 in the African subpopulation of the gnomAD cohort)<br>BP2 (Observed in homozygote count in GnomAD)<br>BP4 (SpliceAI=0, MaxEntScan and SSF for natural splice does not change)<br>BP7 (Silent variant and do not impact splicing)		1	0	1	0	0	0
CDH1	synonymous_variant	NM_004360.5:c.987C>T	NP_004351.1:p.Val329=	Likely Benign	PM2_supporting (Absent from GnomAD)<br>BP4 (SpliceAI=0, MaxEntScan and SSF for natural splice does not change)<br>BP7 (Silent variant and do not impact splicing)		1	0	1	0	0	0
CHEK2	synonymous_variant	NM_007194.4:c.1128A>G	NP_009125.1:p.Gly376=	Likely Benign	BP4 (SpliceAI=0.01)<br>BP7 (Silent variant with no impact on splicing)		1	0	1	0	0	0
CHEK2	missense_variant	NM_007194.4:c.1133C>T	NP_009125.1:p.Thr378Ile	Likely Benign	BP4 (Revel=0.123 and BayesDel=-0.169)<br>BS3 (Functional study showing no damanging effect (PMID: 30851065))		2	0	2	0	0	0
CHEK2	synonymous_variant	NM_007194.4:c.1176G>A	NP_009125.1:p.Ala392=	Likely Benign	BP4 (SpliceAI=0)<br>BP7 (Silent variant and no impact on splicing)<br>BP6 (Laboratories reported it benign in ClinVar)		1	0	0	0	0	0
CHEK2	missense_variant	NM_007194.4:c.1525C>T	NP_009125.1:p.Pro509Ser	Likely Benign	BS3 (Functional study showing no damaging effekt (PMID: 31050813 and PMID: 34903604))<br>BP4 (Revel=0.088 and BayesDel=-0.446)		5	0	5	0	0	0
CHEK2	missense_variant	NM_007194.4:c.1561C>T	NP_009125.1:p.Arg521Trp	Likely Benign	BS3 (Functional study showing benign result (PMID: 30851065))<br>BP4 (Revel=0.205 and BayesDel=-0.0743)		2	0	2	0	1	0
CHEK2	missense_variant	NM_007194.4:c.157T>A	NP_009125.1:p.Ser53Thr	Likely Benign	BP4 (Revel=0.17 and BayesDel=-0.0275)<br>BS3 (Variant assesed functional acording to CHEK2-complementation (PMID: 37449874))		1	0	0	0	1	0
CHEK2	synonymous_variant	NM_007194.4:c.1581C>T	NP_009125.1:p.Ala527=	Likely Benign	BP4 (SpliceAI=0.00)<br>BP7 (Silent variant with no impact on splicing)		3	0	2	0	1	0
CHEK2	synonymous_variant	NM_007194.4:c.252A>G	NP_009125.1:p.Glu84=	Likely Benign	BP4 (SpliceAI=0.00)<br>BP6 (Laboratories reported it benign in ClinVar)<br>BP7 (Silent variant with no impact on splicing)		287	7	171	4	39	1
CHEK2	intron_variant	NM_007194.4:c.320-32A>G		Likely Benign	BP4 (SpliceAI=0.00)<br>BP7 (Deep intronic variant, +7/-21, and no impact on splicing)		1	0	1	0	0	0
CHEK2	splice_region_variant	NM_007194.4:c.320-5T>A		Likely Benign	BP4 (SpliceAI=0.02)<br>BP6 (Laboratories reported it benign at ClinVar)		14	0	10	0	3	0
CHEK2	intron_variant	NM_007194.4:c.444+24C>T		Likely Benign	BP4 (SpliceAI=0.00)<br>BP7 (Deep intronic variant, +7/-21, and no impact on splicing)		30	1	22	1	7	0
CHEK2	synonymous_variant	NM_007194.4:c.474A>G	NP_009125.1:p.Ala158=	Likely Benign	BP4 (SpliceAI=0.01)<br>BP6 (Laboratories reported it benign in ClinVar)<br>BP7 (Silent variant with no impact on splicing)		2	0	1	0	2	0
CHEK2	missense_variant	NM_007194.4:c.480A>G	NP_009125.1:p.Ile160Met	Benign	BS3 (Functional study showing no damaging effect (PMID: 30851065))<br>BS4 (Lack of segregation in family - 3 family members with the variant but not affected (PMID: 22419737))		4	0	4	0	0	0
CHEK2	synonymous_variant	NM_007194.4:c.489C>T	NP_009125.1:p.His163=	Likely Benign	BP4 (SpliceAI=0.02)<br>BP7 (Silent variant with no impact on splicing)		1	0	1	0	0	0
CHEK2	synonymous_variant	NM_007194.4:c.528G>C	NP_009125.1:p.Gly176=	Likely Benign	BP4 (SpliceAI=0)<br>BP7 (Silent variant and no impact on splicing)		1	0	1	0	0	0
CHEK2	missense_variant	NM_007194.4:c.538C>T	NP_009125.1:p.Arg180Cys	Likely Benign	BS3 (Two functional studies showing no damaging effect (PMID: 30851065 and PMID: 31050813))<br>BP6 (Laboratories reported it benign in ClinVar)		46	0	23	0	6	0
CHEK2	intron_variant	NM_007194.4:c.592+50A>T		Likely Benign	BP4 (SpliceAI=0.04)<br>BP7 (Deep intronic variant, +7/-21, and no impact on splicing)		23	0	8	0	5	0
CHEK2	splice_region_variant	NM_007194.4:c.593-14C>T		Likely Benign	BP4 (SpliceAI=0.00)<br>BP6 (Laboratories reported it benign in ClinVar)		4	0	3	0	2	0
CHEK2	intron_variant	NM_007194.4:c.792+39C>T		Likely Benign	BP4 (SpliceAI=0.00)<br>BP7 (Deep intronic variant, +7/-21, and no impact on splicing)		10	0	8	0	0	0
CHEK2	missense_variant	NM_007194.4:c.7C>T	NP_009125.1:p.Arg3Trp	Likely Benign	BS3 (Functional studies showing no damaging effect (PMID: 30851065 and PMID: 31050813))<br>BP6 (Invitae reported it benign in 2024, Jan in ClinVar)		1	0	0	0	0	0
CHEK2	missense_variant	NM_007194.4:c.931G>A	NP_009125.1:p.Asp311Asn	Likely Benign	BP4 (Revel=0.112 and BayesDel=-0.49)<br>BS3 (Fundtionel study showing no damaing effect (PMID: 30851065))		1	0	1	0	0	0
PALB2	missense_variant	NM_024675.4:c.1000T>G	NP_078951.2:p.Tyr334Asp	Likely Benign	ccDK		1	0	1	0	0	0
PALB2	missense_variant	NM_024675.4:c.1001A>G	NP_078951.2:p.Tyr334Cys	Likely Benign	ccDK		1	0	1	0	0	0
PALB2	synonymous_variant	NM_024675.4:c.1119G>A	NP_078951.2:p.Glu373=	Likely Benign	ccDK		1	0	0	0	0	0
PALB2	synonymous_variant	NM_024675.4:c.1170T>A	NP_078951.2:p.Ser390=	Likely Benign	ccDK		2	0	2	0	1	0
PALB2	missense_variant	NM_024675.4:c.1178A>G	NP_078951.2:p.Lys393Arg	Likely Benign	BP1 (Missense)<br>BP4 (SpliceAI=0.00)		1	0	0	0	0	0
PALB2	missense_variant	NM_024675.4:c.1250C>A	NP_078951.2:p.Ser417Tyr	Likely Benign	ccDK		1	0	1	0	0	0
PALB2	synonymous_variant	NM_024675.4:c.1272C>T	NP_078951.2:p.Ala424=	Likely Benign	BP4 (SpliceAI=0.00)<br>BP7_supporting (Silent variant with no impact on splicing)		1	0	0	0	0	0
PALB2	synonymous_variant	NM_024675.4:c.1419A>C	NP_078951.2:p.Pro473=	Likely Benign	BP4 (SpliceAI=0)<br>BP7 (Silent variant and no impact on splicing)<br>BS1 (GnomAD frequency 0.000592)		1	0	1	0	0	0
PALB2	synonymous_variant	NM_024675.4:c.1470C>T	NP_078951.2:p.Pro490=	Likely Benign	ccDK		9	0	7	0	0	0
PALB2	synonymous_variant	NM_024675.4:c.1476G>T	NP_078951.2:p.Gly492=	Likely Benign	ccDK		1	0	0	0	1	0

PALB2	synonymous_variant	NM_024675.4:c.1606C>T	NP_078951.2:p.Leu536=	Benign	ccDK		1	0	1	0	0	0
PALB2	missense_variant	NM_024675.4:c.1697G>A	NP_078951.2:p.Arg566His	Likely Benign	BP4 (SpliceAI=0.00)<br>BP1_supporting (Missense)		2	0	1	0	0	0
PALB2	synonymous_variant	NM_024675.4:c.1872T>C	NP_078951.2:p.Leu624=	Likely Benign	ccDK		2	0	2	0	0	0
PALB2	synonymous_variant	NM_024675.4:c.1917G>A	NP_078951.2:p.Glu639=	Likely Benign	BP7 (Silent variant and no impact on splicing)<br>BP4 (SpliceAI=0.00)		1	0	1	0	0	0
PALB2	synonymous_variant	NM_024675.4:c.1923A>G	NP_078951.2:p.Lys641=	Likely Benign	BP7 (Silent variant with no impact on splicing)<br>BP4 (SpliceAI=0.00)		1	0	1	0	0	0
PALB2	missense_variant	NM_024675.4:c.1925T>C	NP_078951.2:p.Met642Thr	Likely Benign	BP1 (Missense variant)<br>BP4 (SpliceAI=0.00)		2	0	0	0	1	0
PALB2	missense_variant	NM_024675.4:c.194C>T	NP_078951.2:p.Pro65Leu	Likely Benign	BP4_supporting (SpliceAI=0.00)<br>BP1 (Missense variant)		2	0	1	0	1	0
PALB2	intron_variant	NM_024675.4:c.211+23T>C		Likely Benign	BP4 (SpliceAI=0.00)<br>BP7 (Deep intronic variant beyond, +7/-21, and no impact on splicing)		1	0	1	0	0	0
PALB2	missense_variant	NM_024675.4:c.2135C>T	NP_078951.2:p.Ala712Val	Likely Benign	ccDK		15	0	14	0	1	0
PALB2	missense_variant	NM_024675.4:c.2155C>T	NP_078951.2:p.Pro719Ser	Likely Benign	BP1 (Missense variant)<br>BP4 (SpliceAI=0.02)		1	0	1	0	0	0
PALB2	missense_variant	NM_024675.4:c.2230G>A	NP_078951.2:p.Glu744Lys	Likely Benign	BP1 (Missense variant)<br>BP4 (SpliceAI=0.03)		1	0	1	0	0	0
PALB2	synonymous_variant	NM_024675.4:c.2244A>G	NP_078951.2:p.Thr748=	Likely Benign	ccDK		4	0	3	0	0	0
PALB2	missense_variant	NM_024675.4:c.2325A>C	NP_078951.2:p.Gln775His	Likely Benign	BP1 (Missense variant)<br>BP4 (SpliceAI=0.0)		1	0	0	0	1	0
PALB2	missense_variant	NM_024675.4:c.232G>A	NP_078951.2:p.Val78Ile	Likely Benign	ccDK		4	0	2	0	1	0
PALB2	synonymous_variant	NM_024675.4:c.2379C>T	NP_078951.2:p.Gly793=	Likely Benign	BP7_strong (Silent variant and 2 functional studies showing no effect on splicing (PMID: 35806449 and PMID: 31642931))	SpliceAI DG: 0.66. Possibly partiel exon deletion. RAW score naturel donor site changes from 0.98 to 0.95 and alternative donor site changes from 0 to 0.66.	2	0	0	0	2	0
PALB2	synonymous_variant	NM_024675.4:c.2505C>T	NP_078951.2:p.Ser835=	Likely Benign	BP4 (Splice Al 0.01)<br>BP7 (Silent variant and no implact on splicing)		1	0	1	0	0	0
PALB2	intron_variant	NM_024675.4:c.2515-46A>G		Likely Benign	BS1 (GnomAD 0.0001596)<br>BP4 (SpliceAI=0.00)<br>BP7 (Deep intronic variant beyond, +7/-21, and no impact on splicing)		2	0	1	0	1	0
PALB2	intron_variant	NM_024675.4:c.2587-59T>G		Likely Benign	BS1 (GnomAD frequency 0.0002624)		1	0	0	0	0	0
PALB2	splice_region_variant	NM_024675.4:c.2749-18C>T		Likely Benign	ccDK		17	0	9	0	2	0
PALB2	missense_variant	NM_024675.4:c.2798G>C	NP_078951.2:p.Cys933Ser	Likely Benign	BP1 (Missense variant)<br>BP4 (SpliceAI=0.02)		1	0	1	0	0	0
PALB2	synonymous_variant	NM_024675.4:c.2802A>G	NP_078951.2:p.Val934=	Likely Benign	BP4_supporting (SpliceAI=0.01)<br>BP7 (Silent variant and no impact on splicing)		1	0	0	0	0	0
PALB2	splice_region_variant	NM_024675.4:c.2834+16G>T		Likely Benign	ccDK		2	0	1	0	0	0
PALB2	intron_variant	NM_024675.4:c.2834+28T>C		Likely Benign	ccDK		5	0	5	0	3	0
PALB2	missense_variant	NM_024675.4:c.2851T>C	NP_078951.2:p.Ser951Pro	Likely Benign	BS1 (GnomAD 0.000131)<br>BP1 (Missense variant)		5	0	4	0	1	0
PALB2	intron_variant	NM_024675.4:c.2997-34A>C		Likely Benign	BS1 (GnomAD 0.0005355)<br>BP4 (SpliceAI=0.00)<br>BP7 (Deep intronic variant,+7/-21, and no impact on splicing)		2	0	1	0	1	0
PALB2	missense_variant	NM_024675.4:c.3054G>C	NP_078951.2:p.Glu1018Asp	Likely Benign	BS1 (GnomAD 0.00013258)<br>BP1_supporting (Missense variant)		1	0	1	0	0	0
PALB2	missense_variant	NM_024675.4:c.3056T>C	NP_078951.2:p.Val1019Ala	Likely Benign	ccDK		1	0	1	0	0	0
PALB2	missense_variant	NM_024675.4:c.3146T>G	NP_078951.2:p.Met1049Arg	Likely Benign	BP1 (Missense variant)<br>BP4 (SpliceAI=0.04)		1	0	1	0	0	0
PALB2	synonymous_variant	NM_024675.4:c.3276C>T	NP_078951.2:p.Leu1092=	Likely Benign	BP4 (SpliceAI=0)<br>BP7 (Silent variant and no impact on splicing)		1	0	0	0	1	0
PALB2	missense_variant	NM_024675.4:c.3428T>A	NP_078951.2:p.Leu1143His	Likely Benign	BS1 (GnomAD frequency 0.000145)<br>BP1 (Missense variant)		1	0	1	0	0	0
PALB2	synonymous_variant	NM_024675.4:c.3432C>T	NP_078951.2:p.Leu1144=	Likely Benign	BP4 (SpliceAI=0)<br>BP7 (Silent variant and no impact on splicing)		1	0	1	0	0	0
PALB2	intron_variant	NM_024675.4:c.48+79G>A		Likely Benign	BS1 (GnomAD frequency 0.0001236)<br>BP4 (SpliceAI=0.0)<br>BP7 (Deep intronic variant,+7/-21, and no impact on splicing)		2	0	0	0	0	0
PALB2	missense_variant	NM_024675.4:c.656A>G	NP_078951.2:p.Asp219Gly	Likely Benign	ccDK		12	0	8	0	2	0
PALB2	missense_variant	NM_024675.4:c.721A>G	NP_078951.2:p.Asn241Asp	Benign	ccDK		1	0	1	0	0	0
PALB2	synonymous_variant	NM_024675.4:c.735G>A	NP_078951.2:p.Ala245=	Likely Benign	BP4 (SpliceAI=0.00)<br>BP7_supporting (Silent variant with no impact on splicing)		1	0	1	0	0	0
PALB2	synonymous_variant	NM_024675.4:c.78G>A	NP_078951.2:p.Arg26=	Likely Benign	BP4 (SpliceAI=0)<br>BP7 (Silent variant and no impact on splicing)		1	0	0	0	0	0
PALB2	missense_variant	NM_024675.4:c.833T>A	NP_078951.2:p.Leu278Gln	Likely Benign	BP1 (Missense variant)<br>BP4 (SpliceAI=0.00)		1	0	0	0	0	0
PALB2	synonymous_variant	NM_024675.4:c.834A>T	NP_078951.2:p.Leu278=	Likely Benign	BP7 (Silent variant with no impact on splicing)<br>BP4 (SpliceAI=0.00)		1	0	0	0	0	0
PALB2	missense_variant	NM_024675.4:c.87C>G	NP_078951.2:p.Ser29Arg	Likely Benign	BP4 (SpliceAI=0.00)<br>BP1 (Missense variant)		3	0	2	0	1	0
PALB2	missense_variant	NM_024675.4:c.928A>G	NP_078951.2:p.Ser310Gly	Likely Benign	ccDK		2	0	1	0	1	0
PTEN	intron_variant	NM_000314.8:c.164+42T>C		Likely Benign	BP4 (SpliceAI=0.15)<br>BS1_supporting (GnomAD 0.000009217)<br>BP7 (Deep intronic variant, +7/-21, and no impact on splicing)		2	0	1	0	1	0
PTEN	intron_variant	NM_000314.8:c.165-10674G>A		Likely Benign	BP4 (SpliceAI=0.13)<br>BP7 (Deep intronic variant, +7/-21, and no impact on splicing)		1	0	1	0	0	0
PTEN	splice_region_variant	NM_000314.8:c.165-13_165-10delGTTT		Likely Benign	BP4 (SpliceAI=0.01)<br>BS1 (GnomAD 0.0002839)		1	0	1	0	0	0
PTEN	intron_variant	NM_000314.8:c.165-1545T>A		Likely Benign	BS1 (GnomAD frequency 0.000335)<br>BP4 (SpliceAI=0.15)<br>BP7 (Deep intronic variant, +7/-40, and no effect on splicing)		5	0	2	0	1	0
PTEN	intron_variant	NM_000314.8:c.165-9985C>G		Likely Benign	BS1 (GnomAD frequency 0.0004798)<br>BP4 (SpliceAI=0.12)<br>BP7 (Deep intronic variant, +7/-40, and no effect on splicing)		4	0	3	0	1	0
PTEN	intron_variant	NM_000314.8:c.209+2024G>A		Likely Benign	PM2_supporting (Absent from GnomAD)<br>BP4 (SpliceAI=0.11)<br>BP7 (Deep intronic variant, +7/-40, and no effect on splicing)		1	0	1	0	0	0
PTEN	intron_variant	NM_000314.8:c.210-2363A>G		Likely Benign	PM2_supporting (Absent from GnomAD)<br>BP4 (SpliceAI=0.13)<br>BP7 (Deep intronic variant, +7/-40, and no effect on splicing)		1	0	0	0	0	0
PTEN	splice_region_variant	NM_000314.8:c.210-7_210-3delCTTTT		Benign	BS1 (GnomAD 0.000411)<br>BS2_supporting (2 homozygote variants in GnomAD)<br>BS3 (Functional study showing no effect on splicing (PMID: 28677221))		1	0	0	0	0	0
PTEN	synonymous_variant	NM_000314.8:c.234C>T	NP_000305.3:p.Thr78=	Likely Benign	BS1 (GnomAD 0.000128838)<br>BP7 (Silent variant and no impact on splicing)		3	0	2	0	0	0
PTEN	missense_variant	NM_000314.8:c.235G>A	NP_000305.3:p.Ala79Thr	Likely Benign	BS1 (GnomAD frequency 0.000128898)<br>BS3_supporting (Phosphatase activity 0.21(PMID: 29706350))		1	0	0	0	0	0
PTEN	intron_variant	NM_000314.8:c.254-37T>G		Likely Benign	BP4 (SpliceAI=0.06)<br>BP7 (Deep intronic variant, +7/-40, and no effect on splicing)		1	0	1	0	0	0
PTEN	synonymous_variant	NM_000314.8:c.354T>C	NP_000305.3:p.His118=	Likely Benign	BP7 (Silent variant and no impact on splicing)<br>BP4 (SpliceAI=0.01)		3	0	3	0	0	0
PTEN	splice_region_variant	NM_000314.8:c.492+14T>A		Likely Benign	BP4 (SpliceAI=0.00)<br>BP7 (Deep intronic variant, +7/-40, and no effect on splicing)		2	0	1	0	1	0
PTEN	intron_variant	NM_000314.8:c.492+9363delG		Likely Benign	BS1 (GnomAD frequency 0.0003939)<br>BP4 (SpliceAI=0.14)<br>BP7 (Deep intronic variant, +7/-40, and no effect on splicing)		2	0	0	0	1	0

PTEN	intron_variant	NM_000314.8:c.492+9402A>G		Likely Benign	PM2_supporting (GnomAD only 1)<br>BP4 (SpliceAI=0.11)<br>BP7 (Deep intronic variant, +7/-40, and no effect on splicing)		1	0	1	0	0	0
PTEN	intron_variant	NM_000314.8:c.493-31A>G		Likely Benign	BP4 (SpliceAI=0.04)<br>BP7 (Deep intronic variant, +7/-40, and no effect on splicing)<br>BS1 (GnomAD 0.00004908966)		1	0	0	0	0	0
PTEN	intron_variant	NM_000314.8:c.493-34dupT		Likely Benign	BP4 (SpliceAI=0.00)<br>BP7 (Deep intronic variant, +7/-40, and no effect on splicing)<br>BS1_supporting (GnomAD 0.00002000333)		2	0	2	0	0	0
PTEN	intron_variant	NM_000314.8:c.493-9345T>C		Likely Benign	PM2_supporting (Absent from GnomAD)<br>BP4 (SpliceAI=0.13)<br>BP7 (Deep intronic variant, +7/-40, and no effect on splicing)		1	0	1	0	0	0
PTEN	intron_variant	NM_000314.8:c.635-32G>A		Likely Benign	BP4 (SpliceAI=0.01)<br>BP7 (Deep intronic variant, +7/-40, and no effect on splicing)		3	0	3	0	0	0
PTEN	intron_variant	NM_000314.8:c.635-668T>C		Likely Benign	BP4 (SpliceAI=0.11)<br>BP7 (Deep intronic variant, +7/-40, and no effect on splicing)		1	0	1	0	0	0
PTEN	synonymous_variant	NM_000314.8:c.720C>T	NP_000305.3:p.Tyr240=	Likely Benign	BS1 (GnomAD frequency 0.00016)<br>BP4 (SpliceAI=0)<br>BP7 (Silent variant and no impact on splicing)		1	0	1	0	0	0
PTEN	intron_variant	NM_000314.8:c.79+11782A>G		Likely Benign	PM2_supporting (GnomAD only 1)<br>BP4 (SpliceAI=0.12)<br>BP7 (Deep intronic variant, +7/-40, and no effect on splicing)		1	0	1	0	0	0
PTEN	intron_variant	NM_000314.8:c.79+13786C>G		Likely Benign	BS1_supporting (GnomAD frequency 0.0000394)<br>BP4 (SpliceAI=0.12)<br>BP7 (Deep intronic variant, +7/-40, and no effect on splicing)		2	0	2	0	0	0
PTEN	intron_variant	NM_000314.8:c.79+2049A>G		Likely Benign	BP4 (SpliceAI=0.15)<br>BP7 (Deep intronic variant, +7/-40, and no effect on splicing)		2	0	0	0	1	0
PTEN	intron_variant	NM_000314.8:c.79+33T>A		Likely Benign	BP4 (SpliceAI=0.01)<br>BS1_supporting (GnomAD 0.0000369)<br>BP7 (Deep intronic variant, +7/-40, and no effect on splicing)		7	0	6	0	0	0
PTEN	intron_variant	NM_000314.8:c.79+41C>G		Likely Benign	BP4 (SpliceAI=0.05)<br>BS1 (GnomAD 0.00004318)<br>BP7 (Deep intronic variant, +7/-40, and no effect on splicing)		2	0	0	0	2	0
PTEN	intron_variant	NM_000314.8:c.79+8030A>G		Likely Benign	BP4 (SpliceAI=0.11)<br>BP7 (Deep intronic variant, +7/-40, and no effect on splicing)<br>PM2_supporting (GnomAD only 1)		1	0	1	0	0	0
PTEN	intron_variant	NM_000314.8:c.80-13628T>C		Likely Benign	BS1 (GnomAD frequency 0.000447)<br>BP4 (SpliceAI=0.17)<br>BP7 (Deep intronic variant, +7/-40, and no effect on splicing)		7	0	4	0	1	0
PTEN	splice_region_variant	NM_000314.8:c.802-12T>C		Likely Benign	BS1 (GnomAD 0.0001196)<br>BP4 (SpliceAI=0.01)		1	0	0	0	1	0
RAD51C	synonymous_variant	NM_058216.3:c.1059T>A	NP_478123.1:p.Ser353=	Likely Benign	BP4 (SpliceAI=0.04)<br>BP7 (Silent variant with no impact on splicing)		1	0	1	0	0	0
RAD51C	missense_variant	NM_058216.3:c.1102C>T	NP_478123.1:p.Arg368Trp	Likely Benign	BS3 (Functional study showing HDR assay retaining neutral effekt (PMID: 37253112))<br>BP4 (Revel=0.186 and BayesDel=-0.282)		1	0	1	0	0	0
RAD51C	missense_variant	NM_058216.3:c.134A>G	NP_478123.1:p.Glu45Gly	Likely Benign	BP4 (Revel=0.076 and BayesDel=0.3998)<br>BS3 (Functional study showing neutral HDR result (PMID: 37253112))		1	0	0	0	0	0
RAD51C	splice_region_variant	NM_058216.3:c.146-8A>G		Likely Benign	ccDK		1	0	1	0	0	0
RAD51C	synonymous_variant	NM_058216.3:c.186A>G	NP_478123.1:p.Gln62=	Likely Benign	BP4 (SpliceAI=0.05)<br>BP6 (Laboratories reported it benign in ClinVar)<br>BP7 (Silent variant with no impact on splicing)		2	0	2	0	0	0
RAD51C	synonymous_variant	NM_058216.3:c.195A>G	NP_478123.1:p.Arg65=	Likely Benign	BP4 (SpliceAI=0)<br>BP6 (Laboratories reported it benign in ClinVar)<br>BP7 (Silent variant and no impact on splicing)		1	0	1	0	0	0
RAD51C	missense_variant	NM_058216.3:c.376G>A	NP_478123.1:p.Ala126Thr	Benign	ccDK		71	0	43	0	12	0
RAD51C	intron_variant	NM_058216.3:c.571+49delT		Likely Benign	BP4 (SpliceAI=0.00)<br>BP7 (Deep intronic variant, +7/-21, and no impact on splicing)		1	0	0	0	0	0
RAD51C	splice_region_variant	NM_058216.3:c.572-17G>T		Likely Benign	BP4 (SpliceAI=0.00)<br>BP6 (Laboratories reported it benign in ClinVar)		71	0	43	0	12	0
RAD51C	synonymous_variant	NM_058216.3:c.654G>A	NP_478123.1:p.Glu218=	Likely Benign	BP4 (SpliceAI=0.00)<br>BP7 (Silent variant with no impact on splicing)		4	0	2	0	1	0
RAD51C	synonymous_variant	NM_058216.3:c.783A>G	NP_478123.1:p.Leu261=	Likely Benign	BP4 (SpliceAI=0)<br>BP7 (Silent variant and no impact on splicing)		1	0	0	0	1	0
RAD51C	missense_variant	NM_058216.3:c.790G>A	NP_478123.1:p.Gly264Ser	Likely Benign	ccDK		75	0	50	0	12	0
RAD51C	missense_variant	NM_058216.3:c.7G>A	NP_478123.1:p.Gly3Arg	Likely Benign	BS3 (Functional study showed remained neutral in HDR assay (PMID: 37253112))<br>BP4 (Revel=score 0.054 and BayesDel=-0.6575)		2	0	0	0	1	0
RAD51C	missense_variant	NM_058216.3:c.859A>G	NP_478123.1:p.Thr287Ala	Likely Benign	ccDK		81	0	51	0	10	0
RAD51C	synonymous_variant	NM_058216.3:c.90G>A	NP_478123.1:p.Ala30=	Likely Benign	BP4 (SpliceAI=0)<br>BP6 (Laboratories reported it benign in ClinVar)<br>BP7 (Silent variant and no impact on splicing)		1	0	1	0	0	0
RAD51C	synonymous_variant	NM_058216.3:c.945T>C	NP_478123.1:p.Phe315=	Likely Benign	BP4 (SpliceAI=0.05)<br>BP7 (Silent variant with no impact on splicing)		1	0	1	0	0	0
RAD51D	synonymous_variant	NM_002878.4:c.117A>T	NP_002869.3:p.Val39=	Likely Benign	BP4 (SpliceAI=0)<br>BP7 (Silent variant and no impact on splicing)		2	0	2	0	1	0
RAD51D	intron_variant	NM_002878.4:c.144+30T>C		Likely Benign	BP4 (SpliceAI=0.00)<br>BP7 (Deep intronic variant, +7/-21, and no impact on splicing)		1	0	0	0	1	0
RAD51D	synonymous_variant	NM_002878.4:c.195C>T	NP_002869.3:p.Pro65=	Likely Benign	ccDK		2	0	2	0	0	0
RAD51D	synonymous_variant	NM_002878.4:c.552G>A	NP_002869.3:p.Glu184=	Likely Benign	ccDK		1	0	0	0	0	0
RAD51D	synonymous_variant	NM_002878.4:c.564T>A	NP_002869.3:p.Thr188=	Likely Benign	ccDK		3	0	1	0	2	0
RAD51D	splice_region_variant	NM_002878.4:c.576+18C>T		Likely Benign	BP4 (SpliceAI=0.00)<br>BP7 (Deep intronic variant, +7/-21, and no impact on splicing)		1	0	1	0	0	0
RAD51D	intron_variant	NM_002878.4:c.667+48C>T		Likely Benign	BP4 (SpliceAI=0.00)<br>BP7 (Deep intronic variant, +7/-21, and no impact on splicing)		1	0	1	0	0	0
RAD51D	splice_region_variant	NM_002878.4:c.667+9T>C		Likely Benign	BP4 (SpliceAI=0.01)<br>BP7 (Deep intronic variant, +7/-21, and no impact on splicing)		1	0	1	0	0	0
RAD51D	missense_variant	NM_002878.4:c.698A>G	NP_002869.3:p.Glu233Gly	Benign	ccDK		126	1	78	0	17	0
RAD51D	intron_variant	NM_002878.4:c.738+42T>C		Likely Benign	BP4 (SpliceAI=0.00)<br>BP7 (Deep intronic variant, +7/-21, and no impact on splicing)		1	0	0	0	1	0
RAD51D	synonymous_variant	NM_002878.4:c.792C>T	NP_002869.3:p.Leu264=	Likely Benign	ccDK		1	0	0	0	1	0
RAD51D	synonymous_variant	NM_002878.4:c.864C>T	NP_002869.3:p.Gly288=	Likely Benign	ccDK		3	0	2	0	1	0
RAD51D	synonymous_variant	NM_002878.4:c.883C>T	NP_002869.3:p.Leu295=	Likely Benign	BP4 (SpliceAI=0.00)<br>BP7 (Silent variant with no impact on splicing)		1	0	1	0	0	0

RAD51D	intron_variant	NM_002878.4:c.903+40delT		Likely Benign	BP4 (SpliceAI=0.00)<br>BP7 (Deep intronic variant, +7/-21, and no impact on splicing)		1	0	1	0	0	0
STK11	splice_region_variant	NM_000455.5:c.*16+8G>A		Likely Benign	BP4 (SpliceAI=0.05)<br>BP7 (Deep intronic variant, +7/-40, and no effect on splicing)		1	0	1	0	0	0
STK11	missense_variant	NM_000455.5:c.1062C>G	NP_000446.1:p.Phe354Leu	Benign	ccDK		55	2	35	1	8	1
STK11	synonymous_variant	NM_000455.5:c.1107C>T	NP_000446.1:p.Pro369=	Likely Benign	BP4 (SpliceAI=0.01)<br>BP7 (Silent variant and no impact on splicing)		1	0	1	0	0	0
STK11	synonymous_variant	NM_000455.5:c.1128G>A	NP_000446.1:p.Glu376=	Likely Benign	BP4 (SpliceAI=0)<br>BP7 (Silent variant and no impact on splicing)		1	0	1	0	0	0
STK11	synonymous_variant	NM_000455.5:c.1185A>G	NP_000446.1:p.Thr395=	Likely Benign	ccDK		4	0	3	0	0	0
STK11	synonymous_variant	NM_000455.5:c.1194G>A	NP_000446.1:p.Ala398=	Likely Benign	ccDK		2	0	1	0	0	0
STK11	synonymous_variant	NM_000455.5:c.1218G>A	NP_000446.1:p.Ala406=	Likely Benign	BP4 (SpliceAI=0.00)<br>BP7 (Silent variant with no impact on splicing)		1	0	0	0	0	0
STK11	synonymous_variant	NM_000455.5:c.1257C>T	NP_000446.1:p.Ser419=	Likely Benign	BP4 (SpliceAI=0)<br>BP7 (Silent variant and no impact on splicing)<br>BP6 (Laboratories reported it benign in ClinVar)		1	0	1	0	0	0
STK11	synonymous_variant	NM_000455.5:c.1281G>A	NP_000446.1:p.Leu427=	Likely Benign	BP4 (SpliceAI=0)<br>BP7 (Silent variant and no impact on splicing)		1	0	1	0	0	0
STK11	synonymous_variant	NM_000455.5:c.135C>G	NP_000446.1:p.Leu45=	Likely Benign	BP4 (SpliceAI=0.00)<br>BP7 (Silent variant and no impact on splicing)		1	0	0	0	1	0
STK11	synonymous_variant	NM_000455.5:c.264C>A	NP_000446.1:p.Ile88=	Likely Benign	ccDK		5	1	3	0	0	0
STK11	intron_variant	NM_000455.5:c.291-32C>T		Likely Benign	BP4 (SpliceAI=0.00)<br>BP6 (Laboratories reported it benign in ClinVar)<br>BP7 (Deep intronic variant, +7/-40, and no effect on splicing)		2	0	0	0	1	0
STK11	synonymous_variant	NM_000455.5:c.357C>T	NP_000446.1:p.Asn119=	Likely Benign	ccDK		4	0	4	0	0	0
STK11	intron_variant	NM_000455.5:c.374+23C>T		Likely Benign	BP4 (SpliceAI=0.00)<br>BP7 (Deep intronic variant, +7/-40, and no effect on splicing)		3	0	0	0	0	0
STK11	splice_region_variant	NM_000455.5:c.375-7G>A		Likely Benign	BP4 (SpliceAI=0.04)<br>BP6 (2 laboratories reported it benign in ClinVar)		1	0	0	0	0	0
STK11	synonymous_variant	NM_000455.5:c.45C>T	NP_000446.1:p.Gly15=	Likely Benign	BP4 (SpliceAI=0.00)<br>BP7 (Silent variant with no impact on splicing)		1	0	1	0	0	0
STK11	intron_variant	NM_000455.5:c.464+44dupG		Likely Benign	BP4 (SpliceAI=0.00)<br>BP7 (Deep intronic variant, +7/-40, and no effect on splicing)		12	1	7	1	3	0
STK11	splice_region_variant	NM_000455.5:c.464+8C>T		Likely Benign	BP4 (SpliceAI=0)<br>BP7 (Deep intronic variant, +7/-40, and no effect on splicing)		2	0	0	0	0	0
STK11	synonymous_variant	NM_000455.5:c.492G>A	NP_000446.1:p.Leu164=	Likely Benign	BP4 (SpliceAI=0)<br>BP7 (Silent variant and no impact on splicing)		1	0	1	0	1	0
STK11	synonymous_variant	NM_000455.5:c.528C>T	NP_000446.1:p.Asp176=	Likely Benign	BP4 (SpliceAI=0)<br>BP7 (Silent variant and no impact on splicing)		1	0	1	0	0	0
STK11	splice_region_variant	NM_000455.5:c.598-7G>A		Likely Benign	ccDK		10	0	4	0	0	0
STK11	synonymous_variant	NM_000455.5:c.609G>A	NP_000446.1:p.Pro203=	Likely Benign	BP4 (SpliceAI=0)<br>BP7 (Silent variant and no impact on splicing)		1	0	0	0	1	0
STK11	synonymous_variant	NM_000455.5:c.615G>A	NP_000446.1:p.Ala205=	Likely Benign	ccDK		6	0	5	0	1	0
STK11	synonymous_variant	NM_000455.5:c.678C>T	NP_000446.1:p.Asn226=	Likely Benign	ccDK		1	0	1	0	0	0
STK11	splice_region_variant	NM_000455.5:c.735-13C>T		Likely Benign	ccDK		1	0	1	0	0	0
STK11	synonymous_variant	NM_000455.5:c.787T>C	NP_000446.1:p.Leu263=	Likely Benign	ccDK		4	0	4	0	0	0
STK11	synonymous_variant	NM_000455.5:c.816C>T	NP_000446.1:p.Tyr272=	Likely Benign	ccDK		6	0	5	0	2	0
STK11	intron_variant	NM_000455.5:c.920+32G>A		Likely Benign	ccDK		14	0	9	0	2	0
STK11	splice_region_variant	NM_000455.5:c.920+6C>T		Likely Benign	ccDK		3	0	3	0	0	0
STK11	splice_region_variant	NM_000455.5:c.920+7G>A		Likely Benign	BP4 (SpliceAI=0)<br>BP7 (Deep intronic variant, +7/-21, and no impact on splicing)		1	0	1	0	0	0
STK11	synonymous_variant	NM_000455.5:c.945G>A	NP_000446.1:p.Pro315=	Likely Benign	ccDK		4	0	3	0	0	0
TP53	missense_variant	NM_000546.6:c.1007A>G	NP_000537.3:p.Glu336Gly	Likely Benign	BS1 (Kato et al.:Transactivation Functional and Giacomelli: notDNE_notLOF)<br>BP4: (BayesDel: -0.0545532 and aGVGD: C0)		1	0	1	0	0	0
TP53	missense_variant	NM_000546.6:c.1015G>A	NP_000537.3:p.Glu339Lys	Benign	ccDK		2	0	1	0	1	0
TP53	missense_variant	NM_000546.6:c.1079G>T	NP_000537.3:p.Gly360Val	Likely Benign	ccDK		2	0	2	0	0	0
TP53	intron_variant	NM_000546.6:c.1101-43C>T		Likely Benign	BP4 (SpliceAI=0.04)<br>BP7 (Deep intron variant, +7/-21, and no impact on splicing)		2	0	0	0	0	0
TP53	missense_variant	NM_000546.6:c.1160C>G	NP_000537.3:p.Thr387Arg	Likely Benign	BS3 (Transactivation functional and noDNE_noLOF)<br>BP4 (aGVGD C0 and BayesDel=-0.313)		1	0	1	0	0	0
TP53	synonymous_variant	NM_000546.6:c.216C>T	NP_000537.3:p.Pro72=	Likely Benign	BP4 (SpliceAI=0)<br>BP7 (Silent variant and no impact on splicing)		1	0	1	0	0	0
TP53	missense_variant	NM_000546.6:c.221C>T	NP_000537.3:p.Ala74Val	Likely Benign	ccDK		2	0	2	0	0	0
TP53	synonymous_variant	NM_000546.6:c.27C>T	NP_000537.3:p.Ser9=	Likely Benign	BP4 (SpliceAI=0)<br>BP7 (Silent with no splice effect and not highly conserved)		1	0	1	0	0	0
TP53	missense_variant	NM_000546.6:c.341T>C	NP_000537.3:p.Leu114Ser	Likely Benign	BS3 (Transactivation functional and notDNE_notLOF)		1	0	0	0	0	0
TP53	synonymous_variant	NM_000546.6:c.354A>T	NP_000537.3:p.Thr118=	Likely Benign	BP4 (SpliceAI=0)<br>BP7 (Silent variant and no impact on splicing)		1	0	1	0	0	0
TP53	missense_variant	NM_000546.6:c.521G>A	NP_000537.3:p.Arg174Lys	Likely Benign	BS3 (Kato: functional and notDNE_notLOF)<br>BP4 (BayesDel: -0.0648127 + aGVGD C0)		1	0	1	0	0	0
TP53	synonymous_variant	NM_000546.6:c.612G>A	NP_000537.3:p.Glu204=	Likely Benign	ccDK		1	0	1	0	0	0
TP53	missense_variant	NM_000546.6:c.642T>G	NP_000537.3:p.His214Gln	Likely Benign	ccDK		1	0	1	0	0	0
TP53	intron_variant	NM_000546.6:c.672+48G>A		Likely Benign	BS1 (GnomAD frequency 0.000682)<br>BP4 (SpliceAI=0.02)<br>BP7 (Deep intron variant, +7/-21, and no impact on splicing)		3	0	3	0	0	0
TP53	missense_variant	NM_000546.6:c.704A>G	NP_000537.3:p.Asn235Ser	Benign	ccDK		7	0	6	0	0	0
TP53	splice_region_variant	NM_000546.6:c.782+12C>T		Likely Benign	ccDK		3	0	2	0	1	0
TP53	synonymous_variant	NM_000546.6:c.855G>A	NP_000537.3:p.Glu285=	Likely Benign	BP7 (SpliceAI=0.00)<br>BP7 (Silent variant and no impact on splicing)		1	0	1	0	0	0
TP53	missense_variant	NM_000546.6:c.869G>A	NP_000537.3:p.Arg290His	Likely Benign	BS3 (Transactivation: supertransactivation and notDNE+notLOF)<br>BP4_supporting (aGVGD C0 and BayesDel=0.0916)		6	0	3	0	1	0
TP53	missense_variant	NM_000546.6:c.91G>A	NP_000537.3:p.Val31Ile	Likely Benign	BS3 (Kato: Functional and Giacomelli: notDNE_notLOF)<br>BP4 (BayesDel=score: -0.0168 and aGVGD class: C0)		1	0	0	0	0	0
TP53	missense_variant	NM_000546.6:c.929A>G	NP_000537.3:p.Asn310Ser	Likely Benign	BS3 (Kato et al.: supertransactivation and Giacomelli: notDNE_notLOF)<br>BP4 (BayesDel: -0.326582 and aGVGD: C0)		1	0	0	0	0	0

Table S7: All the identified 5'-UTR variants found in our study populaiton of 5,923 patients with a clinical suspicion of predisposition to heredirary breast- and/or ovarian cancer

AF: Allele Frequency
AG: Acceptor gain
AL: Acceptor loss
BC: Breast cancer
ccDK: Cancer (variant) classification DK (The variants classified by this group were not assigned ACMG criteria)
DL: Donor loss
DG: Donor gain
ENIGMA: Evidence-based Network for the Interpretation of Germline Mutant Alleles (The variants classified by this group were not assigned ACMG criteria)
GnomAD: Genome Aggregation Database
Hom: Homozygot
OC: Ovarian cancer

Gene	Sequence Ontology	HGVS c.	Assigned ACMG criteria	Classification according to ACMG criteria	Suggested, not validated, criteria	Classification according to suggested, not validated, criteria	PreTIS assessment	Comments	#Samples_Total (N=5.923)	#Hom samples_Total (N=5.923)	#Samples_BC (N=3.706)	#Hom samples_BC (N=3.706)	#Samples_OC (N=890)	#Hom samples_OC (N=890)
ATM	5'-UTR	NM_000051.4:c.-112C>G	PM2_supporting (Absent in GnomAD)	Unknown Significance	PM2_supporting (Absent in GnomAD)<br>BP7 (Beyond +7/-21 and SpliceAI=0)<br>BP4 (CADD=8.679)	Likely Benign	Increase in PreTIS score was less than 0		2	0	1	0	1	0
ATM	5'-UTR	NM_000051.4:c.-129A>C	No criteria met	Unknown Significance	BP7 (Beyond +7/-21 and SpliceAI=0)<br>BP4 (CADD=8.558)	Likely Benign	Increase in PreTIS score was less than 0		9	0	7	0	1	0
ATM	5'-UTR	NM_000051.4:c.-12A>C	PM2_supporting (Absent in GnomAD)	Unknown Significance	PM2_supporting (Absent in GnomAD)<br>BP4 (CADD=14.37)	Unknown Significance	Increase in PreTIS score was less than 0		1	0	0	0	0	0
ATM	5'-UTR	NM_000051.4:c.-138G>A	PM2_supporting (Absent in GnomAD)	Unknown Significance	PM2_supporting (Absent in GnomAD)<br>BP7 (Beyond +7/-21 and SpliceAI=0)<br>BP4 (CADD=12)	Likely Benign	PreTIS score for the mutated allele was decreasing compared to the wildtype allele		3	0	0	0	1	0
ATM	5'-UTR	NM_000051.4:c.-18G>A	No criteria met	Unknown Significance	BP4 (CADD=3.67)<br>PP3 (Positive PreTIS assessment)	Unknown Significance	PreTIS score 0.74 for alternative start codon GUG in extension -7 nts. Moderate confidence. PreTIS score increased from 0.69 in wildtype allele to 0.74 in mutated allele<br>PreTIS score 0.80 for alternative start codon CUG in extension -15 nts. High confidence. PreTIS score increased from 0.71 in wildtype allele to 0.80 in mutated allele<br>PreTIS score 0.72 for alternative start codon GUG in extension -22 nts. Moderate confidence. PreTIS score increased from 0.65 in wildtype allele to 0.72 in mutated allele<br>PreTIS score 0.81 for alternative start codon GUG in extension -27 nts. High confidence. PreTIS score increased from 0.76 in wildtype allele to 0.81 in mutated allele		1	0	0	0	0	0
ATM	5'-UTR	NM_000051.4:c.-42C>T	PM2_supporting (Absent from GnomAD)	Unknown Significance	PM2_supporting (Absent from GnomAD)<br>BP7 (Beyond +7/-21 and SpliceAI=0)<br>BP4 (CADD=12.07)	Likely Benign	PreTIS score for the mutated allele was the same compared to the wildtype allele		1	0	1	0	0	0
ATM	5'-UTR	NM_000051.4:c.-52C>A	PM2_supporting (Only 1 in GnomAD)	Unknown Significance	PM2_supporting (Only 1 in GnomAD)<br>BP4 (CADD=12.01)	Unknown Significance	Increase in PreTIS score was less than 0.05 for mutated allele compared to wildtype allele		1	0	1	0	0	0
ATM	5'-UTR	NM_000051.4:c.-69C>A	PM2_supporting (Only 1 in GnomAD)	Unknown Significance	PM2_supporting (Only 1 in GnomAD)<br>BP7 (Beyond +7/-21 and SpliceAI=0)<br>BP4 (CADD=10.43)	Likely Benign	Increase in PreTIS score was less than 0.05 for mutated allele compared to wildtype allele		1	0	1	0	0	0
BARD1	5'-UTR	NM_004465.4:c.-114A>G	No criteria met	Unknown Significance	BP7 (Beyond +7/-21 and SpliceAI=0)<br>BP4 (CADD=9.662)	Likely Benign	Increase in PreTIS score was less than 0.05 for mutated allele compared to wildtype allele		1	0	1	0	0	0
BARD1	5'-UTR	NM_004465.4:c.-36C>T	No criteria met	Unknown Significance	BP7 (Beyond +7/-21 and SpliceAI=0)<br>BP4 (CADD=8.706)	Likely Benign	PreTIS score for the mutated allele was decreasing compared to the wildtype allele		1	0	0	0	0	0
BARD1	5'-UTR	NM_004465.4:c.-83C>T	BP6 (Laboratories reported it benign in ClinVar)	Unknown Significance	BP6 (Laboratories reported it benign in ClinVar)<br>BP7 (Beyond +7/-21 and SpliceAI=0)<br>BP4 (CADD=8.485)	Likely Benign	PreTIS score for the mutated allele was decreasing compared to the wildtype allele		299	4	174	1	54	2
BARD1	5'-UTR	NM_004465.4:c.-88C>G	PM2_supporting (Absent from GnomAD)	Unknown Significance	PM2_supporting (Absent from GnomAD)<br>BP7 (Beyond +7/-21 and SpliceAI=0)<br>BP4 (CADD=9.289)	Likely Benign	PreTIS score for the mutated allele was decreasing compared to the wildtype allele		2	0	1	0	1	0
BRCA1	5'-UTR	NM_007294.4:c.-10A>C	ccDK	Unknown Significance	ccDK	Unknown Significance	PreTIS score <0.7. Low confidence		1	0	1	0	0	0
BRCA1	5'-UTR	NM_007294.4:c.-29C>G	No criteria met	Unknown Significance	BP4 (CADD=8.347)	Unknown Significance	PreTIS score <0.7. Low confidence	SpliceAI DL: 0.29. Possibly partiel exon skipping. RAW score alternative donor changes from 0.59 to 0.29 and natural donor changes from 0.79 to 0.80	1	0	1	0	0	0
BRCA1	5'-UTR	NM_007294.4:c.-66C>G	No criteria met	Unknown Significance	BP4 (CADD=9.612)	Unknown Significance	PreTIS score <0.7. Low confidence		2	0	2	0	0	0
BRCA1	5'-UTR	NM_007294.4:c.-86C>T	BS1_supporting (GnomAD frequency 0.0000818)	Unknown Significance	BS1_supporting (GnomAD frequency 0.0000818)<br>BP7 (Beyond +7/-21 and SpliceAI=0)<br>BP4 (CADD=8.822)	Likely Benign	PreTIS score <0.7. Low confidence		7	0	4	0	1	0
BRCA2	5'-UTR	NM_000059.4:c.-11C>T	ENIGMA	Benign	ENIGMA	Benign	PreTIS score 0.82 for alternative start codon UUG in extension -12 nts. High confidence. Not exiting in the wild type allele		1	0	1	0	0	0
BRCA2	5'-UTR	NM_000059.4:c.-162G>A	ENIGMA	Benign	ENIGMA	Benign	PreTIS score for the mutated allele was decreasing compared to the wildtype allele		1	0	1	0	0	0
BRCA2	5'-UTR	NM_000059.4:c.-166_-156delTGCTGCGCCTC	No criteria met	Unknown Significance	BP7 (Beyond +7/-21 and SpliceAI=0)<br>BP4 (CADD=7.629)	Likely Benign	PreTIS score for the mutated allele was decreasing compared to the wildtype allele		1	0	1	0	0	0
BRCA2	5'-UTR	NM_000059.4:c.-171G>C	No criteria met	Unknown Significance	BP7 (Beyond +7/-21 and SpliceAI=0)<br>BP4 (CADD=4.479)	Likely Benign	PreTIS score for the mutated allele was the same compared to the wildtype allele		2	0	2	0	0	0
BRCA2	5'-UTR	NM_000059.4:c.-197A>C	BS1_strong (GnomAD frequency 0.0001839)	Likely Benign	BS1_strong (GnomAD frequency 0.0001839)<br>BP7 (Beyond +7/-21 and SpliceAI=0)<br>BP4 (CADD=9.74)	Likely Benign	PreTIS score for the mutated allele was the same compared to the wildtype allele		1	0	1	0	0	0
BRCA2	5'-UTR	NM_000059.4:c.-34T>C	No criteria met	Unknown Significance	BP7 (Beyond +7/-21 and SpliceAI=0)<br>BP4 (CADD=2.36)	Likely Benign	PreTIS score for the mutated allele was the same or decreasing compared to the wildtype allele		1	0	1	0	0	0
BRCA2	5'-UTR	NM_000059.4:c.-57A>G	PM2_supporting (Absent from GnomAD)	Unknown Significance	PM2_supporting (Absent from GnomAD)<br>BP7 (Beyond +7/-21 and SpliceAI=0)<br>BP4 (CADD=7.778)<br>PP3 (Positive PreTIS assessment)	Unknown Significance	PreTIS score 0.90 for alternative start codon AUU in extension -24 nts. Very high confidence. PreTIS score increased from 0.85 in wildtype allele to 0.90 in mutated allele		1	0	0	0	0	0
BRCA2	5'-UTR	NM_000059.4:c.-72T>C	PM2_supporting (Absent from GnomAD)	Unknown Significance	PM2_supporting (Absent from GnomAD)<br>BP7 (Beyond +7/-21 and SpliceAI=0)<br>BP4 (CADD=9.828)	Likely Benign	PreTIS score for the mutated allele was decreasing compared to the wildtype allele		1	0	1	0	0	0
BRCA2	5'-UTR	NM_000059.4:c.-82G>C	No criteria met	Unknown Significance	BP7 (Beyond +7/-21 and SpliceAI=0)<br>BP4 (CADD=8.988)	Likely Benign	Increase in PreTIS scores was less than 0.05 for mutated allele compared to wildtype allele		2	0	2	0	0	0
BRIP1	5'-UTR	NM_032043.3:c.-114T>G	PM2_supporting (Absent from GnomAD)	Unknown Significance	BP7 (Beyond +7/-21 and SpliceAI=0)<br>BP4 (CADD=12.19)<br>PM2_supporting (Absent from GnomAD)	Likely Benign	PreTIS score for the mutated allele was the same compared to the wildtype allele		2	0	2	0	0	0
BRIP1	5'-UTR	NM_032043.3:c.-127C>T	No criteria met	Unknown Significance	BP7 (Beyond +7/-21 and SpliceAI=0)<br>BP4 (CADD=9.943)	Likely Benign	PreTIS score for the mutated allele was the same compared to the wildtype allele		1	0	0	0	0	0

BRIP1	5'-UTR	NM_032043.3:c.-197C>A	BP6 (Laboratories reported it benign in ClinVar)	Unknown Significance	BP6 (Laboratories reported it benign in ClinVar)<br>BP7 (Beyond +7/-21 and SpliceAI=0)<br>BP4 (CADD=7.034)	Likely Benign	PreTIS score for the mutated allele was the same compared to the wildtype allele		2	0	2	0	0	0
BRIP1	5'-UTR	NM_032043.3:c.-229C>G	PM2_supporting (Only 5 in GnomAD)	Unknown Significance	BP7 (Beyond +7/-21 and SpliceAI=0)<br>BP4 (CADD=0.101)<br>PM2_supporting (Only 5 in GnomAD)	Likely Benign	PreTIS score for the mutated allele was the same compared to the wildtype allele		1	0	1	0	0	0
BRIP1	5'-UTR	NM_032043.3:c.-64G>C	PM2_supporting (Absent from GnomAD)	Unknown Significance	BP7 (Beyond +7/-21 and SpliceAI=0)<br>BP4 (CADD=9.827)<br>PM2_supporting (Absent from GnomAD)<br>PP3 (Positive PreTIS assessment)	Unknown Significance	PreTIS score 0.71 for alternative start codon GUG in extension -74 nts. Moderate confidence.<br>PreTIS score increased from 0.66 in wildtype allele to 0.71 in mutated allele		2	0	2	0	0	0
CDH1	5'-UTR	NM_004360.5:c.-61C>T	PM2_supporting (Absent from GnomAD)	Unknown Significance	PM2_supporting (Absent from GnomAD)<br>BP7 (Beyond +7/-21 and SpliceAI=0)<br>BP4 (CADD=13.68)	Likely Benign	No alternative start codon		1	0	1	0	0	0
CDH1	5'-UTR	NM_004360.5:c.-63_-54dupCTCCAGCCCG	No criteria met	Unknown Significance	BP7 (Beyond +7/-21 and SpliceAI=0)<br>BP4 (CADD=16.28)	Likely Benign	PreTIS score <0.7. Low confidence		1	0	0	0	0	0
CDH1	5'-UTR	NM_004360.5:c.-65C>G	No criteria met	Unknown Significance	BP7 (Beyond +7/-21 and SpliceAI=0)<br>BP4 (CADD=12.4)	Likely Benign	No alternative start codon		1	0	0	0	1	0
CDH1	5'-UTR	NM_004360.5:c.-65C>T	No criteria met	Unknown Significance	BP7 (Beyond +7/-21 and SpliceAI=0)<br>BP4 (CADD=12.8)	Likely Benign	No alternative start codon		1	0	1	0	0	0
CDH1	5'-UTR	NM_004360.5:c.-8G>A	No criteria met	Unknown Significance	BP4 (CADD=8.759)	Unknown Significance	No alternative start codon		1	0	0	0	0	0
CHEK2	5'-UTR	NM_007194.4:c.-10dupC	No criteria met	Unknown Significance	BP4 (CADD=18.18)	Unknown Significance	No alternative start codon		8	0	7	0	0	0
CHEK2	5'-UTR	NM_007194.4:c.-41T>C	PM2_supporting (Only 2 in GnomAD)	Unknown Significance	PM2_supporting (Only 2 in GnomAD)<br>BP7 (Beyond +7/-21 and SpliceAI=0)<br>BP4 (CADD=14.44)	Likely Benign	No alternative start codon		1	0	1	0	0	0
PALB2	5'-UTR	NM_024675.4:c.-128A>C	No criteria met	Unknown Significance	BP7 (Beyond +7/-21 and SpliceAI=0)<br>BP4 (CADD=7.58)	Likely Benign	Not possible to perform due to lack of mouse sequencing data		3	0	3	0	0	0
PALB2	5'-UTR	NM_024675.4:c.-24T>C	No criteria met	Unknown Significance	BP7 (Beyond +7/-21 and SpliceAI=0)<br>BP4 (CADD=10.92)	Likely Benign	Not possible to perform due to lack of mouse sequencing data		1	0	0	0	1	0
PTEN	5'-UTR	NM_000314.8:c.-111G>T	BS1 (GnomAD frequency 0.000135)	Likely Benign	BS1 (GnomAD frequency 0.000135)<br>BP7 (Beyond +7/-21 and SpliceAI=0)	Likely Benign	Increase in PreTIS score was less than 0.05 for mutated allele compared to wildtype allele		2	0	2	0	0	0
PTEN	5'-UTR	NM_000314.8:c.-152A>G	BP6 (A laboratory reported it benign in ClinVar)	Unknown Significance	BP6 (A laboratory reported it benign in ClinVar)<br>BP7 (Beyond +7/-21 and SpliceAI=0)	Likely Benign	Increase in PreTIS score was less than 0.05 for mutated allele compared to wildtype allele		1	0	1	0	0	0
PTEN	5'-UTR	NM_000314.8:c.-246C>T	BS1_ supporting (GnomAD frequency 0.0000202)	Unknown Significance	BS1_ supporting (GnomAD frequency 0.0000202)<br>BP7 (Beyond +7/-21 and SpliceAI=0)	Likely Benign	Increase in PreTIS score was less than 0.05 for mutated allele compared to wildtype allele		2	0	1	0	0	0
PTEN	5'-UTR	NM_000314.8:c.-295T>C	BS1 (GnomAD frequency 0.00001952)	Likely Benign	BS1 (GnomAD frequency 0.00001952)<br>BP7 (Beyond +7/-21 and SpliceAI=0)	Likely Benign	Increase in PreTIS score was less than 0.05 for mutated allele compared to wildtype allele		1	0	1	0	0	0
PTEN	5'-UTR	NM_000314.8:c.-307C>A	BS1_supporting (GnomAD frequency 0.0000283)	Unknown Significance	BS1_supporting (GnomAD frequency 0.0000283)<br>BP7 (Beyond +7/-21 and SpliceAI=0)<br>BP4 (CADD=19.19)	Likely Benign	Increase in PreTIS score was less than 005 for mutated allel compared to wildtype allel.		2	0	2	0	0	0
PTEN	5'-UTR	NM_000314.8:c.-367_-366insGG	BS1 (GnomAD frequency 0.000353569)	Likely Benign	BS1 (GnomAD frequency 0.000353569)<br>BP7 (Beyond +7/-21 and SpliceAI=0)<br>PP3 (Positive PreTIS assessment)	Unknown Significance	PreTIS score 0.73 for alternative start codon GUG in extension -367 nts. Moderate confidence.<br>PreTIS score increased from 0.38 in wildtype allele to 0.73 in mutated allele.<br>PreTIS score 0.77 for alternative start codon ACG in extension -385 nts. Moderate confidence.<br>Not exiting in the wild type allele.<br>PreTIS score 0.95 for alternative start codon CUG in extension -396 nts. Very high confidence.<br>Not exiting in the wild type allele.<br>PreTIS score 0.90 for alternative start codon UUG in extension -414 nts. Very high confidence.<br>Not exiting in the wild type allele.<br>PreTIS score 0.72 for alternative start codon CUG in extension -431 nts. Moderate confidence.<br>Not exiting in the wild type allele.<br>PreTIS score 0.79 for alternative start codon AUU in extension -441 nts. Moderate confidence.<br>Not exiting in the wild type allele.<br>PreTIS score 0.77 for alternative start codon GUG in extension -576 nts. Moderate confidence.<br>PreTIS score increased from 0.72 in wildtype allele to 0.77 in mutated allele		1	0	0	0	0	0
PTEN	5'-UTR	NM_000314.8:c.-401_-400delCC	PM2_supporting (Absent from GnomAD)	Unknown Significance	BP7 (Beyond +7/-21 and SpliceAI=0)<br>PM2_supporting (Absent from GnomAD)<br>PP3 (Positive PreTIS assessment)	Unknown Significance	PreTIS score 0.74 for alternative start codon GUG in extension -583 nts. Moderate confidence.<br>Not exiting in the wild type allele PreTIS score 0.74 for alternative start codon GUG in extension -574 nts. Moderate confidence. Not exiting in the wild type allele PreTIS score 0.75 for alternative start codon AUU in extension -437 nts. Moderate confidence. Not exiting in the wild type allele PreTIS score 0.93 for alternative start codon UUG in extension -410 nts. Very high confidence. Not exiting in the wild type allele PreTIS score 0.84 for alternative start codon ACG in extension -383 nts. High confidence. PreTIS score increased from 0.76 in wildtype allele to 0.84 in mutated allele.		1	0	1	0	0	0
PTEN	5'-UTR	NM_000314.8:c.-435C>G	PM2_supporting (Absent from GnomAD)	Unknown Significance	PM2_supporting (Absent from GnomAD)<br>BP7 (Beyond +7/-21 and SpliceAI=0)	Unknown Significance	PreTIS score for the mutated allele was decreasing compared to the wildtype allele		1	0	1	0	0	0
PTEN	5'-UTR	NM_000314.8:c.-443C>T	PM2_supporting (Only 1 in GnomAD)	Unknown Significance	PM2_supporting (Only 1 in GnomAD)<br>BP7 (Beyond +7/-21 and SpliceAI=0)	Unknown Significance	PreTIS score for the mutated allele was decreasing compared to the wildtype allele		1	0	0	0	0	0
PTEN	5'-UTR	NM_000314.8:c.-466G>A	PM2_supporting (Only 2 in GnomAD)	Unknown Significance	PM2_supporting (Only 2 in GnomAD)<br>BP7 (Beyond +7/-21 and SpliceAI=0)<br>BP4 (CADD=15.43)	Likely Benign	PreTIS score for the mutated allele was decreasing compared to the wildtype allele		2	0	1	0	0	0
PTEN	5'-UTR	NM_000314.8:c.-488G>A	PM2_supporting (Only 1 in GnomAD)	Unknown Significance	PM2_supporting (Only 1 in GnomAD)<br>BP7 (Beyond +7/-21 and SpliceAI=0)<br>BP4 (CADD=14.83)	Likely Benign	Increase in PreTIS score was less than 0.05 for mutated allele compared to wildtype allele		1	0	0	0	0	0
PTEN	5'-UTR	NM_000314.8:c.-513G>A	No criteria met	Unknown Significance	BP7 (Beyond +7/-21 and SpliceAI=0)	Unknown Significance	PreTIS score for the mutated allele was the same compared to the wildtype allele		1	0	0	0	1	0
PTEN	5'-UTR	NM_000314.8:c.-513G>C	BS1 (GnomAD frequency 0.00009407)	Likely Benign	BS1 (GnomAD frequency 0.00009407)<br>BP7 (Beyond +7/-21 and SpliceAI=0)	Likely Benign	Increase in PreTIS score was less than 0.05 for mutated allele compared to wildtype allele		4	0	2	0	0	0
PTEN	5'-UTR	NM_000314.8:c.-525C>T	PM2_supporting (Absent from GnomAD)	Unknown Significance	BP7 (Beyond +7/-21 and SpliceAI=0)<br>PM2_supporting (Absent from GnomAD)	Unknown Significance	PreTIS score for the mutated allele was the same compared to the wildtype allele		1	0	0	0	1	0
PTEN	5'-UTR	NM_000314.8:c.-532C>T	BS1 (GnomAD frequency 0.00030)	Likely Benign	BS1 (GnomAD frequency 0.00030)<br>BP7 (Beyond +7/-21 and SpliceAI=0)<br>BP4 (CADD=18.1)	Likely Benign	Increase in PreTIS score was less than 0.05 for mutated allele compared to wildtype allele		1	0	0	0	0	0
PTEN	5'-UTR	NM_000314.8:c.-533G>T	BS1 (GnomAD frequency 0.0002977)	Likely Benign	BS1 (GnomAD frequency 0.0002977)<br>BP7 (Beyond +7/-21 and SpliceAI=0)<br>BP4 (CADD=19.42)	Likely Benign	Increase in PreTIS score was less than 0.05 for mutated allele compared to wildtype allele		1	0	0	0	0	0
PTEN	5'-UTR	NM_000314.8:c.-569C>T	PM2_supporting (Only 2 in GnomAD)	Unknown Significance	BP7 (Beyond +7/-21 and SpliceAI=0)<br>BP4 (CADD=12.53)	Likely Benign	PreTIS score for the mutated allele was the same compared to the wildtype allele		1	0	0	0	1	0
PTEN	5'-UTR	NM_000314.8:c.-594A>T	BS1 (GnomAD frequency 0.00008918)	Likely Benign	BS1 (GnomAD frequency 0.00008918)<br>BP4 (CADD=9.589)	Likely Benign	Increase in PreTIS score was less than 0.05 for mutated allele compared to wildtype allele	Splice DG: 0.50. Possibly cryptic splite site in 5'-UTR region. RAW score alternative donor changes from 0 to 0.50 and natural donor changes from 0.63 to 0.60	6	0	3	0	2	0
PTEN	5'-UTR	NM_000314.8:c.-606C>T	PM2_supporting (Only 1 in GnomAD)	Unknown Significance	BP7 (Beyond +7/-21 and SpliceAI=0)<br>BP4 (CADD=12.81)<br>PP3 (Positive PreTIS assessment)<br>PM2_supporting (Only 1 in GnomAD)	Unknown Significance	PreTIS score 0.85 for alternative start codon UUG in extension -607 nts. High confidence. Not exiting in the wild type allele		1	0	1	0	0	0

PTEN	5'-UTR	NM_000314.8:c.-622C>T	BS1 (GnomAD frequency 0.00026)	Likely Benign	BS1 (GnomAD frequency 0.00026)<br>BP7 (Beyond +7/-21 and SpliceAI=0)<br>BP4 (CADD=14.42)	Likely Benign	PreTIS score for the mutated allele was the same compared to the wildtype allele		3	0	2	0	0	0
PTEN	5'-UTR	NM_000314.8:c.-656C>T	No criteria met	Unknown Significance	BP7 (Beyond +7/-21 and SpliceAI=0)<br>PP3 (Positive PreTIS assessment)	Unknown Significance	PreTIS score 0.80 for alternative start codon GUG in extension -657 nts. High confidence. Not exiting in the wild type allele		1	0	1	0	0	0
PTEN	5'-UTR	NM_000314.8:c.-66A>C	BS1 (GnomAD frequency 0.00004614)	Likely Benign	BS1 (GnomAD frequency 0.00004614)<br>BP7 (Beyond +7/-21 and SpliceAI=0)	Likely Benign	Increase in PreTIS score was less than 0.05 for mutated allele compared to wildtype allele		3	0	2	0	0	0
PTEN	5'-UTR	NM_000314.8:c.-686G>A	BS1_supporting (GnomAD frequency 0.00001796)	Unknown Significance	BS1_supporting (GnomAD frequency 0.00001796)<br>BP7 (Beyond +7/-21 and SpliceAI=0)	Likely Benign	PreTIS score for the mutated allele was the same compared to the wildtype allele		1	0	0	0	0	0
PTEN	5'-UTR	NM_000314.8:c.-68A>G	PM2_supporting (Absent from GnomAD)	Unknown Significance	BP7 (Beyond +7/-21 and SpliceAI=0)<br>PM2_supporting (Absent from GnomAD)	Unknown Significance	PreTIS score for the mutated allele was decreasing compared to the wildtype allele		1	0	0	0	0	0
PTEN	5'-UTR	NM_000314.8:c.-693C>G	PM2_supporting (Absent from GnomAD)	Unknown Significance	BP7 (Beyond +7/-21 and SpliceAI=0)<br>PM2_supporting (Absent from GnomAD)	Unknown Significance	PreTIS score for the mutated allele was the same compared to the wildtype allele		1	0	1	0	0	0
PTEN	5'-UTR	NM_000314.8:c.-694C>G	PM2_supporting (Absent from GnomAD)	Unknown Significance	BP7 (Beyond +7/-21 and SpliceAI=0)<br>PM2_supporting (Absent from GnomAD)	Unknown Significance	PreTIS score for the mutated allele was the same compared to the wildtype allele		1	0	0	0	0	0
PTEN	5'-UTR	NM_000314.8:c.-701G>A	BS1 (GnomAD frequency 0.000159)	Likely Benign	BS1 (GnomAD frequency 0.000159)<br>BP7 (Beyond +7/-21 and SpliceAI=0)	Likely Benign	PreTIS score for the mutated allele was the same compared to the wildtype allele		1	0	0	0	0	0
PTEN	5'-UTR	NM_000314.8:c.-724C>T	PM2_supporting (Only 1 in GnomAD)	Unknown Significance	PM2_supporting (Only 1 in GnomAD)<br>BP7 (Beyond +7/-21 and SpliceAI=0)	Unknown Significance	PreTIS score for the mutated allele was the same compared to the wildtype allele		2	0	2	0	0	0
PTEN	5'-UTR	NM_000314.8:c.-72C>T	No criteria met	Unknown Significance	BP7 (Beyond +7/-21 and SpliceAI=0)	Unknown Significance	PreTIS score for the mutated allele was decreasing compared to the wildtype allele		1	0	1	0	0	0
PTEN	5'-UTR	NM_000314.8:c.-734G>A	BS1 (GnomAD frequency 0.000162)	Likely Benign	BS1 (GnomAD frequency 0.000162)<br>BP7 (Beyond +7/-21 and SpliceAI=0)	Likely Benign	PreTIS score for the mutated allele was the same compared to the wildtype allele		3	0	2	0	0	0
PTEN	5'-UTR	NM_000314.8:c.-765G>A	BS1 (GnomAD frequency 0.00004323)	Likely Benign	BS1 (GnomAD frequency 0.00004323)<br>BP7 (Beyond +7/-21 and SpliceAI=0)	Likely Benign	PreTIS score for the mutated allele was the same compared to the wildtype allele		1	0	1	0	0	0
PTEN	5'-UTR	NM_000314.8:c.-76C>T	BS1_supporting (GnomAD frequency 0.00000914)	Unknown Significance	BP7 (Beyond +7/-21 and SpliceAI=0)<br>BS1_supporting (GnomAD frequency 0.00000914)	Likely Benign	Increase in PreTIS score was less than 0.05 for mutated allele compared to wildtype allele		1	0	1	0	0	0
PTEN	5'-UTR	NM_000314.8:c.-797G>A	PM2_supporting (Only 1 in GnomAD)	Unknown Significance	PM2_supporting (Only 1 in GnomAD)<br>BP7 (Beyond +7/-21 and SpliceAI=0)	Unknown Significance	PreTIS score for the mutated allele was the same compared to the wildtype allele		1	0	0	0	0	0
PTEN	5'-UTR	NM_000314.8:c.-824C>A	PM2_supporting (Only 1 in GnomAD)	Unknown Significance	PM2_supporting (Only 1 in GnomAD)<br>BP7 (Beyond +7/-21 and SpliceAI=0)	Unknown Significance	PreTIS score for the mutated allele was the same compared to the wildtype allele		1	0	0	0	1	0
PTEN	5'-UTR	NM_000314.8:c.-835C>T	BS1 (GnomAD frequency 0.000163)	Likely Benign	BS1 (GnomAD frequency 0.000163)<br>BP7 (Beyond +7/-21 and SpliceAI=0)	Likely Benign	PreTIS score for the mutated allele was the same compared to the wildtype allele		8	0	4	0	0	0
RAD51D	5'-UTR	NM_002878.4:c.-139C>T	No criteria met	Unknown Significance	BP7 (Beyond +7/-21 and SpliceAI=0)<br>BP4 (CADD=8.919)	Likely Benign	PreTIS score for the mutated allele was the same compared to the wildtype allele		1	0	1	0	0	0
RAD51D	5'-UTR	NM_002878.4:c.-166C>T	No criteria met	Unknown Significance	BP7 (Beyond +7/-21 and SpliceAI=0)<br>BP4 (CADD=10.21)	Likely Benign	PreTIS score for the mutated allele was the same compared to the wildtype allele		2	0	2	0	0	0
RAD51D	5'-UTR	NM_002878.4:c.-183G>A	No criteria met	Unknown Significance	BP7 (Beyond +7/-21 and SpliceAI=0)<br>BP4 (CADD=10.72)	Likely Benign	PreTIS score for the mutated allele was decreasing compared to the wildtype allele		1	0	1	0	0	0
RAD51D	5'-UTR	NM_002878.4:c.-239C>T	No criteria met	Unknown Significance	BP4 (CADD=13.12)	Unknown Significance	PreTIS score for the mutated allele was the same compared to the wildtype allele		7	0	5	0	0	0
RAD51D	5'-UTR	NM_002878.4:c.-77G>A	No criteria met	Unknown Significance	BP4 (CADD=10.88)<br>BP7 (Beyond +7/-21 and SpliceAI=0)	Likely Benign	PreTIS score for the mutated allele was decreasing compared to the wildtype allele		1	0	1	0	0	0
STK11	5'-UTR	NM_000455.5:c.-106_-101delCTTTTT	PM2_supporting (Absent from GnomAD)	Unknown Significance	BP7 (Beyond +7/-21 and SpliceAI=0)<br>BP4 (CADD=10.18)<br>PM2_supporting (Absent from GnomAD)<br>PP3 (Positive PreTIS assessment)	Unknown Significance	PreTIS score 0.71 for alternative start codon AUG in extension -365 nts. Moderate confidence. Not exiting in the wild type allele<br>PreTIS score 0.72 for alternative start codon CUG in extension -304 nts. Moderate confidence. Not exiting in the wild type allele<br>PreTIS score 0.70 for alternative start codon AUC in extension -163 nts. Moderate confidence. Not exiting in the wild type allele		1	0	1	0	0	0
STK11	5'-UTR	NM_000455.5:c.-106C>G	No criteria met	Unknown Significance	BP7 (Beyond +7/-21 and SpliceAI=0)<br>BP4 (CADD=6.403)	Likely Benign	PreTIS score for the mutated allele was decreasing compared to the wildtype allele		2	0	1	0	1	0
STK11	5'-UTR	NM_000455.5:c.-107dupT	BP6 (A laboratory reported it benign in ClinVar)	Unknown Significance	BP6 (A laboratory reported it benign in ClinVar)<br>BP7 (Beyond +7/-21 and SpliceAI=0)<br>BP4 (CADD=12.66)<br>PP3 (Positive PreTIS assessment)	Unknown Significance	PreTIS score 0.72 for alternative start codon AUG in extension -372 nts. Moderate confidence. Not exiting in the wild type allele<br>PreTIS score 0.73 for alternative start codon CUG in extension -311 nts. Moderate confidence. Not exiting in the wild type allele<br>PreTIS score 0.70 for alternative start codon AUC in extension -170 nts. Moderate confidence. Not exiting in the wild type allele		5	0	3	0	0	0
STK11	5'-UTR	NM_000455.5:c.-1103C>T	No criteria met	Unknown Significance	BP7 (Beyond +7/-21 and SpliceAI=0)<br>BP4 (CADD=16.17)	Likely Benign	PreTIS score for the mutated allele was the same compared to the wildtype allele		4	1	4	1	1	0
STK11	5'-UTR	NM_000455.5:c.-1112_-1104delTGTCGGGCG	PM2_supporting (Absent from GnomAD)	Unknown Significance	PM2_supporting (Absent from GnomAD)<br>BP7 (Beyond +7/-21 and SpliceAI=0)	Unknown Significance	PreTIS score for the mutated allele was the same compared to the wildtype allele		1	0	1	0	0	0
STK11	5'-UTR	NM_000455.5:c.-11C>T	No criteria met	Unknown Significance	BP4 (CADD=10.53)	Unknown Significance	PreTIS score for the mutated allele was decreasing compared to the wildtype allele		1	0	0	0	1	0
STK11	5'-UTR	NM_000455.5:c.-127T>C	BP6 (Reported benign in ClinVar by laboratories)	Unknown Significance	BP6 (Reported benign in ClinVar by laboratories)<br>BP7 (Beyond +7/-21 and SpliceAI=0)<br>BP4 (CADD=13.67)	Likely Benign	PreTIS score for the mutated allele was decreasing compared to the wildtype allele		81	0	51	0	14	0
STK11	5'-UTR	NM_000455.5:c.-128G>T	No criteria met	Unknown Significance	BP7 (Beyond +7/-21 and SpliceAI=0)<br>BP4 (CADD=12.45)	Likely Benign	PreTIS score for the mutated allele was the same compared to the wildtype allele		2	0	1	0	1	0
STK11	5'-UTR	NM_000455.5:c.-136C>T	No criteria met	Unknown Significance	BP7 (Beyond +7/-21 and SpliceAI=0)<br>BP4 (CADD=4.06)	Likely Benign	PreTIS score for the mutated allele was the same compared to the wildtype allele		2	0	1	0	0	0
STK11	5'-UTR	NM_000455.5:c.-137C>T	BP6 (Laboratory reported it benign in ClinVar)	Unknown Significance	BP6 (Laboratory reported it benign in ClinVar)<br>BP7 (Beyond +7/-21 and SpliceAI=0)<br>BP4 (CADD=8.635)	Likely Benign	PreTIS score for the mutated allele was the same compared to the wildtype allele		1	0	0	0	0	0
STK11	5'-UTR	NM_000455.5:c.-141C>T	No criteria met	Unknown Significance	BP7 (Beyond +7/-21 and SpliceAI=0)<br>BP4 (CADD=2.399)	Likely Benign	PreTIS score for the mutated allele was the same compared to the wildtype allele		3	0	2	0	0	0
STK11	5'-UTR	NM_000455.5:c.-163T>G	No criteria met	Unknown Significance	BP7 (Beyond +7/-21 and SpliceAI=0)<br>BP4 (CADD=14.54)	Likely Benign	PreTIS score for the mutated allele was the same compared to the wildtype allele		1	0	1	0	0	0
STK11	5'-UTR	NM_000455.5:c.-174G>T	PM2_supporting (Absent from GnomAD)	Unknown Significance	BP7 (Beyond +7/-21 and SpliceAI=0)<br>BP4 (CADD=12.59)<br>PM2_supporting (Absent from GnomAD)<br>PP3 (Positive PreTIS assessment)	Unknown Significance	PreTIS score 0.75 for alternative start codon AUC in extension -169 nts. Moderate confidence.<br>PreTIS score increased from 0.69 in wildtype allele to 0.75 in mutated allele		1	0	1	0	0	0
STK11	5'-UTR	NM_000455.5:c.-193C>G	No criteria met	Unknown Significance	BP7 (Beyond +7/-21 and SpliceAI=0)<br>BP4 (CADD=14.36)	Likely Benign	Increase in PreTIS score was less than 0.05 for mutated allele compared to wildtype allele		1	0	0	0	1	0
STK11	5'-UTR	NM_000455.5:c.-201T>G	PM2_supporting (Absent from GnomAD)	Unknown Significance	BP7 (Beyond +7/-21 and SpliceAI=0)<br>BP4 (CADD=11.23)<br>PM2_supporting (Absent from GnomAD)	Likely Benign	PreTIS score for the mutated allele was the same compared to the wildtype allele		1	0	0	0	0	0
STK11	5'-UTR	NM_000455.5:c.-206G>T	No criteria met	Unknown Significance	BP7 (Beyond +7/-21 and SpliceAI=0)<br>BP4 (CADD=12.23)	Likely Benign	PreTIS score for the mutated allele was the same compared to the wildtype allele		1	0	0	0	0	0
STK11	5'-UTR	NM_000455.5:c.-210C>T	PM2_supporting (Only 1 in GnomAD)	Unknown Significance	BP7 (Beyond +7/-21 and SpliceAI=0)<br>BP4 (CADD=8.19)<br>PM2_supporting (Only 1 in GnomAD)	Likely Benign	Increase in PreTIS score was less than 0.05 for mutated allele compared to wildtype allele		1	0	0	0	0	0
STK11	5'-UTR	NM_000455.5:c.-263C>G	PM2_supporting (Absent from GnomAD)	Unknown Significance	BP7 (Beyond +7/-21 and SpliceAI=0)<br>BP4 (CADD=8.51)<br>PM2_supporting (Absent from GnomAD)<br>PP3 (Positive PreTIS assessment)	Unknown Significance	PreTIS score 0.78 for alternative start codon CUG in extension -265 nts. Moderate confidence. Not exiting in the wild type allele		2	0	0	0	1	0
STK11	5'-UTR	NM_000455.5:c.-274C>T	BP6 (A laboratory reproted it benign in ClinVar)	Unknown Significance	BP6 (A laboratory reproted it benign in ClinVar)<br>BP7 (Beyond +7/-21 and SpliceAI=0)<br>BP4 (CADD=5.834)	Likely Benign	PreTIS score for the mutated allele was the same compared to the wildtype allele		2	0	1	0	1	0
STK11	5'-UTR	NM_000455.5:c.-311C>T	BP6 (Reported benign by laboratories in ClinVar)	Unknown Significance	BP6 (Reported benign by laboratories in ClinVar)<br>BP4 (CADD=10.64)<br>BP7 (Beyond +7/-21 and SpliceAI=0)	Likely Benign	PreTIS score for the mutated allele was decreasing compared to the wildtype allele		246	0	145	0	38	0
STK11	5'-UTR	NM_000455.5:c.-365T>C	No criteria met	Unknown Significance	BP7 (Beyond +7/-21 and SpliceAI=0)	Unknown Significance	Increase in PreTIS score was less than 0.05 for mutated allele compared to wildtype allele		1	0	1	0	0	0
STK11	5'-UTR	NM_000455.5:c.-367C>T	PM2_supporting (Only 1 in GnomAD)	Unknown Significance	BP7 (Beyond +7/-21 and SpliceAI=0)<br>PM2_supporting (Only 1 in GnomAD)	Unknown Significance	Increase in PreTIS score was less than 0.05 for mutated allele compared to wildtype allele		1	0	1	0	0	0
STK11	5'-UTR	NM_000455.5:c.-396T>C	No criteria met	Unknown Significance	BP7 (Beyond +7/-21 and SpliceAI=0)<br>BP4 (CADD=10.05)	Likely Benign	PreTIS score for the mutated allele was decreasing compared to the wildtype allele		1	0	1	0	0	0
STK11	5'-UTR	NM_000455.5:c.-400C>T	No criteria met	Unknown Significance	BP7 (Beyond +7/-21 and SpliceAI=0)<br>BP4 (CADD=9.873)	Likely Benign	Increase in PreTIS score was less than 0.05 for mutated allele compared to wildtype allele		1	0	0	0	0	0

STK11	5'-UTR	NM_000455.5:c.-418C>T	PM2_supporting (Absent from GnomAD)	Unknown Significance	BP7 (Beyond +7/-21 and SpliceAI=0)<br>BP4 (CADD=7.372)<br>PM2_supporting (Absent from GnomAD)	Likely Benign	PreTIS score for the mutated allele was the same compared to the wildtype allele		1	0	1	0	0	0
STK11	5'-UTR	NM_000455.5:c.-430C>T	BP6 (Reported benign in ClinVar by laboratories)	Unknown Significance	BP6 (Reported benign in ClinVar by laboratories)<br>BP7 (Beyond +7/-21 and SpliceAI=0)<br>BP4 (CADD=11.2)	Likely Benign	PreTIS score for the mutated allele was the same compared to the wildtype allele		2	0	1	0	0	0
STK11	5'-UTR	NM_000455.5:c.-462C>G	PM2_supporting (Absent from GnomAD)	Unknown Significance	BP7 (Beyond +7/-21 and SpliceAI=0)<br>BP4 (CADD=7.05)<br>PM2_supporting (Absent from GnomAD)	Likely Benign	Increase in PreTIS score was less than 0.05 for mutated allele compared to wildtype allele		1	0	1	0	0	0
STK11	5'-UTR	NM_000455.5:c.-473G>C	No criteria met	Unknown Significance	BP7 (Beyond +7/-21 and SpliceAI=0)<br>BP4 (CADD=8.64)	Likely Benign	PreTIS score for the mutated allele was the same compared to the wildtype allele		3	0	1	0	1	0
STK11	5'-UTR	NM_000455.5:c.-474G>A	No criteria met	Unknown Significance	BP7 (Beyond +7/-21 and SpliceAI=0)<br>BP4 (CADD=7.56)	Likely Benign	PreTIS score for the mutated allele was the same compared to the wildtype allele		10	0	5	0	2	0
STK11	5'-UTR	NM_000455.5:c.-480G>C	PM2_supporting (Only 1 in GnomAD)	Unknown Significance	BP7 (Beyond +7/-21 and SpliceAI=0)<br>BP4 (CADD=7.383)<br>PM2_supporting (Only 1 in GnomAD)	Likely Benign	PreTIS score for the mutated allele was the same compared to the wildtype allele		1	0	1	0	0	0
STK11	5'-UTR	NM_000455.5:c.-501G>A	No criteria met	Unknown Significance	BP7 (Beyond +7/-21 and SpliceAI=0)<br>BP4 (CADD=9.96)	Likely Benign	PreTIS score for the mutated allele was the same compared to the wildtype allele		4	0	3	0	2	0
STK11	5'-UTR	NM_000455.5:c.-507A>C	PM2_supporting (Absent from GnomAD)	Unknown Significance	BP7 (Beyond +7/-21 and SpliceAI=0)<br>BP4 (CADD=9.086)<br>PM2_supporting (Absent from GnomAD)	Likely Benign	PreTIS score for the mutated allele was the same compared to the wildtype allele		1	0	0	0	1	0
STK11	5'-UTR	NM_000455.5:c.-546G>A	No criteria met	Unknown Significance	BP7 (Beyond +7/-21 and SpliceAI=0)<br>BP4 (CADD=11.13)	Likely Benign	PreTIS score for the mutated allele was the same compared to the wildtype allele		2	0	1	0	1	0
STK11	5'-UTR	NM_000455.5:c.-631C>T	PM2_supporting (Only 4 in GnomAD)	Unknown Significance	PM2_supporting (Only 4 in GnomAD)<br>BP7 (Beyond +7/-21 and SpliceAI=0)<br>BP4 (CADD=8.918)	Likely Benign	PreTIS score for the mutated allele was the same compared to the wildtype allele		3	0	2	0	1	0
STK11	5'-UTR	NM_000455.5:c.-81A>G	No criteria met	Unknown Significance	BP7 (Beyond +7/-21 and SpliceAI=0)<br>BP4 (CADD=19.77)	Likely Benign	PreTIS score for the mutated allele was the same compared to the wildtype allele		3	0	2	0	0	0
TP53	5'-UTR	NM_000546.6:c.-48G>A	No criteria met	Unknown Significance	BP7 (Beyond +7/-21 and SpliceAI=0)	Unknown Significance	Increase in PreTIS score was less than 0.05 for mutated allele compared to wildtype allele		1	0	0	0	0	0
TP53	5'-UTR	NM_000546.6:c.-89A>G	No criteria met	Unknown Significance	BP7 (Beyond +7/-21 and SpliceAI=0)<br>BP4 (CADD=10.6)	Likely Benign	Increase in PreTIS score was less than 0.05 for mutated allele compared to wildtype allele		3	0	1	0	0	0

Table S8: All the identified Copy number variants (CNV) found in our study populaiton of 5,923 patients with a clinical suspicion of predispostion to herediary breast- and/or ovarian cancer

BC: Breast cancer
OC: Ovarian cancer
MLPA: Multiplex ligation-dependent probe amplification
VUS: variant with unknown significance

Gene	Region	HGVS c.	Overlapping Exons	MLPA (if avaliable)	CNV State	Classification	Comments regarding assesment	#Samples_Total (N=5,923)	#Samples_BC (N=3,706)	#Samples_OC (N=890)
ATM	11:108235789-108236255	NM_000051.4:c.8851-20_9171delins(894)	62-63		Duplication	Unknown significance	SWEA-2889: Z-score 6.18833 and ratio 1.35022<br>SWEA-0604: Z-score 11.1242 and ratio 1.43142<br>SWEA-1574: Z-score 12.7753 and ratio 1.48583<br>Bins: indicates duplication of exon 62-63 in *ATM*<br>Classified as VUS because we do not know the breakpoints or if the duplication is in tandem	3	2	1
BARD1	2:215593380-215674313	NM_000465.4:c.(?_-114)_(*3031_?)del	All		Het Deletion	Likely pathogenic	SWEA-2871: Z-score -3.94969 and ratio 0.643246<br>Bins: indicates deletion of all *BARD1*	1	1	0
BRCA1	17:41231331-41234612	NM_007294.4:c.4186-20_4358-2700delins(6564)	12	Positive(all four)	Duplication	Unknown significance	SWEA-2895: Z-score 8.09395 and ratio 1.46091<br>SWEA-0901: Z-score 7.24308 and ratio 1.37697<br>SWEA-0512: Z-score 9.65101 ad ratio 1.48717<br>SWEA-2737: Z-score 6.26122 and ratio 1.48632<br>Bins: indicates duplication of exon 12 in *BRCA1*<br>Classified as VUS because we do not know the breakpoints or if the duplication is in tandem	4	3	0
BRCA1	17:41219605-41219732	NM_007294.4:c.4987-20_5074+20del	16	Positive	Het Deletion	Likely pathogenic	SWEA-0270: Z-score -8.78883 and ratio 0.533856<br>Bins: indicates deletion of exon 16 in *BRCA1*	1	1	0
BRCA1	17:41215330-41219732	NM_007294.4:c.4987-20_5193+20del	16-18		Het Deletion	Likely pathogenic	SWEA-2896: Z-score -5.73609 and ratio 0.561666<br>Bins: indicates deletion of exon 16-18 in *BRCA1*	1	0	0
BRCA1	17:41209049-41209172	NM_007294.4:c.5194-20_5277+20delins(248)	19	Positive	Duplication	Unknown significance	SWEA-2897: Z-score 5.72844 and ratio 1.37799<br>Bins: indecates duplikation of exon 19 in *BRCA1*<br>Classified as VUS because we do not know the breakpoints or if the duplication is in tandem	1	1	0
BRCA1	17:41199640-41199740	NM_007294.4:c.5407-20_5467+20del	22	Positive	Het Deletion	Likely pathogenic	SWEA-2898: Z-score -9.83744 and ratio 0.530866<br>Bins: indicates deletion of exon 22 in *BRCA1*	1	1	0
BRCA1	17:41222925-41267816	NM_007294.4:c.81-20_4986+20del	3-15.	Positive(all three)	Het Deletion	Likely pathogenic	SWEA-1830: Z-score -3.90684 and ratio 0.589295<br>SWEA-2741: Z-score -9.91711 and ratio 0.560014<br>SWEA-2137: Z-score -5.10022 and ratio 0.589209<br>Bins indecates deletion of exon 3-15 of *BRCA1*	3	3	0
BRCA2	13:32893194-32893482	NM_000059.4:c.68-20_316+20del	3	Positive	Het Deletion	Likely pathogenic	SWEA-0935: Z-score -10.4588 and ratio 0.510172<br>Bins: indicates deletion of exon 3 in *BRCA2*	1	1	1
BRIP1	17:59793292-59793444	NM_032043.3:c.2380-20_2492+20del	17	Positive	Het Deletion	Likely pathogenic	SWEA-2899: Z-score -727794 and ratio 0.486089<br>Bins: indicates deletion of exon 17 in *BRIP1*	1	0	1
BRIP1	17:59861611-59937288	NM_032043.3:c.94-20_1935+20delins(159374)	3-13.		Duplication	Unknown significance	SWEA-0152: Z-score 4.41269 and ratio 1.20378<br>Bins: indecates duplication of exon 3-13 of *BRIP1*<br>Classified as VUS because we do not know the breakpoints or if the duplication is in tandem	1	1	0
CHEK2	22:29092869-29095945	NM_007194.4:c.909-20_1095+20del	9-10.		Het Deletion	Likely pathogenic	SWEA-2363: Z-score -13.0443 and ratio 0.496254<br>SWEA-2892: Z-score -13.6579 and ratio 0.511339<br>SWEA-2893: Z-score -16.0059 and ratio 0.516891<br>SWEA-2894: Z-score -6.83297 and ratio 0.552624<br>Bins: indecates deletion of exon 9-10 of *CHEK2*	4	2	1
PALB2	16:23637537-23637738	NM_024675.4:c.2587-20_2748+20del	7	Positive(SWEA-0823)	Het Deletion	Likely pathogenic	SWEA-0823: Z-score -10.7398 and ratio 0.537104<br>SWEA-1929: Z-score -14.2029 and ratio 0.502184<br>Bins: indecates a deletion of exon 7 in *PALB2*	2	1	0
RAD51C	17:56787200-56787371	NM_058216.3:c.706-20_837+20del	5	Positive	Het Deletion	Likely pathogenic	SWEA-2900: Z-score -10.9286 and ratio 0.535749<br>Bins: indicates deletion of exon 5 in *RAD51C*	1	0	1
RAD51D	17:33427952-33428075	NM_002878.4:c.904-20_987del	10	Positive	Het Deletion	Likely pathogenic	SWEA-2890: Z-score -8.56361 and ratio 0.12376<br>SWEA-2891: Z-score -9.82928 and ratio 0.511553<br>Bins: indicates deletion of exon 10 in *RAD51D*	2	0	2

Table S9: Association analysis of variants with unknown significance (VUS) found in our study populaiton of 5,923 patients with a clinical suspicion of predispostion to heredirary breast- and/or ovarian cancer.
BC: Breast cancer
Bold letter: indecates significant FDR (FDR <0.05)
GnomAD:Genome Aggregation Database
NFE: Non-Finnish European
OC: Ovarian cancer

			Number of samples and frequencies												Association study: GnomAD 2.1 Swedish vs		
			Study population						Genome Aggregation Database (GnomAD) groups						Total group	Breast cancer group	Ovarian cancer group
**Gene**	**Sequence Ontology**	**HGVSc.**	**Samples Total (N=5,923)**	**Frequency Total**	**Samples BC (N=3,706)**	**Frequency BC**	**Samples OC (N=890)**	**Frequency OC**	**Gnomad 2.1 (Swedish) (N=13,067)**	**Gnomad 2.1 (Sweden) frequency**	**Gnomad 2.1 (NFE) (N=56,885)**	**Gnomad 2.1 (NFE) frequency**	**Gnomad 4.0 (NFE) (N=590,031)**	**GnomAD 4.0 (NFE) frequency**	**OR (95% CI, FDR)**	**OR (95% CI, FDR)**	**OR (95% CI, FDR)**
ATM	missense_variant	NM_000051.4:c.1021G>A	1	0.000168833	0	0	0	0	1	7.65287E-05	1	1.75793E-05	8	1.3559E-05	2.21(0.03-172.97, 0.57702)	NA	NA
ATM	missense_variant	NM_000051.4:c.115A>G	1	0.000168833	1	0.00026983	0	0	1	7.65287E-05	6	0.000105476	56	9.491E-05	2.21(0.03-172.97, 0.57702)	3.53(0.04-276.22, 0.58964)	NA
ATM	splice_region_variant	NM_000051.4:c.1236-8T>C	2	0.000337667	2	0.00053967	0	0	1	7.65287E-05	1	1.75793E-05	2	3.3897E-06	4.41(0.23-259.98, 0.36694)	7.05(0.37-415.12, 0.36074)	NA
ATM	missense_variant	NM_000051.4:c.1273G>T	1	0.000168833	1	0.00026983	0	0	0	0	0	0	1	1.6948E-06	∞(0.06-∞,0.36694)	∞(0.09-∞,0.36074)	NA
ATM	missense_variant	NM_000051.4:c.131A>G	1	0.000168833	1	0.00026983	0	0	0	0	0	0	0	0	∞(0.06-∞,0.36694)	∞(0.09-∞,0.36074)	NA
ATM	missense_variant	NM_000051.4:c.1516G>T	1	0.000168833	1	0.00026983	0	0	0	0	8	0.000140635	27	4.576E-05	∞(0.06-∞,0.36694)	∞(0.09-∞,0.36074)	NA
ATM	missense_variant	NM_000051.4:c.1595G>A	1	0.000168833	0	0	0	0	0	0	26	0.000457062	171	0.00028982	∞(0.06-∞,0.36694)	NA	NA
ATM	missense_variant	NM_000051.4:c.1600C>A	1	0.000168833	1	0.00026983	0	0	0	0	0	0	0	0	∞(0.06-∞,0.36694)	∞(0.09-∞,0.36074)	NA
ATM	missense_variant	NM_000051.4:c.1601C>T	1	0.000168833	1	0.00026983	0	0	0	0	0	0	1	1.6948E-06	∞(0.06-∞,0.36694)	∞(0.09-∞,0.36074)	NA
ATM	synonymous_variant	NM_000051.4:c.1638G>A	1	0.000168833	0	0	0	0	0	0	0	0	0	0	∞(0.06-∞,0.36694)	NA	NA
ATM	missense_variant	NM_000051.4:c.1705A>C	1	0.000168833	0	0	1	0.0011236	0	0	0	0	0	0	∞(0.06-∞,0.36694)	NA	∞(0.38-∞,0.47826)
ATM	missense_variant	NM_000051.4:c.1709T>C	4	0.000675333	3	0.0008095	0	0	1	7.65287E-05	2	3.51587E-05	2	3.3897E-06	8.83(0.87-433.87, 0.33405)	10.58(0.85-553.91, 0.36074)	NA
ATM	missense_variant	NM_000051.4:c.1727T>C	2	0.000337667	1	0.00026983	1	0.0011236	12	0.000918344	14	0.000246111	90	0.00015253	0.37(0.04-1.65, 0.36694)	0.29(0.01-1.99, 0.51762)	1.22(0.03-8.29, 1)
ATM	synonymous_variant	NM_000051.4:c.1773T>C	1	0.000168833	1	0.00026983	0	0	0	0	21	0.000369166	243	0.00041184	∞(0.06-∞,0.36694)	∞(0.09-∞,0.36074)	NA
ATM	missense_variant	NM_000051.4:c.1814A>G	3	0.0005065	2	0.00053967	0	0	1	7.65287E-05	1	1.75793E-05	6	1.0169E-05	6.62(0.53-346.94, 0.36694)	7.05(0.37-415.12, 0.36074)	NA
ATM	synonymous_variant	NM_000051.4:c.2040C>T	2	0.000337667	2	0.00053967	0	0	1	7.65287E-05	10	0.000175793	61	0.00010338	4.41(0.23-259.98, 0.36694)	7.05(0.37-415.12, 0.36074)	NA
ATM	missense_variant	NM_000051.4:c.2071G>C	3	0.0005065	2	0.00053967	1	0.0011236	1	7.65287E-05	1	1.75793E-05	2	3.3897E-06	6.62(0.53-346.94, 0.36694)	7.05(0.37-415.12, 0.36074)	14.69(0.19-1141.88, 0.77983)
ATM	missense_variant	NM_000051.4:c.2075G>A	1	0.000168833	1	0.00026983	0	0	0	0	1	1.75793E-05	3	5.0845E-06	∞(0.06-∞,0.36694)	∞(0.09-∞,0.36074)	NA
ATM	splice_region_variant	NM_000051.4:c.2124+7T>A	1	0.000168833	1	0.00026983	1	0.0011236	0	0	0	0	1	1.6948E-06	∞(0.06-∞,0.36694)	∞(0.09-∞,0.36074)	∞(0.38-∞,0.47826)
ATM	missense_variant	NM_000051.4:c.2149C>T	1	0.000168833	1	0.00026983	1	0.0011236	3	0.000229586	4	7.03173E-05	27	4.576E-05	0.74(0.01-9.16, 1)	1.18(0.02-14.64, 1)	4.9(0.09-61.12, 1)
ATM	missense_variant	NM_000051.4:c.214A>G	1	0.000168833	1	0.00026983	0	0	0	0	0	0	0	0	∞(0.06-∞,0.36694)	∞(0.09-∞,0.36074)	NA
ATM	missense_variant	NM_000051.4:c.2150G>A	1	0.000168833	1	0.00026983	0	0	0	0	2	3.51587E-05	7	1.1864E-05	∞(0.06-∞,0.36694)	∞(0.09-∞,0.36074)	NA
ATM	missense_variant	NM_000051.4:c.221G>A	1	0.000168833	1	0.00026983	0	0	0	0	0	0	7	1.1864E-05	∞(0.06-∞,0.36694)	∞(0.09-∞,0.36074)	NA
ATM	missense_variant	NM_000051.4:c.2221T>C	2	0.000337667	1	0.00026983	0	0	0	0	0	0	0	0	∞(0.41-∞,0.36694)	∞(0.09-∞,0.36074)	NA
ATM	missense_variant	NM_000051.4:c.2248A>G	2	0.000337667	2	0.00053967	0	0	0	0	0	0	1	1.6948E-06	∞(0.41-∞,0.36694)	∞(0.66-∞,0.36074)	NA
ATM	splice_region_variant	NM_000051.4:c.2251-11A>G	3	0.0005065	0	0	0	0	1	7.65287E-05	1	1.75793E-05	3	5.0845E-06	6.62(0.53-346.94, 0.36694)	NA	NA
ATM	missense_variant	NM_000051.4:c.2266G>T	1	0.000168833	1	0.00026983	0	0	0	0	0	0	0	0	∞(0.06-∞,0.36694)	∞(0.09-∞,0.36074)	NA
ATM	missense_variant	NM_000051.4:c.2305G>A	1	0.000168833	1	0.00026983	0	0	0	0	0	0	0	0	∞(0.06-∞,0.36694)	∞(0.09-∞,0.36074)	NA
ATM	splice_region_variant	NM_000051.4:c.2466+7A>G	1	0.000168833	1	0.00026983	0	0	0	0	0	0	2	3.3897E-06	∞(0.06-∞,0.36694)	∞(0.09-∞,0.36074)	NA
ATM	missense_variant	NM_000051.4:c.2476A>C	2	0.000337667	1	0.00026983	0	0	0	0	10	0.000175793	86	0.00014576	∞(0.41-∞,0.36694)	∞(0.09-∞,0.36074)	NA
ATM	missense_variant	NM_000051.4:c.2485C>T	1	0.000168833	1	0.00026983	0	0	0	0	0	0	0	0	∞(0.06-∞,0.36694)	∞(0.09-∞,0.36074)	NA
ATM	missense_variant	NM_000051.4:c.2494C>T	1	0.000168833	1	0.00026983	0	0	2	0.000153057	37	0.000650435	298	0.00050506	1.1(0.02-21.2, 1)	1.76(0.03-33.88, 0.76453)	NA
ATM	missense_variant	NM_000051.4:c.2500G>C	1	0.000168833	1	0.00026983	0	0	0	0	0	0	0	0	∞(0.06-∞,0.36694)	∞(0.09-∞,0.36074)	NA
ATM	missense_variant	NM_000051.4:c.2519A>T	1	0.000168833	1	0.00026983	0	0	0	0	2	3.51587E-05	18	3.0507E-05	∞(0.06-∞,0.36694)	∞(0.09-∞,0.36074)	NA
ATM	missense_variant	NM_000051.4:c.2564T>C	1	0.000168833	1	0.00026983	0	0	0	0	2	3.51587E-05	3	5.0845E-06	∞(0.06-∞,0.36694)	∞(0.09-∞,0.36074)	NA
ATM	missense_variant	NM_000051.4:c.2602G>A	1	0.000168833	0	0	0	0	0	0	0	0	0	0	∞(0.06-∞,0.36694)	NA	NA
ATM	missense_variant	NM_000051.4:c.2650C>G	1	0.000168833	1	0.00026983	0	0	0	0	0	0	0	0	∞(0.06-∞,0.36694)	∞(0.09-∞,0.36074)	NA
ATM	missense_variant	NM_000051.4:c.2693T>C	1	0.000168833	1	0.00026983	0	0	0	0	0	0	1	1.6948E-06	∞(0.06-∞,0.36694)	∞(0.09-∞,0.36074)	NA
ATM	missense_variant	NM_000051.4:c.2716T>A	1	0.000168833	0	0	0	0	0	0	0	0	2	3.3897E-06	∞(0.06-∞,0.36694)	NA	NA
ATM	missense_variant	NM_000051.4:c.2804C>T	1	0.000168833	1	0.00026983	0	0	2	0.000153057	11	0.000193373	140	0.00023728	1.1(0.02-21.2, 1)	1.76(0.03-33.88, 0.76453)	NA
ATM	missense_variant	NM_000051.4:c.280A>G	1	0.000168833	0	0	1	0.0011236	0	0	1	1.75793E-05	6	1.0169E-05	∞(0.06-∞,0.36694)	NA	∞(0.38-∞,0.47826)
ATM	missense_variant	NM_000051.4:c.283C>A	1	0.000168833	0	0	0	0	0	0	0	0	0	0	∞(0.06-∞,0.36694)	NA	NA
ATM	missense_variant	NM_000051.4:c.2867G>A	1	0.000168833	1	0.00026983	0	0	1	7.65287E-05	2	3.51587E-05	3	5.0845E-06	2.21(0.03-172.97, 0.57702)	3.53(0.04-276.22, 0.58964)	NA
ATM	missense_variant	NM_000051.4:c.2924A>G	2	0.000337667	2	0.00053967	0	0	0	0	1	1.75793E-05	5	8.4741E-06	∞(0.41-∞,0.36694)	∞(0.66-∞,0.36074)	NA
ATM	missense_variant	NM_000051.4:c.2932T>C	9	0.0015195	3	0.0008095	3	0.00337079	12	0.000918344	79	0.001388767	748	0.00126773	1.66(0.62-4.28, 0.36694)	0.88(0.16-3.27, 1)	3.68(0.67-13.67, 0.48873)
ATM	missense_variant	NM_000051.4:c.2942G>A	1	0.000168833	1	0.00026983	1	0.0011236	0	0	3	5.2738E-05	14	2.3728E-05	∞(0.06-∞,0.36694)	∞(0.09-∞,0.36074)	∞(0.38-∞,0.47826)
ATM	missense_variant	NM_000051.4:c.295A>G	1	0.000168833	1	0.00026983	0	0	0	0	3	5.2738E-05	21	3.5591E-05	∞(0.06-∞,0.36694)	∞(0.09-∞,0.36074)	NA

ATM	missense_variant	NM_000051.4:c.2960G>A	1	0.000168833	0	0	0	0	0	0	0	0	0	0	∞(0.06-∞,0.36694)	NA	NA
ATM	missense_variant	NM_000051.4:c.2983C>G	1	0.000168833	0	0	1	0.0011236	0	0	0	0	2	3.3897E-06	∞(0.06-∞,0.36694)	NA	∞(0.38-∞,0.47826)
ATM	splice_region_variant	NM_000051.4:c.-30-5dupT	1	0.000168833	1	0.00026983	1	0.0011236	1	7.65287E-05	10	0.000175793	222	0.00037625	2.21(0.03-172.97, 0.57702)	3.53(0.04-276.22, 0.58964)	14.69(0.19-1141.88, 0.77983)
ATM	missense_variant	NM_000051.4:c.3061G>C	4	0.000675333	1	0.00026983	0	0	0	0	0	0	1	1.6948E-06	∞(1.46-∞,0.18158)	∞(0.09-∞,0.36074)	NA
ATM	missense_variant	NM_000051.4:c.3071C>T	2	0.000337667	2	0.00053967	0	0	6	0.000459172	6	0.000105476	8	1.3559E-05	0.74(0.07-4.11, 1)	1.18(0.12-6.58, 0.98241)	NA
ATM	splice_region_variant	NM_000051.4:c.3078-10T>G	1	0.000168833	0	0	1	0.0011236	0	0	0	0	0	0	∞(0.06-∞,0.36694)	NA	∞(0.38-∞,0.47826)
ATM	missense_variant	NM_000051.4:c.3121G>A	1	0.000168833	1	0.00026983	1	0.0011236	0	0	0	0	0	0	∞(0.06-∞,0.36694)	∞(0.09-∞,0.36074)	∞(0.38-∞,0.47826)
ATM	splice_region_variant	NM_000051.4:c.3154-4G>A	2	0.000337667	2	0.00053967	0	0	1	7.65287E-05	35	0.000615276	236	0.00039998	4.41(0.23-259.98, 0.36694)	7.05(0.37-415.12, 0.36074)	NA
ATM	splice_region_variant	NM_000051.4:c.3154-4G>T	3	0.0005065	2	0.00053967	1	0.0011236	2	0.000153057	3	5.2738E-05	10	1.6948E-05	3.31(0.38-39.65, 0.36694)	3.53(0.26-48.66, 0.36074)	7.35(0.12-141.34, 1)
ATM	splice_region_variant	NM_000051.4:c.-31G>A	1	0.000168833	1	0.00026983	0	0	0	0	0	0	0	0	∞(0.06-∞,0.36694)	∞(0.09-∞,0.36074)	NA
ATM	missense_variant	NM_000051.4:c.3260T>C	1	0.000168833	0	0	0	0	0	0	1	1.75793E-05	2	3.3897E-06	∞(0.06-∞,0.36694)	NA	NA
ATM	missense_variant	NM_000051.4:c.3281A>G	1	0.000168833	1	0.00026983	0	0	0	0	2	3.51587E-05	7	1.1864E-05	∞(0.06-∞,0.36694)	∞(0.09-∞,0.36074)	NA
ATM	missense_variant	NM_000051.4:c.3376A>G	2	0.000337667	1	0.00026983	0	0	0	0	0	0	0	0	∞(0.41-∞,0.36694)	∞(0.09-∞,0.36074)	NA
ATM	missense_variant	NM_000051.4:c.3407A>G	2	0.000337667	0	0	1	0.0011236	0	0	0	0	10	1.6948E-05	∞(0.41-∞,0.36694)	NA	∞(0.38-∞,0.47826)
ATM	missense_variant	NM_000051.4:c.3496C>T	1	0.000168833	1	0.00026983	0	0	0	0	0	0	0	0	∞(0.06-∞,0.36694)	∞(0.09-∞,0.36074)	NA
ATM	missense_variant	NM_000051.4:c.3519G>C	6	0.001013	2	0.00053967	3	0.00337079	0	0	0	0	2	3.3897E-06	∞(2.60-∞,**0.03642**)	∞(0.66-∞,0.36074)	∞(6.08-∞,**0.04136**)
ATM	missense_variant	NM_000051.4:c.3676G>C	3	0.0005065	2	0.00053967	0	0	0	0	2	3.51587E-05	7	1.1864E-05	∞(0.91-∞,0.30332)	∞(0.66-∞,0.36074)	NA
ATM	missense_variant	NM_000051.4:c.3743A>G	1	0.000168833	1	0.00026983	0	0	0	0	3	5.2738E-05	14	2.3728E-05	∞(0.06-∞,0.36694)	∞(0.09-∞,0.36074)	NA
ATM	missense_variant	NM_000051.4:c.3806A>G	1	0.000168833	1	0.00026983	0	0	0	0	4	7.03173E-05	103	0.00017457	∞(0.06-∞,0.36694)	∞(0.09-∞,0.36074)	NA
ATM	missense_variant	NM_000051.4:c.3843T>G	1	0.000168833	1	0.00026983	0	0	0	0	0	0	2	3.3897E-06	∞(0.06-∞,0.36694)	∞(0.09-∞,0.36074)	NA
ATM	missense_variant	NM_000051.4:c.3964C>A	1	0.000168833	0	0	0	0	0	0	6	0.000105476	66	0.00011186	∞(0.06-∞,0.36694)	NA	NA
ATM	missense_variant	NM_000051.4:c.4060C>A	2	0.000337667	2	0.00053967	0	0	5	0.000382643	44	0.00077349	273	0.00046269	0.88(0.08-5.39, 1)	1.41(0.13-8.62, 0.93457)	NA
ATM	missense_variant	NM_000051.4:c.4082A>G	1	0.000168833	1	0.00026983	0	0	0	0	0	0	0	0	∞(0.06-∞,0.36694)	∞(0.09-∞,0.36074)	NA
ATM	missense_variant	NM_000051.4:c.4148C>T	1	0.000168833	1	0.00026983	0	0	0	0	6	0.000105476	63	0.00010677	∞(0.06-∞,0.36694)	∞(0.09-∞,0.36074)	NA
ATM	missense_variant	NM_000051.4:c.4150C>A	1	0.000168833	1	0.00026983	0	0	0	0	0	0	0	0	∞(0.06-∞,0.36694)	∞(0.09-∞,0.36074)	NA
ATM	missense_variant	NM_000051.4:c.4264A>T	1	0.000168833	0	0	1	0.0011236	0	0	0	0	0	0	∞(0.06-∞,0.36694)	NA	∞(0.38-∞,0.47826)
ATM	missense_variant	NM_000051.4:c.4324T>C	21	0.003545501	14	0.00377766	3	0.00337079	36	0.002755032	74	0.00130087	599	0.0010152	1.29(0.71-2.27, 0.45702)	1.37(0.68-2.61, 0.49907)	1.22(0.24-3.89, 1)
ATM	missense_variant	NM_000051.4:c.4375G>A	1	0.000168833	1	0.00026983	0	0	0	0	11	0.000193373	248	0.00042032	∞(0.06-∞,0.36694)	∞(0.09-∞,0.36074)	NA
ATM	missense_variant	NM_000051.4:c.4385C>T	1	0.000168833	1	0.00026983	0	0	0	0	0	0	0	0	∞(0.06-∞,0.36694)	∞(0.09-∞,0.36074)	NA
ATM	missense_variant	NM_000051.4:c.4388T>G	1	0.000168833	1	0.00026983	0	0	1	7.65287E-05	62	0.001089918	385	0.00065251	2.21(0.03-172.97, 0.57702)	3.53(0.04-276.22, 0.58964)	NA
ATM	missense_variant	NM_000051.4:c.4397G>A	1	0.000168833	0	0	0	0	0	0	0	0	5	8.4741E-06	∞(0.06-∞,0.36694)	NA	NA
ATM	in-frame_deletion	NM_000051.4:c.4442_4444delCTT	1	0.000168833	0	0	0	0	0	0	1	1.75793E-05	2	3.3897E-06	∞(0.06-∞,0.36694)	NA	NA
ATM	missense_variant	NM_000051.4:c.4465C>T	1	0.000168833	1	0.00026983	0	0	0	0	2	3.51587E-05	17	2.8812E-05	∞(0.06-∞,0.36694)	∞(0.09-∞,0.36074)	NA
ATM	missense_variant	NM_000051.4:c.4547A>G	2	0.000337667	0	0	0	0	0	0	0	0	1	1.6948E-06	∞(0.41-∞,0.36694)	NA	NA
ATM	missense_variant	NM_000051.4:c.4561G>C	1	0.000168833	1	0.00026983	0	0	0	0	2	3.51587E-05	33	5.5929E-05	∞(0.06-∞,0.36694)	∞(0.09-∞,0.36074)	NA
ATM	missense_variant	NM_000051.4:c.4588G>C	1	0.000168833	1	0.00026983	0	0	0	0	0	0	0	0	∞(0.06-∞,0.36694)	∞(0.09-∞,0.36074)	NA
ATM	splice_region_variant	NM_000051.4:c.4612-8A>T	1	0.000168833	0	0	1	0.0011236	0	0	0	0	0	0	∞(0.06-∞,0.36694)	NA	∞(0.38-∞,0.47826)
ATM	missense_variant	NM_000051.4:c.4639A>G	2	0.000337667	2	0.00053967	0	0	0	0	0	0	2	3.3897E-06	∞(0.41-∞,0.36694)	∞(0.66-∞,0.36074)	NA
ATM	missense_variant	NM_000051.4:c.4662C>A	1	0.000168833	1	0.00026983	0	0	3	0.000229586	4	7.03173E-05	4	6.7793E-06	0.74(0.01-9.16, 1)	1.18(0.02-14.64, 1)	NA
ATM	missense_variant	NM_000051.4:c.4724G>A	1	0.000168833	0	0	0	0	3	0.000229586	14	0.000246111	136	0.0002305	0.74(0.01-9.16, 1)	NA	NA
ATM	missense_variant	NM_000051.4:c.4768C>T	1	0.000168833	0	0	1	0.0011236	21	0.001607102	28	0.000492221	185	0.00031354	0.1(0-0.65, 0.11911)	NA	0.7(0.02-4.36, 1)
ATM	missense_variant	NM_000051.4:c.4846G>T	1	0.000168833	1	0.00026983	0	0	0	0	0	0	0	0	∞(0.06-∞,0.36694)	∞(0.09-∞,0.36074)	NA
ATM	splice_region_variant	NM_000051.4:c.496+4T>C	1	0.000168833	1	0.00026983	0	0	0	0	22	0.000386745	122	0.00020677	∞(0.06-∞,0.36694)	∞(0.09-∞,0.36074)	NA
ATM	missense_variant	NM_000051.4:c.4972G>T	1	0.000168833	1	0.00026983	0	0	0	0	0	0	0	0	∞(0.06-∞,0.36694)	∞(0.09-∞,0.36074)	NA
ATM	splice_region_variant	NM_000051.4:c.497-3A>T	1	0.000168833	0	0	1	0.0011236	0	0	0	0	1	1.6948E-06	∞(0.06-∞,0.36694)	NA	∞(0.38-∞,0.47826)
ATM	missense_variant	NM_000051.4:c.5089A>G	1	0.000168833	0	0	0	0	1	7.65287E-05	10	0.000175793	292	0.00049489	2.21(0.03-172.97, 0.57702)	NA	NA
ATM	missense_variant	NM_000051.4:c.5262G>T	2	0.000337667	1	0.00026983	0	0	0	0	3	5.2738E-05	17	2.8812E-05	∞(0.41-∞,0.36694)	∞(0.09-∞,0.36074)	NA
ATM	missense_variant	NM_000051.4:c.5278A>G	2	0.000337667	1	0.00026983	0	0	0	0	4	7.03173E-05	36	6.1014E-05	∞(0.41-∞,0.36694)	∞(0.09-∞,0.36074)	NA
ATM	splice_region_variant	NM_000051.4:c.5319+3C>A	1	0.000168833	1	0.00026983	0	0	0	0	3	5.2738E-05	3	5.0845E-06	∞(0.06-∞,0.36694)	∞(0.09-∞,0.36074)	NA
ATM	in-frame_deletion	NM_000051.4:c.5417_5422delTAAAGA	3	0.0005065	3	0.0008095	0	0	0	0	0	0	0	0	∞(0.91-∞,0.30332)	∞(1.46-∞,0.20697)	NA
ATM	missense_variant	NM_000051.4:c.550A>G	1	0.000168833	1	0.00026983	0	0	0	0	0	0	4	6.7793E-06	∞(0.06-∞,0.36694)	∞(0.09-∞,0.36074)	NA
ATM	missense_variant	NM_000051.4:c.5511T>A	1	0.000168833	0	0	0	0	0	0	0	0	0	0	∞(0.06-∞,0.36694)	NA	NA
ATM	missense_variant	NM_000051.4:c.5590A>G	1	0.000168833	1	0.00026983	0	0	0	0	0	0	0	0	∞(0.06-∞,0.36694)	∞(0.09-∞,0.36074)	NA
ATM	missense_variant	NM_000051.4:c.5618G>A	1	0.000168833	1	0.00026983	0	0	0	0	0	0	15	2.5422E-05	∞(0.06-∞,0.36694)	∞(0.09-∞,0.36074)	NA
ATM	missense_variant	NM_000051.4:c.5645G>A	3	0.0005065	1	0.00026983	0	0	0	0	0	0	5	8.4741E-06	∞(0.91-∞,0.30332)	∞(0.09-∞,0.36074)	NA
ATM	missense_variant	NM_000051.4:c.5740G>C	2	0.000337667	2	0.00053967	0	0	0	0	0	0	0	0	∞(0.41-∞,0.36694)	∞(0.66-∞,0.36074)	NA
ATM	splice_region_variant	NM_000051.4:c.5762+6G>A	3	0.0005065	1	0.00026983	1	0.0011236	8	0.000612229	9	0.000158214	25	4.2371E-05	0.83(0.14-3.45, 1)	0.44(0.01-3.29, 0.98241)	1.84(0.04-13.72, 1)
ATM	missense_variant	NM_000051.4:c.584C>T	1	0.000168833	1	0.00026983	0	0	0	0	1	1.75793E-05	2	3.3897E-06	∞(0.06-∞,0.36694)	∞(0.09-∞,0.36074)	NA
ATM	missense_variant	NM_000051.4:c.5870A>G	1	0.000168833	1	0.00026983	0	0	0	0	0	0	2	3.3897E-06	∞(0.06-∞,0.36694)	∞(0.09-∞,0.36074)	NA
ATM	missense_variant	NM_000051.4:c.5890A>G	3	0.0005065	3	0.0008095	0	0	2	0.000153057	12	0.000210952	96	0.0001627	3.31(0.38-39.65, 0.36694)	5.29(0.61-63.43, 0.36074)	NA
ATM	missense_variant	NM_000051.4:c.6025T>C	1	0.000168833	0	0	0	0	0	0	2	3.51587E-05	33	5.5929E-05	∞(0.06-∞,0.36694)	NA	NA

ATM	missense_variant	NM_000051.4:c.610G>A	2	0.000337667	1	0.00026983	1	0.0011236	0	0	14	0.000246111	100	0.00016948	∞(0.41-∞,0.36694)	∞(0.09-∞,0.36074)	∞(0.38-∞,0.47826)
ATM	missense_variant	NM_000051.4:c.6490G>C	1	0.000168833	1	0.00026983	0	0	0	0	0	0	0	0	∞(0.06-∞,0.36694)	∞(0.09-∞,0.36074)	NA
ATM	missense_variant	NM_000051.4:c.654G>T	1	0.000168833	1	0.00026983	0	0	0	0	0	0	0	0	∞(0.06-∞,0.36694)	∞(0.09-∞,0.36074)	NA
ATM	missense_variant	NM_000051.4:c.6554T>C	3	0.0005065	2	0.00053967	0	0	1	7.65287E-05	2	3.51587E-05	6	1.0169E-05	6.62(0.53-346.94, 0.36694)	7.05(0.37-415.12, 0.36074)	NA
ATM	splice_region_variant	NM_000051.4:c.6572+4T>C	1	0.000168833	1	0.00026983	0	0	0	0	7	0.000123055	22	3.7286E-05	∞(0.06-∞,0.36694)	∞(0.09-∞,0.36074)	NA
ATM	missense_variant	NM_000051.4:c.6652A>C	2	0.000337667	2	0.00053967	0	0	1	7.65287E-05	2	3.51587E-05	6	1.0169E-05	4.41(0.23-259.98, 0.36694)	7.05(0.37-415.12, 0.36074)	NA
ATM	missense_variant	NM_000051.4:c.670A>G	4	0.000675333	1	0.00026983	1	0.0011236	0	0	20	0.000351587	156	0.00026439	∞(1.46-∞,0.18158)	∞(0.09-∞,0.36074)	∞(0.38-∞,0.47826)
ATM	in-frame-delins	NM_000051.4:c.6736_6755delinsCA	1	0.000168833	1	0.00026983	0	0	0	0	0	0	0	0	∞(0.06-∞,0.36694)	∞(0.09-∞,0.36074)	NA
ATM	missense_variant	NM_000051.4:c.6820G>A	1	0.000168833	0	0	0	0	2	0.000153057	24	0.000421904	215	0.00036439	1.1(0.02-21.2, 1)	NA	NA
ATM	missense_variant	NM_000051.4:c.6895T>C	2	0.000337667	1	0.00026983	0	0	0	0	0	0	0	0	∞(0.41-∞,0.36694)	∞(0.09-∞,0.36074)	NA
ATM	missense_variant	NM_000051.4:c.68G>A	2	0.000337667	2	0.00053967	0	0	0	0	0	0	12	2.0338E-05	∞(0.41-∞,0.36694)	∞(0.66-∞,0.36074)	NA
ATM	missense_variant	NM_000051.4:c.7075A>C	1	0.000168833	1	0.00026983	0	0	0	0	0	0	0	0	∞(0.06-∞,0.36694)	∞(0.09-∞,0.36074)	NA
ATM	missense_variant	NM_000051.4:c.7223C>T	1	0.000168833	1	0.00026983	0	0	0	0	1	1.75793E-05	10	1.6948E-05	∞(0.06-∞,0.36694)	∞(0.09-∞,0.36074)	NA
ATM	missense_variant	NM_000051.4:c.7309T>C	1	0.000168833	1	0.00026983	0	0	0	0	0	0	1	1.6948E-06	∞(0.06-∞,0.36694)	∞(0.09-∞,0.36074)	NA
ATM	synonymous_variant	NM_000051.4:c.7314A>C	1	0.000168833	1	0.00026983	0	0	2	0.000153057	5	8.78966E-05	47	7.9657E-05	1.1(0.02-21.2, 1)	1.76(0.03-33.88, 0.76453)	NA
ATM	missense_variant	NM_000051.4:c.7316T>C	1	0.000168833	1	0.00026983	0	0	0	0	1	1.75793E-05	5	8.4741E-06	∞(0.06-∞,0.36694)	∞(0.09-∞,0.36074)	NA
ATM	missense_variant	NM_000051.4:c.7429G>A	1	0.000168833	1	0.00026983	0	0	0	0	0	0	0	0	∞(0.06-∞,0.36694)	∞(0.09-∞,0.36074)	NA
ATM	missense_variant	NM_000051.4:c.7475T>G	7	0.001181834	4	0.00107933	0	0	1	7.65287E-05	24	0.000421904	251	0.0004254	15.46(1.99-694.4, 0.05)	14.11(1.4-692.59, 0.20697)	NA
ATM	missense_variant	NM_000051.4:c.749G>A	2	0.000337667	2	0.00053967	0	0	2	0.000153057	9	0.000158214	83	0.00014067	2.21(0.16-30.45, 0.64306)	3.53(0.26-48.66, 0.36074)	NA
ATM	missense_variant	NM_000051.4:c.7502A>G	1	0.000168833	0	0	0	0	0	0	1	1.75793E-05	2	3.3897E-06	∞(0.06-∞,0.36694)	NA	NA
ATM	splice_region_variant	NM_000051.4:c.7516-9dupT	1	0.000168833	0	0	1	0.0011236	2	0.000153057	20	0.000351587	107	0.00018135	1.1(0.02-21.2, 1)	NA	7.35(0.12-141.34, 1)
ATM	synonymous_variant	NM_000051.4:c.7521C>T	7	0.001181834	1	0.00026983	4	0.00449438	0	0	5	8.78966E-05	90	0.00015253	∞(3.18-∞,**0.01375**)	∞(0.09-∞,0.36074)	∞(9.71-∞,**0.00393**)
ATM	missense_variant	NM_000051.4:c.7740A>C	1	0.000168833	1	0.00026983	0	0	0	0	5	8.78966E-05	48	8.1352E-05	∞(0.06-∞,0.36694)	∞(0.09-∞,0.36074)	NA
ATM	missense_variant	NM_000051.4:c.7775C>G	3	0.0005065	2	0.00053967	1	0.0011236	0	0	3	5.2738E-05	49	8.3046E-05	∞(0.91-∞,0.30332)	∞(0.66-∞,0.36074)	∞(0.38-∞,0.47826)
ATM	missense_variant	NM_000051.4:c.7871G>C	1	0.000168833	0	0	0	0	0	0	3	5.2738E-05	4	6.7793E-06	∞(0.06-∞,0.36694)	NA	NA
ATM	missense_variant	NM_000051.4:c.7875T>G	1	0.000168833	0	0	0	0	1	7.65287E-05	1	1.75793E-05	11	1.8643E-05	2.21(0.03-172.97, 0.57702)	NA	NA
ATM	missense_variant	NM_000051.4:c.7876G>C	1	0.000168833	0	0	0	0	1	7.65287E-05	1	1.75793E-05	11	1.8643E-05	2.21(0.03-172.97, 0.57702)	NA	NA
ATM	missense_variant	NM_000051.4:c.7912T>G	1	0.000168833	0	0	0	0	0	0	0	0	0	0	∞(0.06-∞,0.36694)	NA	NA
ATM	missense_variant	NM_000051.4:c.7919C>T	2	0.000337667	2	0.00053967	0	0	1	7.65287E-05	23	0.000404325	361	0.00061183	4.41(0.23-259.98, 0.36694)	7.05(0.37-415.12, 0.36074)	NA
ATM	missense_variant	NM_000051.4:c.8014G>T	1	0.000168833	0	0	0	0	0	0	0	0	1	1.6948E-06	∞(0.06-∞,0.36694)	NA	NA
ATM	missense_variant	NM_000051.4:c.8071C>T	1	0.000168833	1	0.00026983	0	0	1	7.65287E-05	3	5.2738E-05	10	1.6948E-05	2.21(0.03-172.97, 0.57702)	3.53(0.04-276.22, 0.58964)	NA
ATM	missense_variant	NM_000051.4:c.8072G>A	1	0.000168833	1	0.00026983	0	0	0	0	1	1.75793E-05	6	1.0169E-05	∞(0.06-∞,0.36694)	∞(0.09-∞,0.36074)	NA
ATM	missense_variant	NM_000051.4:c.8096C>T	2	0.000337667	0	0	2	0.00224719	0	0	0	0	0	0	∞(0.41-∞,0.36694)	NA	∞(2.76-∞,0.21442)
ATM	missense_variant	NM_000051.4:c.8153G>A	1	0.000168833	1	0.00026983	0	0	0	0	0	0	0	0	∞(0.06-∞,0.36694)	∞(0.09-∞,0.36074)	NA
ATM	missense_variant	NM_000051.4:c.8165T>C	1	0.000168833	0	0	0	0	0	0	0	0	0	0	∞(0.06-∞,0.36694)	NA	NA
ATM	missense_variant	NM_000051.4:c.8327T>C	3	0.0005065	1	0.00026983	1	0.0011236	1	7.65287E-05	6	0.000105476	25	4.2371E-05	6.62(0.53-346.94, 0.36694)	3.53(0.04-276.22, 0.58964)	14.69(0.19-1141.88, 0.77983)
ATM	missense_variant	NM_000051.4:c.8354A>G	1	0.000168833	1	0.00026983	0	0	0	0	0	0	0	0	∞(0.06-∞,0.36694)	∞(0.09-∞,0.36074)	NA
ATM	missense_variant	NM_000051.4:c.8428A>C	7	0.001181834	7	0.00188883	0	0	0	0	7	0.000123055	46	7.7962E-05	∞(3.18-∞,**0.01375**)	∞(5.09-∞,**0.00410**)	NA
ATM	in-frame_deletion	NM_000051.4:c.8504_8518delGCATGGAAAAATTCT	2	0.000337667	1	0.00026983	1	0.0011236	0	0	0	0	0	0	∞(0.41-∞,0.36694)	∞(0.09-∞,0.36074)	∞(0.38-∞,0.47826)
ATM	missense_variant	NM_000051.4:c.8560C>T	1	0.000168833	1	0.00026983	0	0	3	0.000229586	24	0.000421904	235	0.00039828	0.74(0.01-9.16, 1)	1.18(0.02-14.64, 1)	NA
ATM	missense_variant	NM_000051.4:c.8624A>C	1	0.000168833	0	0	0	0	0	0	0	0	1	1.6948E-06	∞(0.06-∞,0.36694)	NA	NA
ATM	missense_variant	NM_000051.4:c.8625T>G	1	0.000168833	0	0	1	0.0011236	0	0	0	0	0	0	∞(0.06-∞,0.36694)	NA	∞(0.38-∞,0.47826)
ATM	missense_variant	NM_000051.4:c.8732C>T	2	0.000337667	0	0	0	0	0	0	0	0	0	0	∞(0.41-∞,0.36694)	NA	NA
ATM	missense_variant	NM_000051.4:c.8734A>G	1	0.000168833	0	0	1	0.0011236	18	0.001377516	46	0.000808649	257	0.00043557	0.12(0-0.78, 0.19367)	NA	0.82(0.02-5.18, 1)
ATM	splice_region_variant	NM_000051.4:c.8787-15T>G	1	0.000168833	0	0	0	0	0	0	0	0	0	0	∞(0.06-∞,0.36694)	NA	NA
ATM	splice_region_variant	NM_000051.4:c.8787-6C>T	2	0.000337667	2	0.00053967	0	0	0	0	0	0	2	3.3897E-06	∞(0.41-∞,0.36694)	∞(0.66-∞,0.36074)	NA
ATM	splice_region_variant	NM_000051.4:c.8850+4A>C	2	0.000337667	2	0.00053967	0	0	4	0.000306115	6	0.000105476	54	9.1521E-05	1.1(0.1-7.7, 1)	1.76(0.16-12.31, 0.88768)	NA
ATM	missense_variant	NM_000051.4:c.8965C>G	1	0.000168833	1	0.00026983	0	0	0	0	4	7.03173E-05	17	2.8812E-05	∞(0.06-∞,0.36694)	∞(0.09-∞,0.36074)	NA
ATM	missense_variant	NM_000051.4:c.9023G>C	2	0.000337667	0	0	1	0.0011236	0	0	0	0	1	1.6948E-06	∞(0.41-∞,0.36694)	NA	∞(0.38-∞,0.47826)
ATM	missense_variant	NM_000051.4:c.902G>A	3	0.0005065	1	0.00026983	0	0	2	0.000153057	13	0.000228531	137	0.00023219	3.31(0.38-39.65, 0.36694)	1.76(0.03-33.88, 0.76453)	NA
ATM	missense_variant	NM_000051.4:c.9032T>G	1	0.000168833	1	0.00026983	0	0	0	0	0	0	2	3.3897E-06	∞(0.06-∞,0.36694)	∞(0.09-∞,0.36074)	NA
ATM	missense_variant	NM_000051.4:c.9086G>A	5	0.000844167	4	0.00107933	0	0	6	0.000459172	28	0.000492221	608	0.00103045	1.84(0.44-7.24, 0.3933)	2.35(0.49-9.92, 0.39509)	NA
ATM	missense_variant	NM_000051.4:c.9086G>T	1	0.000168833	1	0.00026983	1	0.0011236	1	7.65287E-05	1	1.75793E-05	2	3.3897E-06	2.21(0.03-172.97, 0.57702)	3.53(0.04-276.22, 0.58964)	14.69(0.19-1141.88, 0.77983)
ATM	missense_variant	NM_000051.4:c.9156G>T	1	0.000168833	0	0	0	0	0	0	0	0	0	0	∞(0.06-∞,0.36694)	NA	NA
ATM	synonymous_variant	NM_000051.4:c.9168G>A	3	0.0005065	2	0.00053967	0	0	0	0	0	0	2	3.3897E-06	∞(0.91-∞,0.30332)	∞(0.66-∞,0.36074)	NA
ATM	missense_variant	NM_000051.4:c.94C>T	1	0.000168833	0	0	1	0.0011236	0	0	2	3.51587E-05	21	3.5591E-05	∞(0.06-∞,0.36694)	NA	∞(0.38-∞,0.47826)
ATM	missense_variant	NM_000051.4:c.986G>A	1	0.000168833	1	0.00026983	0	0	0	0	0	0	0	0	∞(0.06-∞,0.36694)	∞(0.09-∞,0.36074)	NA

BARD1	missense_variant	NM_000465.4:c.1013C>A	1	0.000168833	0	0	0	0	0	0	0	0	1	1.6948E-06	∞(0.06-∞,0.36694)	NA	NA
BARD1	in-frame_deletion	NM_000465.4:c.1075_1095del	176	0.029714672	116	0.03130059	23	0.0258427	286	0.021887197	2009	0.035316867	20053	0.03398635	1.37(1.12-1.66, 0.05)	1.44(1.15-1.8, 0.05976)	1.19(0.74-1.83, 1)
BARD1	missense_variant	NM_000465.4:c.1115T>G	1	0.000168833	0	0	0	0	0	0	0	0	0	0	∞(0.06-∞,0.36694)	NA	NA
BARD1	missense_variant	NM_000465.4:c.1282G>A	1	0.000168833	1	0.00026983	0	0	0	0	0	0	0	0	∞(0.06-∞,0.36694)	∞(0.09-∞,0.36074)	NA
BARD1	missense_variant	NM_000465.4:c.1307C>G	1	0.000168833	1	0.00026983	0	0	1	7.65287E-05	2	3.51587E-05	3	5.0845E-06	2.21(0.03-172.97, 0.57702)	3.53(0.04-276.22, 0.58964)	NA
BARD1	splice_region_variant	NM_000465.4:c.1315-15T>C	1	0.000168833	1	0.00026983	0	0	0	0	0	0	0	0	∞(0.06-∞,0.36694)	∞(0.09-∞,0.36074)	NA
BARD1	missense_variant	NM_000465.4:c.13C>T	1	0.000168833	0	0	0	0	0	0	0	0	2	3.3897E-06	∞(0.06-∞,0.36694)	NA	NA
BARD1	missense_variant	NM_000465.4:c.1498G>A	1	0.000168833	1	0.00026983	0	0	0	0	1	1.75793E-05	15	2.5422E-05	∞(0.06-∞,0.36694)	∞(0.09-∞,0.36074)	NA
BARD1	splice_region_variant	NM_000465.4:c.1569-13C>G	7	0.001181834	5	0.00134916	2	0.00224719	0	0	5	8.78966E-05	12	2.0338E-05	∞(3.18-∞,**0.01375**)	∞(3.23-∞,**0.02803**)	∞(2.76-∞,0.21442)
BARD1	splice_region_variant	NM_000465.4:c.158+3A>C	1	0.000168833	1	0.00026983	0	0	0	0	0	0	0	0	∞(0.06-∞,0.36694)	∞(0.09-∞,0.36074)	NA
BARD1	missense_variant	NM_000465.4:c.1586G>A	1	0.000168833	1	0.00026983	0	0	2	0.000153057	4	7.03173E-05	12	2.0338E-05	1.1(0.02-21.2, 1)	1.76(0.03-33.88, 0.76453)	NA
BARD1	synonymous_variant	NM_000465.4:c.1593C>T	5	0.000844167	5	0.00134916	0	0	3	0.000229586	7	0.000123055	25	4.2371E-05	3.68(0.72-23.7, 0.36694)	5.88(1.14-37.9, 0.28557)	NA
BARD1	missense_variant	NM_000465.4:c.1598A>T	1	0.000168833	1	0.00026983	0	0	0	0	0	0	1	1.6948E-06	∞(0.06-∞,0.36694)	∞(0.09-∞,0.36074)	NA
BARD1	missense_variant	NM_000465.4:c.1646A>G	1	0.000168833	0	0	1	0.0011236	8	0.000612229	9	0.000158214	18	3.0507E-05	0.28(0.01-2.06, 0.36694)	NA	1.84(0.04-13.72, 1)
BARD1	missense_variant	NM_000465.4:c.1693C>T	1	0.000168833	1	0.00026983	0	0	1	7.65287E-05	2	3.51587E-05	22	3.7286E-05	2.21(0.03-172.97, 0.57702)	3.53(0.04-276.22, 0.58964)	NA
BARD1	missense_variant	NM_000465.4:c.1718T>C	1	0.000168833	1	0.00026983	0	0	0	0	5	8.78966E-05	32	5.4234E-05	∞(0.06-∞,0.36694)	∞(0.09-∞,0.36074)	NA
BARD1	missense_variant	NM_000465.4:c.1757G>T	3	0.0005065	3	0.0008095	0	0	1	7.65287E-05	3	5.2738E-05	6	1.0169E-05	6.62(0.53-346.94, 0.36694)	10.58(0.85-553.91, 0.36074)	NA
BARD1	synonymous_variant	NM_000465.4:c.1767A>G	1	0.000168833	0	0	0	0	0	0	0	0	0	0	∞(0.06-∞,0.36694)	NA	NA
BARD1	missense_variant	NM_000465.4:c.1813A>G	1	0.000168833	0	0	1	0.0011236	0	0	0	0	1	1.6948E-06	∞(0.06-∞,0.36694)	NA	∞(0.38-∞,0.47826)
BARD1	missense_variant	NM_000465.4:c.1915T>C	7	0.001181834	6	0.001619	0	0	0	0	5	8.78966E-05	64	0.00010847	∞(3.18-∞,**0.01375**)	∞(4.16-∞,**0.007953**)	NA
BARD1	missense_variant	NM_000465.4:c.1943A>G	2	0.000337667	2	0.00053967	0	0	0	0	0	0	0	0	∞(0.41-∞,0.36694)	∞(0.66-∞,0.36074)	NA
BARD1	missense_variant	NM_000465.4:c.1961C>T	1	0.000168833	1	0.00026983	0	0	0	0	0	0	0	0	∞(0.06-∞,0.36694)	∞(0.09-∞,0.36074)	NA
BARD1	missense_variant	NM_000465.4:c.2125C>T	2	0.000337667	0	0	0	0	0	0	0	0	25	4.2371E-05	∞(0.41-∞,0.36694)	NA	NA
BARD1	missense_variant	NM_000465.4:c.2153A>G	1	0.000168833	1	0.00026983	0	0	0	0	0	0	1	1.6948E-06	∞(0.06-∞,0.36694)	∞(0.09-∞,0.36074)	NA
BARD1	missense_variant	NM_000465.4:c.2311G>C	1	0.000168833	1	0.00026983	0	0	0	0	0	0	0	0	∞(0.06-∞,0.36694)	∞(0.09-∞,0.36074)	NA
BARD1	in-frame_deletion	NM_000465.4:c.26_40delACCGGCAGCCGAGGA	1	0.000168833	1	0.00026983	0	0	0	0	4	7.03173E-05	54	9.1521E-05	∞(0.06-∞,0.36694)	∞(0.09-∞,0.36074)	NA
BARD1	missense_variant	NM_000465.4:c.386G>C	1	0.000168833	0	0	0	0	0	0	0	0	0	0	∞(0.06-∞,0.36694)	NA	NA
BARD1	missense_variant	NM_000465.4:c.464G>C	1	0.000168833	0	0	1	0.0011236	0	0	0	0	0	0	∞(0.06-∞,0.36694)	NA	∞(0.38-∞,0.47826)
BARD1	missense_variant	NM_000465.4:c.54C>G	1	0.000168833	1	0.00026983	0	0	0	0	0	0	8	1.3559E-05	∞(0.06-∞,0.36694)	∞(0.09-∞,0.36074)	NA
BARD1	missense_variant	NM_000465.4:c.580A>T	1	0.000168833	0	0	0	0	0	0	2	3.51587E-05	5	8.4741E-06	∞(0.06-∞,0.36694)	NA	NA
BARD1	missense_variant	NM_000465.4:c.617A>G	1	0.000168833	1	0.00026983	0	0	0	0	0	0	0	0	∞(0.06-∞,0.36694)	∞(0.09-∞,0.36074)	NA
BARD1	missense_variant	NM_000465.4:c.632T>C	2	0.000337667	1	0.00026983	1	0.0011236	2	0.000153057	3	5.2738E-05	28	4.7455E-05	2.21(0.16-30.45, 0.64306)	1.76(0.03-33.88, 0.76453)	7.35(0.12-141.34, 1)
BARD1	missense_variant	NM_000465.4:c.647A>T	1	0.000168833	0	0	0	0	0	0	0	0	0	0	∞(0.06-∞,0.36694)	NA	NA
BARD1	missense_variant	NM_000465.4:c.652T>A	1	0.000168833	0	0	0	0	0	0	0	0	0	0	∞(0.06-∞,0.36694)	NA	NA
BARD1	missense_variant	NM_000465.4:c.659T>C	3	0.0005065	3	0.0008095	1	0.0011236	5	0.000382643	16	0.000281269	224	0.00037964	1.32(0.21-6.81, 0.76501)	2.12(0.33-10.89, 0.58964)	2.94(0.06-26.31, 1)
BARD1	missense_variant	NM_000465.4:c.667G>C	1	0.000168833	1	0.00026983	0	0	0	0	0	0	0	0	∞(0.06-∞,0.36694)	∞(0.09-∞,0.36074)	NA
BARD1	missense_variant	NM_000465.4:c.701A>T	2	0.000337667	2	0.00053967	0	0	0	0	0	0	0	0	∞(0.41-∞,0.36694)	∞(0.66-∞,0.36074)	NA
BARD1	missense_variant	NM_000465.4:c.709C>G	4	0.000675333	2	0.00053967	0	0	6	0.000459172	17	0.000298849	195	0.00033049	1.47(0.31-6.21, 0.57702)	1.18(0.12-6.58, 0.98241)	NA
BARD1	missense_variant	NM_000465.4:c.722C>G	1	0.000168833	1	0.00026983	0	0	0	0	0	0	0	0	∞(0.06-∞,0.36694)	∞(0.09-∞,0.36074)	NA
BRCA1	missense_variant	NM_007294.4:c.1030G>A	4	0.000675333	3	0.0008095	1	0.0011236	2	0.000153057	2	3.51587E-05	4	6.7793E-06	4.41(0.63-48.8, 0.36694)	5.29(0.61-63.43, 0.36074)	7.35(0.12-141.34, 1)
BRCA1	missense_variant	NM_007294.4:c.1100C>G	1	0.000168833	1	0.00026983	0	0	0	0	0	0	0	0	∞(0.06-∞,0.36694)	∞(0.09-∞,0.36074)	NA
BRCA1	missense_variant	NM_007294.4:c.1117A>C	1	0.000168833	1	0.00026983	0	0	0	0	0	0	0	0	∞(0.06-∞,0.36694)	∞(0.09-∞,0.36074)	NA
BRCA1	missense_variant	NM_007294.4:c.1589A>C	2	0.000337667	0	0	0	0	0	0	0	0	0	0	∞(0.41-∞,0.36694)	NA	NA
BRCA1	missense_variant	NM_007294.4:c.1786C>G	1	0.000168833	1	0.00026983	0	0	0	0	2	3.51587E-05	4	6.7793E-06	∞(0.06-∞,0.36694)	∞(0.09-∞,0.36074)	NA
BRCA1	missense_variant	NM_007294.4:c.2993T>A	1	0.000168833	1	0.00026983	0	0	0	0	0	0	2	3.3897E-06	∞(0.06-∞,0.36694)	∞(0.09-∞,0.36074)	NA
BRCA1	splice_region_variant	NM_007294.4:c.302-15C>G	2	0.000337667	0	0	2	0.00224719	0	0	0	0	0	0	∞(0.41-∞,0.36694)	NA	∞(2.76-∞,0.21442)
BRCA1	missense_variant	NM_007294.4:c.3230G>A	1	0.000168833	1	0.00026983	0	0	0	0	0	0	1	1.6948E-06	∞(0.06-∞,0.36694)	∞(0.09-∞,0.36074)	NA
BRCA1	missense_variant	NM_007294.4:c.3454G>A	1	0.000168833	0	0	1	0.0011236	1	7.65287E-05	10	0.000175793	43	7.2878E-05	2.21(0.03-172.97, 0.57702)	NA	14.69(0.19-1141.88, 0.77983)
BRCA1	missense_variant	NM_007294.4:c.3889T>A	1	0.000168833	1	0.00026983	0	0	0	0	0	0	0	0	∞(0.06-∞,0.36694)	∞(0.09-∞,0.36074)	NA
BRCA1	splice_region_variant	NM_007294.4:c.4096+3A>G	11	0.001857167	8	0.00215866	5	0.00561798	0	0	0	0	3	5.0845E-06	∞(5.54-∞,**0.00065**)	∞(6.03-∞,**0.00136**)	∞(13.50-∞,**0.00005**)
BRCA1	missense_variant	NM_007294.4:c.4166G>A	1	0.000168833	1	0.00026983	0	0	0	0	0	0	0	0	∞(0.06-∞,0.36694)	∞(0.09-∞,0.36074)	NA
BRCA1	missense_variant	NM_007294.4:c.4193A>G	2	0.000337667	1	0.00026983	1	0.0011236	0	0	0	0	2	3.3897E-06	∞(0.41-∞,0.36694)	∞(0.09-∞,0.36074)	∞(0.38-∞,0.47826)
BRCA1	missense_variant	NM_007294.4:c.4625C>G	1	0.000168833	1	0.00026983	0	0	6	0.000459172	9	0.000158214	36	6.1014E-05	0.37(0.01-3.03, 0.52042)	0.59(0.01-4.85, 1)	NA
BRCA1	splice_region_variant	NM_007294.4:c.4676-8C>G	6	0.001013	4	0.00107933	1	0.0011236	4	0.000306115	7	0.000123055	38	6.4403E-05	3.31(0.78-15.96, 0.36694)	3.53(0.66-18.95, 0.36074)	3.67(0.07-37.14, 1)
BRCA1	missense_variant	NM_007294.4:c.5050A>G	2	0.000337667	1	0.00026983	0	0	0	0	0	0	0	0	∞(0.41-∞,0.36694)	∞(0.09-∞,0.36074)	NA
BRCA1	splice_region_variant	NM_007294.4:c.5075-7T>C	1	0.000168833	0	0	0	0	0	0	1	1.75793E-05	0	0	∞(0.06-∞,0.36694)	NA	NA
BRCA1	missense_variant	NM_007294.4:c.5348T>C	1	0.000168833	0	0	1	0.0011236	0	0	0	0	0	0	∞(0.06-∞,0.36694)	NA	∞(0.38-∞,0.47826)
BRCA1	missense_variant	NM_007294.4:c.587A>T	1	0.000168833	0	0	0	0	0	0	0	0	7	1.1864E-05	∞(0.06-∞,0.36694)	NA	NA

BRCA1	missense_variant	NM_007294.4:c.656A>T	1	0.000168833	1	0.00026983	0	0	0	0	0	0	0	0	∞(0.06-∞,0.36694)	∞(0.09-∞,0.36074)	NA
BRCA1	missense_variant	NM_007294.4:c.922A>T	1	0.000168833	1	0.00026983	0	0	0	0	0	0	0	0	∞(0.06-∞,0.36694)	∞(0.09-∞,0.36074)	NA
BRCA2	in-frame_deletion	NM_000059.4:c.3733_3738delGAGAAT	1	0.000168833	1	0.00026983	0	0	0	0	0	0	0	0	∞(0.06-∞,0.36694)	∞(0.09-∞,0.36074)	NA
BRCA2	synonymous_variant	NM_000059.4:c.436C>T	1	0.000168833	1	0.00026983	0	0	0	0	0	0	2	3.3897E-06	∞(0.06-∞,0.36694)	∞(0.09-∞,0.36074)	NA
BRCA2	splice_region_variant	NM_000059.4:c.517-7C>T	1	0.000168833	1	0.00026983	0	0	0	0	0	0	4	6.7793E-06	∞(0.06-∞,0.36694)	∞(0.09-∞,0.36074)	NA
BRCA2	in-frame_deletion	NM_000059.4:c.5272_5274delAAT	7	0.001181834	6	0.001619	0	0	5	0.000382643	5	8.78966E-05	12	2.0338E-05	3.09(0.84-12.35, 0.36694)	4.24(1.08-17.56, 0.3246)	NA
BRCA2	missense_variant	NM_000059.4:c.565G>T	1	0.000168833	1	0.00026983	0	0	0	0	0	0	0	0	∞(0.06-∞,0.36694)	∞(0.09-∞,0.36074)	NA
BRCA2	splice_region_variant	NM_000059.4:c.6842-4T>C	1	0.000168833	1	0.00026983	0	0	0	0	0	0	0	0	∞(0.06-∞,0.36694)	∞(0.09-∞,0.36074)	NA
BRCA2	missense_variant	NM_000059.4:c.6842G>A	1	0.000168833	0	0	0	0	0	0	0	0	1	1.6948E-06	∞(0.06-∞,0.36694)	NA	NA
BRCA2	missense_variant	NM_000059.4:c.7448G>A	1	0.000168833	0	0	0	0	0	0	4	7.03173E-05	30	5.0845E-05	∞(0.06-∞,0.36694)	NA	NA
BRCA2	missense_variant	NM_000059.4:c.7504C>T	1	0.000168833	1	0.00026983	0	0	4	0.000306115	6	0.000105476	32	5.4234E-05	0.55(0.01-5.57, 1)	0.88(0.02-8.91, 1)	NA
BRCA2	in-frame_deletion	NM_000059.4:c.7584_7586delAGG	1	0.000168833	1	0.00026983	0	0	0	0	0	0	0	0	∞(0.06-∞,0.36694)	∞(0.09-∞,0.36074)	NA
BRCA2	missense_variant	NM_000059.4:c.7616A>G	2	0.000337667	1	0.00026983	0	0	2	0.000153057	4	7.03173E-05	5	8.4741E-06	2.21(0.16-30.45, 0.64306)	1.76(0.03-33.88, 0.76453)	NA
BRCA2	missense_variant	NM_000059.4:c.7883T>C	1	0.000168833	1	0.00026983	1	0.0011236	0	0	0	0	3	5.0845E-06	∞(0.06-∞,0.36694)	∞(0.09-∞,0.36074)	∞(0.38-∞,0.47826)
BRCA2	missense_variant	NM_000059.4:c.7895C>A	1	0.000168833	1	0.00026983	0	0	0	0	0	0	0	0	∞(0.06-∞,0.36694)	∞(0.09-∞,0.36074)	NA
BRCA2	missense_variant	NM_000059.4:c.8116A>G	1	0.000168833	0	0	0	0	0	0	0	0	1	1.6948E-06	∞(0.06-∞,0.36694)	NA	NA
BRCA2	missense_variant	NM_000059.4:c.8131G>A	1	0.000168833	1	0.00026983	0	0	0	0	0	0	0	0	∞(0.06-∞,0.36694)	∞(0.09-∞,0.36074)	NA
BRCA2	missense_variant	NM_000059.4:c.8140C>G	1	0.000168833	1	0.00026983	0	0	0	0	0	0	2	3.3897E-06	∞(0.06-∞,0.36694)	∞(0.09-∞,0.36074)	NA
BRCA2	missense_variant	NM_000059.4:c.8351G>A	2	0.000337667	0	0	0	0	0	0	1	1.75793E-05	23	3.8981E-05	∞(0.41-∞,0.36694)	NA	NA
BRCA2	missense_variant	NM_000059.4:c.8452G>A	1	0.000168833	1	0.00026983	0	0	0	0	1	1.75793E-05	3	5.0845E-06	∞(0.06-∞,0.36694)	∞(0.09-∞,0.36074)	NA
BRCA2	splice_region_variant	NM_000059.4:c.8488-6T>G	1	0.000168833	0	0	0	0	0	0	0	0	0	0	∞(0.06-∞,0.36694)	NA	NA
BRCA2	missense_variant	NM_000059.4:c.8663G>A	1	0.000168833	1	0.00026983	0	0	1	7.65287E-05	1	1.75793E-05	8	1.3559E-05	2.21(0.03-172.97, 0.57702)	3.53(0.04-276.22, 0.58964)	NA
BRCA2	missense_variant	NM_000059.4:c.8902A>G	1	0.000168833	1	0.00026983	0	0	0	0	0	0	11	1.8643E-05	∞(0.06-∞,0.36694)	∞(0.09-∞,0.36074)	NA
BRCA2	splice_region_variant	NM_000059.4:c.8954-17C>T	2	0.000337667	1	0.00026983	0	0	0	0	0	0	0	0	∞(0.41-∞,0.36694)	∞(0.09-∞,0.36074)	NA
BRCA2	missense_variant	NM_000059.4:c.8957T>C	1	0.000168833	0	0	0	0	0	0	0	0	2	3.3897E-06	∞(0.06-∞,0.36694)	NA	NA
BRCA2	missense_variant	NM_000059.4:c.9171C>G	1	0.000168833	0	0	0	0	0	0	1	1.75793E-05	1	1.6948E-06	∞(0.06-∞,0.36694)	NA	NA
BRIP1	missense_variant	NM_032043.3:c.1093A>G	1	0.000168833	1	0.00026983	0	0	1	7.65287E-05	1	1.75793E-05	3	5.0845E-06	2.21(0.03-172.97, 0.57702)	3.53(0.04-276.22, 0.58964)	NA
BRIP1	missense_variant	NM_032043.3:c.1247G>A	1	0.000168833	0	0	0	0	0	0	0	0	11	1.8643E-05	∞(0.06-∞,0.36694)	NA	NA
BRIP1	missense_variant	NM_032043.3:c.1255C>T	10	0.001688334	7	0.00188883	1	0.0011236	8	0.000612229	54	0.000949284	333	0.00056438	2.76(0.98-8.05, 0.35473)	3.09(0.95-9.76, 0.36074)	1.84(0.04-13.72, 1)
BRIP1	missense_variant	NM_032043.3:c.1352C>T	1	0.000168833	1	0.00026983	0	0	0	0	0	0	0	0	∞(0.06-∞,0.36694)	∞(0.09-∞,0.36074)	NA
BRIP1	missense_variant	NM_032043.3:c.139C>G	7	0.001181834	3	0.0008095	3	0.00337079	3	0.000229586	52	0.000914125	537	0.00091012	5.15(1.18-30.89, 0.19367)	3.53(0.47-26.35, 0.36074)	14.72(1.97-110.28, 0.21442)
BRIP1	missense_variant	NM_032043.3:c.1433A>G	1	0.000168833	0	0	0	0	0	0	0	0	11	1.8643E-05	∞(0.06-∞,0.36694)	NA	NA
BRIP1	missense_variant	NM_032043.3:c.1547T>A	1	0.000168833	0	0	1	0.0011236	0	0	0	0	0	0	∞(0.06-∞,0.36694)	NA	∞(0.38-∞,0.47826)
BRIP1	missense_variant	NM_032043.3:c.1660C>G	1	0.000168833	0	0	0	0	0	0	2	3.51587E-05	35	5.9319E-05	∞(0.06-∞,0.36694)	NA	NA
BRIP1	in-frame_deletion	NM_032043.3:c.1687_1689delGAT	11	0.001857167	9	0.00242849	2	0.00224719	2	0.000153057	2	3.51587E-05	0	0	12.16(2.65-112.95, **0.00861**)	15.9(3.29-151.18, **0.00528**)	14.7(1.06-202.8, 0.47825)
BRIP1	splice_region_variant	NM_032043.3:c.1795-9T>G	1	0.000168833	1	0.00026983	0	0	3	0.000229586	4	7.03173E-05	23	3.8981E-05	0.74(0.01-9.16, 1)	1.18(0.02-14.64, 1)	NA
BRIP1	missense_variant	NM_032043.3:c.1899C>G	1	0.000168833	0	0	1	0.0011236	0	0	0	0	8	1.3559E-05	∞(0.06-∞,0.36694)	NA	∞(0.38-∞,0.47826)
BRIP1	splice_region_variant	NM_032043.3:c.1935+4_1935+7delAGTT	1	0.000168833	1	0.00026983	0	0	1	7.65287E-05	1	1.75793E-05	2	3.3897E-06	2.21(0.03-172.97, 0.57702)	3.53(0.04-276.22, 0.58964)	NA
BRIP1	missense_variant	NM_032043.3:c.2146A>G	1	0.000168833	1	0.00026983	0	0	0	0	0	0	0	0	∞(0.06-∞,0.36694)	∞(0.09-∞,0.36074)	NA
BRIP1	missense_variant	NM_032043.3:c.2233G>A	1	0.000168833	0	0	0	0	0	0	1	1.75793E-05	19	3.2202E-05	∞(0.06-∞,0.36694)	NA	NA
BRIP1	missense_variant	NM_032043.3:c.2325T>G	1	0.000168833	0	0	0	0	0	0	1	1.75793E-05	14	2.3728E-05	∞(0.06-∞,0.36694)	NA	NA
BRIP1	missense_variant	NM_032043.3:c.2440C>T	1	0.000168833	1	0.00026983	0	0	0	0	2	3.51587E-05	11	1.8643E-05	∞(0.06-∞,0.36694)	∞(0.09-∞,0.36074)	NA
BRIP1	missense_variant	NM_032043.3:c.2441G>A	11	0.001857167	6	0.001619	2	0.00224719	2	0.000153057	8	0.000140635	53	8.9826E-05	12.16(2.65-112.95, **0.00861**)	10.59(1.89-107.39, 0.07719)	14.7(1.06-202.8, 0.47825)
BRIP1	synonymous_variant	NM_032043.3:c.2484C>T	1	0.000168833	0	0	0	0	0	0	0	0	0	0	∞(0.06-∞,0.36694)	NA	NA
BRIP1	splice_region_variant	NM_032043.3:c.2493-10T>A	4	0.000675333	4	0.00107933	0	0	1	7.65287E-05	1	1.75793E-05	1	1.6948E-06	8.83(0.87-433.87, 0.33405)	14.11(1.4-692.59, 0.20697)	NA
BRIP1	in-frame_deletion	NM_032043.3:c.258_269delTTGTTGTGCATG	2	0.000337667	1	0.00026983	0	0	0	0	3	5.2738E-05	101	0.00017118	∞(0.41-∞,0.36694)	∞(0.09-∞,0.36074)	NA
BRIP1	missense_variant	NM_032043.3:c.2840A>G	1	0.000168833	0	0	0	0	0	0	0	0	0	0	∞(0.06-∞,0.36694)	NA	NA
BRIP1	splice_donor_variant	NM_032043.3:c.2905+2T>A	1	0.000168833	1	0.00026983	0	0	0	0	0	0	1	1.6948E-06	∞(0.06-∞,0.36694)	∞(0.09-∞,0.36074)	NA
BRIP1	missense_variant	NM_032043.3:c.293A>G	1	0.000168833	1	0.00026983	0	0	0	0	4	7.03173E-05	51	8.6436E-05	∞(0.06-∞,0.36694)	∞(0.09-∞,0.36074)	NA
BRIP1	missense_variant	NM_032043.3:c.3050C>T	4	0.000675333	4	0.00107933	0	0	0	0	1	1.75793E-05	50	8.4741E-05	∞(1.46-∞,0.18158)	∞(2.33-∞,0.07719)	NA
BRIP1	synonymous_variant	NM_032043.3:c.3069C>T	1	0.000168833	1	0.00026983	0	0	1	7.65287E-05	8	0.000140635	81	0.00013728	2.21(0.03-172.97, 0.57702)	3.53(0.04-276.22, 0.58964)	NA
BRIP1	missense_variant	NM_032043.3:c.3103C>T	2	0.000337667	1	0.00026983	0	0	1	7.65287E-05	11	0.000193373	80	0.00013559	4.41(0.23-259.98, 0.36694)	3.53(0.04-276.22, 0.58964)	NA
BRIP1	missense_variant	NM_032043.3:c.3149C>A	1	0.000168833	1	0.00026983	0	0	0	0	6	0.000105476	17	2.8812E-05	∞(0.06-∞,0.36694)	∞(0.09-∞,0.36074)	NA
BRIP1	missense_variant	NM_032043.3:c.316C>T	3	0.0005065	1	0.00026983	0	0	9	0.000688758	20	0.000351587	88	0.00014914	0.74(0.13-2.95, 0.81909)	0.39(0.01-2.83, 0.99084)	NA
BRIP1	missense_variant	NM_032043.3:c.3205C>G	1	0.000168833	1	0.00026983	0	0	0	0	0	0	0	0	∞(0.06-∞,0.36694)	∞(0.09-∞,0.36074)	NA
BRIP1	missense_variant	NM_032043.3:c.3254G>C	1	0.000168833	1	0.00026983	0	0	0	0	0	0	0	0	∞(0.06-∞,0.36694)	∞(0.09-∞,0.36074)	NA
BRIP1	missense_variant	NM_032043.3:c.3371A>G	1	0.000168833	1	0.00026983	0	0	0	0	0	0	2	3.3897E-06	∞(0.06-∞,0.36694)	∞(0.09-∞,0.36074)	NA
BRIP1	disruptive_in-frame_insertion	NM_032043.3:c.3404_3405insCAG	1	0.000168833	1	0.00026983	0	0	0	0	0	0	0	0	∞(0.06-∞,0.36694)	∞(0.09-∞,0.36074)	NA
BRIP1	missense_variant	NM_032043.3:c.3444C>A	2	0.000337667	2	0.00053967	0	0	0	0	24	0.000421904	376	0.00063725	∞(0.41-∞,0.36694)	∞(0.66-∞,0.36074)	NA

BRIP1	synonymous_variant	NM_032043.3:c.3498A>G	1	0.000168833	1	0.00026983	0	0	0	0	0	0	0	0	∞(0.06-∞,0.36694)	∞(0.09-∞,0.36074)	NA
BRIP1	synonymous_variant	NM_032043.3:c.3519T>C	1	0.000168833	0	0	1	0.0011236	0	0	0	0	0	0	∞(0.06-∞,0.36694)	NA	∞(0.38-∞,0.47826)
BRIP1	missense_variant	NM_032043.3:c.3529A>C	1	0.000168833	0	0	0	0	8	0.000612229	9	0.000158214	11	1.8643E-05	0.28(0.01-2.06, 0.36694)	NA	NA
BRIP1	splice_region_variant	NM_032043.3:c.380-17T>A	2	0.000337667	0	0	1	0.0011236	14	0.001071401	41	0.000720752	289	0.0004898	0.31(0.03-1.37, 0.36694)	NA	1.05(0.02-6.91, 1)
BRIP1	missense_variant	NM_032043.3:c.415T>G	2	0.000337667	1	0.00026983	0	0	0	0	9	0.000158214	88	0.00014914	∞(0.41-∞,0.36694)	∞(0.09-∞,0.36074)	NA
BRIP1	missense_variant	NM_032043.3:c.475A>G	1	0.000168833	1	0.00026983	0	0	0	0	0	0	0	0	∞(0.06-∞,0.36694)	∞(0.09-∞,0.36074)	NA
BRIP1	missense_variant	NM_032043.3:c.550G>T	2	0.000337667	1	0.00026983	0	0	6	0.000459172	25	0.000439483	158	0.00026778	0.74(0.07-4.11, 1)	0.59(0.01-4.85, 1)	NA
BRIP1	splice_region_variant	NM_032043.3:c.627+5G>A	4	0.000675333	2	0.00053967	0	0	2	0.000153057	2	3.51587E-05	2	3.3897E-06	4.41(0.63-48.8, 0.36694)	3.53(0.26-48.66, 0.36074)	NA
BRIP1	splice_region_variant	NM_032043.3:c.627+6T>C	2	0.000337667	0	0	2	0.00224719	0	0	0	0	0	0	∞(0.41-∞,0.36694)	NA	∞(2.76-∞,0.21442)
BRIP1	missense_variant	NM_032043.3:c.641G>C	1	0.000168833	1	0.00026983	0	0	1	7.65287E-05	2	3.51587E-05	7	1.1864E-05	2.21(0.03-172.97, 0.57702)	3.53(0.04-276.22, 0.58964)	NA
BRIP1	missense_variant	NM_032043.3:c.728T>C	1	0.000168833	1	0.00026983	0	0	4	0.000306115	8	0.000140635	60	0.00010169	0.55(0.01-5.57, 1)	0.88(0.02-8.91, 1)	NA
BRIP1	missense_variant	NM_032043.3:c.774G>C	2	0.000337667	1	0.00026983	0	0	0	0	0	0	1	1.6948E-06	∞(0.41-∞,0.36694)	∞(0.09-∞,0.36074)	NA
BRIP1	missense_variant	NM_032043.3:c.790C>T	12	0.002026	5	0.00134916	2	0.00224719	12	0.000918344	129	0.002267733	1584	0.0026846	2.21(0.91-5.38, 0.36694)	1.47(0.41-4.48, 0.80454)	2.45(0.27-11.03, 1)
BRIP1	missense_variant	NM_032043.3:c.897G>A	2	0.000337667	2	0.00053967	0	0	0	0	0	0	0	0	∞(0.41-∞,0.36694)	∞(0.66-∞,0.36074)	NA
BRIP1	missense_variant	NM_032043.3:c.956T>C	1	0.000168833	1	0.00026983	0	0	0	0	0	0	0	0	∞(0.06-∞,0.36694)	∞(0.09-∞,0.36074)	NA
CDH1	missense_variant	NM_004360.5:c.1143G>T	2	0.000337667	0	0	1	0.0011236	0	0	0	0	0	0	∞(0.41-∞,0.36694)	NA	∞(0.38-∞,0.47826)
CDH1	missense_variant	NM_004360.5:c.1336G>A	1	0.000168833	0	0	0	0	0	0	0	0	1	1.6948E-06	∞(0.06-∞,0.36694)	NA	NA
CDH1	missense_variant	NM_004360.5:c.1360G>A	2	0.000337667	2	0.00053967	0	0	0	0	2	3.51587E-05	50	8.4741E-05	∞(0.41-∞,0.36694)	∞(0.66-∞,0.36074)	NA
CDH1	missense_variant	NM_004360.5:c.1417G>A	5	0.000844167	2	0.00053967	3	0.00337079	2	0.000153057	10	0.000175793	87	0.00014745	5.52(0.9-57.9, 0.3335)	3.53(0.26-48.66, 0.36074)	22.08(2.53-263.64, 0.21442)
CDH1	missense_variant	NM_004360.5:c.1774G>T	1	0.000168833	1	0.00026983	0	0	5	0.000382643	6	0.000105476	11	1.8643E-05	0.44(0.01-3.94, 0.72736)	0.71(0.01-6.3, 1)	NA
CDH1	missense_variant	NM_004360.5:c.1817A>T	1	0.000168833	0	0	1	0.0011236	0	0	0	0	0	0	∞(0.06-∞,0.36694)	NA	∞(0.38-∞,0.47826)
CDH1	missense_variant	NM_004360.5:c.1873C>T	1	0.000168833	0	0	0	0	0	0	0	0	0	0	∞(0.06-∞,0.36694)	NA	NA
CDH1	missense_variant	NM_004360.5:c.1876T>G	1	0.000168833	0	0	0	0	0	0	0	0	3	5.0845E-06	∞(0.06-∞,0.36694)	NA	NA
CDH1	missense_variant	NM_004360.5:c.1930G>A	2	0.000337667	2	0.00053967	0	0	0	0	0	0	7	1.1864E-05	∞(0.41-∞,0.36694)	∞(0.66-∞,0.36074)	NA
CDH1	missense_variant	NM_004360.5:c.1939C>G	1	0.000168833	1	0.00026983	0	0	0	0	0	0	1	1.6948E-06	∞(0.06-∞,0.36694)	∞(0.09-∞,0.36074)	NA
CDH1	missense_variant	NM_004360.5:c.2005C>T	1	0.000168833	1	0.00026983	0	0	0	0	0	0	2	3.3897E-06	∞(0.06-∞,0.36694)	∞(0.09-∞,0.36074)	NA
CDH1	missense_variant	NM_004360.5:c.2017C>A	1	0.000168833	0	0	0	0	0	0	6	0.000105476	96	0.0001627	∞(0.06-∞,0.36694)	NA	NA
CDH1	missense_variant	NM_004360.5:c.2202A>T	2	0.000337667	1	0.00026983	1	0.0011236	0	0	1	1.75793E-05	20	3.3897E-05	∞(0.41-∞,0.36694)	∞(0.09-∞,0.36074)	∞(0.38-∞,0.47826)
CDH1	missense_variant	NM_004360.5:c.2307G>C	1	0.000168833	0	0	0	0	0	0	0	0	0	0	∞(0.06-∞,0.36694)	NA	NA
CDH1	missense_variant	NM_004360.5:c.2335C>T	2	0.000337667	2	0.00053967	0	0	0	0	0	0	13	2.2033E-05	∞(0.41-∞,0.36694)	∞(0.66-∞,0.36074)	NA
CDH1	missense_variant	NM_004360.5:c.2350C>T	1	0.000168833	1	0.00026983	0	0	0	0	2	3.51587E-05	4	6.7793E-06	∞(0.06-∞,0.36694)	∞(0.09-∞,0.36074)	NA
CDH1	missense_variant	NM_004360.5:c.2359G>T	1	0.000168833	1	0.00026983	0	0	0	0	0	0	0	0	∞(0.06-∞,0.36694)	∞(0.09-∞,0.36074)	NA
CDH1	missense_variant	NM_004360.5:c.2363C>T	2	0.000337667	2	0.00053967	0	0	0	0	0	0	0	0	∞(0.41-∞,0.36694)	∞(0.66-∞,0.36074)	NA
CDH1	missense_variant	NM_004360.5:c.2390A>G	1	0.000168833	0	0	0	0	0	0	0	0	4	6.7793E-06	∞(0.06-∞,0.36694)	NA	NA
CDH1	splice_region_variant	NM_004360.5:c.2440-6_2440-4delCTT	2	0.000337667	2	0.00053967	0	0	1	7.65287E-05	6	0.000105476	53	8.9826E-05	4.41(0.23-259.98, 0.36694)	7.05(0.37-415.12, 0.36074)	NA
CDH1	missense_variant	NM_004360.5:c.2474C>T	5	0.000844167	4	0.00107933	1	0.0011236	1	7.65287E-05	3	5.2738E-05	59	9.9995E-05	11.03(1.23-520.75, 0.19367)	14.11(1.4-692.59, 0.20697)	14.69(0.19-1141.88, 0.77983)
CDH1	missense_variant	NM_004360.5:c.2515G>A	1	0.000168833	0	0	0	0	0	0	4	7.03173E-05	20	3.3897E-05	∞(0.06-∞,0.36694)	NA	NA
CDH1	missense_variant	NM_004360.5:c.2561A>T	1	0.000168833	1	0.00026983	0	0	0	0	0	0	0	0	∞(0.06-∞,0.36694)	∞(0.09-∞,0.36074)	NA
CDH1	missense_variant	NM_004360.5:c.2597G>T	1	0.000168833	1	0.00026983	0	0	0	0	0	0	0	0	∞(0.06-∞,0.36694)	∞(0.09-∞,0.36074)	NA
CDH1	missense_variant	NM_004360.5:c.304G>T	1	0.000168833	1	0.00026983	0	0	0	0	2	3.51587E-05	4	6.7793E-06	∞(0.06-∞,0.36694)	∞(0.09-∞,0.36074)	NA
CDH1	splice_region_variant	NM_004360.5:c.388-8C>T	2	0.000337667	2	0.00053967	0	0	2	0.000153057	7	0.000123055	43	7.2878E-05	2.21(0.16-30.45, 0.64306)	3.53(0.26-48.66, 0.36074)	NA
CDH1	missense_variant	NM_004360.5:c.455A>G	2	0.000337667	1	0.00026983	1	0.0011236	0	0	0	0	3	5.0845E-06	∞(0.41-∞,0.36694)	∞(0.09-∞,0.36074)	∞(0.38-∞,0.47826)
CDH1	splice_region_variant	NM_004360.5:c.532-19dupT	1	0.000168833	1	0.00026983	0	0	0	0	0	0	0	0	∞(0.06-∞,0.36694)	∞(0.09-∞,0.36074)	NA
CDH1	missense_variant	NM_004360.5:c.616A>T	1	0.000168833	0	0	0	0	0	0	0	0	0	0	∞(0.06-∞,0.36694)	NA	NA
CDH1	missense_variant	NM_004360.5:c.937G>C	2	0.000337667	1	0.00026983	0	0	0	0	0	0	0	0	∞(0.41-∞,0.36694)	∞(0.09-∞,0.36074)	NA
CDH1	in-frame_insertion	NM_004360.5:c.984_986dupGGT	1	0.000168833	0	0	0	0	0	0	0	0	0	0	∞(0.06-∞,0.36694)	NA	NA
CHEK2	missense_variant	NM_007194.4:c.1007A>T	1	0.000168833	1	0.00026983	0	0	0	0	0	0	0	0	∞(0.06-∞,0.36694)	∞(0.09-∞,0.36074)	NA
CHEK2	missense_variant	NM_007194.4:c.1022A>C	5	0.000844167	1	0.00026983	1	0.0011236	1	7.65287E-05	3	5.2738E-05	37	6.2709E-05	11.03(1.23-520.75, 0.19367)	3.53(0.04-276.22, 0.58964)	14.69(0.19-1141.88, 0.77983)
CHEK2	missense_variant	NM_007194.4:c.1039G>A	1	0.000168833	1	0.00026983	0	0	0	0	3	5.2738E-05	40	6.7793E-05	∞(0.06-∞,0.36694)	∞(0.09-∞,0.36074)	NA
CHEK2	missense_variant	NM_007194.4:c.1067C>T	1	0.000168833	1	0.00026983	0	0	0	0	4	7.03173E-05	4	6.7793E-06	∞(0.06-∞,0.36694)	∞(0.09-∞,0.36074)	NA
CHEK2	missense_variant	NM_007194.4:c.1091T>C	1	0.000168833	1	0.00026983	0	0	3	0.000229586	4	7.03173E-05	30	5.0845E-05	0.74(0.01-9.16, 1)	1.18(0.02-14.64, 1)	NA
CHEK2	splice_region_variant	NM_007194.4:c.1096-4T>C	1	0.000168833	0	0	0	0	0	0	8	0.000140635	30	5.0845E-05	∞(0.06-∞,0.36694)	NA	NA
CHEK2	splice_region_variant	NM_007194.4:c.1096-6T>G	1	0.000168833	1	0.00026983	0	0	0	0	0	0	3	5.0845E-06	∞(0.06-∞,0.36694)	∞(0.09-∞,0.36074)	NA
CHEK2	disruptive_in-frame_deletion	NM_007194.4:c.1109_1111delGGC	1	0.000168833	0	0	1	0.0011236	0	0	0	0	0	0	∞(0.06-∞,0.36694)	NA	∞(0.38-∞,0.47826)
CHEK2	missense_variant	NM_007194.4:c.1111C>T	1	0.000168833	0	0	0	0	4	0.000306115	5	8.78966E-05	14	2.3728E-05	0.55(0.01-5.57, 1)	NA	NA
CHEK2	missense_variant	NM_007194.4:c.1175C>T	1	0.000168833	1	0.00026983	0	0	4	0.000306115	7	0.000123055	71	0.00012033	0.55(0.01-5.57, 1)	0.88(0.02-8.91, 1)	NA
CHEK2	missense_variant	NM_007194.4:c.1183G>C	14	0.002363667	8	0.00215866	2	0.00224719	3	0.000229586	5	8.78966E-05	33	5.5929E-05	10.31(2.88-56.01, **0.00317**)	9.42(2.26-55.12, **0.02803**)	9.8(0.82-85.67, 0.47825)
CHEK2	splice_region_variant	NM_007194.4:c.1260-6delA	1	0.000168833	1	0.00026983	0	0	0	0	0	0	0	0	∞(0.06-∞,0.36694)	∞(0.09-∞,0.36074)	NA
CHEK2	missense_variant	NM_007194.4:c.1265G>A	3	0.0005065	2	0.00053967	0	0	0	0	1	1.75793E-05	27	4.576E-05	∞(0.91-∞,0.30332)	∞(0.66-∞,0.36074)	NA
CHEK2	missense_variant	NM_007194.4:c.1283C>T	2	0.000337667	2	0.00053967	0	0	0	0	8	0.000140635	83	0.00014067	∞(0.41-∞,0.36694)	∞(0.66-∞,0.36074)	NA

CHEK2	synonymous_variant	NM_007194.4:c.1287G>A	1	0.000168833	0	0	1	0.0011236	3	0.000229586	8	0.000140635	26	4.4065E-05	0.74(0.01-9.16, 1)	NA	4.9(0.09-61.12, 1)
CHEK2	missense_variant	NM_007194.4:c.1307T>G	1	0.000168833	1	0.00026983	0	0	0	0	0	0	0	0	∞(0.06-∞,0.36694)	∞(0.09-∞,0.36074)	NA
CHEK2	missense_variant	NM_007194.4:c.1312G>T	3	0.0005065	2	0.00053967	0	0	2	0.000153057	52	0.000914125	436	0.00073894	3.31(0.38-39.65, 0.36694)	3.53(0.26-48.66, 0.36074)	NA
CHEK2	missense_variant	NM_007194.4:c.1383C>G	5	0.000844167	4	0.00107933	1	0.0011236	0	0	2	3.51587E-05	19	3.2202E-05	∞(2.02-∞,0.08325)	∞(2.33-∞,0.07719)	∞(0.38-∞,0.47826)
CHEK2	missense_variant	NM_007194.4:c.1421G>A	3	0.0005065	2	0.00053967	0	0	0	0	10	0.000175793	66	0.00011186	∞(0.91-∞,0.30332)	∞(0.66-∞,0.36074)	NA
CHEK2	missense_variant	NM_007194.4:c.1421G>C	1	0.000168833	0	0	0	0	0	0	0	0	0	0	∞(0.06-∞,0.36694)	NA	NA
CHEK2	missense_variant	NM_007194.4:c.1427C>T	30	0.005065001	22	0.00593632	2	0.00224719	14	0.001071401	59	0.00103718	577	0.00097791	4.75(2.44-9.69, **0.00027**)	5.57(2.72-11.78, **0.00024**)	2.1(0.23-9.16, 1)
CHEK2	missense_variant	NM_007194.4:c.1450C>A	2	0.000337667	1	0.00026983	0	0	0	0	1	1.75793E-05	5	8.4741E-06	∞(0.41-∞,0.36694)	∞(0.09-∞,0.36074)	NA
CHEK2	splice_region_variant	NM_007194.4:c.1461+5G>T	2	0.000337667	1	0.00026983	0	0	0	0	0	0	0	0	∞(0.41-∞,0.36694)	∞(0.09-∞,0.36074)	NA
CHEK2	missense_variant	NM_007194.4:c.1522C>G	3	0.0005065	3	0.0008095	0	0	0	0	3	5.2738E-05	7	1.1864E-05	∞(0.91-∞,0.30332)	∞(1.46-∞,0.20697)	NA
CHEK2	missense_variant	NM_007194.4:c.153G>C	2	0.000337667	0	0	1	0.0011236	0	0	0	0	0	0	∞(0.41-∞,0.36694)	NA	∞(0.38-∞,0.47826)
CHEK2	missense_variant	NM_007194.4:c.190G>A	15	0.0025325	8	0.00215866	1	0.0011236	8	0.000612229	34	0.000597697	256	0.00043388	4.14(1.65-11.29, **0.03642**)	3.53(1.15-10.8, 0.23932)	1.84(0.04-13.72, 1)
CHEK2	in-frame_deletion	NM_007194.4:c.246_260delCCAAGAACCTGAGGA	1	0.000168833	1	0.00026983	0	0	2	0.000153057	12	0.000210952	131	0.00022202	1.1(0.02-21.2, 1)	1.76(0.03-33.88, 0.76453)	NA
CHEK2	missense_variant	NM_007194.4:c.323G>C	1	0.000168833	1	0.00026983	0	0	0	0	0	0	0	0	∞(0.06-∞,0.36694)	∞(0.09-∞,0.36074)	NA
CHEK2	missense_variant	NM_007194.4:c.332A>G	1	0.000168833	1	0.00026983	0	0	0	0	0	0	0	0	∞(0.06-∞,0.36694)	∞(0.09-∞,0.36074)	NA
CHEK2	missense_variant	NM_007194.4:c.433C>T	6	0.001013	6	0.001619	1	0.0011236	0	0	6	0.000105476	63	0.00010677	∞(2.60-∞,**0.03642**)	∞(4.16-∞,**0.007953**)	∞(0.38-∞,0.47826)
CHEK2	missense_variant	NM_007194.4:c.434G>A	1	0.000168833	1	0.00026983	0	0	0	0	4	7.03173E-05	18	3.0507E-05	∞(0.06-∞,0.36694)	∞(0.09-∞,0.36074)	NA
CHEK2	missense_variant	NM_007194.4:c.442A>G	1	0.000168833	0	0	1	0.0011236	0	0	0	0	3	5.0845E-06	∞(0.06-∞,0.36694)	NA	∞(0.38-∞,0.47826)
CHEK2	splice_region_variant	NM_007194.4:c.445-7A>C	1	0.000168833	1	0.00026983	0	0	0	0	0	0	0	0	∞(0.06-∞,0.36694)	∞(0.09-∞,0.36074)	NA
CHEK2	missense_variant	NM_007194.4:c.451G>T	1	0.000168833	0	0	0	0	0	0	1	1.75793E-05	0	0	∞(0.06-∞,0.36694)	NA	NA
CHEK2	missense_variant	NM_007194.4:c.470T>C	51	0.008610501	32	0.00863465	10	0.01123596	161	0.012321114	445	0.0078228	2598	0.00440316	0.7(0.5-0.96, 0.30332)	0.7(0.46-1.03, 0.36074)	0.91(0.43-1.73, 1)
CHEK2	missense_variant	NM_007194.4:c.478A>G	1	0.000168833	1	0.00026983	0	0	0	0	1	1.75793E-05	9	1.5253E-05	∞(0.06-∞,0.36694)	∞(0.09-∞,0.36074)	NA
CHEK2	missense_variant	NM_007194.4:c.524T>C	3	0.0005065	1	0.00026983	1	0.0011236	0	0	0	0	0	0	∞(0.91-∞,0.30332)	∞(0.09-∞,0.36074)	∞(0.38-∞,0.47826)
CHEK2	missense_variant	NM_007194.4:c.539G>A	5	0.000844167	4	0.00107933	0	0	1	7.65287E-05	6	0.000105476	71	0.00012033	11.03(1.23-520.75, 0.19367)	14.11(1.4-692.59, 0.20697)	NA
CHEK2	missense_variant	NM_007194.4:c.541C>T	1	0.000168833	1	0.00026983	0	0	1	7.65287E-05	7	0.000123055	69	0.00011694	2.21(0.03-172.97, 0.57702)	3.53(0.04-276.22, 0.58964)	NA
CHEK2	missense_variant	NM_007194.4:c.542G>A	2	0.000337667	1	0.00026983	0	0	2	0.000153057	4	7.03173E-05	20	3.3897E-05	2.21(0.16-30.45, 0.64306)	1.76(0.03-33.88, 0.76453)	NA
CHEK2	missense_variant	NM_007194.4:c.604T>C	6	0.001013	5	0.00134916	1	0.0011236	1	7.65287E-05	1	1.75793E-05	1	1.6948E-06	13.25(1.61-607.6, 0.11911)	17.65(1.97-831.15, 0.07719)	14.69(0.19-1141.88, 0.77983)
CHEK2	synonymous_variant	NM_007194.4:c.612G>T	1	0.000168833	1	0.00026983	0	0	0	0	1	1.75793E-05	1	1.6948E-06	∞(0.06-∞,0.36694)	∞(0.09-∞,0.36074)	NA
CHEK2	missense_variant	NM_007194.4:c.683G>A	1	0.000168833	1	0.00026983	0	0	0	0	0	0	0	0	∞(0.06-∞,0.36694)	∞(0.09-∞,0.36074)	NA
CHEK2	missense_variant	NM_007194.4:c.715G>A	9	0.0015195	6	0.001619	2	0.00224719	0	0	6	0.000105476	134	0.00022711	∞(4.36-∞,**0.00333**)	∞(4.16-∞,**0.007953**)	∞(2.76-∞,0.21442)
CHEK2	missense_variant	NM_007194.4:c.839T>G	3	0.0005065	3	0.0008095	0	0	0	0	0	0	1	1.6948E-06	∞(0.91-∞,0.30332)	∞(1.46-∞,0.20697)	NA
CHEK2	splice_region_variant	NM_007194.4:c.847-17T>C	1	0.000168833	1	0.00026983	0	0	0	0	18	0.000316428	79	0.00013389	∞(0.06-∞,0.36694)	∞(0.09-∞,0.36074)	NA
CHEK2	missense_variant	NM_007194.4:c.851G>A	2	0.000337667	1	0.00026983	0	0	0	0	0	0	0	0	∞(0.41-∞,0.36694)	∞(0.09-∞,0.36074)	NA
CHEK2	missense_variant	NM_007194.4:c.855C>G	1	0.000168833	1	0.00026983	0	0	0	0	1	1.75793E-05	7	1.1864E-05	∞(0.06-∞,0.36694)	∞(0.09-∞,0.36074)	NA
CHEK2	missense_variant	NM_007194.4:c.8G>T	1	0.000168833	1	0.00026983	0	0	0	0	0	0	2	3.3897E-06	∞(0.06-∞,0.36694)	∞(0.09-∞,0.36074)	NA
CHEK2	missense_variant	NM_007194.4:c.980A>G	1	0.000168833	0	0	0	0	2	0.000153057	3	5.2738E-05	20	3.3897E-05	1.1(0.02-21.2, 1)	NA	NA
CHEK2	missense_variant	NM_007194.4:c.991A>G	1	0.000168833	1	0.00026983	0	0	0	0	0	0	0	0	∞(0.06-∞,0.36694)	∞(0.09-∞,0.36074)	NA
PALB2	missense_variant	NM_024675.4:c.1427G>T	3	0.0005065	0	0	0	0	0	0	0	0	0	0	∞(0.91-∞,0.30332)	NA	NA
PALB2	splice_region_variant	NM_024675.4:c.1685-3T>C	1	0.000168833	1	0.00026983	0	0	0	0	1	1.75793E-05	4	6.7793E-06	∞(0.06-∞,0.36694)	∞(0.09-∞,0.36074)	NA
PALB2	missense_variant	NM_024675.4:c.1960A>G	1	0.000168833	0	0	0	0	0	0	0	0	0	0	∞(0.06-∞,0.36694)	NA	NA
PALB2	missense_variant	NM_024675.4:c.2272C>A	1	0.000168833	1	0.00026983	0	0	0	0	0	0	1	1.6948E-06	∞(0.06-∞,0.36694)	∞(0.09-∞,0.36074)	NA
PALB2	missense_variant	NM_024675.4:c.2456A>G	1	0.000168833	1	0.00026983	0	0	0	0	0	0	0	0	∞(0.06-∞,0.36694)	∞(0.09-∞,0.36074)	NA
PALB2	missense_variant	NM_024675.4:c.2564T>C	3	0.0005065	1	0.00026983	1	0.0011236	4	0.000306115	4	7.03173E-05	5	8.4741E-06	1.65(0.24-9.79, 0.73866)	0.88(0.02-8.91, 1)	3.67(0.07-37.14, 1)
PALB2	missense_variant	NM_024675.4:c.2755G>A	1	0.000168833	1	0.00026983	0	0	2	0.000153057	4	7.03173E-05	68	0.00011525	1.1(0.02-21.2, 1)	1.76(0.03-33.88, 0.76453)	NA
PALB2	splice_region_variant	NM_024675.4:c.2835-11A>G	2	0.000337667	2	0.00053967	0	0	0	0	1	1.75793E-05	12	2.0338E-05	∞(0.41-∞,0.36694)	∞(0.66-∞,0.36074)	NA
PALB2	splice_region_variant	NM_024675.4:c.2835-3T>C	1	0.000168833	0	0	1	0.0011236	0	0	0	0	0	0	∞(0.06-∞,0.36694)	NA	∞(0.38-∞,0.47826)
PALB2	missense_variant	NM_024675.4:c.2897T>C	1	0.000168833	1	0.00026983	0	0	0	0	6	0.000105476	32	5.4234E-05	∞(0.06-∞,0.36694)	∞(0.09-∞,0.36074)	NA
PALB2	missense_variant	NM_024675.4:c.3054G>T	2	0.000337667	1	0.00026983	0	0	0	0	0	0	3	5.0845E-06	∞(0.41-∞,0.36694)	∞(0.09-∞,0.36074)	NA
PALB2	missense_variant	NM_024675.4:c.3055G>T	1	0.000168833	0	0	1	0.0011236	0	0	0	0	0	0	∞(0.06-∞,0.36694)	NA	∞(0.38-∞,0.47826)
PALB2	splice_region_variant	NM_024675.4:c.3114-4T>A	1	0.000168833	0	0	0	0	0	0	0	0	0	0	∞(0.06-∞,0.36694)	NA	NA
PALB2	missense_variant	NM_024675.4:c.3146T>C	1	0.000168833	1	0.00026983	0	0	0	0	3	5.2738E-05	22	3.7286E-05	∞(0.06-∞,0.36694)	∞(0.09-∞,0.36074)	NA
PALB2	in-frame_deletion	NM_024675.4:c.3157_3159delGAT	1	0.000168833	1	0.00026983	0	0	0	0	0	0	1	1.6948E-06	∞(0.06-∞,0.36694)	∞(0.09-∞,0.36074)	NA
PALB2	missense_variant	NM_024675.4:c.3203G>A	1	0.000168833	1	0.00026983	0	0	0	0	1	1.75793E-05	6	1.0169E-05	∞(0.06-∞,0.36694)	∞(0.09-∞,0.36074)	NA
PALB2	missense_variant	NM_024675.4:c.3350G>A	1	0.000168833	1	0.00026983	0	0	0	0	0	0	1	1.6948E-06	∞(0.06-∞,0.36694)	∞(0.09-∞,0.36074)	NA
PALB2	splice_region_variant	NM_024675.4:c.3351-6T>C	2	0.000337667	1	0.00026983	0	0	0	0	0	0	48	8.1352E-05	∞(0.41-∞,0.36694)	∞(0.09-∞,0.36074)	NA
PALB2	missense_variant	NM_024675.4:c.3508C>T	1	0.000168833	1	0.00026983	0	0	6	0.000459172	11	0.000193373	82	0.00013898	0.37(0.01-3.03, 0.52042)	0.59(0.01-4.85, 1)	NA
PALB2	missense_variant	NM_024675.4:c.3518C>T	1	0.000168833	1	0.00026983	0	0	0	0	2	3.51587E-05	22	3.7286E-05	∞(0.06-∞,0.36694)	∞(0.09-∞,0.36074)	NA
PALB2	missense_variant	NM_024675.4:c.632A>G	4	0.000675333	4	0.00107933	0	0	0	0	0	0	1	1.6948E-06	∞(1.46-∞,0.18158)	∞(2.33-∞,0.07719)	NA
PALB2	missense_variant	NM_024675.4:c.656A>C	1	0.000168833	1	0.00026983	0	0	0	0	0	0	0	0	∞(0.06-∞,0.36694)	∞(0.09-∞,0.36074)	NA
PALB2	missense_variant	NM_024675.4:c.766A>G	1	0.000168833	1	0.00026983	0	0	0	0	0	0	1	1.6948E-06	∞(0.06-∞,0.36694)	∞(0.09-∞,0.36074)	NA

PTEN	missense_variant	NM_000314.8:c.1061C>A	3	0.0005065	2	0.00053967	0	0	2	0.000153057	18	0.000316428	178	0.00030168	3.31(0.38-39.65, 0.36694)	3.53(0.26-48.66, 0.36074)	NA
PTEN	missense_variant	NM_000314.8:c.450G>T	1	0.000168833	1	0.00026983	0	0	0	0	0	0	0	0	∞(0.06-∞,0.36694)	∞(0.09-∞,0.36074)	NA
PTEN	missense_variant	NM_000314.8:c.700C>T	1	0.000168833	1	0.00026983	0	0	0	0	0	0	5	8.4741E-06	∞(0.06-∞,0.36694)	∞(0.09-∞,0.36074)	NA
PTEN	missense_variant	NM_000314.8:c.956C>T	1	0.000168833	1	0.00026983	0	0	0	0	0	0	0	0	∞(0.06-∞,0.36694)	∞(0.09-∞,0.36074)	NA
RAD51C	frameshift_variant	NM_058216.3:c.1025_1026delAA	1	0.000168833	1	0.00026983	0	0	0	0	0	0	0	0	∞(0.06-∞,0.36694)	∞(0.09-∞,0.36074)	NA
RAD51C	splice_region_variant	NM_058216.3:c.1026+6T>C	1	0.000168833	0	0	1	0.0011236	0	0	0	0	0	0	∞(0.06-∞,0.36694)	NA	∞(0.38-∞,0.47826)
RAD51C	splice_region_variant	NM_058216.3:c.145+3A>G	1	0.000168833	0	0	0	0	0	0	0	0	0	0	∞(0.06-∞,0.36694)	NA	NA
RAD51C	missense_variant	NM_058216.3:c.158C>G	1	0.000168833	0	0	1	0.0011236	0	0	0	0	1	1.6948E-06	∞(0.06-∞,0.36694)	NA	∞(0.38-∞,0.47826)
RAD51C	splice_region_variant	NM_058216.3:c.405-8G>A	1	0.000168833	1	0.00026983	0	0	0	0	0	0	0	0	∞(0.06-∞,0.36694)	∞(0.09-∞,0.36074)	NA
RAD51C	missense_variant	NM_058216.3:c.422A>G	1	0.000168833	1	0.00026983	0	0	0	0	0	0	0	0	∞(0.06-∞,0.36694)	∞(0.09-∞,0.36074)	NA
RAD51C	missense_variant	NM_058216.3:c.425T>C	1	0.000168833	1	0.00026983	0	0	0	0	0	0	0	0	∞(0.06-∞,0.36694)	∞(0.09-∞,0.36074)	NA
RAD51C	missense_variant	NM_058216.3:c.428A>G	1	0.000168833	1	0.00026983	0	0	0	0	9	0.000158214	157	0.00026609	∞(0.06-∞,0.36694)	∞(0.09-∞,0.36074)	NA
RAD51C	missense_variant	NM_058216.3:c.479C>G	1	0.000168833	0	0	1	0.0011236	0	0	0	0	0	0	∞(0.06-∞,0.36694)	NA	∞(0.38-∞,0.47826)
RAD51C	missense_variant	NM_058216.3:c.482A>T	1	0.000168833	1	0.00026983	0	0	0	0	0	0	5	8.4741E-06	∞(0.06-∞,0.36694)	∞(0.09-∞,0.36074)	NA
RAD51C	missense_variant	NM_058216.3:c.523G>A	1	0.000168833	0	0	0	0	2	0.000153057	4	7.03173E-05	28	4.7455E-05	1.1(0.02-21.2, 1)	NA	NA
RAD51C	missense_variant	NM_058216.3:c.578G>A	1	0.000168833	1	0.00026983	0	0	0	0	2	3.51587E-05	7	1.1864E-05	∞(0.06-∞,0.36694)	∞(0.09-∞,0.36074)	NA
RAD51C	missense_variant	NM_058216.3:c.61C>T	2	0.000337667	1	0.00026983	0	0	0	0	0	0	0	0	∞(0.41-∞,0.36694)	∞(0.09-∞,0.36074)	NA
RAD51C	missense_variant	NM_058216.3:c.784T>G	5	0.000844167	3	0.0008095	1	0.0011236	1	7.65287E-05	11	0.000193373	167	0.00028304	11.03(1.23-520.75, 0.19367)	10.58(0.85-553.91, 0.36074)	14.69(0.19-1141.88, 0.77983)
RAD51C	missense_variant	NM_058216.3:c.952G>A	4	0.000675333	2	0.00053967	0	0	0	0	0	0	1	1.6948E-06	∞(1.46-∞,0.18158)	∞(0.66-∞,0.36074)	NA
RAD51D	missense_variant	NM_002878.4:c.131G>A	2	0.000337667	1	0.00026983	0	0	0	0	2	3.51587E-05	22	3.7286E-05	∞(0.41-∞,0.36694)	∞(0.09-∞,0.36074)	NA
RAD51D	missense_variant	NM_002878.4:c.167T>G	1	0.000168833	1	0.00026983	1	0.0011236	2	0.000153057	36	0.000632856	1	1.6948E-06	1.1(0.02-21.2, 1)	1.76(0.03-33.88, 0.76453)	7.35(0.12-141.34, 1)
RAD51D	missense_variant	NM_002878.4:c.180G>T	1	0.000168833	1	0.00026983	0	0	0	0	0	0	2	3.3897E-06	∞(0.06-∞,0.36694)	∞(0.09-∞,0.36074)	NA
RAD51D	initiator_codon_variant	NM_002878.4:c.1A>T	1	0.000168833	0	0	0	0	0	0	0	0	3	5.0845E-06	∞(0.06-∞,0.36694)	NA	NA
RAD51D	missense_variant	NM_002878.4:c.26G>C	4	0.000675333	3	0.0008095	1	0.0011236	5	0.000382643	75	0.00131845	980	0.00166093	1.77(0.35-8.21, 0.55039)	2.12(0.33-10.89, 0.58964)	2.94(0.06-26.31, 1)
RAD51D	missense_variant	NM_002878.4:c.316G>A	1	0.000168833	1	0.00026983	0	0	0	0	0	0	0	0	∞(0.06-∞,0.36694)	∞(0.09-∞,0.36074)	NA
RAD51D	missense_variant	NM_002878.4:c.325C>T	1	0.000168833	1	0.00026983	0	0	0	0	0	0	0	0	∞(0.06-∞,0.36694)	∞(0.09-∞,0.36074)	NA
RAD51D	synonymous_variant	NM_002878.4:c.333C>T	1	0.000168833	1	0.00026983	0	0	0	0	0	0	4	6.7793E-06	∞(0.06-∞,0.36694)	∞(0.09-∞,0.36074)	NA
RAD51D	missense_variant	NM_002878.4:c.334G>A	2	0.000337667	1	0.00026983	0	0	0	0	6	0.000105476	12	2.0338E-05	∞(0.41-∞,0.36694)	∞(0.09-∞,0.36074)	NA
RAD51D	missense_variant	NM_002878.4:c.355T>C	5	0.000844167	5	0.00134916	1	0.0011236	1	7.65287E-05	9	0.000158214	206	0.00034913	11.03(1.23-520.75, 0.19367)	17.65(1.97-831.15, 0.07719)	14.69(0.19-1141.88, 0.77983)
RAD51D	missense_variant	NM_002878.4:c.412A>G	3	0.0005065	1	0.00026983	1	0.0011236	0	0	8	0.000140635	32	5.4234E-05	∞(0.91-∞,0.30332)	∞(0.09-∞,0.36074)	∞(0.38-∞,0.47826)
RAD51D	missense_variant	NM_002878.4:c.422T>C	1	0.000168833	0	0	1	0.0011236	0	0	1	1.75793E-05	18	3.0507E-05	∞(0.06-∞,0.36694)	NA	∞(0.38-∞,0.47826)
RAD51D	missense_variant	NM_002878.4:c.433C>G	1	0.000168833	1	0.00026983	0	0	0	0	0	0	0	0	∞(0.06-∞,0.36694)	∞(0.09-∞,0.36074)	NA
RAD51D	missense_variant	NM_002878.4:c.433C>T	1	0.000168833	1	0.00026983	0	0	1	7.65287E-05	1	1.75793E-05	11	1.8643E-05	2.21(0.03-172.97, 0.57702)	3.53(0.04-276.22, 0.58964)	NA
RAD51D	missense_variant	NM_002878.4:c.493C>T	1	0.000168833	1	0.00026983	0	0	0	0	6	0.000105476	89	0.00015084	∞(0.06-∞,0.36694)	∞(0.09-∞,0.36074)	NA
RAD51D	missense_variant	NM_002878.4:c.712G>T	1	0.000168833	0	0	1	0.0011236	0	0	0	0	0	0	∞(0.06-∞,0.36694)	NA	∞(0.38-∞,0.47826)
RAD51D	missense_variant	NM_002878.4:c.715C>T	1	0.000168833	1	0.00026983	0	0	1	7.65287E-05	3	5.2738E-05	16	2.7117E-05	2.21(0.03-172.97, 0.57702)	3.53(0.04-276.22, 0.58964)	NA
RAD51D	missense_variant	NM_002878.4:c.716G>A	1	0.000168833	0	0	0	0	0	0	3	5.2738E-05	9	1.5253E-05	∞(0.06-∞,0.36694)	NA	NA

RAD51D	missense_variant	NM_002878.4:c.758G>A	2	0.000337667	0	0	1	0.0011236	0	0	1	1.75793E-05	4	6.7793E-06	∞(0.41-∞,0.36694)	NA	∞(0.38-∞,0.47826)
RAD51D	missense_variant	NM_002878.4:c.824G>A	1	0.000168833	1	0.00026983	0	0	0	0	1	1.75793E-05	6	1.0169E-05	∞(0.06-∞,0.36694)	∞(0.09-∞,0.36074)	NA
RAD51D	missense_variant	NM_002878.4:c.872G>A	7	0.001181834	3	0.0008095	2	0.00224719	1	7.65287E-05	4	7.03173E-05	47	7.9657E-05	15.46(1.99-694.4, 0.05)	10.58(0.85-553.91, 0.36074)	29.42(1.53-1713.77, 0.47825)
RAD51D	missense_variant	NM_002878.4:c.878C>T	1	0.000168833	0	0	0	0	0	0	2	3.51587E-05	34	5.7624E-05	∞(0.06-∞,0.36694)	NA	NA
RAD51D	missense_variant	NM_002878.4:c.899G>A	1	0.000168833	1	0.00026983	0	0	1	7.65287E-05	6	0.000105476	28	4.7455E-05	2.21(0.03-172.97, 0.57702)	3.53(0.04-276.22, 0.58964)	NA
RAD51D	splice_region_variant	NM_002878.4:c.904-3C>T	4	0.000675333	1	0.00026983	2	0.00224719	12	0.000918344	50	0.000878966	465	0.00078809	0.74(0.17-2.43, 0.84325)	0.29(0.01-1.99, 0.51762)	2.45(0.27-11.03, 1)
RAD51D	missense_variant	NM_002878.4:c.932T>A	2	0.000337667	2	0.00053967	0	0	9	0.000688758	24	0.000421904	91	0.00015423	0.49(0.05-2.37, 0.57702)	0.78(0.08-3.79, 1)	NA
STK11	missense_variant	NM_000455.5:c.1036G>A	1	0.000168833	1	0.00026983	0	0	0	0	0	0	5	8.4741E-06	∞(0.06-∞,0.36694)	∞(0.09-∞,0.36074)	NA
STK11	missense_variant	NM_000455.5:c.1077C>G	1	0.000168833	1	0.00026983	1	0.0011236	0	0	0	0	0	0	∞(0.06-∞,0.36694)	∞(0.09-∞,0.36074)	∞(0.38-∞,0.47826)
STK11	missense_variant	NM_000455.5:c.113C>T	1	0.000168833	0	0	0	0	0	0	0	0	1	1.6948E-06	∞(0.06-∞,0.36694)	NA	NA
STK11	missense_variant	NM_000455.5:c.1151G>A	3	0.0005065	2	0.00053967	0	0	0	0	0	0	5	8.4741E-06	∞(0.91-∞,0.30332)	∞(0.66-∞,0.36074)	NA
STK11	missense_variant	NM_000455.5:c.1211C>T	3	0.0005065	3	0.0008095	0	0	9	0.000688758	86	0.001511822	1147	0.00194397	0.74(0.13-2.95, 0.81909)	1.18(0.2-4.71, 1)	NA
STK11	missense_variant	NM_000455.5:c.1220A>C	2	0.000337667	0	0	0	0	0	0	0	0	0	0	∞(0.41-∞,0.36694)	NA	NA
STK11	missense_variant	NM_000455.5:c.1225C>T	1	0.000168833	0	0	0	0	1	7.65287E-05	9	0.000158214	46	7.7962E-05	2.21(0.03-172.97, 0.57702)	NA	NA
STK11	missense_variant	NM_000455.5:c.1244G>A	1	0.000168833	0	0	1	0.0011236	0	0	0	0	4	6.7793E-06	∞(0.06-∞,0.36694)	NA	∞(0.38-∞,0.47826)
STK11	missense_variant	NM_000455.5:c.124C>G	1	0.000168833	1	0.00026983	0	0	0	0	0	0	0	0	∞(0.06-∞,0.36694)	∞(0.09-∞,0.36074)	NA
STK11	missense_variant	NM_000455.5:c.1274G>A	1	0.000168833	1	0.00026983	0	0	0	0	1	1.75793E-05	12	2.0338E-05	∞(0.06-∞,0.36694)	∞(0.09-∞,0.36074)	NA
STK11	synonymous_variant	NM_000455.5:c.1301G>A	1	0.000168833	1	0.00026983	0	0	0	0	0	0	1	1.6948E-06	∞(0.06-∞,0.36694)	∞(0.09-∞,0.36074)	NA
STK11	missense_variant	NM_000455.5:c.139G>A	1	0.000168833	1	0.00026983	0	0	0	0	0	0	1	1.6948E-06	∞(0.06-∞,0.36694)	∞(0.09-∞,0.36074)	NA
STK11	missense_variant	NM_000455.5:c.566C>T	1	0.000168833	0	0	0	0	2	0.000153057	5	8.78966E-05	59	9.9995E-05	1.1(0.02-21.2, 1)	NA	NA
STK11	synonymous_variant	NM_000455.5:c.618G>A	2	0.000337667	2	0.00053967	0	0	0	0	1	1.75793E-05	12	2.0338E-05	∞(0.41-∞,0.36694)	∞(0.66-∞,0.36074)	NA
STK11	missense_variant	NM_000455.5:c.631C>T	1	0.000168833	0	0	1	0.0011236	0	0	5	8.78966E-05	83	0.00014067	∞(0.06-∞,0.36694)	NA	∞(0.38-∞,0.47826)
STK11	missense_variant	NM_000455.5:c.632G>A	1	0.000168833	1	0.00026983	0	0	3	0.000229586	5	8.78966E-05	18	3.0507E-05	0.74(0.01-9.16, 1)	1.18(0.02-14.64, 1)	NA
STK11	missense_variant	NM_000455.5:c.721G>A	1	0.000168833	1	0.00026983	0	0	0	0	2	3.51587E-05	15	2.5422E-05	∞(0.06-∞,0.36694)	∞(0.09-∞,0.36074)	NA
STK11	missense_variant	NM_000455.5:c.875A>T	1	0.000168833	1	0.00026983	0	0	0	0	1	1.75793E-05	6	1.0169E-05	∞(0.06-∞,0.36694)	∞(0.09-∞,0.36074)	NA
STK11	missense_variant	NM_000455.5:c.944C>T	2	0.000337667	1	0.00026983	0	0	0	0	1	1.75793E-05	46	7.7962E-05	∞(0.41-∞,0.36694)	∞(0.09-∞,0.36074)	NA
STK11	missense_variant	NM_000455.5:c.992G>A	2	0.000337667	1	0.00026983	1	0.0011236	0	0	7	0.000123055	55	9.3215E-05	∞(0.41-∞,0.36694)	∞(0.09-∞,0.36074)	∞(0.38-∞,0.47826)
TP53	missense_variant	NM_000546.6:c.14A>G	1	0.000168833	1	0.00026983	0	0	3	0.000229586	3	5.2738E-05	6	1.0169E-05	0.74(0.01-9.16, 1)	1.18(0.02-14.64, 1)	NA
TP53	missense_variant	NM_000546.6:c.214C>A	1	0.000168833	1	0.00026983	0	0	0	0	1	1.75793E-05	3	5.0845E-06	∞(0.06-∞,0.36694)	∞(0.09-∞,0.36074)	NA
TP53	missense_variant	NM_000546.6:c.251C>T	1	0.000168833	0	0	0	0	0	0	0	0	0	0	∞(0.06-∞,0.36694)	NA	NA
TP53	in-frame_deletion	NM_000546.6:c.336_350delCTTCTTGCATTCTGG	1	0.000168833	0	0	1	0.0011236	0	0	0	0	0	0	∞(0.06-∞,0.36694)	NA	∞(0.38-∞,0.47826)
TP53	missense_variant	NM_000546.6:c.460G>C	1	0.000168833	0	0	0	0	0	0	0	0	0	0	∞(0.06-∞,0.36694)	NA	NA
TP53	missense_variant	NM_000546.6:c.523C>T	3	0.0005065	0	0	0	0	0	0	1	1.75793E-05	4	6.7793E-06	∞(0.91-∞,0.30332)	NA	NA
TP53	splice_region_variant	NM_000546.6:c.560-11_560-8dupATTG	1	0.000168833	1	0.00026983	0	0	0	0	0	0	0	0	∞(0.06-∞,0.36694)	∞(0.09-∞,0.36074)	NA
TP53	missense_variant	NM_000546.6:c.572C>G	5	0.000844167	3	0.0008095	1	0.0011236	1	7.65287E-05	1	1.75793E-05	3	5.0845E-06	11.03(1.23-520.75, 0.19367)	10.58(0.85-553.91, 0.36074)	14.69(0.19-1141.88, 0.77983)
TP53	synonymous_variant	NM_000546.6:c.666G>T	4	0.000675333	3	0.0008095	0	0	0	0	10	0.000175793	153	0.00025931	∞(1.46-∞,0.18158)	∞(1.46-∞,0.20697)	NA
TP53	splice_region_variant	NM_000546.6:c.672+9T>C	1	0.000168833	1	0.00026983	0	0	0	0	0	0	0	0	∞(0.06-∞,0.36694)	∞(0.09-∞,0.36074)	NA
TP53	missense_variant	NM_000546.6:c.82G>A	1	0.000168833	1	0.00026983	0	0	0	0	0	0	0	0	∞(0.06-∞,0.36694)	∞(0.09-∞,0.36074)	NA
TP53	missense_variant	NM_000546.6:c.847C>T	1	0.000168833	1	0.00026983	0	0	0	0	15	0.00026369	101	0.00017118	∞(0.06-∞,0.36694)	∞(0.09-∞,0.36074)	NA
TP53	splice_region_variant	NM_000546.6:c.97-4A>G	2	0.000337667	1	0.00026983	0	0	0	0	0	0	0	0	∞(0.41-∞,0.36694)	∞(0.09-∞,0.36074)	NA
TP53	splice_region_variant	NM_000546.6:c.97-9C>T	1	0.000168833	0	0	0	0	4	0.000306115	32	0.000562538	184	0.00031185	0.55(0.01-5.57, 1)	NA	NA

Table S10: Blank version of declaration of consent

Anmeldelses nr. 95825 **Samtykkeerklæring V3** **d. 03.03.2023**

Voksne habile

Informeret samtykke til voksne om deltagelse i et sundhedsvidenskabeligt forskningsprojekt, hvori der indgår omfattende kortlægning af arvematerialet.

Forskningsprojektets titel: Forbedret tolkning af genetiske varianter med Functional Genomics

Erklæring fra forsøgspersonen:

Jeg har fået skriftlig og mundtlig information og jeg ved nok om formål, metode, fordele og ulemper til at sige ja til at deltage.

Jeg ved, at det er frivilligt at deltage, og at jeg altid kan trække mit samtykke tilbage uden at miste mine nuværende eller fremtidige rettigheder til behandling.

Jeg er informeret om, at dette er et forskningsprojekt, hvori der indgår omfattende kortlægning af arvematerialet. Jeg er også informeret om, at der i sjældne tilfælde kan blive opdaget ændringer i mine gener, som kan medføre en alvorlig sygdom, der kan forebygges eller behandles. Jeg kan i givet fald blive kontaktet.

Jeg giver samtykke til, at deltage i forskningsprojektet, og at mit biologiske materiale må udtages med henblik på opbevaring i en forskningsbiobank. Jeg har fået en kopi af dette samtykkeark samt en kopi af den skriftlige information om projektet til eget brug.

Forsøgspersonens navn: ____

Dato: ______________ Underskrift: ____

Erklæring fra den, der afgiver information:

Jeg erklærer, at forsøgspersonen har modtaget mundtlig og skriftlig information om forsøget.

Efter min overbevisning er der givet tilstrækkelig information til, at der kan træffes beslutning om deltagelse i forsøget.

Navnet på den, der har afgivet information: ____________________________________

Dato: ______________ Underskrift: ____

Videnskabsetisk Komités Projekt-ID nr. S-20220080

Figure S1: Sashimi plots of the eight distinct variants (nine samples) showing first treated (NMDi) and then untreated with NMD inhibitor. First comes the proband(s) and afterwards the controls. The arrow indecates the position of the gene variant.

A) *ATM* c.1066-6T>G

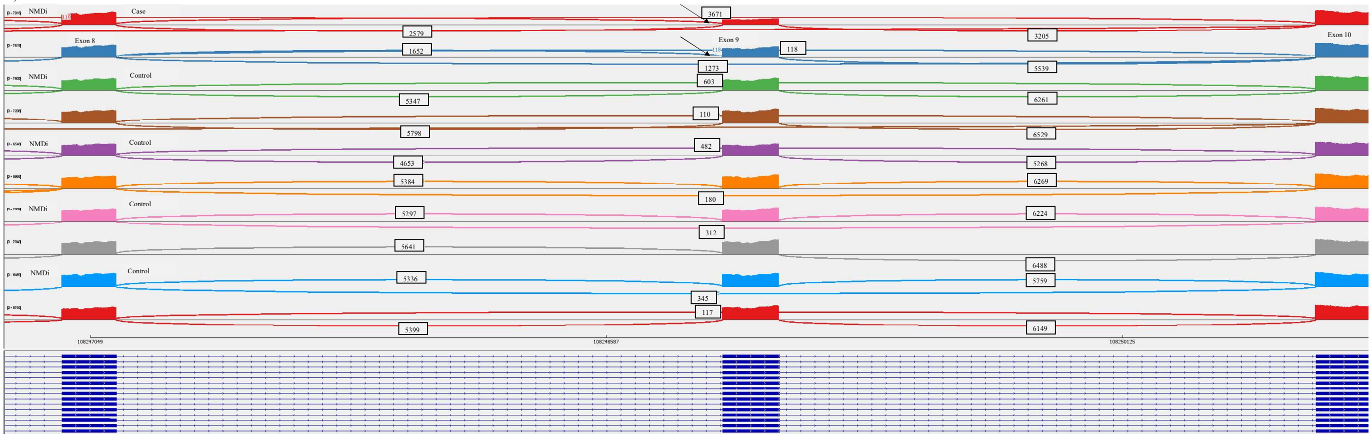

B) *ATM* c.3078-10T>G

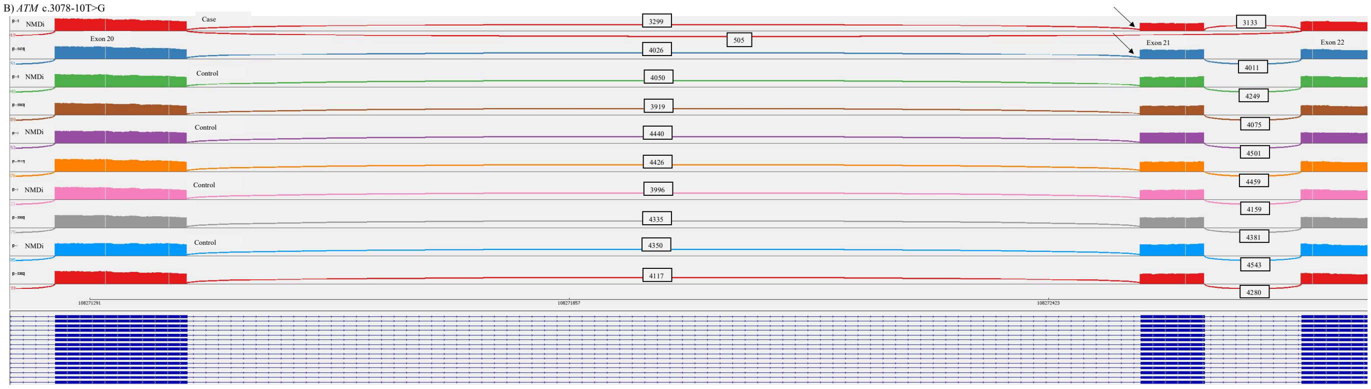

C) *ATM* c.3994-2A>G

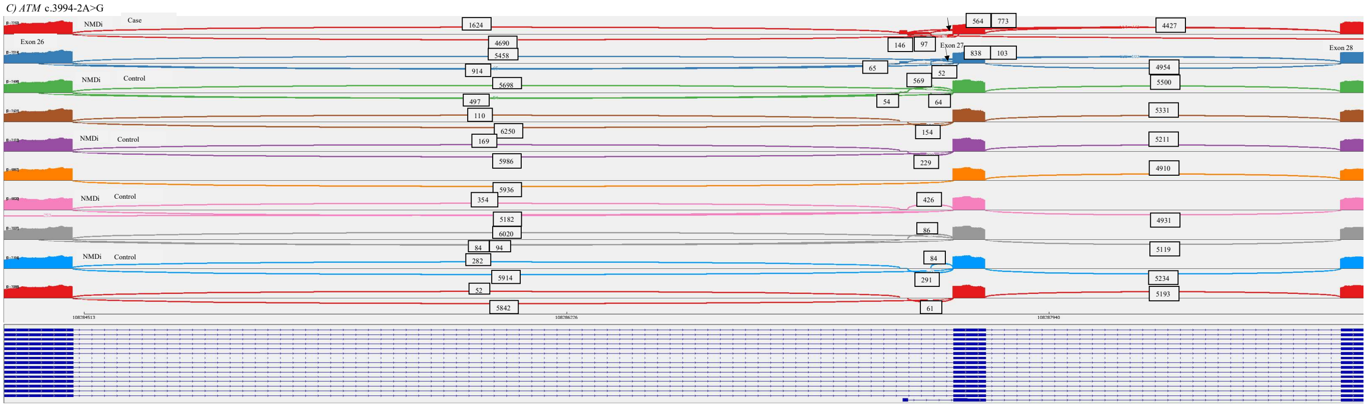

D) *ATM* c.6007-1581A>G

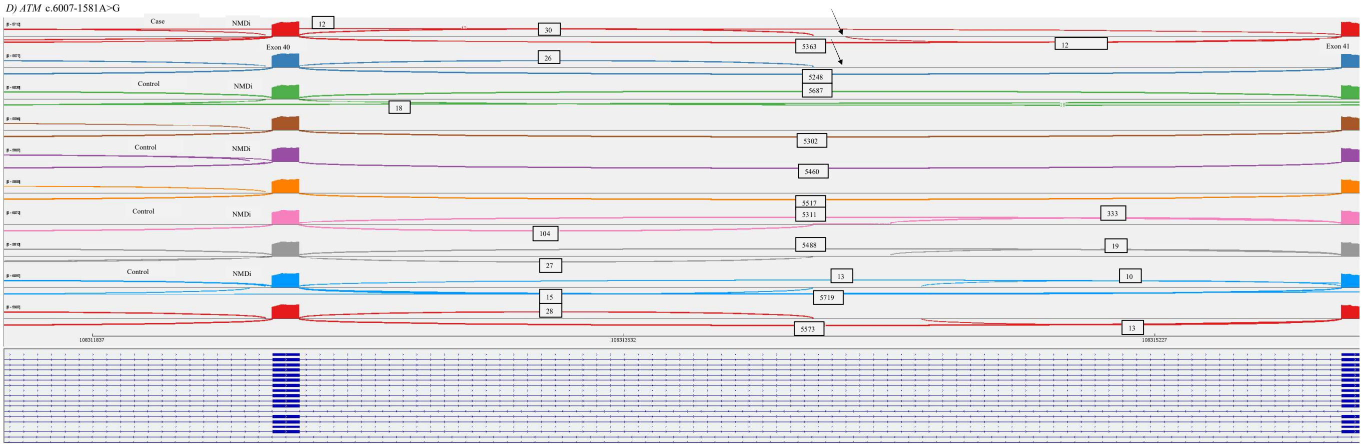

E) *BRIP1* c.2493-10T>A

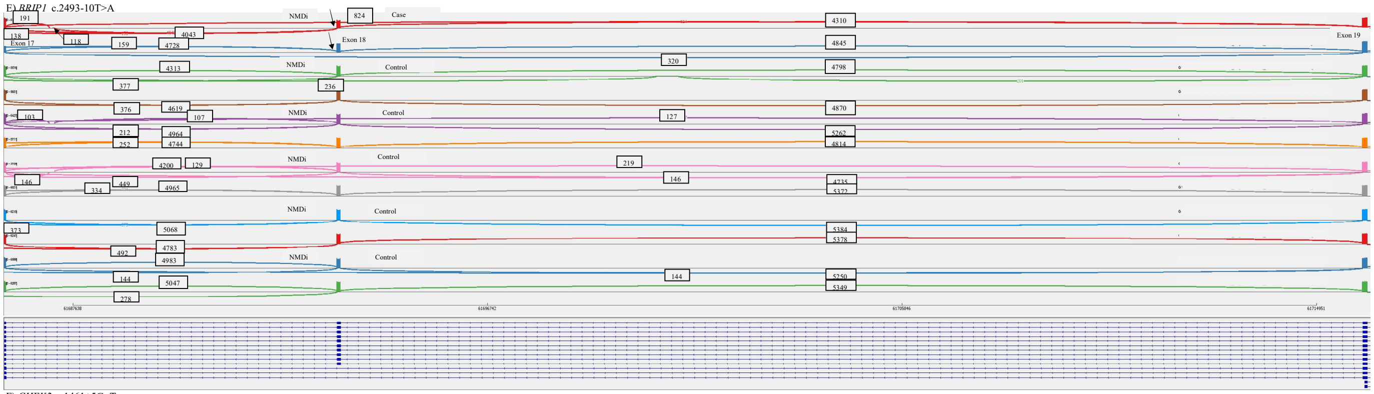

F) *CHEK2* c.1461+5G>T

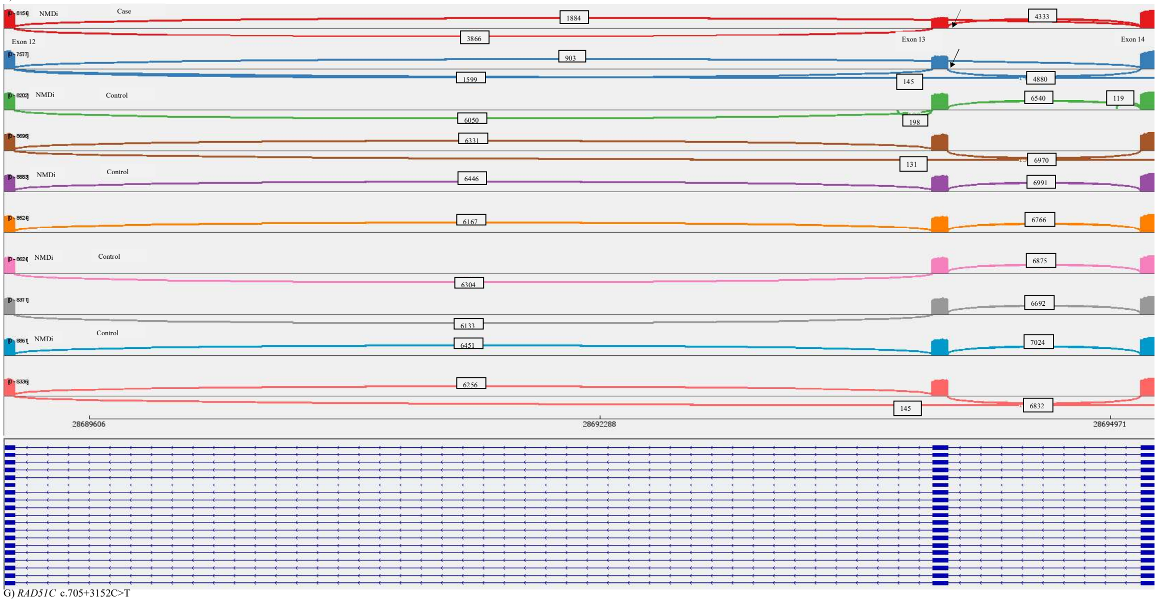

G) *RAD51C* c.705+3152C>T

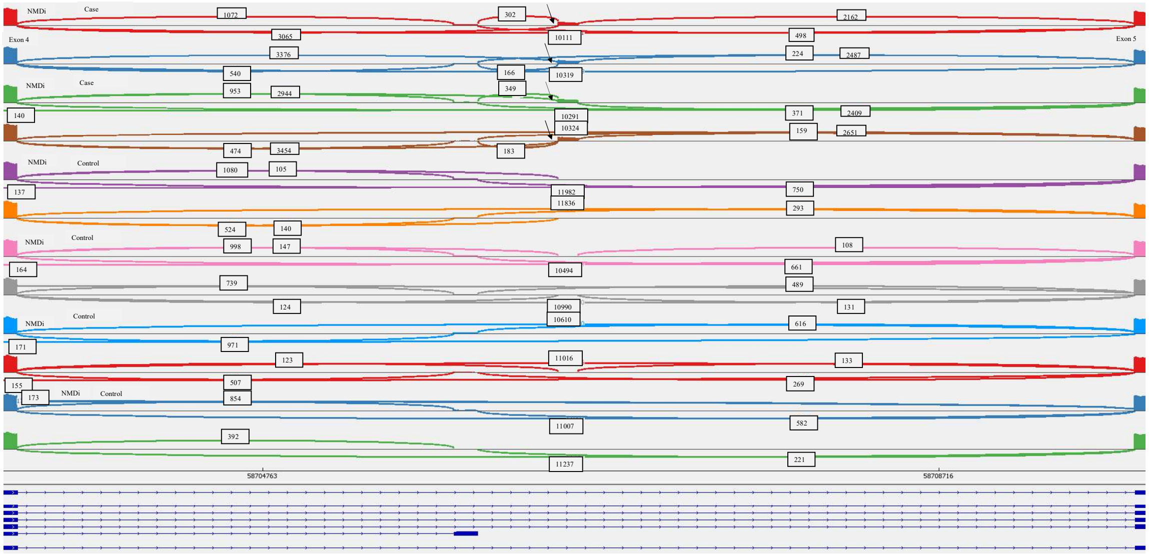

H) *RAD51C* c.837+731A>G

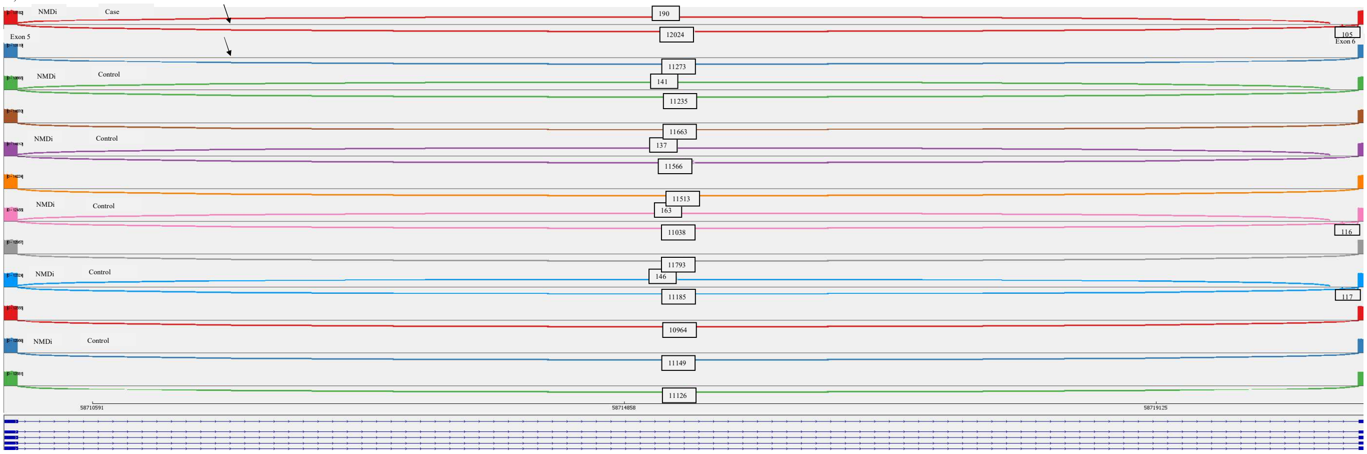

Supplement: Supplementary file 1 [file cancers-17-01819-s001.zip › cancers-3594035-supplementary.pdf]
